# Direct Electrochemical Synthesis of Tetrahydroisoquinolines through Shono-Type Oxidation

**DOI:** 10.1021/acs.orglett.6c01513

**Published:** 2026-07-07

**Authors:** Finn Moeller, Siegfried R. Waldvogel

**Affiliations:** † 28313 Max-Planck-Institute for Chemical Energy Conversion, Department of Electrosynthesis, Stiftstr. 34−36, 45470 Mülheim an der Ruhr, Germany; ‡ Karlsruhe Institute of Technology, Institute of Biological and Chemical Systems - Functional Molecular Systems (IBCS FMS), 76131 Karlsruhe, Germany

## Abstract

Direct electrochemical access to the valuable tetrahydroisoquinoline scaffold has been developed. In a simple undivided setup using inexpensive, reusable electrodes, amides are transformed via Shono-type oxidation into multisubstituted tetrahydroisoquinolines. A broad scope with 34 examples containing electron-poor and heterocyclic substrates was synthesized. Additionally, access to the benzazepine scaffold could also be realized in this way.

## Graphical abstract

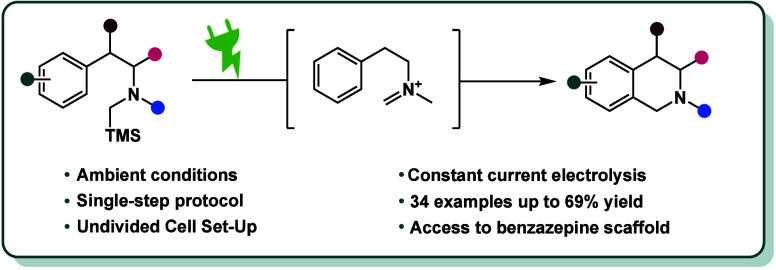

Nitrogen-containing heterocycles can be found in more than 50% of drugs recently approved by the FDA.^1^ Among these falls the compound class of tetrahydroisoquinolines, regarded as a privileged scaffold for active pharmaceutical ingredients (APIs), showing a broad range of biological effects, the most important being antitumor and antibacterial activities.^2^ They are also commonly found in nature, making up a large class of tetrahydroisoquinoline alkaloids to which opioids like morphine belong, including biosynthetic precursor higenamine (**3**), also called norcoclaurine.^3–5^ Several of these tetrahydroisoquinolines can serve as substrates for the anodic conversion to morphine derivatives.^6,7^ Conventional methods for providing access to these compounds rely often on Mannich-type transformations, with the most relevant being the Pictet–Spengler reaction, initially reported in 1911.^8^ In this transformation, an iminium species is formed via condensation of an aldehyde and a phenethylamine in the presence of an acid, followed by electrophilic aromatic substitution. The formation of the reactive intermediate relies on the equilibrium between the protonated imine and the free base; thus, Brønsted and Lewis acids need to be employed for their formation.^9^ For less electron-rich substrates, harsh reaction conditions with strong acids and/or increased temperatures are required.^10,11^ Although this reaction was discovered more than a century ago, efforts to improve the reaction by asymmetric catalysis by the use of enzymes for this transformation continue.^3,12^ Another method for accessing tetrahydroisoquinolines is the Bischler–Napieralski reaction, in which an amide is activated as a nitrilium intermediate, which then reacts with the aromatic core to form dihydroisoquinolines, which can be reduced to the corresponding tetrahydrosioquinoline.^13,14^ An alternative approach to the formation of the key intermediate lies in the oxidation of *N*-alkyl phenylethylamines. Research on this pathway has been limited to reactions with (over)­stoichiometric amounts of oxidants with limited synthetic utility or requiring multistep procedures.^15–17^ In the past decade, electrochemistry has emerged as a tool to drive oxidative transformations, by either direct oxidation of the substrate within the electrolysis cell or decoupled synthesis by platform oxidizers, which could then be previously and electrochemically generated in an electrolysis cell.^18–24^ Recently, the tetrahydroisoquinoline motif has been accessed electrochemically via a reductive approach, hydrogenating isoquinolines with H_2_O as the hydrogen source.^25^ Thus, electrochemistry offers an unexplored possibility to generate the iminium species needed by an oxidative step.^26–28^ Meanwhile, the formation of iminium intermediates is one of the most prominent examples for electrochemical oxidations, pioneered by Shono in the 1980s.^29,30^ By his method, amides, usually protected as carbamates, are oxidized on the anode to form iminium species upon loss of H^+^. Since this reaction is usually conducted in methanol or other alcohols, the iminium intermediate reacts with alcohol-forming α-alkoxylated products. This product is then isolated, and the iminium reactivity can be regenerated in a follow-up reaction, by employing a Lewis acid at low temperatures in aprotic solvents to undergo Mannich-type reactions with a diverse range of nucleophiles (Scheme 1). Since a two-step process for this desired transformation is required, attempts have been made to transform this method into a one-step process. Yoshida achieved great success in this attempt by developing the so-called cation-pool method.^31^ By generating the iminium species in aprotic, apolar solvents at −72 °C, these iminium intermediates are stable enough to be mixed with a suitable nucleophile, which could be done efficiently in microflow reactors.^32,33^ Of course, the downside of this is that an elaborate setup for micromixing and an electrolyzer operated at −72 °C are required, making this approach poorly accessible. Furthermore, chlorinated solvents are used and electrolysis is performed at very low substrate concentrations, resulting in this method being considered far from practical and green.

**Scheme 1:**
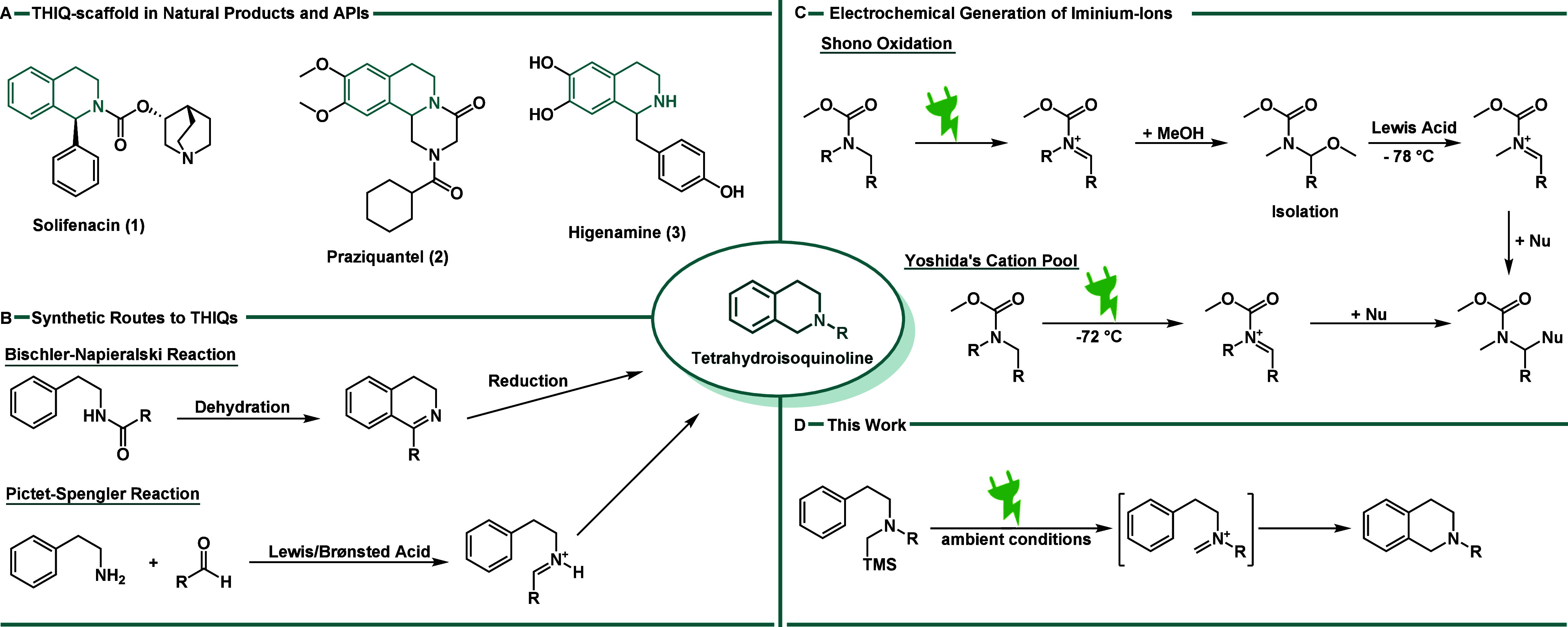
Occurrence of the Tetrahydroisoquinoline Scaffold in Nature and APIs and Synthetic Access to It

We set out to develop a reaction yielding tetrahydroisoquinolines in a one-step fashion with a simple protocol. As a model substrate, we started with the *p*-nitrobenzenesulfonamide of phenylethylamine, substituted with a TMS-methyl group. The TMS group serves as an electroauxiliary, increasing the selectivity of the initial oxidation step by suppressing side reactions like aromatic oxidation and overoxidation of the product.^34^

We first sought to optimize the solvent, which we believe played a very important role in the reaction, since it should not react with the generated iminium ion but also stabilize it to a certain degree to prevent decomposition. First attempts in acetonitrile afforded the desired product, albeit in a low yield of 15% with the dealkylated amide as the dominating side product, stemming from hydrolysis through trace amounts of water (Table 1). This shows that the electrochemical oxidation proceeds as expected, but the intramolecular cyclization is the challenging step. Anhydrous acetonitrile led to only minor improvements, while DCM led to a decrease in the yield of **5a**; therefore, we next tested 1,1,1,3,3,3-hexafluoropropan-2-ol (HFIP), a solvent with unique properties enabling novel types of transformations,^35,36^ leading to a strong increase to a 53% yield of **5a**, while suppressing the formation of the dealkylation product to only 12%. Interestingly, no traces of the corresponding *N*,*O*-acetal, produced by Waldvogel et al. in the case of benzylic oxidations in HFIP and by Lin et al. for amide oxidations in 2,2,2-trifluoroethanol, were detected under these conditions.^37,38^ The improved yield can be attributed to the strong solvating effects of HFIP, prolonging the lifetime of the ionic intermediate.^39–41^ Attempts to change the anode material to BDD or platinum resulted in a drastic decrease in yield. Regarding cathode materials, only platinum gave the product in a comparable yield, since both stainless steel and platinum have a low overpotential for hydrogen evolution. Therefore, we decided to stay with the inexpensive and sustainable electrode materials graphite and stainless steel. We then followed with a screening of the supporting electrolyte, which revealed that tetraethylammonium tetrafluoroborate works best, improving the yield further to 67%. To optimize the amount of applied charge, current density, supporting electrolyte concentration, and substrate concentration, we used a design of experiments approach (details in the Supporting Information), allowing us to gain insight into the influence of the parameters with a small number of experiments,^42–46^ but unfortunately only a minor improvement in the yield was achieved by changing the current density and substrate concentration (entry 11). A control experiment without the TMS group resulted in a drastically diminished yield of 4%, emphasizing the necessity of the electroauxiliary for site selectivity.

**Table 1:**
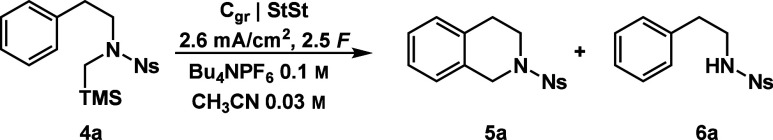
Optimization of the Reaction Parameters

		yield (%)^b^	
entry	deviation from the starting conditions^a^	**5a**	**6a**
1	none	15	62
2	anhydrous CH_3_CN	23	40
3	anhydrous CH_2_Cl_2_	14	21
4	anhydrous 1:1 CH_3_CN/HFIP	18	22
5	HFIP	53	12
6	BDD anode	12	<1
7	Pt anode	10	22
8	0.1 M Bu_4_NBF_4_	58	<1
9	0.1 M Et_4_NBF_4_	67	<1
10	*j* = 1.33 mA/cm^2^, *c*_**4a**_ = 0.02 M	69^c^	<1

a If the deviation led to an increase in the yield of **5a**, the condition was kept.

b Yields were determined by ^1^H NMR with 1,3,5-trimethoxybenzene as an internal standard.

c Yield of the isolated product.

With a 69% yield of the isolated product of our test substrate **5a**, we then explored the scope of this transformation (Scheme 2). First, we explored the substitution pattern of the aromatic core, which we expected to align with the rate of electrophilic aromatic substitution reactions. Slightly electron-donating alkyl groups like *tert*-butyl (**5b**) and methyl (**5c**) at the *para* position were well tolerated, whereas more electron-donating moieties like methoxy (**5d**) resulted in decreased yields. This originates from overoxidation, resulting in larger amounts of oligomeric byproducts. When electron-withdrawing halo substituents were used, the product was obtained in lower yields, accompanied by the HFIP *N,O*-acetal observed for the first time in this transformation. *p*-Bromo compound **5g** was isolated in 25% yield, along with a 32% yield of the HFIP *N*,*O*-acetal (all yields of the acetal are reported in the Supporting Information). Attempts to convert the HFIP-ether into the tetrahydroisoquinoline proved to be unsuccessful, only increasing the yield of the dealkylated amide (see the Supporting Information). Since halo substituents are *ortho*- and *para*-directing due to their mesomeric effect, *meta*-substituted halo-benzenes gave the product in good yields and regioselectivity of up to 64% for fluoro derivative **5h** alongside minor amounts of the separable regioisomer. Also, an iodo substituent, labile under oxidative conditions but of high interest for follow-up reactions, was well tolerated and gave a 43% yield of **5k**. Electron-donating and electron-withdrawing substituents at the *ortho* position gave desired products **5m** and **5n**, respectively, but only in poor yields of 16% and 14%, respectively. Also, trisubstituted benzenes were successfully used in the reaction, with the 3,4-dimethoxy motif commonly found in natural products and being highly important in medicinal chemistry, resulting in a 31% yield of **5o** and a 46% yield of 3,5-dichloro derivative **5p**. Pleasingly, naphthalene could also be employed in this reaction, as well as heterocyclic benzofuran, yielding fused tricyclic scaffolds. Oxidatively labile thiophene derivative **5s** was also successfully synthesized in 17% yield.

**Scheme 2:**
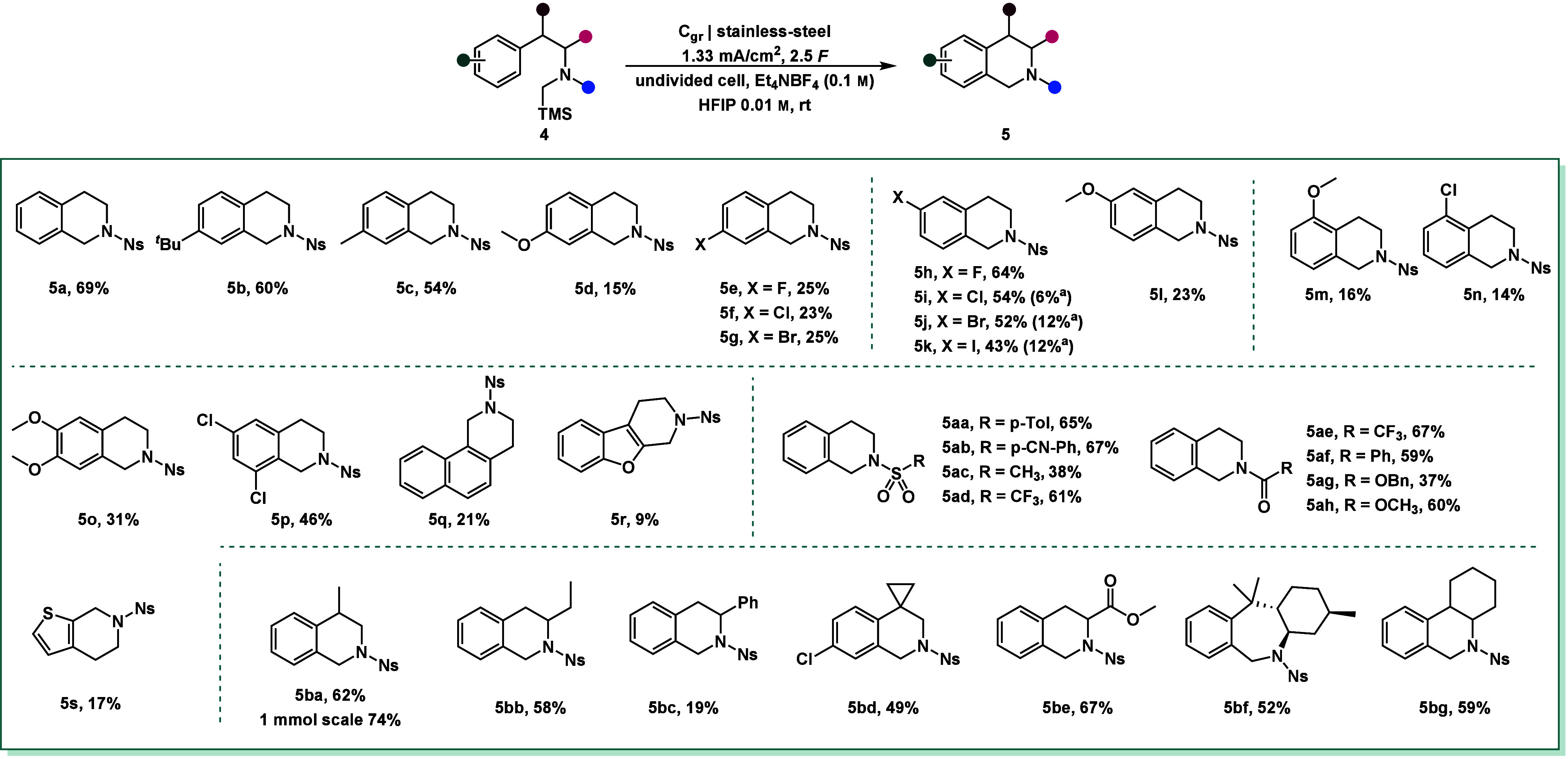
Scope[Fn s2fn66]

a Yield of the 8-halo-tetrahydroisoquinoline.
b All yields refer to yields of the isolated product.

Subsequently, by changing the substituent on the nitrogen, different sulfonamides could be employed in the reaction. Benzenesulfonamides **5aa** and **5ab** were isolated in good yields between 65% and 69%, while mesyl (**5ac**) and triflylamide (**5ad**) groups also gave the desired product, albeit in yields of 38% and 61%, respectively. Carbamates were also suitable for the reaction, with the Cbz group giving product **5ag** in 37% yield and the classic Shono protection group methoxycarbonyl affording a 60% yield of **5ah**. Meanwhile, a Boc group was not tolerated, probably due to decomposition in the slightly acidic solvent. Simple amides like benzamide (**5af**) and trifluoroacetamide (**5ae**) were also well tolerated and resulted in satisfying yields of up to 67%.

Lastly, the influence of different substituents on the ethylene bridge was studied. Alkyl groups worked well in general, besides the 1,2-cyclopropyl derivative, which decomposed under the reaction conditions (see the Supporting Information for more failed substrates). 2-Methyl derivative **5ba** was obtained in 62% yield on a 0.1 mmol scale. By increasing the reaction scale 10-fold, the yield was increased significantly to 74%. Cyclopropane **5bd** gave the desired product in 49% yield, even though the chloro substituent was in the deactivating *para* position, showing that substituents in the benzylic position accelerate the cyclization step. Pleasingly, a substrate derived from phenylalanine could also be employed, giving product **5be** in a good yield of 67%. To our delight, a rather sophisticated seven-membered ring could also be constructed in a good yield of 52%, giving access to the important compound class of benzazepines.^47^ A cyclohexyl derivative gave partially hydrogenated phenanthridine **5bg** in a good yield of 56%.

To conclude, we established a protocol for the electrochemical oxidative synthesis of the important tetrahydroisoquinoline scaffold via an intramolecular cyclization reaction. The straightforward protocol employs a galvanostatic setup with inexpensive reusable electrodes in an undivided cell. The method proceeds efficiently in a single-step, one-pot fashion, and the substrate scope demonstrates high functional group tolerance, with 34 examples synthesized, providing access to highly substituted tetrahydroisoquinolines bearing substituents at nearly all positions of the scaffold. Notably, not only electron-rich but also electron-deficient benzenes could be employed and successfully transformed into the corresponding tetrahydroisoquinolines, which are typically difficult to access under conventional conditions because of their low reactivity. Furthermore, heteroaromatic substrates also participated in the cyclization, yielding structures deviating from the tetrahydroisoquinoline scaffold. In addition, seven-membered benzazepine derivatives could be accessed by using this method, further highlighting the versatility and applicability of the developed protocol.

## Supplementary Material



## Data Availability

The data underlying this study are available in the published article and its Supporting Information.

## Notes

General information, experimental procedures, detailed optimization, and compound characterization with spectra (PDF)
