# Supplementary material for: Direct Electrochemical Synthesis of Tetrahydroisoquinolines through Shono-Type Oxidation
Source: Org Lett. 2026 Jul 7;28(28):8755–9. doi: 10.1021/acs.orglett.6c01513 (PMC13386534; doi:10.1021/acs.orglett.6c01513)
Supplement: Supplementary file 1 [file ol6c01513_si_001.pdf]

Supporting Information

Direct Electrochemical Synthesis of Tetrahydroisoquinolines through Shono-type Oxidation

Finn Moeller, [a] Siegfried R. Waldvogel [a,b]*

[a] *Max Planck Institute for Chemical Energy Conversion, Stiftstraße 34–36, 45470 Mülheim an der Ruhr, Germany.*
[b] *Karlsruhe Institute of Biological and Chemical Systems – Functional Molecular Systems (IBCS FMS), Kaiserstraße 12, 76131 Karslruhe, Germany.*

1 Contents

2 General Information S3
3 Protocols S6
4 Optimization S8
5 List of Unsuccessful Substrates and Yields of HFIP N,O-Acetals S15
6 Cyclic Voltammetry S16
7 Compound Characterization S17
8 Spectral Information S52
9 References S124

2 General Information

2.1 Materials

If not stated otherwise, all reactions were performed at ambient conditions and if they were carried out at elevated temperatures heated with a heating mantle. Chemicals in analytical grade were used as purchased without further purification. Cyclohexane, pentane, *tert*-butyl methyl ether and ethyl acetate used for column chromatography were purchased in HPLC grade.

2.2 Analytical Methods and Devices

NMR

^{1}H NMR, ^{13}C and ^{19}F NMR spectra were recorded at 25°C on a Bruker Ascend Evo 400 MHz NMR spectrometer with a Bruker Prodigy probe (Bruker BioSpin GmbH, Rheinstetten, Germany). All chemical shifts are reported in δ-scale as parts per million [ppm] (multiplicity, coupling constant J, number of protons), relative to the solvent residual peaks as the internal standard. Coupling constants *J* are given in Hertz [Hz]. The following abbreviations were used to describe the signals: s (singlet), d (doublet), t (triplet), q (quartet), dd (doublet of doublet), tt (triplet of triplet), m (multiplet), br (broad signal). The spectra obtained were evaluated with MestReNova 15 (Mestrelab Research S.L., Spain).

Chromatography

Thin layer chromatography was performed using Silica 60 F_{254} on aluminium plates purchased from Merck KGaA (Darmstadt, Germany). An UV lamp (λ = 254 nm, NU-4 KL, Benda, Wiesloch, Germany), (2,4-dinitrophenyl)hydrazine solution (1.0 g 2,4-DNPH in 250 mL 1 M HCl_{aq}) and cer-molybdenum (0.38 g ammonium cerium(IV) sulfate, 0.8 g ammonium molybdate, 100 mL 2 M H_2SO_4) were used for substance detection upon heating. Automated preparative chromatography was performed using a puriFlash™ XS 520 Plus (Interchim, Montluçon, France), using a prepacked puriFlash™ SI-HP silica gel PF-15SIHP-F0040 column (Interchim, Montluçon, France).

High Resolution Mass Spectrometry

High resolution mass spectra were recorded using a Q Exactive Plus (Thermo Fischer Scientific, San Jose, CA, USA), or Q Exactive GC Orbitrap with Trace 1310 GC (Thermo Fischer Scientific, San Jose, CA, USA) spectrometer. For the GC-MS a 30 m long Zebron ZB-1 column (inner diameter: 250 µm; film thickness 0.25 µm) was used. The temperature started at 35 °C and was heated to 320 °C with a temperature rise of 15 °C/min.

Cyclic Voltammetry (CV) Measurements

Cyclic voltammetry was performed using a Metrohm 663 VA Stand equipped with an Autolab type III potentiostat (Metrohm AG, Herisau, Switzerland). WE: graphite rod (d = 2 mm); CE: platinum wire; RE: $Ag/AgNO_3$; Scan rate v = 100 mV/s. Electrolyte: NEt_4BF_4 (0.1 mol/L) in HFIP; c(substrate) = 0.1 mol/L; V = 5 mL. Electrodes have been thoroughly rinsed before and after each measurement with acetonitrile. All data is displayed against the half-wave potential of ferrocene/ferrocenium redox couple (FcH/FcH^{+} ; -0.20 V vs. Ag/AgNO3) as internal reference.

2.3 Electrochemical Set-Up

Power Supply

Galvanostat Rohde & Schwarz - HMP4040 programmable power supply was used as a power supply in all the electrochemical reactions (4 channels per device; max. electric current per channel: 10 A; max. power per channel: 160 W; total output power per device: 384 W; upper terminal voltage limit per channel: 32 V; Rohde & Schwarz GmbH & Co. KG, Munich, Germany). The experiments were performed under galvanostatic conditions using a simple two-electrode reaction setup.

Electrochemical Reactions

Batch-type screening experiments were conducted in an undivided 5 mL Teflon™ electrolysis cells which are commercially available as IKA ElectraSyn Screeningsystem (IKA®-Werke GmbH & Co. KG, Germany).[1] The cell was fitted with a 1 cm x 1 cm magnetic stirring cross and 1 cm x 6 cm electrodes with an interelectrode gap of 5 mm. All electrolysis experiments, unless stated otherwise, were conducted at room temperature (23 °C) and open-air conditions with a constant stirring setting of the stirring plate to 300 rpm.

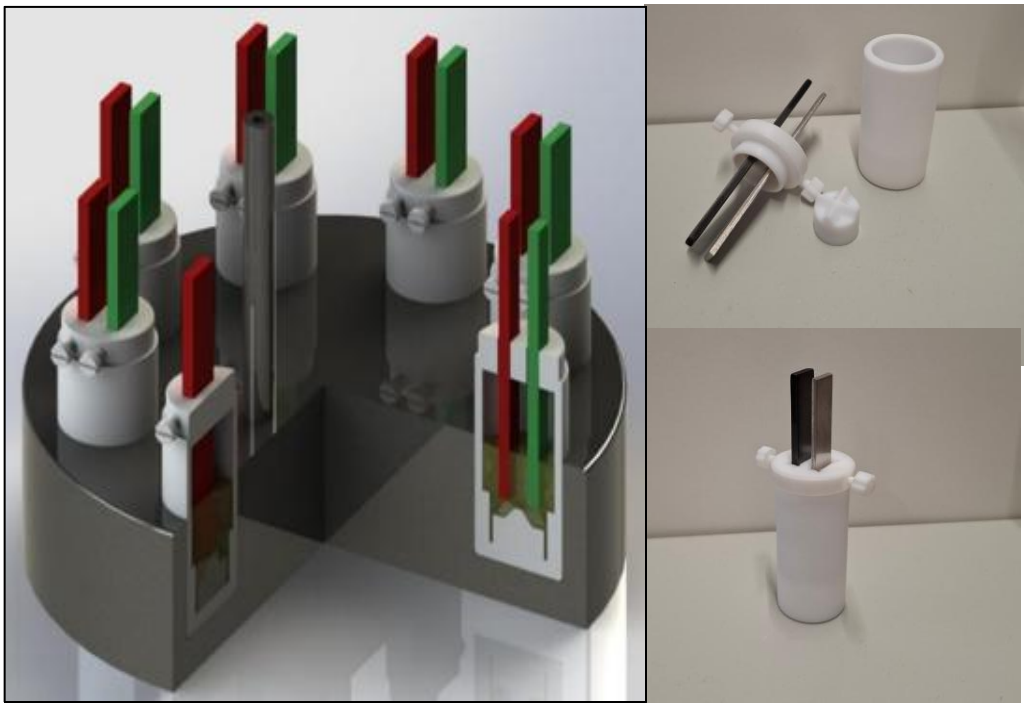

Figure S1. Schematic of the used IKA ElectraSyn Screeningsystem and the disassembled and assembled Teflon cell with stirring bar and electrodes.

A reaction on a 1 mmol-scale was carried out in an undivided, jacketed beaker-type glass cell, equipped with a 3 cm x 3 cm stirring cross and a Teflon stopper, fitted with electrode holders and electrodes in the dimension 2 cm x 6 cm with an interelectrode gap of 1.2 cm. The cell is commercially available at Sigma-Aldrich within the SynLectro™ series.[2]

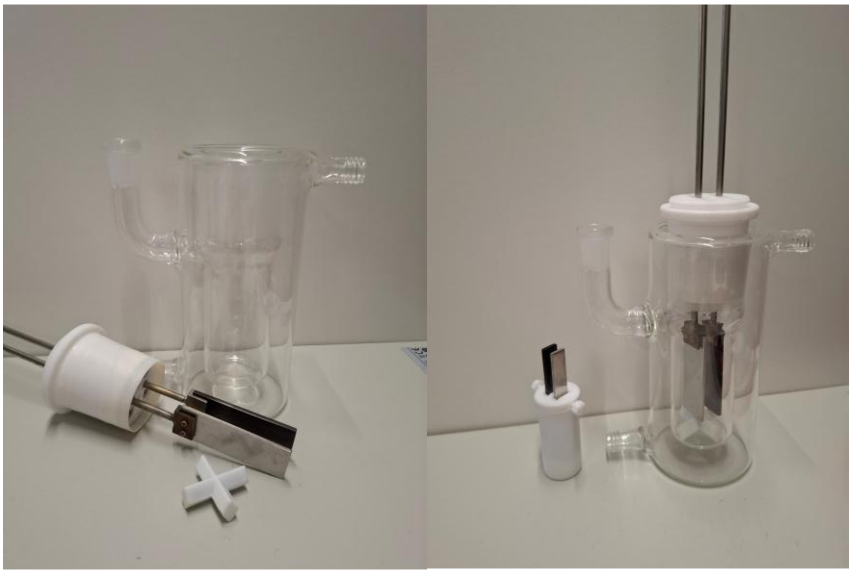

Figure S2. 100 ml beaker-type cell with stirring cross, graphite and stainless-steel electrode disassembled and assembled.

Electrode Materials

All electrodes were cleaned by rinsing with water and acetone after the reaction. Graphite was additionally rubbed with a cleaning tissue until a metallic shine was observed on the surface. Boron-doped diamond (BDD) electrodes were treated prior to electrosynthesis in 20% aqueous sulfuric acid with current density of 10 mA/cm^2 by polarizing subsequently anodically and then cathodically.[2]

Table S1. Listing of used electrodes with specifications and supplier.

**Material**	**Specifications**	**Supplier**
Graphite	highly isostatic, Sigrafine™, V2100	*SGL Carbon*, Bonn, Germany
Stainless-Steel	1.4571	*various suppliers* (metal traders)
Platinum	99.9% Pt, foil, 0.1 mm	*ÖGUSSA*, Vienna, Austria
Nickel	99.9% Ni, sheet	Rheinische Stahlhandel, Germany
Glassy Carbon	Sigradur G	*HTW*, Thierhaupten, Germany
Boron-Doped Diamond (BDD)	15 µm BDD on silicon support	*CONDIAS GmbH*, Itzehoe, Germany
Sigraflex™	Graphite foil, F02012Z	*SGL Carbon*, Meitingen, Germany

3 Protocols

3.1 General Protocol 1

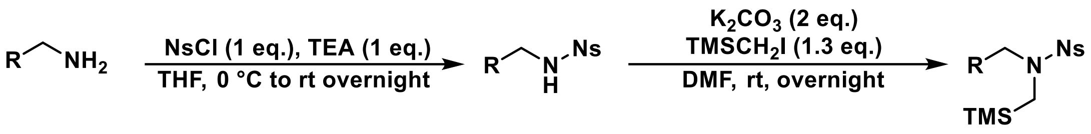

Scheme S1. Reaction scheme for the synthesis of TMS-methyl amides.

2 mmol of amine and 2 mmol of triethylamine (1 eq., 0.28 mL; if instead of the free amine the HCl salt was used, 2 eq. were added) were dissolved in 10 mL of THF and the resulting mixture placed in an ice bath and stirred. After 5 minutes 2 mmol of nosyl chloride (1 eq., 443 mg) were added and the mixture left to stir overnight. Next, 10 mL of 1 M NaOH(aq) were added and the mixture transferred into a separatory funnel and extracted with CH_2Cl_2 (3x20 mL). The combined organic fractions were washed thoroughly with 30 mL of 1 M HCl(aq), dried over $MgSO_4$, filtered and evaporated to dryness under reduced pressure, yielding the crude product which was used without further purification.

1.4 mmol of amide were dissolved in DMF (10 mL) and 2.4 mmol of K_2CO_3 (2 eq., 576 mg) were added and the suspension stirred at rt. After this 1.8 mmol of TMS-methyliodide (1.3 eq., 0.387 g, 0.27 mL) was added by a syringe and stirring continued overnight. Then, the reaction mixture was partitioned between ethyl acetate (20 mL) and brine (20 mL) and the aqueous phase extracted 2 more times with ethyl acetate (20 mL). The combined organic fractions were dried over $MgSO_4$, filtered and evaporated to dryness and purified via automated column chromatography (Cyclohexane: ethyl acetate;5% to 15% ethyl acetate).

3.2 General Protocol 2

The starting material (0.1 mmol) and Et_4NBF_4 (0.5 mmol, 108.5 mg) were added to a 5 mL Teflon vial equipped with a stirrer and dissolved in 5 mL HFIP. The vial was sealed with the lid equipped with a graphite and a stainless-steel electrode (depth of immersion = 2.3 cm; width = 1 cm) and the electrodes connected to a galvanostat and stirring as well as the electrochemical reaction started. After the reaction finished, the mixture was transferred to a round-bottom flask and the solvent evaporated under reduced pressure and collected for redistillation. The crude product was purified via automated column chromatography.

3.3 Electrochemical Oxidation on 1 mmol scale

Compound **4ba** (1 mmol, 407 mg) and Et_4NBF_4 (5 mmol, 1.09 g) were added to a 50 mL beaker-type cell equipped with a stirrer and dissolved in 50 mL HFIP. The cell was sealed with a Teflon stopper equipped with a graphite and a stainless-steel electrode (depth of immersion = 3.7 cm; width = 2 cm) and the electrodes connected to a galvanostat and stirring as well as the electrochemical reaction with a current density of 1.33 mA/cm^2 and an amount of applied charge of 2.5 *F* started. After the reaction finished, the mixture was transferred to a round-bottom flask and the solvent evaporated under reduced pressure and collected for redistillation. The crude product was partitioned between brine (20 mL) and ethyl acetate (20 mL) and the aqueous phase extracted two more times with ethyl acetate

(20 mL). The combined organic fractions were dried over $MgSO_4$, filtered and evaporated to dryness and purified via automated column chromatography (Cy:EA, 4% to 10%) to yield 246.2 mg (0.741 mmol, 74%) of **5ba**.

4 Optimization

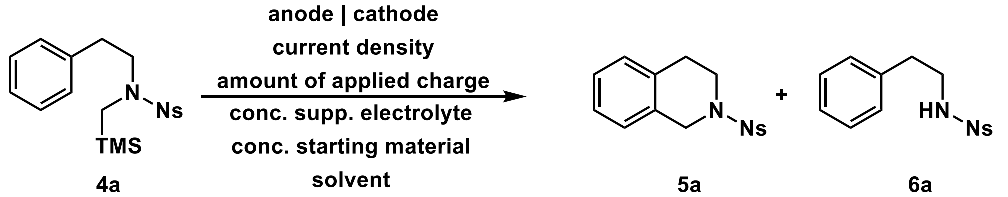

Scheme S2. Electrochemical synthesis of tetrahydroquinolines with all reaction parameters.

4.1 Influence of the Solvent

Table S2. Conversion of **4a** and yield of **5a** and **6a** in dependence of the solvent. Constant parameters were: Anode: Graphite; cathode: stainless-steel; j = 2.6 mA/cm^2; Q = 2.5 F, Bu_4NPF_6 (0.1 M) conc. of starting material 0.04 M. Yields were determined by ^{1}H NMR with 1,3,5-trimethoxybenzene as internal standard.

Solvent	Conversion of **4a**	Yield **5a**	Yield **6a**
Acetonitrile	90%	15%	62%
Anh. acetonitrile	80%	23%	40%
HFIP	90%	53%	12%
HFIP/anh. acetonitrile (1/9)	76%	13%	21%
HFIP/anh. acetonitrile (1/1)	62%	18%	22%
Anh. CH_2Cl_2	73%	14%	21%
HFIP/anh. CH_2Cl_2 (1/16)	91%	13%	70%

4.2 Influence of the Electrode Material

Table S3. Conversion of **4a** and yield of **5a** and **6a** in dependence of the electrode material. Constant parameters were: j = 2.6 mA/cm^2; Q = 2.5 F, Bu_4NPF_6 (0.1 M) conc. of starting material 0.04 M in HFIP. Yields were determined by ^{1}H NMR with 1,3,5-trimethoxybenzene as internal standard.

Anode|Stainless-Steel	Conversion of **4a**	Yield **5a**	Yield **6a**
C_{gr}	90%	53%	12%
BDD	36%	12%	n.d.
Sigraflex	67%	28%	26%
Pt	69%	10%	22%
Glassy Carbon	28%	4%	n.d.
C_{gr}|Cathode			
Stainless-Steel	90%	53%	12%
Pt	84%	57%	<1%
Ni	91%	50%	<1%
C_{gr}	59%	9%	<1%
Stainless-Steel wire	62%	9%	<1%

4.3 Influence of the Supporting Electrolyte

Table S4. Conversion of **4a** and yield of **5a** and **6a** in dependence of the supporting electrolyte. Constant parameters were: Anode: Graphite; cathode: stainless-steel; j = 2.6 mA/cm^2; Q = 2.5 F, supporting electrolyte (0.1 M), conc. of starting material in HFIP 0.04 M. Yields were determined by ^{1}H NMR with 1,3,5-trimethoxybenzene as internal standard.

Supporting Electrolyte	Conversion of **4a**	Yield **5a**	Yield **6a**
Bu_4NPF_6	90%	53%	12%
Bu_4NBF_4	89%	58%	<1%
Et_4NBF_4	89%	67%	<1%
Et_4NPF_6	64%	22%	<1%
Et_4NBr	0%	n.d.	n.d.
Et_4NTfO	69%	19%	<1%
Et_4NTf_2N	66%	23%	<1%
Et_3MeNBF_4	93%	67%	<1%
Me_4NBF_4	56%	18%	<1%

4.4 Influence of Temperature and Stirring Rate

Table S5. Conversion of **4a** and yield of **5a** and **6a** in dependence of temperature and stirring rate. The temperature given, is the temperature of the screening setup measured with a thermometer. Constant parameters were: Anode: Graphite; cathode: stainless-steel; j = 2.6 mA/cm^2; Q = 2.5 F, Et_4NBF_6 (0.1 M), conc. of starting material in HFIP 0.04 M. Yields were determined by ^{1}H NMR with 1,3,5-trimethoxybenzene as internal standard.

Temperature	Stirring Rate	Conversion of **4a**	Yield **5a**	Yield **6a**
15 °C	300 rpm	70%	21%	20%
23 °C (rt)	300 rpm	89%	67%	<1%
35 °C	300 rpm	90%	67%	10%
45 °C	300 rpm	90%	60%	10%
23 °C	Off	59%	11%	<1%
23 °C	300 rpm	89%	67%	<1%
23 °C	600 rpm	77%	32%	<1%
15 °C	300 rpm	70%	21%	20%
23 °C	300 rpm	89%	67%	<1%

4.5 Influence of the Continuous Parameters with Design of Experiments

The Design of Experiments (DoE) was conducted using the software StatEase and consisted of a 2^4 experimental plan with 2 repetitions and 3 centre points. Due to multifactor interactions starpoints and additional experiments to improve resolution were added.

Table S6. Parameters and their +/- level used in the Design of Experiments.

Parameter	- level	+ level
Current Density [mA/cm^2]	0.67	4.00
Amount of Appl. Charge [*F*]	1.5	3.0
Concentration of **4a** [mol/L]	0.01	0.04
Concentration of Et_4NBF_4 [mol/L]	0.05	0.15

Table S7. Conducted experiments in the full-factorial design with star-points and additional randomized experiments. Constant parameters were: Anode: Graphite; cathode: stainless-steel; solvent: HFIP. Yields were determined by ^{1}H NMR with 1,3,5-trimethoxybenzene as internal standard.

Experiment	*j* [mA/cm^2]	*Q* [*F*]	*c*(**4a**) [mol/L]	*c*(Et_4NBF_4) [mol/L]	Yield **5a**
1	4	1.5	0.04	0.05	38%
2	4	3	0.01	0.05	26%
3	0.67	1.5	0.01	0.15	25%
4	0.67	1.5	0.04	0.15	40%
5	4	1.5	0.01	0.05	26%
6	4	1.5	0.04	0.15	25%
7	0.67	1.5	0.04	0.05	35%
8	0.67	3	0.04	0.05	45%
9	0.67	1.5	0.04	0.15	35%
10	0.67	3	0.04	0.15	55%
11	4	3	0.01	0.05	25%
12	2.33	2.25	0.025	0.1	53%
13	4	1.5	0.04	0.05	38%
14	4	3	0.04	0.15	41%
15	0.67	3	0.01	0.05	75%
16	4	3	0.04	0.15	43%
17	0.67	3	0.01	0.05	70%
18	0.67	1.5	0.04	0.05	37%
19	0.67	1.5	0.01	0.05	51%
20	4	3	0.01	0.15	29%
21	0.67	1.5	0.01	0.05	55%
22	2.33	2.25	0.025	0.1	54%
23	0.67	1.5	0.01	0.15	43%
24	0.67	3	0.01	0.15	63%
25	4	3	0.04	0.05	36%
26	4	1.5	0.01	0.15	22%
27	4	3	0.04	0.05	33%
28	4	1.5	0.04	0.15	21%

29	2.33	2.25	0.025	0.1	58%
30	0.67	3	0.04	0.15	44%
31	4	1.5	0.01	0.15	22%
32	0.67	3	0.01	0.15	68%
33	4	3	0.01	0.15	19%
34	4	1.5	0.01	0.05	16%
35	0.67	3	0.04	0.05	53%
36	4	2.25	0.025	0.15	42%
37	2.33	3	0.01	0.11	38%
38	0.67	3	0.025	0.095	63%
39	2.33	1.5	0.025	0.05	51%
40	4	2.16	0.028	0.05	35%
41	4	2.23	0.011	0.10	23%
42	0.67	1.5	0.026	0.09	44%
43	2.33	1.5	0.025	0.15	60%
44	2.33	1.5	0.01	0.10	32%
45	2.67	2.33	0.04	0.10	66%
46	2.33	2.25	0.025	0.1	59%
47	2.33	1.5	0.025	0.15	55%
48	4	2.16	0.028	0.05	36%
49	0.67	3	0.025	0.095	52%
50	1.33	2.5	0.02	0.01	70%
51	1.33	2.5	0.02	0.075	63%
52	1.33	2.5	0.02	0.05	63%
53	1.33	2.5	0.02	0.025	57%

Table S8. Fit Statistics.

Statistical Parameter	Value
Standard Deviation	7.21
Mean	43.92
R^2	0.8494
Adjusted R^2	0.7866
Predicted R^2	0.6560
Adequate Precision	12.7876

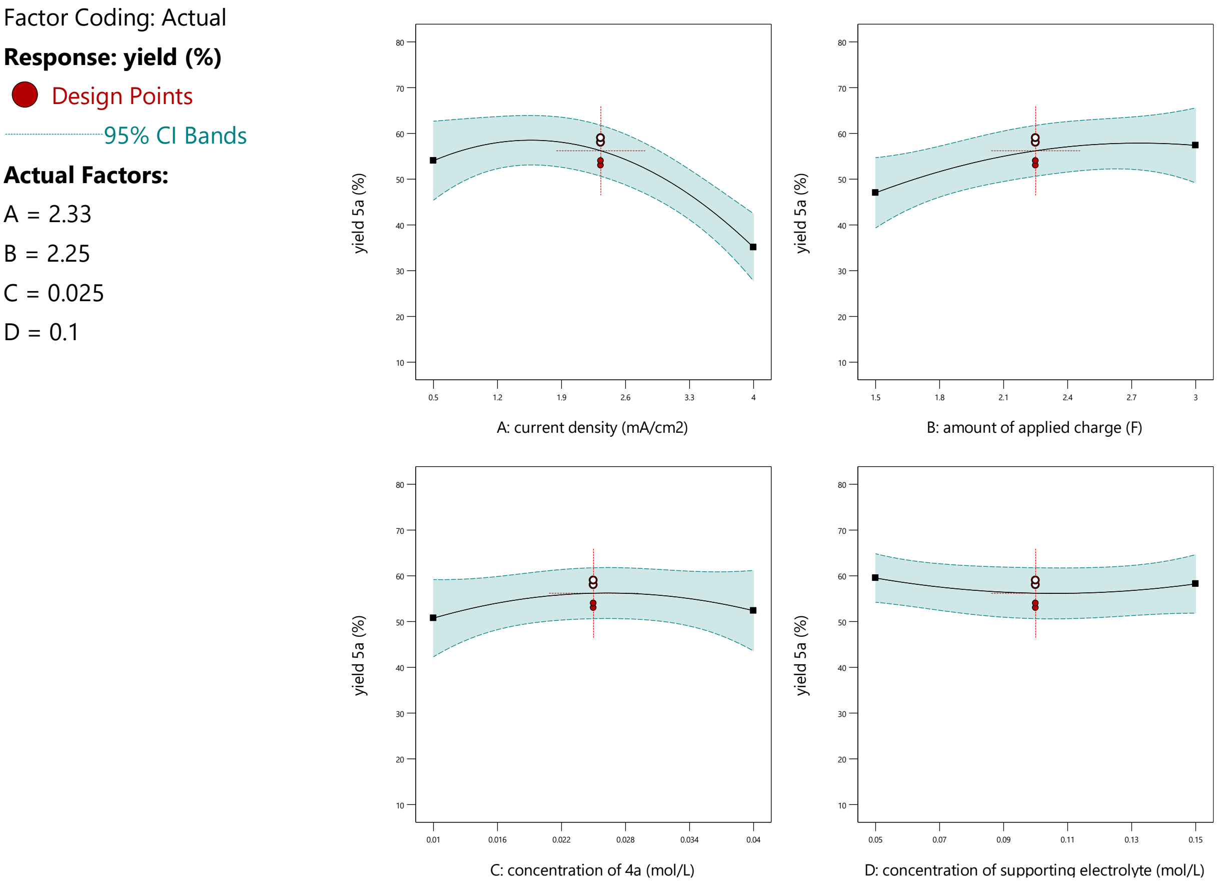

Figure S3. Main effect plot of the paramters in dependency of the yield of **5a**.

Due to the curvature of the parameter 'concentration of supporting electrolyte' a linear screening of this parameter in- and outside the covered parameter space was conducted.

Table S9. Conversion of **4a** and yield of **5a** in dependence of the concentration of supporting electrolyte. Constant parameters were: Anode: graphite; cathode: stainless-steel; j = 1.7 mA/cm^2; Q = 2.5 F, Et_4NBF_6 (X M), conc. of starting material in HFIP 0.02 M Yields were determined by ^{1}H NMR with 1,3,5-trimethoxybenzene as internal standard.

Conc. of Et_4NBF_4	Conversion of **4a**	Yield **5a**
0.200 M	90%	66%
0.175 M	92%	69%
0.150 M	92%	65%
0.125 M	89%	59%
0.100 M	94%	70%
0.075 M	94%	63%
0.050 M	94%	63%
0.025 M	90%	57%

Additionally, a small linear screening of the amount of applied charge was performed, with stainless-steal and platinum as the cathode material.

Table S10. Conversion of **4a** and yield of **5a** in dependence of the amount of applied charge and cathode material. Constant parameters were: Anode: graphite; j = 1.7 mA/cm^2; Q = 2.5 F, Et_4NBF_6 (0.1 M), conc. of starting material in HFIP 0.02 M Yields were determined by ^{1}H NMR with 1,3,5-trimethoxybenzene as internal standard.

Amount of Applied Charge	Cathode	Conversion of **4a**	Yield **5a**
2.5 *F*	Stainless-Steel	94%	70%
2.5 *F*	Pt	93%	73%
2.75 *F*	Stainless-Steel	94%	66%
2.75 *F*	Pt	95%	65%
3.0 *F*	Stainless-Steel	96%	62%
3.0 *F*	Pt	97%	59%

4.6 Control Reactions

To investigate the influence of dissolved oxygen and the necessity of the TMS electro auxiliary control reactions were performe

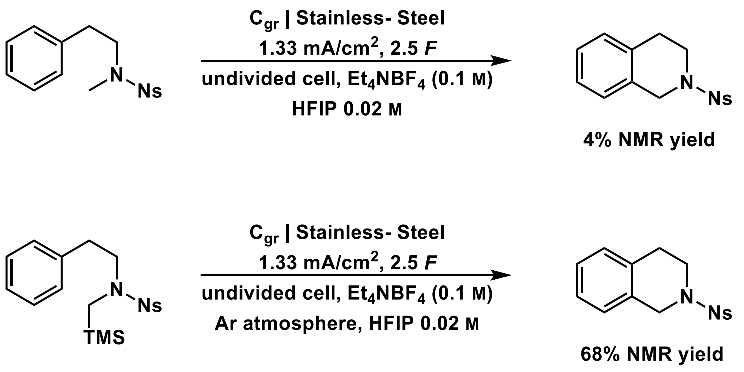

Scheme S3. Control experiments under optimized conditions to investigate the influence of dissolved oxygen and the electro auxiliary.

If no electro auxiliary was used, the yield dropped drastically and resulted in a poor mass balance. A reaction under Ar atmosphere with purged solvent resulted in the same yield as under open-air conditions, showing no influence of dissolved oxygen on the reaction.

4.7 Attempts to Convert HFIP-*N,O*-Acetal to the Tetrahydroisoquinoline

Attempts were made to convert the HFIP-*N,O*-acetal, detected when very electron-deficient aromatics were used in the reaction, to the desired product **5a** by regenerating the iminium-species through addition of an acid.

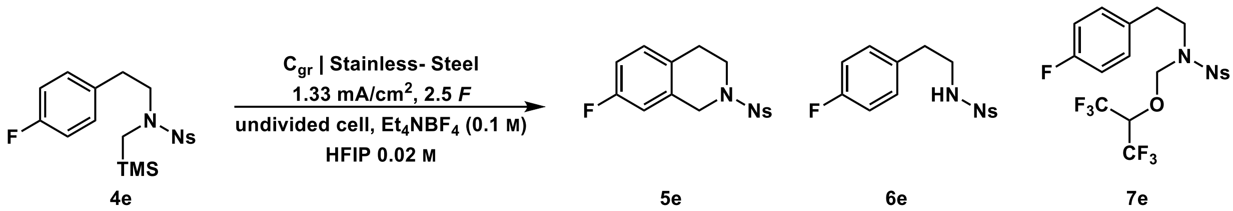

Table S11. Conversion of **4e** and yield of **5e**, **6e** and **7e** with different additions of acids. Yields were determined by ^{1}H NMR with 1,3,5-trimethoxybenzene as internal standard.

Deviation	Conversion of **4e**	Yield **5e**	Yield **6e**	Yield **7e**
no	91%	25%	12%	32%
4 eq. of TFA before electrolysis	93%	19%	11%	34%
4 eq. of TFA after electrolysis	92%	75%	14%	25%
10 eq. of TFA after electrolysis and 40 °C for 4 h	>99%	24%	37%	<1%
10 eq. of AcOH after electrolysis and 40 °C for 4 h	92%	27%	11%	39%
Solvent switch to 1,2-dichloroethane after electrolysis; 10 eq. TFA, 40 °C, 4 h	94%	26%	26%	15%

5 List of Unsuccessful Substrates and Yields of HFIP N,O-Acetals

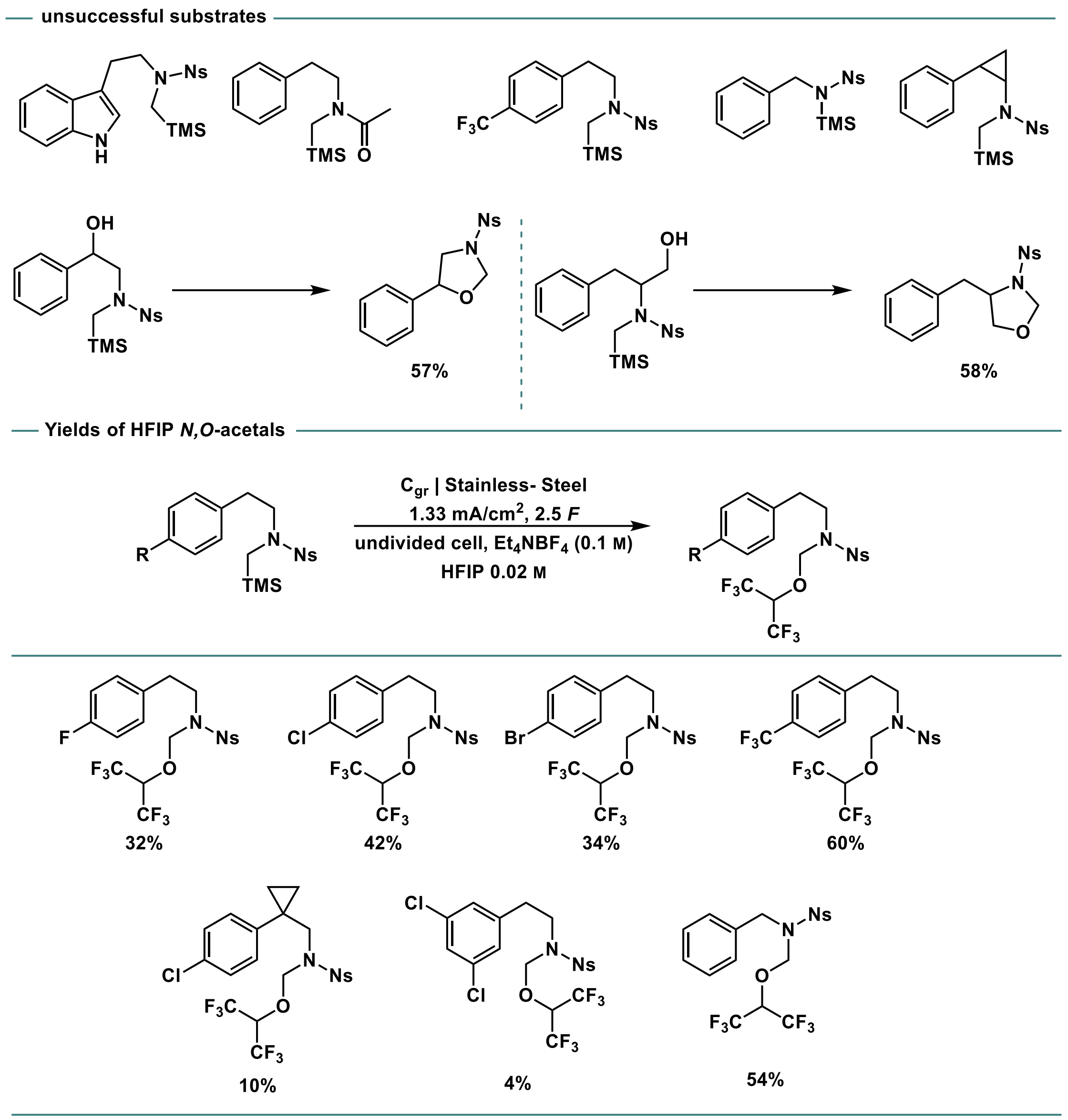

Scheme S4. List of unsuccessful substrates and yields of the isolated HFIP *N,O*-acetal.

6 Cyclic Voltammetry

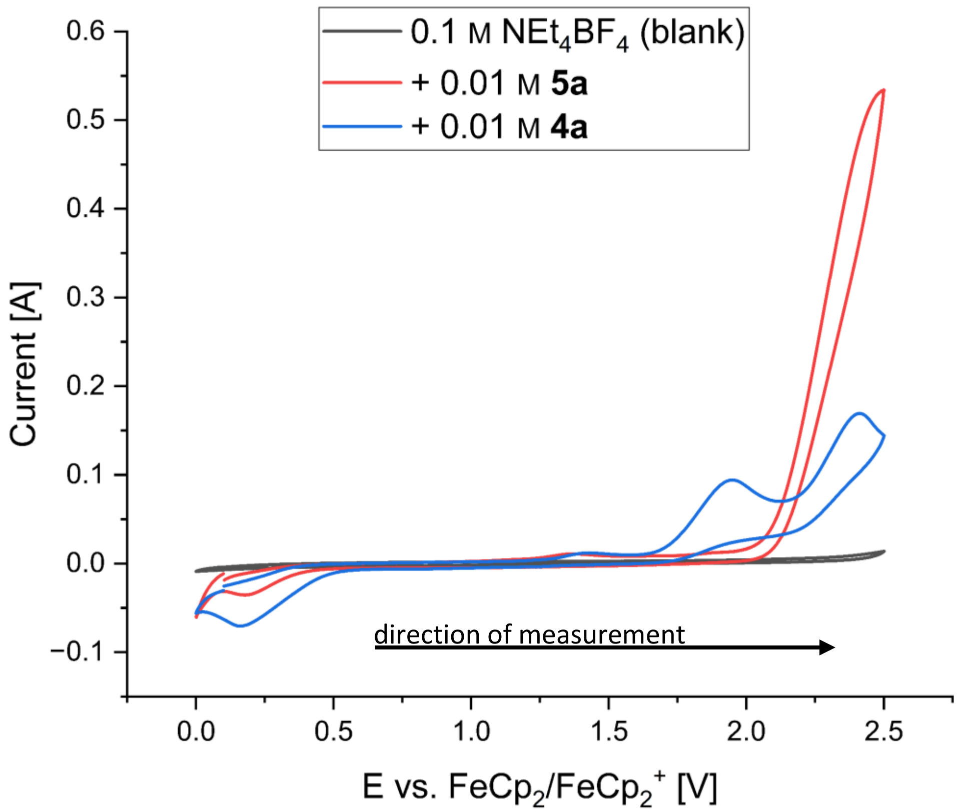

Figure S4. Cyclic voltammogram of: 0.1 M NEt_4BF_4 in HFIP (black), 0.1 M NEt_4BF_4 and 0.01 M **4a** in HFIP (blue) and 0.1 M NEt_4BF_4 and 0.01 M **5a** in HFIP (red). The working electrode was a graphite rod, the counter electrode a platinum wire and the reference electrode was Ag/AgCl and the potentials are referenced against the redox couple $FeCp_2/FeCp_2^+$ measured in 0.1 M NEt_4BF_4 in HFIP.

7 Compound Characterization

7.1 Characterisation of 4-nitro-*N*-phenethyl-*N*-((trimethylsilyl)methyl)-benzenesulfonamide (4a)

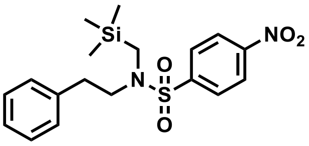

4a

5.38 mmol of amine and 5.38 mmol of triethylamine (1 eq.) were dissolved in 20 mL of THF and the resulting mixture placed in an ice bath and stirred. After 5 minutes 5.38 mmol of nosyl chloride (1 eq.) were added and the mixture left to stir overnight. Next, 20 mL of 1 M NaOH(aq.) were added and the solution transferred into a separatory funnel and extracted with CH_2Cl_2 (3x20 mL). The combined organic fractions were washed thoroughly with 30 mL of 1 M HCl(aq.), dried over $MgSO_4$, filtered and evaporated to dryness under reduced pressure, yielding the crude product which was used without further purification.
NaH (301.5 mg, 7.54 mmol) was added to a flame-dried flask under Ar atmosphere and suspended in DMF (25 mL). The amide (1.65 g, 5.38 mmol) was then added at 0 °C and the mixture stirred for 1 h at rt with an argon stream. After this 7.0 mmol of the iodide (1.3 eq) was added at rt and the mixture stirred at 50 °C overnight. Then, the reaction mixture was partitioned between ethyl acetate (20 mL) and brine (20 mL) and the aqueous phase extracted 2 more times with ethyl acetate (20 mL). The combined organic extracts were dried over $MgSO_4$ and evaporated to dryness. The crude product was purified by column chromatography yielding 941 mg (2.4 mmol, 44%) of the pure product as an off-white solid.

^{1}H NMR (400 MHz, CD_2Cl_2) δ = 8.36–8.29 (m, 2H), 8.00–7.92 (m, 2H), 7.32–7.17 (m, 3H), 7.16–7.09 (m, 2H), 3.48–3.28 (m, 2H), 2.82–2.75 (m, 2H), 2.72 (s, 2H), 0.15 (s, 9H).

^{13}C NMR (101 MHz, CD_2Cl_2) δ = 150.3, 145.1, 138.4, 129.1, 129.0, 128.9, 127.0, 124.6, 52.3, 39.3, 34.8, -1.8.

HR-MS (ESI) $[M+Na]^+$: 415.1121; calc. for $C_{18}H_{24}N_2O_4SSiNa$ 415.1118.

7.2 Characterisation of *N*-(4-(*tert*-butyl)phenethyl)-4-nitro-*N*-((trimethyl-silyl)methyl)benzenesulfonamide (4b)

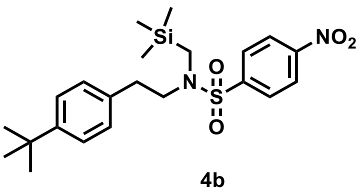

4b

The compound was synthesized according to GP1 starting from 1.55 mmol of amine, yielding 583 mg (1.3 mmol, 84%) of the product as an off-white solid.

^{1}H NMR (400 MHz, DMSO-d$_6$) δ = 8.44–8.36 (m, 2H), 8.12–8.04 (m, 2H), 7.33–7.25 (m, 2H), 7.12–7.05 (m, 2H), 3.31–3.26 (m, 2H), 2.73–2.63 (m, 4H), 1.24 (s, 9H), 0.10 (s, 9H).

^{13}C NMR (101 MHz, DMSO-d$_6$) δ = 149.7, 148.8, 143.5, 134.9, 128.8, 128.3, 125.3, 124.6, 51.6, 38.5, 34.1, 33.2, 31.1, -1.7.

HR-MS (ESI+) [M+Na]$^+$: 471.1748, calc. for $C_{22}H_{32}N_2O_4SSiNa$ 471.1744.

7.3 Characterisation of *N*-(4-methyl-phenethyl)-4-nitro-*N*-((trimethylsilyl)-methyl)benzenesulfonamide (4c)

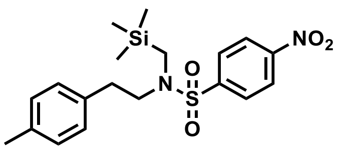

4c

The compound was synthesized according to GP1 starting from 2.18 mmol of amine, yielding 820 mg (2.02 mmol, 93%) of the product as an off-white solid.

^{1}H NMR (400 MHz, DMSO-d$_6$) δ = 8.42–8.34 (m, 2H), 8.08–8.00 (m, 2H), 7.10–6.99 (m, 4H), 3.30 (m, 2H), 2.73 (s, 2H), 2.70–2.63 (m, 2H), 2.24 (s, 3H), 0.11 (s, 9H).

^{13}C NMR (101 MHz, DMSO-d$_6$) δ = 149.6, 143.7, 135.4, 134.8, 129.0, 128.6, 128.4, 124.5, 51.5, 38.2, 33.0, 20.5, -1.7.

HR-MS (ESI+) [M+Na]$^+$: 429.1278, calc. for $C_{19}H_{26}N_2O_4SSiNa$ 429.1275.

7.4 Characterisation of *N*-(4-methoxy-phenethyl)-4-nitro-*N*-((trimethylsilyl)-methyl)benzenesulfonamide (4d)

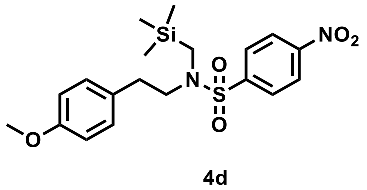

4d

1.49 mmol of amine and 1.49 mmol of triethylamine (1 eq.) were dissolved in 10 mL of THF and the resulting mixture placed in an ice bath and stirred. After 5 minutes 5.38 mmol of nosyl chloride (1 eq.) were added and the mixture left to stir overnight. Next, 10 mL of 1 M NaOH(aq.) were added and the solution transferred into a separatory funnel and extracted with CH_2Cl_2 (3x20 mL). The combined organic fractions were washed thoroughly with 30 mL of 1 M HCl(aq.), dried over $MgSO_4$, filtered and evaporated to dryness under reduced pressure and the crude product used without further purification.

NaH (72 mg, 16.4 mmol) was added to a flame-dried flask under Ar atmosphere and suspended in DMF (10 mL). The amide (0.5 g, 1.49 mmol) was then added at rt and the mixture stirred for 1 h with an argon stream. After this 1.94 mmol of the iodide (1.3 eq) was added and the mixture stirred overnight. Then, the reaction mixture was partitioned between ethyl acetate (20 mL) and brine (20 mL) and the aqueous phase extracted 2 more times with ethyl acetate (20 mL). The combined organic extracts were dried over $MgSO_4$ and evaporated to dryness. The crude product was purified by column chromatography yielding 191 mg (0.45 mmol, 30%) of the pure product as an off-white solid.

^{1}H NMR (400 MHz, CD_2Cl_2) δ = 8.35–8.28 (m, 2H), 7.97–7.89 (m, 2H), 7.07–6.98 (m, 2H), 6.87–6.75 (m, 2H), 3.75 (s, 3H), 3.40–3.31 (m, 2H), 2.75–2.66 (m, 4H), 0.15 (s, 9H).

^{13}C NMR (101 MHz, CD_2Cl_2) δ = 158.8, 150.1, 145.2, 130.2, 129.9, 128.8, 124.6, 114.3, 55.5, 52.4, 39.1, 33.7, -1.5.

HR-MS (ESI+) $[M+Na]^+$: 445.1228, calc. for $C_{19}H_{26}N_2O_5SSiNa$ 445.1224.

7.5 Characterisation of *N*-(4-fluoro-phenethyl)-4-nitro-*N*-((trimethylsilyl)-methyl)benzenesulfonamide (4e)

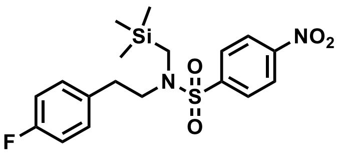

4e

The compound was synthesized according to GP1 starting from 7.18 mmol of amine, yielding 2.25 g (5.49 mmol, 76%) of the product as an off-white solid.

^{1}H NMR (400 MHz, CD_2Cl_2) δ = 8.38–8.30 (m, 2H), 8.00–7.92 (m, 2H), 7.16–7.06 (m, 2H), 7.02–6.92 (m, 2H), 3.39–3.30 (m, 2H), 2.82–2.73 (m, 2H), 2.70 (s, 2H), 0.14 (s, 9H).

^{13}C NMR (101 MHz, DMSO-d_6) δ = 162.1 (d, J = 244.1 Hz), 150.3, 145.0, 134.2 (d, J = 3.3 Hz), 130.6 (d, J = 8.0 Hz), 128.9, 124.6, 115.7 (d, J = 21.3 Hz), 52.3 (d, J = 1.5 Hz)., 39.4, 34.0, -1.5.

^{19}F NMR (376 MHz, CD_2Cl_2) δ = -116.96 (ddd, J = 13.9, 8.3, 4.9 Hz).

HR-MS (ESI+) $[M+Na]^+$: 433.1024, calc. for $C_{18}H_{23}N_2O_4FSSiNa$ 433.1024.

7.6 Characterisation of *N*-(4-chloro-phenethyl)-4-nitro-*N*-((trimethylsilyl)-methyl)benzenesulfonamide (4f)

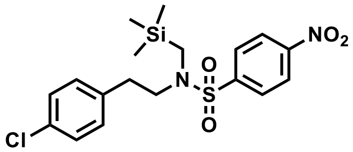

4f

The compound was synthesized according to GP1 starting from 1.22 mmol of amine, yielding 460 mg (1.08 mmol, 89%) of the product as an off-white solid.

^{1}H NMR (400 MHz, CD_2Cl_2) δ = 8.37–8.29 (m, 2H), 7.98–7.90 (m, 2H), 7.28–7.21 (m, 2H), 7.12–7.04 (m, 2H), 3.44–3.32 (m, 2H), 2.82–2.74 (m, 2H), 2.71 (s, 2H), 0.14 (s, 9H).

^{13}C NMR (101 MHz, CD_2Cl_2) δ = 150.3, 145.1, 137.0, 132.8, 130.5, 129.1, 128.9, 124.7, 52.1, 39.4, 34.2, -1.6.

HR-MS (ESI+) $[M+Na]^+$: 449.0730, calc. for $C_{18}H_{23}N_2O_4SSi^{35}ClNa$ 449.0729.

7.7 Characterisation of *N*-(4-bromo-phenethyl)-4-nitro-*N*-((trimethylsilyl)-methyl)benzenesulfonamide (4g)

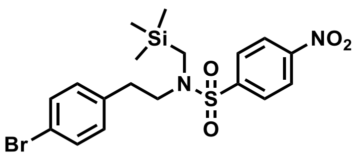

4g

The compound was synthesized according to GP1 starting from 1.57 mmol of amine, yielding 574 mg (1.22 mmol, 78%) of the product as an off-white solid.

^{1}H NMR (400 MHz, CD_2Cl_2) δ = 8.37–8.29 (m, 2H), 7.98–7.90 (m, 2H), 7.44–7.36 (m, 2H), 7.06–6.98 (m, 2H), 3.40–3.32 (m, 2H), 2.84–2.74 (m, 2H), 2.71 (s, 2H), 0.14 (s, 9H).

^{13}C NMR (101 MHz, CD_2Cl_2) δ = 150.3, 145.1, 137.5, 132.1, 130.9, 128.9, 124.7, 120.9, 52.0, 39.5, 34.3, -1.6.

HR-MS (ESI+) $[M+Na]^+$: 493.0227, calc. for $C_{18}H_{23}N_2O_4SSi^{79}BrNa$ 493.0223.

7.8 Characterisation of *N*-(3-fluoro-phenethyl)-4-nitro-*N*-((trimethylsilyl)-methyl)benzenesulfonamide (4h)

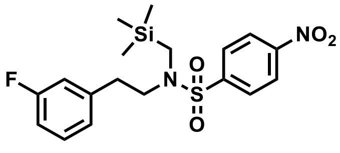

4h

The compound was synthesized according to GP1 starting from 3.21 mmol of amine, yielding 1.09 g (2.66 mmol, 83%) of the product as an off-white solid.

^{1}H NMR (400 MHz, CD_2Cl_2) δ = 8.36–8.30 (m, 2H), 7.99–7.92 (m, 2H), 7.30–7.22 (m, 1H), 6.96–6.88 (m, 2H), 6.87–6.80 (m, 1H), 3.41–3.34 (m, 2H), 2.84–2.77 (m, 2H), 2.71 (s, 2H), 0.15 (s, 9H).

^{13}C NMR (101 MHz, CD_2Cl_2) δ 163.3 (d, J = 245.4 Hz), 150.3, 145.0, 141.0 (d, J = 7.3 Hz), 130.6 (d, J = 8.4 Hz), 128.9, 124.9 (d, J = 2.8 Hz), 124.7, 115.9 (d, J = 21.1 Hz), 113.9 (d, J = 21.0 Hz), 52.0, 39.5, 34.6, -1.6.

^{19}F NMR (376 MHz, CD_2Cl_2) δ = -113.71– -113.82 (m).

HR-MS (ESI+) $[M+Na]^+$: 433.1026, calc. for $C_{18}H_{23}N_2O_4FSSiNa$ 433.1024.

7.9 Characterisation of *N*-(3-chloro-phenethyl)-4-nitro-*N*-((trimethylsilyl)-methyl)benzenesulfonamide (4i)

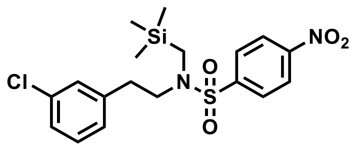

4i

The compound was synthesized according to GP1 starting from 1.39 mmol of amine, yielding 348 mg (0.82 mmol, 59%) of the product as an off-white solid.

^1H NMR (400 MHz, CD_2Cl_2) δ = 8.36–8.28 (m, 2H), 7.98–7.90 (m, 2H), 7.27–7.16 (m, 2H), 7.12–7.08 (m, 1H), 7.07–7.02 (m, 1H), 3.42–3.34 (m, 2H), 2.83–2.75 (m, 2H), 2.71 (s, 2H), 0.15 (s, 9H).

^{13}C NMR (101 MHz, CD_2Cl_2) δ = 150.3, 145.0, 140.6, 134.6, 130.4, 129.2, 128.8, 127.4, 127.2, 124.7, 52.0, 39.5, 34.6, -1.6.

HR-MS (ESI+) $[M+Na]^+$: 449.0729, calc. for $C_{18}H_{23}N_2O_4SSi^{35}ClNa$ 449.0729.

7.10 Characterisation of *N*-(3-bromo-phenethyl)-4-nitro-*N*-((trimethylsilyl)-methyl)benzenesulfonamide (4j)

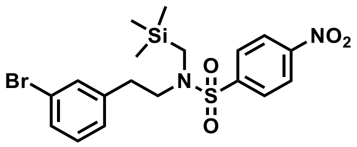

4j

The compound was synthesized according to GP1 starting from 3.26 mmol of amine, yielding 1.26 g (2.67 mmol, 82%) of the product as an off-white solid.

^1H NMR (400 MHz, CD_2Cl_2) δ = 8.36–8.29 (m, 2H), 7.97–7.90 (m, 2H), 7.38–7.32 (m, 1H), 7.28–7.23 (m, 1H), 7.20–7.13 (m, 1H), 7.09 (m, 1H), 3.42–3.35 (m, 2H), 2.81–2.74 (m, 2H), 2.71 (s, 2H), 0.15 (s, 9H).

^{13}C NMR (101 MHz, DMSO-d_6) δ = 150.2, 145.0, 140.8, 132.0, 130.6, 130.1, 128.8, 127.8, 124.6, 122.8, 51.9, 39.4, 34.4, -1.5.

HR-MS (ESI+) $[M+Na]^+$: 493.0226, calc. for $C_{18}H_{23}N_2O_4SSi^{79}BrNa$ 493.0223.

7.11 Characterisation of *N*-(3-iodo-phenethyl)-4-nitro-*N*-((trimethylsilyl)-methyl)benzenesulfonamide (4k)

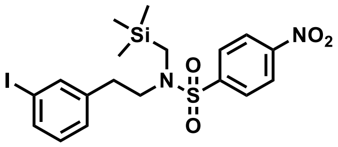

4k

The compound was synthesized according to GP1 starting from 0.55 mmol of amine, yielding 223 mg (0.43 mmol, 78%) of the product as an off-white solid.

^{1}H NMR (400 MHz, CD_2Cl_2) δ = 8.36–8.29 (m, 2H), 7.96–7.89 (m, 2H), 7.56 (ddd, J = 7.7, 1.5, 1.4 Hz, 1H), 7.46 (dd, J = 1.5, 1.5 Hz, 1H), 7.13 (ddd, J = 7.7, 1.5, 1.4 Hz, 1H), 7.03 (dd, J = 7.7, 7.7 Hz, 1H), 3.41–3.34 (m, 2H), 2.78–2.72 (m, 2H), 2.70 (s, 2H), 0.14 (s, 9H).

^{13}C NMR (101 MHz, CD_2Cl_2) δ = 150.3, 145.0, 141.0, 138.1, 136.2, 130.8, 128.8, 128.5, 124.7, 94.8, 52.0, 39.4, 34.4, -1.6.

HR-MS (ESI+) $[M+Na]^+$: 541.0089, calc. for $C_{18}H_{23}N_2O_4SSiINa$ 541.0085.

7.12 Characterisation of *N*-(3-methoxy-phenethyl)-4-nitro-*N*-((trimethylsilyl)-methyl)benzenesulfonamide (4l)

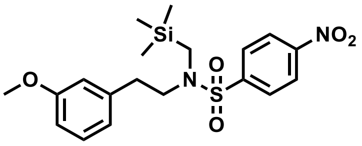

4l

The compound was synthesized according to GP1 starting from 3.26 mmol of amine, yielding 1.11 g (2.63 mmol, 81%) of the product as an off-white solid.

^{1}H NMR (400 MHz, CD_2Cl_2) δ = 8.34–8.28 (m, 2H), 7.97–7.90 (m, 2H), 7.21–7.15 (m, 1H), 6.77–6.68 (m, 2H), 6.65–6.60 (m, 1H),3.75 (s, 3H), 3.43–3.36 (m, 2H), 2.78–2.71 (m, 4H), 0.15 (s, 9H).

^{13}C NMR (101 MHz, CD_2Cl_2) δ = 160.2, 150.2, 145.1, 139.9, 130.0, 128.8, 124.6, 121.2, 114.8, 112.1, 55.4, 52.1, 39.1, 34.7, -1.5.

HR-MS (ESI+) $[M+Na]^+$: 445.1222, calc. for $C_{19}H_{26}N_2O_5SSiNa$ 445.1224.

7.13 Characterisation of *N*-(2-methoxy-phenethyl)-4-nitro-*N*-((trimethylsilyl)-methyl)benzenesulfonamide (4m)

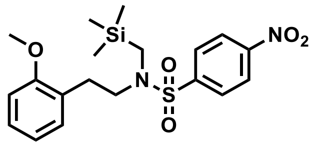

4m

The compound was synthesized according to GP1 starting from 1.37 mmol of amine, yielding 406 mg (0.70 mmol, 70%) of the product as an off-white solid.

^{1}H NMR (400 MHz, CD_2Cl_2) δ = 8.35–8.29 (m, 2H), 8.02–7.95 (m, 2H), 7.19 (td, *J* = 8.1, 1.5 Hz, 1H), 7.03 (dd, *J* = 7.4, 1.8 Hz, 1H), 6.87–6.80 (m, 2H), 3.81 (s, 3H), 3.42–3.35 (m, 2H), 2.77–2.69 (m, 4H), 0.16 (s, 9H).

^{13}C NMR (101 MHz, CD_2Cl_2) δ 157.9, 150.2, 145.6, 130.8, 128.9, 128.4, 126.6, 124.5, 120.9, 110.7, 55.5, 50.3, 38.8, 29.4, -1.7.

HR-MS (ESI+) $[M+Na]^+$: 445.1227, calc. for $C_{19}H_{26}N_2O_5SSiNa$ 445.1224.

7.14 Characterisation of *N*-(2-chloro-phenethyl)-4-nitro-*N*-((trimethylsilyl)-methyl)benzenesulfonamide (4n)

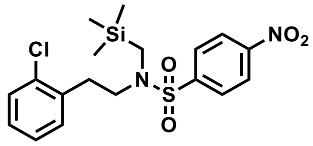

4n

The compound was synthesized according to GP1 starting from 1.37 mmol of amine, yielding 516 mg (1.21 mmol, 88%) of the product as an off-white solid.

^{1}H NMR (400 MHz, CD_2Cl_2) δ = 8.36–8.29 (m, 2H), 8.02–7.94 (m, 2H), 7.37–7.28 (m, 1H), 7.22–7.16 (m, 3H), 3.44–3.35 (m, 2H), 2.96–2.88 (m, 2H), 2.76 (s, 2H), 0.16 (s, 9H).

^{13}C NMR (101 MHz, CD_2Cl_2) δ = 150.3, 145.2, 136.1, 134.3, 131.4, 130.0, 128.9, 128.8, 127.6, 124.6, 50.5, 39.5, 32.6, -1.5.

HR-MS (ESI+) $[M+Na]^+$: 449.0729, calc. for $C_{18}H_{23}N_2O_4SSi^{35}ClNa$ 449.0729.

7.15 Characterisation of *N*-((trimethyl-silyl)methyl)-*N*-(3,4-dimethoxyphen-ethyl)-4-nitro-benzenesulfonamide (4o)

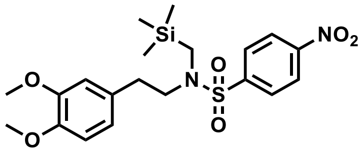

4o

The compound was synthesized according to GP1 starting from 3.33 mmol of amine, yielding 1.20 g (2.65 mmol, 80%) of the product as an off-white solid.

^{1}H NMR (500 MHz, CD_2Cl_2) δ = 8.33–8.27 (m, 2H), 7.96–7.89 (m, 2H), 6.75 (d, J = 8.1 Hz, 1H), 6.64 (dd, J = 8.1, 1.9 Hz, 1H), 6.60 (d, J = 1.9 Hz, 1H), 3.78 (s, 6H), 3.44–3.35 (m, 2H), 2.77–2.68 (m, 4H), 0.15 (s, 9H).

^{13}C NMR (126 MHz, CD_2Cl_2) δ = 150.1, 149.5, 148.4, 145.2, 130.8, 128.7, 124.5, 120.9, 112.4, 111.9, 56.1, 56.0, 52.3, 39.1, 34.2, -1.5.

HR-MS (ESI+) $[M+Na]^+$: 475.1330, calc. for $C_{20}H_{28}N_2O_6SSiNa$ 475.1330.

7.16 Characterisation of *N*-(3,5-dichloro-phenethyl)-4-nitro-*N*-((trimethyl-silyl)methyl)benzenesulfonamide (4p)

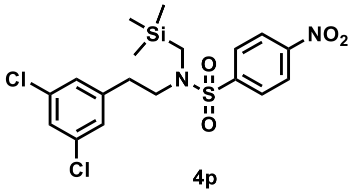

4p

The compound was synthesized according to GP1 starting from 2.39 mmol of amine, yielding 922 mg (2.00 mmol, 84%) of the product as an off-white solid.

^{1}H NMR (400 MHz, DMSO-d_6) δ = 8.39–8.32 (m, 2H), 8.05–7.97 (m, 2H), 7.38 (t, J = 1.9 Hz, 1H), 7.26 (d, J = 1.9 Hz, 2H), 3.39 (t, J = 7.4 Hz, 2H), 2.77 (t, J = 7.4 Hz, 2H), 2.72 (s, 2H), 0.09 (s, 9H).

^{13}C NMR (101 MHz, DMSO-d_6) δ = 149.6, 143.7, 142.7, 133.9, 128.5, 127.7, 126.1, 124.5, 50.8, 38.3, 32.5, -1.7.

HR-MS (ESI+) $[M+Na]^+$: 483.0341, calc. for $C_{18}H_{22}N_2O_4SSi^{35}Cl_2Na$ 483.0339.

7.17 Characterisation of *N*-((2-naphthyl)-ethyl)-4-nitro-*N*-((trimethylsilyl)-methyl)benzenesulfonamide (4q)

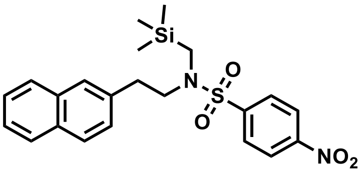

4q

The compound was synthesized according to GP1 starting from 1.28 mmol of amine, yielding 476 mg (1.08 mmol, 84%) of the product as an off-white solid.

^{1}H NMR (400 MHz, CD_2Cl_2) δ = 8.18–8.10 (m, 2H), 7.87–7.81 (m, 2H), 7.82–7.67 (m, 3H), 7.53–7.48 (m, 1H), 7.49–7.39 (m, 2H), 7.27 (dd, J = 8.4, 1.8 Hz, 1H), 3.59–3.51 (m, 2H), 2.99–2.91 (m, 2H), 2.81 (s, 2H), 0.19 (s, 9H).

^{13}C NMR (101 MHz, CD_2Cl_2) δ = 149.9, 145.4, 136.0, 133.8, 132.7, 128.7, 128.6, 127.9, 127.7, 127.5, 127.4, 126.6, 126.2, 124.4, 51.9, 38.9, 34.6, -1.5.

HR-MS (ESI+) $[M+Na]^+$: 465.1273, calc. for $C_{22}H_{26}N_2O_4SSiNa$ 465.1275.

7.18 Characterisation of *N*-(2-(benzofuran-3-yl)ethyl)-4-nitro-*N*-((trimethyl-silyl)methyl)benzenesulfonamide (4r)

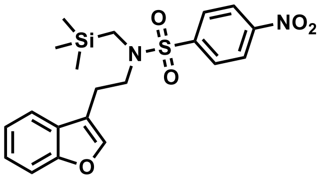

4r

The compound was synthesized according to GP1 starting from 2.39 mmol of amine, yielding 799 mg (1.85 mmol, 77%) of the product as an off-white solid.

^{1}H NMR (400 MHz, CD_2Cl_2) δ = 8.29–8.21 (m, 2H), 7.94–7.86 (m, 2H), 7.58–7.51 (m, 1H), 7.46–7.37 (m, 2H), 7.36–7.22 (m, 2H), 3.54–3.45 (m, 2H), 2.97–2.88 (m, 2H), 2.78 (s, 2H), 0.16 (s, 9H).

^{13}C NMR (101 MHz, CD_2Cl_2) δ = 155.6, 150.2, 145.0, 142.2, 128.7, 127.9, 124.9, 124.5, 123.0, 119.7, 117.0, 111.9, 50.2, 39.4, 23.1, -1.6.

HR-MS (ESI+) $[M+Na]^+$: 455.1069, calc. for $C_{20}H_{24}N_2O_5SSiNa$ 455.1067.

7.19 Characterisation of 4-nitro-*N*-(2-(thien-3-yl)ethyl)-*N*-((trimethylsilyl)-methyl)benzenesulfonamide (4s)

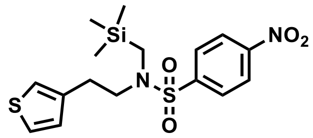

4s

The compound was synthesized according to GP1 starting from 1.37 mmol of amine, yielding 433 mg (1.09 mmol, 80%) of the product as an off-white solid.

^{1}H NMR (400 MHz, CD_2Cl_2) δ = 8.38–8.30 (m, 2H), 8.00–7.92 (m, 2H), 7.27 (dd, J = 4.9, 2.9 Hz, 1H), 6.98–6.97 (m, 1H), 6.91 (dd, J = 4.9, 1.3 Hz, 1H), 3.43–3.35 (m, 2H), 2.88–2.79 (m, 2H), 2.69 (s, 2H), 0.14 (s, 9H).

^{13}C NMR (101 MHz, CD_2Cl_2) δ = 150.3, 145.0, 138.6, 128.9, 128.3, 126.3, 124.7, 121.9, 51.5, 39.5, 29.3, -1.6.

HR-MS (ESI+) $[M+Na]^+$: 421.0679, calc. for $C_{16}H_{22}N_2O_4S_2SiNa$ 421.0683.

7.20 Characterisation of *N*-((trimethylsilyl)methyl)-*N*-(4-methyl-phenethyl)-4-nitro-benzenesulfonamide (4aa)

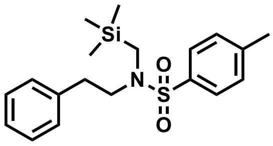

4aa

11.0 mmol of phenethylamine (1.34 g) and 11.0 mmol of triethylamine (1.12 g, 1 eq.) were dissolved in 20 mL of THF and the resulting mixture placed in an ice bath and stirred. After 5 minutes 11.0 mmol of tosyl chloride (2.10 g, 1 eq.) were added and the mixture left to stir overnight. Next, 20 mL of 1 M NaOH(aq.) were added and the solution transferred into a separatory funnel and extracted with CH_2Cl_2 (3x20 mL). The combined organic fractions were washed thoroughly with 30 mL of 1 M HCl(aq.), dried over $MgSO_4$, filtered and evaporated to dryness under reduced pressure and the crude product used without further purification.

6.04 mmol of NaH (242 mg, 1.2 eq.) was added to a flame-dried flask under Ar atmosphere and suspended in dry DMF (20 mL). The amide (1.39 g, 5.03 mmol) was then added at rt and the mixture stirred for 1 h with an argon stream. After this 6.54 mmol of the iodide (1.4 g, 1.3 eq) was added and the mixture stirred overnight. Then, the reaction mixture was partitioned between ethyl acetate (20 mL) and brine (20 mL) and the aqueous phase extracted 2 more times with ethyl acetate (20 mL). The combined organic extracts were dried over $MgSO_4$ and evaporated to dryness. The crude product was purified by column chromatography yielding 1.07 g (2.96 mmol, 59%) of the pure product as an off-white solid.

^{1}H NMR (400 MHz, DMSO-d$_6$) δ = 7.73–7.67 (m, 2H), 7.46–7.39 (m, 2H), 7.32–7.25 (m, 2H), 7.23–7.18 (m, 1H), 7.18–7.13 (m, 2H), 3.24–3.17 (m, 2H), 2.73–2.66 (m, 2H), 2.59 (s, 2H), 2.39 (s, 3H), 0.08 (s, 9H).

^{13}C NMR (101 MHz, DMSO-d$_6$) δ = 143.1, 138.3, 134.9, 129.8, 128.6, 128.5, 127.3, 126.4, 51.7, 38.5, 33.7, 21.0, -1.7.

HR-MS (ESI+) [M+H]$^+$: 362.1606, calc. for $C_{20}H_{27}NO_2SSi$ 362.1605.

7.21 Characterisation of 4-cyano-*N*-phenethyl-*N*-((trimethylsilyl)methyl)-benzenesulfonamide (4ab)

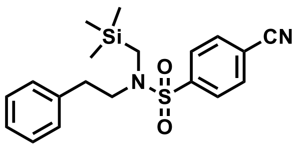

4ab

6.6 mmol of phenethylamine (800 mg) and 6.6 mmol of triethylamine (668 mg, 1 eq.) were dissolved in 20 mL of THF and the resulting mixture placed in an ice bath and stirred. After 5 minutes 6.6 mmol of the sulfonyl chloride (1.33 g, 1 eq.) were added and the mixture left to stir overnight. Next, 20 mL of 1 M NaOH(aq.) were added and the solution transferred into a separatory funnel and extracted with CH_2Cl_2 (3x20 mL). The combined organic fractions were washed thoroughly with 30 mL of 1 M HCl(aq.), dried over $MgSO_4$, filtered and evaporated to dryness under reduced pressure and the crude product used without further purification.
6.6 mmol of NaH (263 mg, 1.1 eq.) was added to a flame-dried flask under Ar atmosphere and suspended in dry DMF (20 mL). The amide (1.72 g, 6 mmol) was then added at rt and the mixture stirred for 1 h with an argon stream. After this 7.8 mmol of the iodide (1.67 g, 1.3 eq) was added and the mixture stirred overnight. Then, the reaction mixture was partitioned between ethyl acetate (20 mL) and brine (20 mL) and the aqueous phase extracted 2 more times with ethyl acetate (20 mL). The combined organic extracts were dried over $MgSO_4$ and evaporated to dryness. The crude product was purified by column chromatography yielding 1.56 g (4.19 mmol, 70%) of the pure product as a colorless liquid.

^{1}H NMR (400 MHz, DMSO-d$_6$) δ = 8.12–8.05 (m, 2H), 8.03–7.95 (m, 2H), 7.32–7.24 (m, 2H), 7.23–7.12 (m, 3H), 3.33–3.25 (m, 2H), 2.75–2.66 (m, 4H), 0.09 (s, 9H).

^{13}C NMR (101 MHz, DMSO-d$_6$) δ = 142.1, 138.0, 133.5, 128.6, 128.5, 128.0, 126.5, 117.7, 115.2, 51.5, 38.4, 33.7, -1.7.

HR-MS (ESI+) [M+Na]$^+$: 395.1220, calc. for $C_{19}H_{24}N_2O_2SSiNa$ 395.1220.

7.22 Characterisation of *N*-phenethyl-*N*-((trimethylsilyl)methyl)methanesulfonamide (4ac)

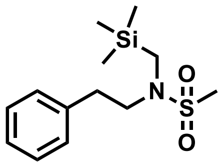

4ac

6.6 mmol of phenethylamine (800 mg) and 6.6 mmol of triethylamine (668 mg, 1 eq.) were dissolved in 20 mL of THF and the resulting mixture placed in an ice bath and stirred. After 5 minutes 6.6 mmol of mesyl chloride (756 mg, 1 eq.) were added and the mixture left to stir overnight. Next, 20 mL of 1 M NaOH(aq.) were added and the solution transferred into a separatory funnel and extracted with CH_2Cl_2 (3x20 mL). The combined organic fractions were washed thoroughly with 30 mL of 1 M HCl(aq.), dried over $MgSO_4$, filtered and evaporated to dryness under reduced pressure and the crude product used without further purification.
3.31 mmol of NaH (133 mg, 1.1 eq.) was added to a flame-dried flask under Ar atmosphere and suspended in dry DMF (10 mL). The amide (0.6 g, 3.01 mmol) was then added at rt and the mixture stirred for 1 h with an argon stream. After this 3.91 mmol of the iodide (838 mg, 1.3 eq) was added and the mixture stirred overnight. Then, the reaction mixture was partitioned between ethyl acetate (20 mL) and brine (20 mL) and the aqueous phase extracted 2 more times with ethyl acetate (20 mL). The combined organic extracts were dried over $MgSO_4$ and evaporated to dryness. The crude product was purified by column chromatography yielding 147 mg (0.52 mmol, 17%) of the pure product as a colorless liquid.

^{1}H NMR (400 MHz, DMSO-d$_6$) δ = 7.35–7.17 (m, 5H), 3.32–3.25 (m, 2H), 2.90–2.83 (m, 2H), 2.79 (s, 3H), 2.70 (s, 2H), 0.10 (s, 9H).

^{13}C NMR (101 MHz, DMSO-d$_6$) δ = 138.6, 128.7, 128.5, 126.4, 51.5, 38.2, 35.1, 34.0, -1.6.

HR-MS (ESI+) $[M+Na]^+$: 308.1109, calc. for $C_{13}H_{23}NO_2SSiNa$ 308.1111.

7.23 Characterisation of *N*-phenethyl-*N*-((trimethylsilyl)methyl)trifluoromethanesulfonamide (4ad)

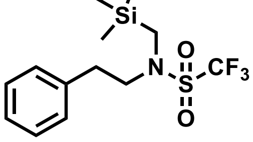

4ad

6.6 mmol of amine (800 mg) and 6.6 mmol of triethylamine (668 mg, 1 eq.) were dissolved in 20 mL of $CH_2Cl_2$and the resulting mixture placed in a dry ice/acetone bath (-78 °C) and stirred. After 5 minutes 6.6 mmol of Tf_2O (1.86 g, 1 eq.) were slowly added with a syringe and the mixture left to stir overnight. Next, 25 mL of sat. bicarbonate solution was added and the solution transferred into a separatory funnel and extracted with CH_2Cl_2 (3x20 mL). The combined organic fractions were washed thoroughly

with 30 mL of 1 M HCl(aq.), dried over $MgSO_4$, filtered and evaporated to dryness under reduced pressure, yielding the crude product which was used without further purification.
1.58 mmol of the amide (400 mg) and 1.58 mmol of K_2CO_3 (158 mg, 1 eq.) were added to round-bottom flask suspended in acetone (15 mL). After this 2.05 mmol of the iodide (440 mg, 1.3 eq) was added and the mixture stirred a rt overnight. Then, the reaction mixture was partitioned between ethyl acetate (20 mL) and brine (20 mL) and the aqueous phase extracted 2 more times with ethyl acetate (20 mL). The combined organic extracts were dried over $MgSO_4$ and evaporated to dryness. The crude product was purified by column chromatography yielding 214 mg (0.63 mmol, 40%) of the pure product as a colorless liquid.

^{1}H NMR (400 MHz, CD_2Cl_2) δ = 7.36–7.29 (m, 2H), 7.28–7.15 (m, 3H), 3.61–3.52 (m, 2H), 3.18–2.80 (m, 4H), 0.18 (s, 9H).

^{13}C NMR (101 MHz, CD_2Cl_2) δ = 137.7, 129.1, 127.3, 120.7 (q, J = 324.7 Hz), 52.5, 39.9, 34.9, -1.6.

^{19}F NMR (376 MHz, CD_2Cl_2) δ = -75.16.

HR-MS (ESI+) $[M+Na]^+$: 362.0829, calc. for $C_{13}H_{20}F_3NO_2SSiNa$ 362.0828.

7.24 Characterisation of *N*-phenethyl-*N*-((trimethylsilyl)methyl)trifluoroacetamide (4ae)

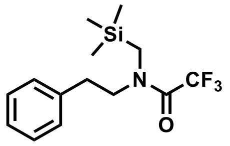

4ae

11.0 mmol of phenethylamine (1.34 mg) and 11.0 mmol of triethylamine (1.11 mg, 1 eq.) were dissolved in 20 mL of $CH_2Cl_2$and the resulting mixture placed in an ice bath and stirred. After 5 minutes 11.0 mmol of trifluoroacetic anhydride (2.32 g, 1 eq.) were added and the mixture left to stir overnight. Next, 20 mL of 1 M NaOH(aq.) were added and the solution transferred into a separatory funnel and extracted with CH_2Cl_2 (3x20 mL). The combined organic fractions were washed thoroughly with 30 mL of 1 M HCl(aq.), dried over $MgSO_4$, filtered and evaporated to dryness under reduced pressure and the crude product used without further purification.
5.28 mmol of NaH (212 mg, 1.05 eq.) was added to a flame-dried flask under Ar atmosphere and suspended in dry DMF (20 mL). The amide (1.09 g, 5.03 mmol) was then added at rt and the mixture stirred for 1 h with an argon stream. After this 6.54 mmol of the iodide (1.40 g, 1.3 eq) was added and the mixture stirred overnight. Then, the reaction mixture was partitioned between ethyl acetate (20 mL) and brine (20 mL) and the aqueous phase extracted 2 more times with ethyl acetate (20 mL). The combined organic extracts were dried over $MgSO_4$ and evaporated to dryness. The crude product was purified by column chromatography yielding 955 mg (3.15 mmol, 63%) of the pure product as a colorless liquid.

^{1}H NMR (400 MHz, DMSO-d_6) δ = 7.36–7.28 (m, 2H), 7.27–7.19 (m, 3H), 3.57–3.48 (m, 2H), 3.04 (s, rotamers, 1.54 H), 2.96–2.83 (m, rotamers, 2.46 H), 0.12–0.03 (m, 9H).

^{13}C NMR (101 MHz, DMSO-d_6) δ = 154.5 (q, J = 34.6 Hz), 138.1, 137.6, 128.7, 128.7, 128.6, 128.5, 126.7, 126.5, 116.6 (q, J = 287.9 Hz), 50.6 (q, J = 2.9 Hz), 49.9, 38.8, 38.4 (q, J = 2.7 Hz), 34.0, 31.5, -1.4, -1.9.

^{19}F NMR (376 MHz, DMSO-d_6) δ = -67.41, -67.70.

HR-MS (GC-EI) $[M]^+$: 303.1259, calc. for $C_{14}H_{20}NOF_3Si$ 303.1261.

7.25 Characterisation of *N*-phenethyl-*N*-((trimethylsilyl)methyl)benzamide (4af)

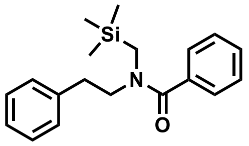

4af

24.7 mmol of phenethylamine (3.0 g) and 24.7 mmol of triethylamine (2.51 g, 1 eq.) were dissolved in 40 mL of $CH_2Cl_2$and the resulting mixture placed in an ice bath and stirred. After 5 minutes 24.7 mmol of benzoyl chloride (3.48 g, 1 eq.) were added and the mixture left to stir overnight. Next, 40 mL of 1 M NaOH(aq.) were added and the solution transferred into a separatory funnel and extracted with CH_2Cl_2 (3x40 mL). The combined organic fractions were washed thoroughly with 30 mL of 1 M HCl(aq.), dried over $MgSO_4$, filtered and evaporated to dryness under reduced pressure and the crude product used without further purification.
5.56 mmol of NaH (227 mg, 1.05 eq.) was added to a flame-dried flask under Ar atmosphere and suspended in DMF (25 mL). The amide (1.21 g, 5.38 mmol) was then added at rt and the mixture stirred for 1 h with an argon stream. After this 7.0 mmol of the iodide (1.5 mg, 1.3 eq) was added and the mixture stirred overnight. Then, the reaction mixture was partitioned between ethyl acetate (20 mL) and brine (20 mL) and the aqueous phase extracted 2 more times with ethyl acetate (20 mL). The combined organic extracts were dried over $MgSO_4$ and evaporated to dryness. The crude product was purified by column chromatography yielding 1.17 g (3.77 mmol, 70%) of the pure product as an off-white solid.

^1H NMR (400 MHz, DMSO-d_6) δ = 7.46–6.90 (m, 10H), 3.64 (s, br, rotamers 0.48H), 3.39–3.27 (m, rotamers, 1.52H), 3.02 (s, rotamers, 1.65H), 2.92 (s, br, rotamers, 0.35H), 2.78 (t, J = 7.4 Hz, 2H), 0.23– -0.16 (m, rotamers, 9H).

^{13}C NMR (101 MHz, DMSO-d_6) δ = 169.8, 138.3, 137.1, 128.8, 128.7, 128.4, 128.2, 126.3, 126.1, 52.4, 36.3, 33.8, -0.9.

HR-MS (ESI+) $[M+Na]^+$: 334.1596, calc. for $C_{19}H_{25}NOSiNa$ 334.1598.

7.26 Characterisation of benzyl *N*-phenethyl-*N*-((trimethylsilyl)methyl)-carbamate (4ag)

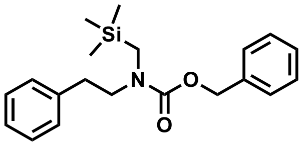

4ag

11.0 mmol of phenethylamine (1.34 g) and 11.0 mmol of triethylamine (1.12 g, 1 eq.) were dissolved in 20 mL of THF and the resulting mixture placed in an ice bath and stirred. After 5 minutes 11.0 mmol of the cbz chloride (1.88 g, 1 eq.) were added and the mixture left to stir overnight. Next, 20 mL of 1 M NaOH(aq.) were added and the solution transferred into a separatory funnel and extracted with CH_2Cl_2 (3x20 mL). The combined organic fractions were washed thoroughly with 30 mL of 1 M HCl(aq.), dried over $MgSO_4$, filtered and evaporated to dryness under reduced pressure and the crude product used without further purification.
2.94 mmol of NaH (120 mg, 1.2 eq.) was added to a flame-dried flask under Ar atmosphere and suspended in dry THF (10 mL). The amide (625 mg, 2.45 mmol) was then added at rt and the mixture stirred for 1 h with an argon stream. After this 3.18 mmol of the iodide (680 mg, 1.3 eq) was added and the mixture stirred overnight. Then, the reaction mixture was partitioned between ethyl acetate (20 mL) and brine (20 mL) and the aqueous phase extracted 2 more times with ethyl acetate (20 mL). The combined organic extracts were dried over $MgSO_4$ and evaporated to dryness. The crude product was purified by column chromatography yielding 331 mg (0.97 mmol, 40%) of the pure product as a colorless liquid.

^{1}H NMR (400 MHz, DMSO-d_6) δ = 7.40–7.09 (m, 10H), 5.03–4.99 (m, rotamers, 2H), 3.47–3.35 (m, 2H), 2.84–2.68 (m, 4H), 0.02– -0.04 (m, rotamers, 9H).

^{13}C NMR (101 MHz, DMSO-d_6, rotamers observed) δ = 155.1, 138.9, 137.2, 137.0, 128.7, 128.4, 128.3, 127.8, 127.7, 127.4, 126.2, 66.0, 50.7, 50.4, 38.4, 37.7, 33.8, 33.1, -1.3, -1.7.

HR-MS (ESI+) $[M+Na]^+$: 364.1705, calc. for $C_{20}H_{27}NO_2SiNa$ 364.1703.

7.27 Characterisation of methyl *N*-phenethyl-*N*-((trimethylsilyl)methyl)-carbamate (4ah)

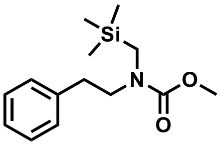

4ah

33 mmol of phenethylamine (4.0 g) and 33 mmol of triethylamine (3.34 g, 1 eq.) were dissolved in 50 mL of THF and the resulting mixture placed in an ice bath and stirred. After 5 minutes 36 mmol of methyl chloroformate (3.43 g, 1.1 eq.) were added and the mixture left to stir overnight. Next, 40 mL of 1 M NaOH(aq.) were added and the solution transferred into a separatory funnel and extracted with CH_2Cl_2 (3x50 mL). The combined organic fractions were washed thoroughly with 30 mL of 1 M HCl(aq.), dried over $MgSO_4$, filtered and evaporated to dryness under reduced pressure and the crude product used without further purification.
15.6 mmol of NaH (625 mg, 1.4 eq.) was added to a flame-dried flask under Ar atmosphere and suspended in dry THF (35 mL). The amide (2.0 g, 11.2 mmol) was then added at rt and the mixture stirred for 1 h with an argon stream. After this 14.5 mmol of the iodide (3.1 g, 1.3 eq) was added and the mixture stirred overnight. Then, the reaction mixture was partitioned between ethyl acetate (20 mL) and brine (20 mL) and the aqueous phase extracted 2 more times with ethyl acetate (20 mL). The combined organic extracts were dried over $MgSO_4$ and evaporated to dryness. The crude product was purified by column chromatography yielding 1.49 g (5.6 mmol, 50%) of the pure product as a colorless liquid.

^{1}H NMR (400 MHz, DMSO-d$_6$) δ = 7.30–7.27 (m, 2H), 7.21–7.18 (m, 3H), 3.54–3.48 (m, rotamers, 3H), 3.38–3.35 (m, 2H), 2.79–2.75 (m, 2H), 2.70 (s, 2H), 0.02 (s, 9H).

^{13}C NMR (101 MHz, DMSO-d$_6$, rotamers observed) δ = 162.6, 155.8, 154.1, 138.9, 138.2, 128.7, 128.4, 128.3, 126.4, 126.2, 53.9, 52.2, 51.9, 50.8, 50.2, 41.4, 38.3, 37.8, 33.7, 33.2, -1.3, -1.7.

HR-MS (ESI+) [M+Na]$^+$: 288.1389, calc. for $C_{14}H_{23}NO_2SiNa$ 288.1390.

7.28 Characterisation of 4-nitro-*N*-(2-phenylpropyl)-*N*-((trimethylsilyl)-methyl)benzenesulfonamide (4ba)

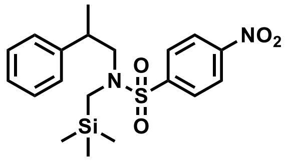

4ba

The compound was synthesized according to GP1 starting from 3.15 mmol of amine, yielding 1.01 g (2.48 mmol, 79%) of the product as an off-white solid.

^{1}H NMR (400 MHz, CD_2Cl_2) δ = 8.30–8.22 (m, 2H), 7.85–7.77 (m, 2H), 7.30–7.17 (m, 3H), 7.17–7.12 (m, 2H), 3.44 (dd, J = 14.1, 7.2 Hz, 1H), 3.27 (dd, J = 14.1, 7.2 Hz, 1H), 3.03 (ddq, J = 7.2, 7.2, 7.2 Hz, 1H), 2.64 (d, J = 15.9 Hz, 1H), 2.58 (d, J = 15.9 Hz, 1H), 1.25 (d, J = 7.2 Hz, 3H), 0.09 (s, 9H).

^{13}C NMR (101 MHz, CD_2Cl_2) δ = 150.1, 145.3, 144.1, 129.0, 128.8, 127.7, 127.2, 124.5, 57.8, 39.6, 38.6, 19.6, -1.3.

HR-MS (ESI+) [M+Na]$^+$: 429.1274; calc. for $C_{19}H_{26}N_2O_4SSiNa$ 429.1275.

7.29 Characterisation of 4-nitro-*N*-(1-phenylbutan-2-yl)-*N*-((trimethylsilyl)-methyl)benzenesulfonamide (4bb)

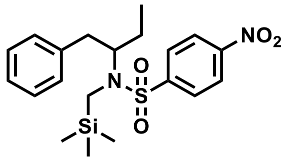

4bb

The compound was synthesized according to GP1 starting from 2.45 mmol of amine, but the reaction mixture was heated after the addition of $TMSCH_2I$ to 60 °C overnight, yielding 694 mg (1.65 mmol, 67%) of the product as an off-white solid.

^{1}H NMR (400 MHz, CD_2Cl_2) δ = 8.29–8.22 (m, 2H), 7.90–7.84 (m, 2H), 7.24–7.13 (m, 3H), 7.09–7.01 (m, 2H), 3.90–3.81 (m, 1H), 2.63–2.48 (m, 4H), 1.50–1.30 (m, 2H), 0.72 (t, J = 7.3 Hz, 3H), 0.22 (s, 9H).

^{13}C NMR (101 MHz, CD_2Cl_2) δ = 150.0, 146.1, 138.7, 129.5, 128.9, 128.6, 126.9, 124.6, 63.6, 39.6, 34.6, 25.2, 11.6, -0.7.

HR-MS (ESI) $[M+Na]^+$: 443.1435; calc. for $C_{20}H_{28}N_2O_4SSiNa$ 443.1431.

7.30 Characterisation of *N*-(1,2-diphenylethyl)-4-nitro-*N*-((trimethylsilyl)-methyl)benzenesulfonamide (4bc)

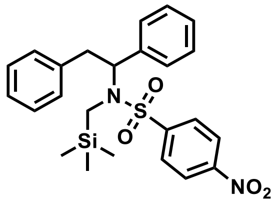

4bc

The compound was synthesized according to GP1 starting from 2.64 mmol of amine, but after the addition of $TMSCH_2I$ the mixture was heated to 40 °C overnight, yielding 674 mg (1.44 mmol, 56%) of the product as an off-white solid.

^1H NMR (400 MHz, CD_2Cl_2) δ = 8.18–8.11 (m, 2H), 7.72–7.66 (m, 2H), 7.41–7.33 (m, 2H), 7.33–7.24 (m, 3H), 7.16–7.08 (m, 3H), 7.07–7.01 (m, 2H), 5.37 (dd, *J* = 8.7, 6.9 Hz, 1H), 3.23 (dd, *J* = 14.3, 8.7 Hz, 1H), 2.94 (dd, *J* = 14.3, 6.9 Hz, 1H), 2.66 (d, *J* = 16.1 Hz, 1H), 2.62 (d, *J* = 16.1 Hz, 1H), -0.09 (s, 9H).

^{13}C NMR (101 MHz, CD_2Cl_2) δ = 149.8, 145.9, 138.6, 137.9, 129.4, 128.9, 128.9, 128.9, 128.6, 128.4, 127.0, 124.4, 63.9, 37.0, 35.3, -1.3.

HR-MS (ESI) $[M+Na]^+$: 491.1431; calc. for $C_{24}H_{28}N_2O_4SSiNa$ 491.1431.

7.31 Characterisation of *N*-((1-(4-chlorophenyl)cyclopropyl)methyl)-4-nitro-*N*-((trimethylsilyl)methyl)benzenesulfonamide (4bd)

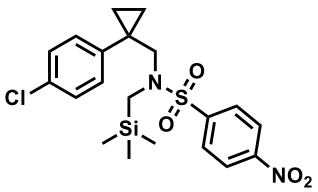

4bd

The compound was synthesized according to GP1 starting from 2.81 mmol of amine yielding 754 mg (1.66 mmol, 60%) of the product as an off-white solid.

^1H NMR (400 MHz, CD_2Cl_2) δ = 8.25–8.19 (m, 2H), 7.72–7.66 (m, 2H), 7.11–7.07 (m, 4H), 3.43 (s, 2H), 2.64 (s, 2H), 0.88–0.79 (m, 4H), 0.07 (s, 9H).

^{13}C NMR (101 MHz, CD_2Cl_2) δ = 149.9, 145.7, 141.4, 132.9, 131.3, 128.7, 128.6, 124.4, 59.3, 39.3, 24.3, 12.2, -0.9.

HR-MS (ESI) $[M+Na]^+$: 475.0886; calc. for $C_{20}H_{25}N_2O_4SSi^{35}ClNa$ 475.0885.

7.32 Characterisation of *N*-((4-nitrophenyl)sulfonyl)-*N*-((trimethylsilyl)-methyl)-phenylalanine methyl ester (4be)

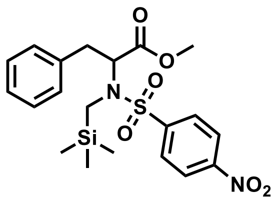

4be

The compound was synthesized according to GP1 starting from 1.09 mmol of amine, but after the addition of $TMSCH_2I$ the mixture was heated to 50 °C overnight, yielding 391 mg (0.87 mmol, 80%) of the product as an off-white solid.

^{1}H NMR (400 MHz, CD_2Cl_2) δ = 8.29–8.23 (m, 2H), 7.89–7.82 (m, 2H), 7.28–7.19 (m, 3H), 7.19–7.14 (m, 2H), 4.73 (dd, *J* = 8.0, 7.1 Hz, 1H), 3.46 (s, 3H), 3.24 (dd, *J* = 14.1, 8.0 Hz, 1H), 2.87 (dd, *J* = 14.1, 7.0 Hz, 1H), 2.73 (d, *J* = 16.1 Hz, 1H), 2.62 (d, *J* = 16.1 Hz, 1H), 0.13 (s, 9H).

^{13}C NMR (101 MHz, CD_2Cl_2) δ = 170.3, 150.3, 144.9, 136.8, 129.6, 129.0, 128.9, 127.4, 124.4, 62.9, 52.3, 37.0, 36.7, -1.1.

HR-MS (ESI) $[M+Na]^+$: 473.1177; calc. for $C_{20}H_{26}N_2O_6SSiNa$ 473.1173.

7.33 Characterisation of *N*-((1*R*,2*R*,5*S*)-5-methyl-2-(2-phenylpropan-2-yl)cyclohexyl)-4-nitro-*N*-((trimethylsilyl)methyl)benzenesulfonamide (4bf)

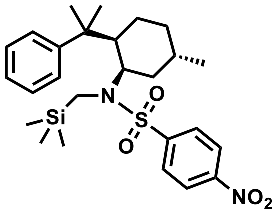

4bf

The compound was synthesized according to GP1 starting from 1.92 mmol of amine, but after the addition of $TMSCH_2I$ the mixture was heated to 50 °C overnight, yielding 244 mg (0.49 mmol, 26%) of the product as an off-white solid.

^{1}H NMR (400 MHz, CD_2Cl_2) δ = 8.46–8.30 (m, 2H), 8.10–7.93 (m, 2H), 7.43–7.18 (m, 4H), 7.20–7.11 (m, 1H), 3.89 (s, 1H), 2.59 (d, *J* = 15.5 Hz, 1H), 2.36 (d, *J* = 15.5 Hz, 1H), 1.91 – 1.77 (m, 1H), 1.57–0.92 (m, 12H), 0.61–0.43 (m, 4H), 0.27 (s, 9H).

^{13}C NMR (101 MHz, CD_2Cl_2) δ = 152.0, 150.3, 146.3, 128.9, 128.4, 125.8, 125.7, 124.7, 61.9, 48.3, 41.8, 39.3, 35.9, 34.7, 32.8, 31.9, 29.1, 21.9, 19.7, 0.6.

HR-MS (ESI) $[M+Na]^+$: 525.2217; calc. for $C_{26}H_{38}N_2O_4SSiNa$ 525.2214.

7.34 Characterisation of 4-nitro-*N*-(2-phenylcyclohexyl)-*N*-((trimethylsilyl)-methyl)benzenesulfonamide (4bg)

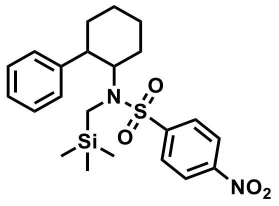

4bg

The compound was synthesized according to GP1 starting from 1.83 mmol of amine, but after the addition of $TMSCH_2I$ the mixture was heated to 50 °C overnight, yielding 284 mg (0.64 mmol, 35%) of the product as an off-white solid.

^{1}H NMR (400 MHz, CD_2Cl_2) *δ* = 8.23–8.07 (m, 2H), 7.71–7.52 (m, 2H), 7.33–6.92 (m, 5H), 3.99 (s, br, 1H), 2.65 (s, br, 1H), 2.55–2.34 (m, 2H), 1.91–1.68 (m, 3H), 1.65–1.35 (m, 4H), 1.32–1.17 (m, 1H), 0.02 (s, 9H).

^{13}C NMR (101 MHz, CD_2Cl_2) *δ* = 149.8, 146.5, 143.7, 128.7, 128.5, 128.1, 126.8, 124.4, 62.7, 48.4, 36.6, 31.7, 27.3, 26.3, 26.1, -0.7.

HR-MS (ESI) $[M+Na]^+$: 469.1589; calc. for $C_{22}H_{30}N_2O_4SSiNa$ 469.1588.

7.35 Characterisation of 2-((4-nitrophenyl)sulfonyl)-1,2,3,4-tetrahydro-isoquinoline (5a)

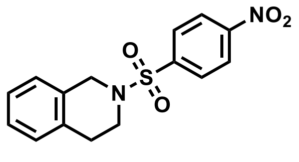

5a

The compound was synthesized according to GP2 yielding 21.5 mg (0.069 mmol, 69%) as an off-white solid.

^{1}H NMR (400 MHz, acetone-d_6) *δ* = 8.49–8.42 (m, 2H), 8.20–8.12 (m, 2H), 7.20–7.07 (m, 4H), 4.36 (s, 2H), 3.48 (t, *J* = 6.0 Hz, 2H), 2.93 (t, *J* = 6.0 Hz, 2H).

^{13}C NMR (101 MHz, acetone-d_6) *δ* = 143.5, 134.0, 132.5, 129.9, 129.7, 127.7, 127.3, 127.2, 125.3, 48.3, 44.7, 29.2.

HR-MS (ESI+) $[M+Na]^+$: 341.0564, calc. for $C_{15}H_{14}N_2O_4SNa$ 341.0567.

7.36 Characterisation of 7-(*tert*-butyl)-2-((4-nitrophenyl)sulfonyl)-1,2,3,4-tetrahydroisoquinoline (5b)

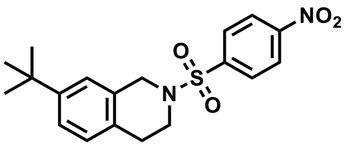

5b

The compound was synthesized according to GP2 yielding 22.6 mg (0.060 mmol, 60%) as an off-white solid.

^{1}H NMR (400 MHz, DMSO-d$_6$) δ = 8.44–8.37 (m, 2H), 8.12–8.06 (m, 2H), 7.19–7.13 (m, 2H), 7.02 (d, *J* = 8.2 Hz, 1H), 4.27 (s, 2H), 3.38 (t, *J* = 6.0 Hz, 2H), 2.81 (t, *J* = 6.0 Hz, 2H), 1.22 (s, 9H).

^{13}C NMR (101 MHz, DMSO-d$_6$) δ = 149.9, 148.6, 141.7, 130.8, 129.9, 129.0, 128.3, 124.6, 123.8, 123.0, 47.4, 43.7, 34.1, 31.1, 27.4.

HR-MS (ESI+) [M+Na]$^+$: 397.1194, calc. for $C_{19}H_{22}N_2O_4SNa$ 397.1193.

7.37 Characterisation of 7-methyl-2-((4-nitrophenyl)sulfonyl)-1,2,3,4-tetrahydroisoquinoline (5c)

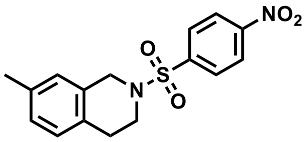

5c

The compound was synthesized according to GP2 yielding 18.1 mg (0.054 mmol, 54%) as an off-white solid.

^{1}H NMR (400 MHz, DMSO-d$_6$) δ = 8.45–8.38 (m, 2H), 8.12–8.05 (m, 2H), 7.02–6.93 (m, 3H), 4.24 (s, 2H), 3.37 (t, *J* = 6.0 Hz, 2H), 2.79 (t, *J* = 6.0 Hz, 2H), 2.22 (s, 3H).

^{13}C NMR (101 MHz, DMSO-d$_6$) δ = 150.0, 141.9, 135.2, 131.1, 129.8, 128.9, 128.6, 127.5, 126.7, 124.7, 47.0, 43.6, 27.5, 20.6.

HR-MS (ESI+) [M+Na]$^+$: 355.0721, calc. for $C_{16}H_{16}N_2O_4SNa$ 355.0723.

7.38 Characterisation of 7-methoxy-2-((4-nitrophenyl)sulfonyl)-1,2,3,4-tetrahydroisoquinoline (5d)

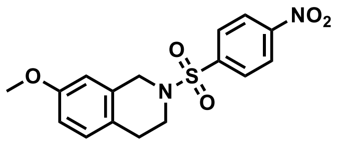

5d

The compound was synthesized according to GP2 yielding 5.2 mg (0.015 mmol, 15%) as an off-white solid.

^{1}H NMR (400 MHz, DMSO-d$_6$) δ = 8.45–8.38 (m, 2H), 8.12–8.04 (m, 2H), 7.01 (d, J = 8.1 Hz, 1H), 6.82–6.69 (m, 2H), 4.26 (s, 2H), 3.69 (s, 3H), 3.37 (t, J = 6.0 Hz, 2H), 2.76 (t, J = 6.0 Hz, 2H).

^{13}C NMR (101 MHz, DMSO-d$_6$) δ = 172.1, 157.6, 150.0, 141.9, 132.4, 129.7, 128.9, 124.7, 113.3, 110.9, 55.1, 47.2, 43.8, 27.1.

HR-MS (ESI+) [M+Na]$^+$: 371.0671, calc. for $C_{16}H_{16}N_2O_5SNa$ 371.0672.

7.39 Characterisation of 7-fluoro-2-((4-nitrophenyl)sulfonyl)-1,2,3,4-tetrahydroisoquinoline (5e)

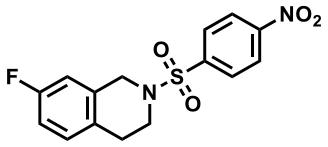

5e

The compound was synthesized according to GP2 yielding 8.0 mg (0.025 mmol, 25%) as an off-white solid.

^{1}H NMR (400 MHz, DMSO-d$_6$) δ = 8.45–8.38 (m, 2H), 8.12–8.05 (m, 2H), 7.14 (dd, J = 8.5, 5.9 Hz, 1H), 7.05 (dd, J = 9.7, 2.7 Hz, 1H), 6.99 (ddd, J = 8.7, 8.7, 2.7 Hz, 1H), 4.32 (s, 2H), 3.41 (t, J = 6.0 Hz, 2H), 2.81 (t, J = 6.0 Hz, 2H).

^{13}C NMR (101 MHz, DMSO-d$_6$) δ = 160.4 (d, J = 242.1 Hz), 150.0, 141.9, 133.6 (d, J = 7.9 Hz), 130.6 (d, J = 8.1 Hz), 128.9 (d, J = 2.8 Hz), 128.9, 124.7, 113.9 (d, J = 21.3 Hz), 112.7 (d, J = 22.1 Hz), 46.9, 43.4, 27.2.

^{19}F NMR (376 MHz, DMSO-d$_6$) δ = -116.42– -116.52 (m).

HR-MS (ESI+) [M+Na]$^+$: 359.0472, calc. for $C_{15}H_{13}N_2O_4FSNa$ 359.0472.

7.40 Characterisation of 7-chloro-2-((4-nitrophenyl)sulfonyl)-1,2,3,4-tetra-hydroisoquinoline (5f)

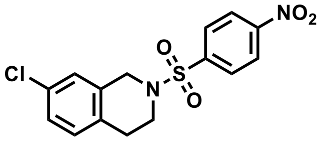

5f

The compound was synthesized according to GP2 yielding 8.0 mg (0.023 mmol, 23%) as an off-white solid.

^{1}H NMR (400 MHz, $CDCl_3$) δ = 8.41–8.33 (m, 2H), 8.06–7.97 (m, 2H), 7.14 (dd, J = 8.3, 2.1 Hz, 1H), 7.08–6.99 (m, 2H), 4.32 (s, 2H), 3.46 (t, J = 6.0 Hz, 2H), 2.88 (t, J = 6.0 Hz, 2H).

^{13}C NMR (101 MHz, $CDCl_3$) δ = 150.4, 143.0, 132.8, 132.5, 131.3, 130.4, 128.9, 127.5, 126.3, 124.6, 47.2, 43.7, 28.3.

HR-MS (ESI+) $[M+Na]^+$: 395.1036, calc. for $C_{15}H_{13}N_2O_4S^{35}ClNa$ 395.1036.

7.41 Characterisation of 7-bromo-2-((4-nitrophenyl)sulfonyl)-1,2,3,4-tetra-hydroisoquinoline (5g)

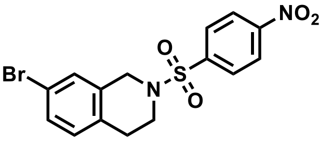

5g

The compound was synthesized according to GP2 yielding 9.8 mg (0.025 mmol, 25%) as an off-white solid.

^{1}H NMR (400 MHz, DMSO-d_6) δ = 8.44–8.38 (m, 2H), 8.11–8.05 (m, 2H), 7.40 (d, J = 2.1 Hz, 1H), 7.32 (dd, J = 8.2, 2.1 Hz, 1H), 7.07 (d, J = 8.2 Hz, 1H), 4.32 (s, 2H), 3.40 (t, J = 6.0 Hz, 2H), 2.80 (t, J = 6.0 Hz, 2H).

^{13}C NMR (101 MHz, DMSO-d_6) δ = 150.0, 141.9, 134.1, 132.4, 130.9, 129.6, 129.0, 128.9, 124.7, 118.9, 46.5, 43.2, 27.4.

HR-MS (ESI+) $[M+Na]^+$: 418.9673, calc. for $C_{15}H_{13}N_2O_4S^{79}BrNa$ 418.9672.

7.42 Characterisation of 6-fluoro-2-((4-nitrophenyl)sulfonyl)-1,2,3,4-tetra-hydroisoquinoline (5h)

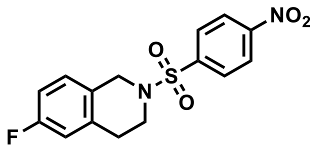

5h

The compound was synthesized according to GP2 yielding 21.4 mg (0.064 mmol, 64%) as an off-white solid.

^{1}H NMR (400 MHz, DMSO-d$_6$) δ = 8.45–8.38 (m, 2H), 8.13–8.05 (m, 2H), 7.21 (dd, J = 8.5, 5.9 Hz, 1H), 7.04–6.94 (m, 2H), 4.27 (s, 2H), 3.39 (t, J = 6.0 Hz, 2H), 2.86 (t, J = 6.0 Hz, 2H).

^{13}C NMR (101 MHz, DMSO-d$_6$) δ = 160.8 (d, J = 242.7 Hz), 150.0, 141.8, 135.5 (d, J = 8.0 Hz), 128.9, 128.4 (d, J = 8.5 Hz), 127.6 (d, J = 2.8 Hz), 124.7, 114.9 (d, J = 21.2 Hz), 113.3 (d, J = 21.7 Hz), 46.6, 43.1, 27.9.

^{19}F NMR (376 MHz, DMSO-d$_6$) δ = -115.92– -116.04 (m).

HR-MS (ESI+) [M+H]$^+$: 337.0650, calc. for $C_{15}H_{14}N_2O_4FS$ 337.0653.

7.43 Characterisation of 6-chloro-2-((4-nitrophenyl)sulfonyl)-1,2,3,4-tetra-hydroisoquinoline (5i)

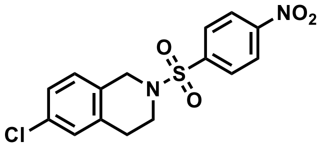

5i

The compound was synthesized according to GP2 yielding 19.8 mg (0.054 mmol, 54%) as an off-white solid.

^{1}H NMR (400 MHz, DMSO-d$_6$) δ = 8.43–8.38 (m, 2H), 8.12–8.06 (m, 2H), 7.25–7.17 (m, 3H), 4.28 (s, 2H), 3.39 (t, J = 6.0 Hz, 2H), 2.85 (t, J = 6.0 Hz, 2H).

^{13}C NMR (101 MHz, DMSO-d$_6$) δ = 150.0, 141.8, 135.4, 131.1, 130.5, 128.9, 128.3, 126.2, 124.7, 46.6, 43.1, 27.7.

HR-MS (ESI+) [M+H]$^+$: 353.0355, calc. for $C_{15}H_{14}N_2O_4S^{35}Cl$ 353.0357.

7.44 Characterisation of 6-bromo-2-((4-nitrophenyl)sulfonyl)-1,2,3,4-tetra-hydroisoquinoline (5j)

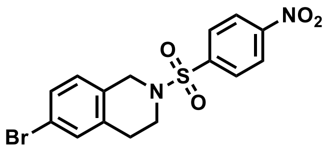

5j

The compound was synthesized according to GP2 yielding 20.8 mg (0.052 mmol, 52%) as an off-white solid.

^{1}H NMR (400 MHz, DMSO-d$_6$) δ = 8.46–8.37 (m, 2H), 8.14–8.05 (m, 2H), 7.38–7.31 (m, 2H), 7.16–7.10 (m, 1H), 4.27 (s, 2H), 3.39 (t, J = 6.0 Hz, 2H), 2.86 (t, J = 6.0 Hz, 2H).

^{13}C NMR (101 MHz, DMSO-d$_6$) δ = 150.0, 141.8, 135.8, 131.2, 130.9, 128.9, 128.6, 124.7, 119.6, 46.7, 43.0, 27.6.

HR-MS (ESI+) [M+Na]$^+$: 418.9670, calc. for $C_{15}H_{13}N_2O_4S^{79}BrNa$ 418.9672.

7.45 Characterisation of 6-iodo-2-((4-nitrophenyl)sulfonyl)-1,2,3,4-tetra-hydroisoquinoline (5k)

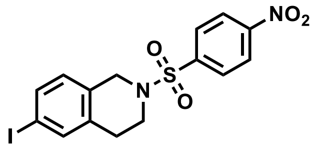

5k

The compound was synthesized according to GP2 yielding 19.3 mg (0.043 mmol, 43%) as an off-white solid.

^{1}H NMR (400 MHz, DMSO-d$_6$) δ = 8.45–8.37 (m, 2H), 8.12–8.06 (m, 2H), 7.53–7.48 (m, 2H), 7.01–6.94 (m, 1H), 4.25 (s, 2H), 3.38 (t, J = 6.0 Hz, 2H), 2.83 (t, J = 6.0 Hz, 2H).

^{13}C NMR (101 MHz, DMSO-d$_6$) δ = 150.0, 141.8, 137.1, 135.8, 134.8, 131.3, 128.9, 128.6, 124.7, 92.4, 46.7, 43.1, 27.4.

HR-MS (ESI+) [M+Na]$^+$: 466.9533, calc. for $C_{15}H_{13}N_2O_4SINa$ 466.9533.

7.46 Characterisation of 6-methoxy-2-((4-nitrophenyl)sulfonyl)-1,2,3,4-tetra-hydroisoquinoline (5l)

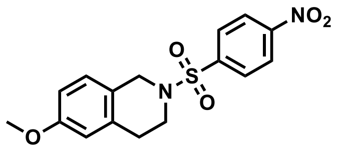

5l

The compound was synthesized according to GP2 yielding 8.1 mg (0.023 mmol, 23%) as an off-white solid.

^{1}H NMR (400 MHz, DMSO-d$_6$) δ = 8.45–8.37 (m, 2H), 8.12–8.04 (m, 2H), 7.06 (d, J = 8.5 Hz, 1H), 6.74 (dd, J = 8.5, 2.6 Hz, 1H), 6.68 (d, J = 2.6 Hz, 1H), 4.21 (s, 2H), 3.68 (s, 3H), 3.36 (t, J = 6.0 Hz, 2H), 2.83 (t, J = 6.0 Hz, 2H)).

^{13}C NMR (101 MHz, DMSO-d$_6$) δ = 157.9, 149.9, 141.8, 134.2, 128.9, 127.4, 124.7, 123.3, 113.2, 112.7, 55.0, 46.7, 43.4, 28.2.

HR-MS (ESI+) [M+Na]$^+$: 371.0671, calc. for $C_{16}H_{16}N_2O_5SNa$ 371.0672.

7.47 Characterisation of 5-methoxy-2-((4-nitrophenyl)sulfonyl)-1,2,3,4-tetra-hydroisoquinoline (5m)

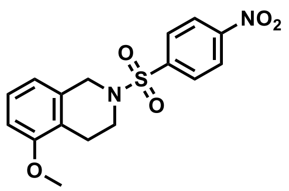

5m

The compound was synthesized according to GP2 yielding 5.5 mg (0.016 mmol, 16%) as an off-white solid.

^{1}H NMR (400 MHz, CDCl$_3$) δ = 8.38–8.30 (m, 2H), 8.05–7.97 (m, 2H), 7.14 (dd, J = 7.9, 7.9 Hz, 1H), 6.68 (d, J = 7.9 Hz, 1H), 6.65 (d, J = 7.9 Hz, 1H), 4.33 (s, 2H), 3.78 (s, 3H), 3.45 (t, J = 6.2 Hz, 2H), 2.79 (t, J = 6.1 Hz, 2H).

^{13}C NMR (101 MHz, CDCl$_3$) δ = 157.2, 150.3, 143.3, 132.3, 128.9, 127.3, 124.4, 121.9, 118.3, 108.3, 55.5, 47.5, 43.7, 22.9.

HR-MS (ESI+) [M+Na]$^+$: 371.0672, calc. for $C_{16}H_{16}N_2O_5SNa$ 371.0672.

7.48 Characterisation of 5-chloro-2-((4-nitrophenyl)sulfonyl)-1,2,3,4-tetra-hydroisoquinoline (5n)

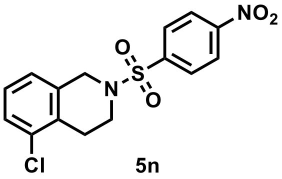

5n

The compound was synthesized according to GP2 yielding 5.0 mg (0.014 mmol, 14%) as an off-white solid.

^{1}H NMR (400 MHz, $CDCl_3$) δ = 8.41–8.33 (m, 2H), 8.06–7.99 (m, 2H), 7.26–7.23 (m, 1H), 7.13 (dd, J = 7.8, 7.8 Hz, 1H), 6.97 (d, J = 7.8 Hz, 1H), 4.34 (s, 2H), 3.49 (t, J = 6.1 Hz, 2H), 2.94 (t, J = 6.1 Hz, 2H).

^{13}C NMR (101 MHz, $CDCl_3$) δ = 142.9, 134.7, 133.3, 131.2, 128.9, 128.1, 127.6, 124.9, 124.6, 47.6, 43.7, 26.8.

HR-MS (ESI+) $[M+Na]^+$: 375.0176, calc. for $C_{15}H_{13}N_2O_4S^{35}ClNa$ 375.0177.

7.49 Characterisation of 6,7-dimethoxy-2-((4-nitrophenyl)sulfonyl)-1,2,3,4-tetrahydroisoquinoline (5o)

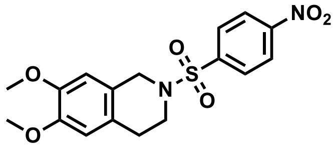

5o

The compound was synthesized according to GP2 yielding 11.9 mg (0.031 mmol, 31%) as an off-white solid.

^{1}H NMR (400 MHz, $CDCl_3$) δ = 8.39–8.32 (m, 2H), 8.05–7.97 (m, 2H), 6.53 (s, 1H), 6.50 (s, 1H), 4.27 (s, 2H), 3.84–3.79 (m, 6H), 3.43 (t, J = 5.9 Hz, 2H), 2.82 (t, J = 5.9 Hz, 2H).

^{13}C NMR (101 MHz, $CDCl_3$) δ = 150.3, 148.2, 148.0, 143.1, 128.8, 124.8, 124.5, 122.7, 111.5, 108.9, 56.1, 56.0, 47.2, 43.9, 28.3.

HR-MS (ESI+) $[M+Na]^+$: 401.0777, calc. for $C_{17}H_{18}N_2O_6SNa$ 401.0778.

7.50 Characterisation of 6,8-dichloro-2-((4-nitrophenyl)sulfonyl)-1,2,3,4-tetrahydroisoquinoline (5p)

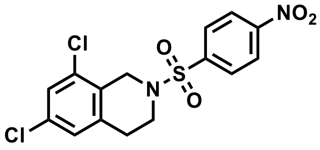

5p

The compound was synthesized according to GP2 yielding 18.0 mg (0.046 mmol, 46%) as an off-white solid.

^{1}H NMR (400 MHz, CD_2Cl_2) δ = 8.42–8.34 (m, 2H), 8.08–8.00 (m, 2H), 7.27 (d, J = 2.1 Hz, 1H), 7.07 (d, J = 2.1 Hz, 1H), 4.29 (s, 2H), 3.42 (t, J = 5.9 Hz, 2H), 2.93 (t, J = 5.9 Hz, 2H).

^{13}C NMR (101 MHz, DMSO-d_6) δ = 150.1, 141.8, 137.8, 131.8, 128.9, 128.3, 127.8, 126.4, 124.9, 44.9, 42.4, 27.9.

HR-MS (ESI+) $[M+Na]^+$: 408.9785, calc. for $C_{15}H_{12}N_2O_4S^{35}Cl_2Na$ 408.9787.

7.51 Characterisation of 2-((4-nitrophenyl)sulfonyl)-1,2,3,4-tetrahydrobenzo-[h]isoquinoline (5q)

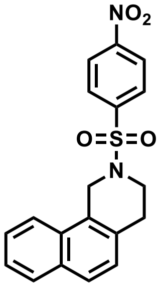

5q

The compound was synthesized according to GP2 yielding 7.9 mg (0.021 mmol, 21%) as an off-white solid.

^{1}H NMR (400 MHz, CD_2Cl_2) δ = 8.41–8.34 (m, 2H), 8.13–8.05 (m, 2H), 7.83 (d, J = 8.4 Hz, 1H), 7.78 (d, J = 8.4 Hz, 1H), 7.69 (d, J = 8.4 Hz, 1H), 7.60–7.53 (m, 1H), 7.52–7.46 (m, 1H), 7.20 (d, J = 8.4 Hz, 1H), 4.74 (s, 2H), 3.55 (t, J = 5.8 Hz, 2H), 3.07 (t, J = 5.8 Hz, 2H).

^{13}C NMR (101 MHz, DMSO-d_6) δ = 150.1, 141.7, 131.6, 130.9, 129.5, 129.2, 128.5, 127.3, 126.9, 126.7, 125.8, 125.6, 124.7, 121.9, 45.1, 42.9, 28.8.

HR-MS (EI) $[M]^+$: 368.0826, calc. for $C_{19}H_{16}N_2O_4S$ 368.0825.

7.52 Characterisation of 2-((4-nitrophenyl)sulfonyl)-1,2,3,4-tetrahydrobenzofuro[2,3-c]pyridine (5r)

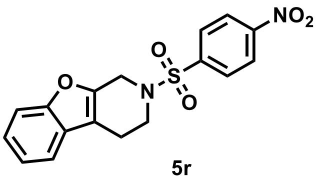

5r

The compound was synthesized according to GP2 yielding 3.3 mg (0.009 mmol, 9%) as an off-white solid.

^{1}H NMR (400 MHz, $CDCl_3$) δ = 8.38–8.31 (m, 2H), 8.07–8.00 (m, 2H), 7.45–7.35 (m, 2H), 7.30–7.18 (m, 2H), 4.46 (t, J = 2.0 Hz, 2H), 3.62 (t, J = 5.6 Hz, 2H), 2.78 (tt, J = 5.6, 2.0 Hz, 2H).

^{13}C NMR (101 MHz, $CDCl_3$) δ = 154.9, 147.3, 143.7, 128.7, 127.1, 124.6, 124.5, 123.1, 118.9, 111.5, 43.9, 43.8, 29.9, 21.1.

HR-MS (ESI+) $[M+Na]^+$: 381.0514, calc. for $C_{17}H_{14}N_2O_5SNa$ 381.0516.

7.53 Characterisation of 6-((4-nitrophenyl)sulfonyl)-4,5,6,7-tetrahydrothieno[2,3-c]pyridine (5s)

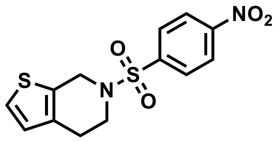

5s

The compound was synthesized according to GP2 yielding 5.4 mg (0.017 mmol, 17%) as an yellow solid.

^{1}H NMR (400 MHz, $CDCl_3$) δ = 8.39–8.30 (m, 2H), 8.06–7.96 (m, 2H), 7.13 (d, J = 5.1 Hz, 1H), 6.70 (d, J = 5.1 Hz, 1H), 4.48 (s, 2H), 3.53 (t, J = 5.9 Hz, 2H), 2.74 (t, J = 5.9 Hz, 2H).

^{13}C NMR (101 MHz, $CDCl_3$) δ = 150.3, 143.8, 133.1, 129.4, 128.7, 126.9, 124.5, 123.9, 45.4, 43.8, 25.4.

HR-MS (ESI+) $[M+Na]^+$: 347.0128, calc. for $C_{13}H_{12}N_2O_4S_2Na$ 347.0131.

7.54 Characterisation of 2-tosyl-1,2,3,4-tetrahydroisoquinoline (5aa)

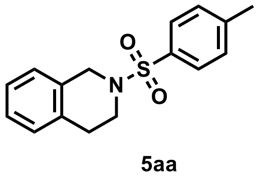

5aa

The compound was synthesized according to GP2 yielding 18.6 mg (0.065 mmol, 65%) as an off-white solid.

^{1}H NMR (400 MHz, DMSO-d$_6$) δ = 7.75–7.67 (m, 2H), 7.47–7.41 (m, 2H), 7.18–7.07 (m, 4H), 4.16 (s, 2H), 3.26 (t, J = 6.0 Hz, 2H), 2.84 (t, J = 6.0 Hz, 2H), 2.39 (s, 3H).

^{13}C NMR (101 MHz, DMSO-d$_6$) δ = 143.7, 133.0, 132.9, 131.6, 129.9, 128.7, 127.5, 126.7, 126.5, 126.1, 47.3, 43.6, 28.0, 21.0.

HR-MS (ESI+) [M+Na]$^+$: 310.0870, calc. for $C_{16}H_{17}NO_2SNa$ 310.0872.

7.55 Characterisation of 2-((4-cyanophenyl)sulfonyl)-1,2,3,4-tetrahydroisoquinoline (5ab)

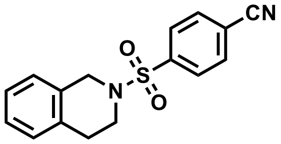

5ab

The compound was synthesized according to GP2 yielding 20.0 mg (0.067 mmol, 67%) as an off-white solid.

^{1}H NMR (400 MHz, CD_2Cl_2) δ = 7.96–7.90 (m, 2H), 7.87–7.80 (m, 2H), 7.20–7.00 (m, 4H), 4.30 (s, 2H), 3.41 (t, J = 5.9 Hz, 2H), 2.91 (t, J = 5.9 Hz, 2H).

^{13}C NMR (101 MHz, CD_2Cl_2) δ = 141.4, 133.4, 133.3, 131.6, 129.2, 128.5, 127.3, 126.8, 126.6, 117.7, 116.9, 47.8, 44.2, 28.9.

HR-MS (ESI+) [M+Na]$^+$: 321.0666, calc. for $C_{16}H_{14}N_2O_2SNa$ 321.0668.

7.56 Characterisation of 2-methylsulfonyl-1,2,3,4-tetrahydroisoquinoline (5ac)

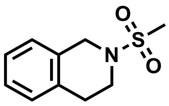

5ac

The compound was synthesized according to GP2 yielding 8.8 mg (0.038 mmol, 38%) as a colorless liquid.

^{1}H NMR (400 MHz, CD_2Cl_2) δ = 7.24–7.14 (m, 3H), 7.14–7.08 (m, 1H), 4.43 (s, 2H), 3.53 (t, J = 6.0 Hz, 2H), 2.98 (t, J = 6.0 Hz, 2H), 2.81 (s, 3H).

^{13}C NMR (101 MHz, CD_2Cl_2) δ = 133.8, 132.3, 129.4, 127.3, 126.8, 126.7, 47.7, 43.9, 35.8, 29.1.

HR-MS (ESI+) [M+Na]$^+$: 234.0558, calc. for $C_{10}H_{13}NO_2SNa$ 234.0559.

7.57 Characterisation of 2-trifluoromethylsulfonyl-1,2,3,4-tetrahydroisoquinoline (5ad)

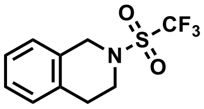

5ad

The literature-known compound was synthesized according to GP2 yielding 16.1 mg (0.061 mmol, 61%) as a colorless liquid.[3]

^{1}H NMR (400 MHz, $CDCl_3$) δ = 7.29–7.22 (m, 2H), 7.21–7.15 (m, 1H), 7.13–7.06 (m, 1H), 4.68 (s, 2H), 3.78 (s, br, 2H), 3.01 (t, J = 5.7 Hz, 2H).

^{13}C NMR (101 MHz, $CDCl_3$) δ = 132.7, 130.8, 129.3, 127.5, 127.0, 126.2, 120.3 (q, J = 323.4 Hz), 47.7, 44.6, 29.0.

^{19}F NMR (376 MHz, $CDCl_3$) δ = -75.51.

MS (APCI+) $[M+H]^+$: 266.0, calc. for $C_{10}H_{11}NO_2F_3S$ 266.1.

7.58 Characterisation of 2-trifluoroacetyl-1,2,3,4-tetrahydroisoquinoline (5ae)

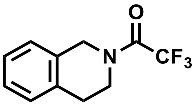

5ae

The literature-known compound was synthesized according to GP2 yielding 15.4 mg (0.067 mmol, 67%) as a colorless liquid.[4]

^{1}H NMR (400 MHz, $CDCl_3$) δ = 7.27–7.06 (m, 4H), 4.77 (s, rotamers, 1.28 H), 4.72 (s, rotamers, 0.71H), 3.90–3.78 (m, rotamers, 2H), 2.98–2.89 (m, 2H).

^{13}C NMR (101 MHz, $CDCl_3$, rotamers observed) δ = 134.2, 133.4, 131.6, 131.6, 129.0, 128.7, 127.7, 127.2, 127.1, 126.9, 126.7, 126.2, 118.1, 115.2, 47.1 (q, J = 4.0 Hz), 45.7, 43.5 (q, J = 3.5 Hz), 41.9, 29.4, 27.9.

^{19}F NMR (376 MHz, $CDCl_3$) δ = -69.36 (rotamers), -69.40 (rotamers).

MS (APCI+) $[M+H]^+$: 230.0, calc. for $C_{11}H_{11}NOF_3$ 230.1.

7.59 Characterisation of 2-benzoyl-1,2,3,4-tetrahydroisoquinoline (5af)

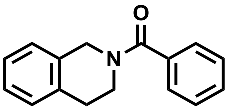

5af

The literature-known compound was synthesized according to GP2 yielding 14.0 mg (0.059 mmol, 59%) as an off-white solid.[5]

^{1}H NMR (400 MHz, DMSO-d_6) δ = 7.55–7.36 (m, 5H), 7.32–6.90 (m, 4H), 4.85–4.43 (m, rotamers, 2H), 3.96–3.46 (m, rotamers, 2H), 2.97–2.77 (m, 2H).

^{13}C NMR (101 MHz, DMSO-d_6) δ = 136.2, 134.2, 133.1, 129.7, 128.7, 128.5, 128.4, 128.3, 127.1, 126.8, 126.6, 126.4, 126.2, 49.1, 44.7, 44.12, 28.8, 27.7.

MS (APCI+) $[M+H]^+$: 238.1, calc. for $C_{16}H_{16}NO$ 238.1.

7.60 Characterisation of 2-benzoxycarbonyl-1,2,3,4-tetrahydro-isoquinoline (5ag)

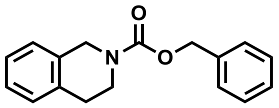

5ag

The literature-known compound was synthesized according to GP2 yielding 10.0 mg (0.037 mmol, 37%) as a colorless liquid.[6]

^{1}H NMR (400 MHz, CD_2Cl_2) δ = 7.44–7.24 (m, 5H), 7.24–7.05 (m, 4H), 5.16 (s, 2H), 4.64 (s, 2H), 3.71 (t, J = 6.0 Hz, 2H), 2.89–2.80 (m, 2H).

^{13}C NMR (101 MHz, CD_2Cl_2, rotamers observed) δ = 155.7, 137.6, 135.1, 129.3, 129.1, 128.9, 128.8, 128.3, 128.2, 126.8, 126.6, 67.3, 46.1, 42.1, 41.8, 29.3, 29.2.

MS (APCI+) $[M+H]^+$: 268.1, calc. for $C_{17}H_{18}NO_2$ 268.1.

7.61 Characterisation of 2-methoxycarbonyl-1,2,3,4-tetrahydroisoquinoline (5ah)

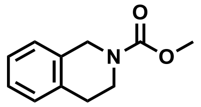

5ah

The literature-known compound was synthesized according to GP2 yielding 11.5 mg (0.060 mmol, 60%) as a colorless liquid.[7]

^{1}H NMR (400 MHz, DMSO-d_6) δ = 7.22–7.12 (m, 4H), 4.53 (s, 2H), 3.64 (s, 3H, rotamers), 3.59 (t, J = 6.0 Hz, 2H), 2.79 (t, J = 6.0 Hz, 2H).

^{13}C NMR (101 MHz, DMSO-d_6, rotamers observed) δ = 155.3, 134.4, 133.2, 128.6, 128.4, 128.3, 127.7, 127.1, 126.4, 126.2, 126.1, 53.6, 52.4, 45.2, 44.5, 41.1, 28.1, 27.4.

MS (APCI+) $[M+H]^+$: 192.0, calc. for $C_{11}H_{14}NO_2$ 192.1.

7.62 Characterisation of 4-methyl-2-((4-nitrophenyl)sulfonyl)-1,2,3,4-tetrahydroisoquinoline (5ba)

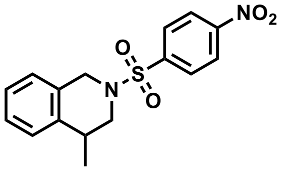

5ba

The compound was synthesized according to GP2 yielding 20.5 mg (0.062 mmol, 62%) as an off-white solid.

^{1}H NMR (400 MHz, $CDCl_3$) δ = 8.42–8.35 (m, 2H), 8.07–7.99 (m, 2H), 7.24–7.12 (m, 3H), 7.06–6.99 (m, 1H), 4.46 (d, *J* = 14.9 Hz, 1H), 4.17 (d, *J* = 14.9 Hz, 1H), 3.35–3.26 (m, 2H), 3.13–3.00 (m, 1H), 1.34 (d, *J* = 7.0 Hz, 3H).

^{13}C NMR (101 MHz, CDCl3) δ = 150.3, 142.9, 138.4, 130.4, 128.9, 128.1, 127.4, 126.7, 126.3, 124.5, 50.1, 47.8, 33.1, 20.5.

HR-MS (ESI+) $[M+Na]^+$: 355.0721, calc. for $C_{16}H_{16}N_2O_4SNa$ 355.0723.

7.63 Characterisation of 3-ethyl-2-((4-nitrophenyl)sulfonyl)-1,2,3,4-tetrahydroisoquinoline (5bb)

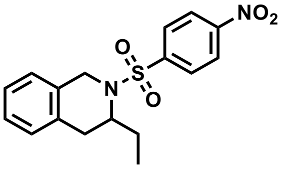

5bb

The compound was synthesized according to GP2 yielding 20.1 mg (0.058 mmol, 58%) as an off-white solid.

^{1}H NMR (400 MHz, $CDCl_3$) δ = 8.27–8.18 (m, 2H), 7.98–7.89 (m, 2H), 7.19–7.08 (m, 2H), 7.06–7.01 (m, 1H), 7.01–6.95 (m, 1H), 4.75 (d, *J* = 16.9 Hz, 1H), 4.33 (d, *J* = 16.9 Hz, 1H), 4.16 (ddt, *J* = 7.6, 6.1, 2.2 Hz, 1H), 2.78 (dd, *J* = 16.3, 6.1 Hz, 1H), 2.58 (dd, *J* = 16.3, 2.2 Hz, 1H), 1.55–1.39 (m, 2H), 0.93 (t, *J* = 7.4 Hz, 3H).

^{13}C NMR (101 MHz, $CDCl_3$) δ = 149.9, 146.5, 132.0, 131.1, 129.6, 128.2, 127.4, 126.6, 125.9, 124.3, 54.2, 43.5, 31.9, 25.3, 10.9.

HR-MS (ESI+) $[M+Na]^+$: 369.0880, calc. for $C_{17}H_{18}N_2O_4SNa$ 369.0880.

7.64 Characterisation of 3-phenyl-2-((4-nitrophenyl)sulfonyl)-1,2,3,4-tetrahydroisoquinoline (5bc)

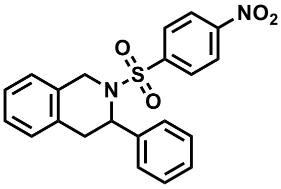

5bc

The compound was synthesized according to GP2 yielding 7.4 mg (0.019 mmol, 19%) as an off-white solid.

^{1}H NMR (400 MHz, DMSO-d$_6$) δ = 8.28–8.21 (m, 2H), 8.07–8.00 (m, 2H), 7.30–7.18 (m, 5H), 7.13–7.02 (m, 4H), 5.27 (t, J = 5.5 Hz, 1H), 4.68 (d, J = 16.2 Hz, 1H), 4.41 (d, J = 16.2 Hz, 1H), 3.09–2.97 (m, 2H).

^{13}C NMR (101 MHz, DMSO-d$_6$) δ = 149.6, 144.5, 141.1, 133.1, 132.5, 128.6, 128.3, 128.1, 127.2, 127.2, 126.5, 126.2, 125.9, 124.4, 55.8, 44.7, 33.6.

HR-MS (ESI+) [M+Na]$^+$: 417.0879, calc. for $C_{21}H_{18}N_2O_4SNa$ 417.0880.

7.65 Characterisation of 1'*H*-7'-chloro-2'-((4-nitrophenyl)sulfonyl)-2',3'-dihydro-spiro[cyclopropane-1,4'-isoquinoline] (5bd)

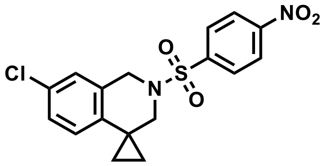

5bd

The compound was synthesized according to GP2 yielding 18.7 mg (0.049 mmol, 49%) as an off-white solid.

^{1}H NMR (400 MHz, CDCl$_3$) δ = 8.38–8.30 (m, 2H), 8.02–7.94 (m, 2H), 7.11 (dd, J = 8.4, 2.2 Hz, 1H), 7.03 (d, J = 2.2 Hz, 1H), 6.58 (d, J = 8.4 Hz, 1H), 4.46 (s, 2H), 3.29 (s, 2H), 1.01–0.94 (m, 4H).

^{13}C NMR (101 MHz, CDCl$_3$) δ = 143.6, 137.1, 133.0, 131.8, 128.9, 128.0, 125.9, 124.4, 123.5, 52.8, 48.4, 19.1, 17.1.

HR-MS (ESI+) [M+Na]$^+$: 401.0333, calc. for $C_{17}H_{15}N_2O_4S^{35}ClNa$ 401.0333.

7.66 Characterisation of 3-(methoxycarbonyl)-2-((4-nitrophenyl)sulfonyl)-1,2,3,4-tetrahydroisoquinoline (5be)

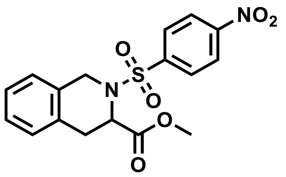

5be

The compound was synthesized according to GP2 yielding 25.1 mg (0.067 mmol, 67%) as an off-white solid.

^{1}H NMR (400 MHz, DMSO-d$_6$) δ = 8.40–8.33 (m, 2H), 8.16–8.08 (m, 2H), 7.17–7.11 (m, 4H), 5.06 (dd, *J* = 5.9, 3.7 Hz, 1H), 4.68 (d, *J* = 15.9 Hz, 1H), 4.39 (d, *J* = 15.9 Hz, 1H), 3.43 (s, 3H), 3.18 – 3.06 (m, 2H).

^{13}C NMR (101 MHz, DMSO-d$_6$) δ = 170.2, 149.9, 143.9, 131.1, 130.9, 128.7, 128.5, 126.9, 126.6, 126.2, 124.5, 54.0, 52.3, 44.2, 30.8.

HR-MS (ESI+) [M+Na]$^+$: 399.0621, calc. for $C_{17}H_{16}N_2O_6SNa$ 399.0621.

7.67 Characterisation of 1*H*-(3*R*,4a*R*,11a*S*)-5-((4-nitrophenyl)-sulfonyl)-3,11,11-trimethyl-2,3,4,4a,5,6,11,11a-octahydro-dibenzo[b,e]azepine (5bf)

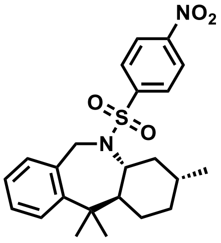

5bf

The compound was synthesized according to GP2 yielding 22.5 mg (0.052 mmol, 52%) as an off-white solid.

^{1}H NMR (400 MHz, CDCl$_3$) δ = 8.01–7.93 (m, 2H), 7.51–7.42 (m, 2H), 7.23 (dd, *J* = 8.1, 1.1 Hz, 1H), 7.18 (ddd, *J* = 8.1, 7.0, 1.5 Hz, 1H), 7.07 (ddd, *J* = 7.3, 7.0, 1.1 Hz, 1H), 7.02 (dd, *J* = 7.3, 1.5 Hz, 1H), 4.83 (d, *J* = 17.0 Hz, 1H), 4.54 (d, *J* = 17.0 Hz, 1H), 3.65 (td, *J* = 11.2, 3.6 Hz, 1H), 2.23–2.13 (m, 1H), 2.10–1.95 (m, 2H), 1.84–1.73 (m, 1H), 1.68–1.56 (m, 1H), 1.36–1.11 (m, 6H), 0.96–0.64 (m, 3H), 0.68 (s, 3H).

^{13}C NMR (101 MHz, CDCl$_3$) δ = 149.4, 148.6, 146.8, 136.2, 130.2, 129.3, 128.4, 127.7, 125.7, 123.7, 59.0, 48.6, 47.5, 42.8, 42.6, 35.0, 32.9, 31.9, 27.4, 23.3, 21.9.

HR-MS (ESI+) [M+Na]$^+$: 451.1662, calc. for $C_{23}H_{28}N_2O_4SNa$ 451.1662.

7.68 Characterisation of 5-((4-nitrophenyl)sulfonyl)-1,2,3,4,4a,5,6,10b-octahydrophenanthridine (5bg)

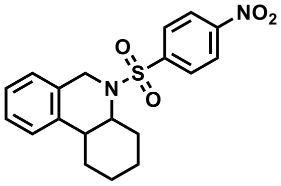

5bg

The compound was synthesized according to GP2 yielding 21.9 mg (0.059 mmol, 59%) as an off-white solid.

^{1}H NMR (400 MHz, $CDCl_3$) δ = 8.05–7.97 (m, 2H), 7.72–7.63 (m, 2H), 7.17–7.07 (m, 1H), 6.99 (d, J = 7.6 Hz, 1H), 6.96–6.89 (m, 1H), 6.77 (d, J = 7.4 Hz, 1H), 4.59 (d, J = 15.9 Hz, 1H), 4.32 (d, J = 15.9 Hz, 1H), 2.96 (td, J = 11.1, 3.4 Hz, 1H), 2.57–2.49 (m, 1H), 2.48–2.36 (m, 2H), 1.99–1.89 (m, 2H), 1.82–1.67 (m, 1H), 1.51–1.36 (m, 3H).

^{13}C NMR (101 MHz, $CDCl_3$) δ = 149.6, 144.9, 138.7, 134.7, 128.7, 128.1, 125.9, 125.5, 123.7, 123.1, 62.1, 46.8, 41.9, 35.3, 28.3, 25.6, 25.6.

HR-MS (ESI+) $[M+Na]^+$: 395.1036, calc. for $C_{19}H_{20}N_2O_4SNa$ 395.1036.

8 Spectral Information

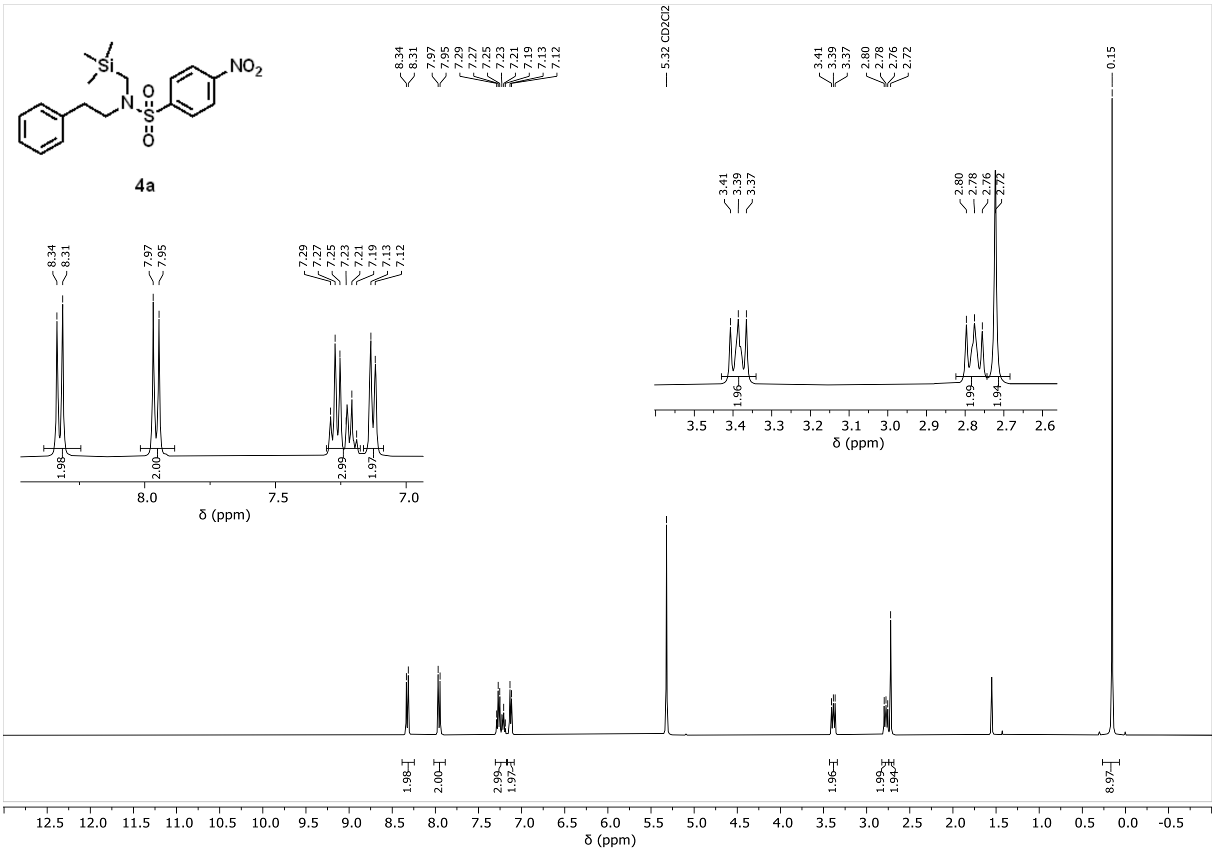

Figure **S5**: ^{1}H NMR (400 MHz, CD_2Cl_2) of **4a**.

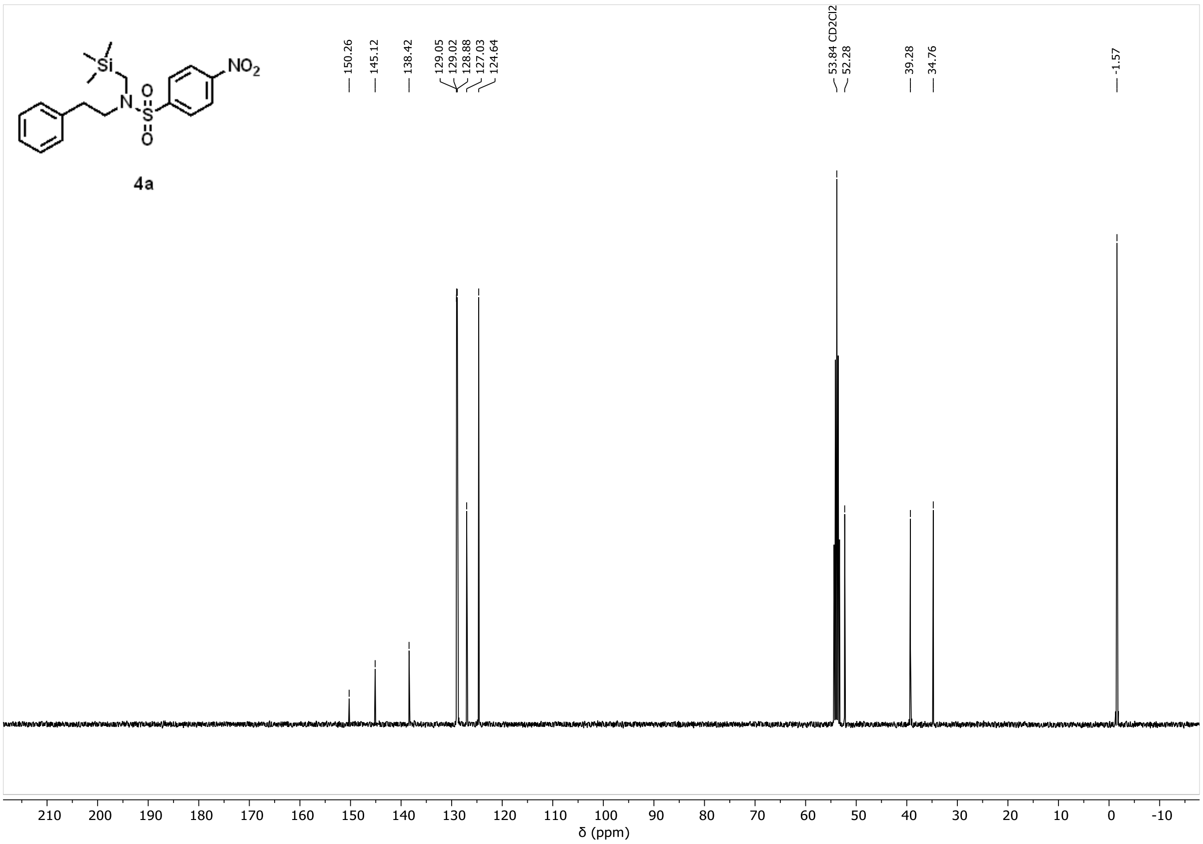

Figure **S6**: ^{13}C NMR (101 MHz, CD_2Cl_2) of **4a**.

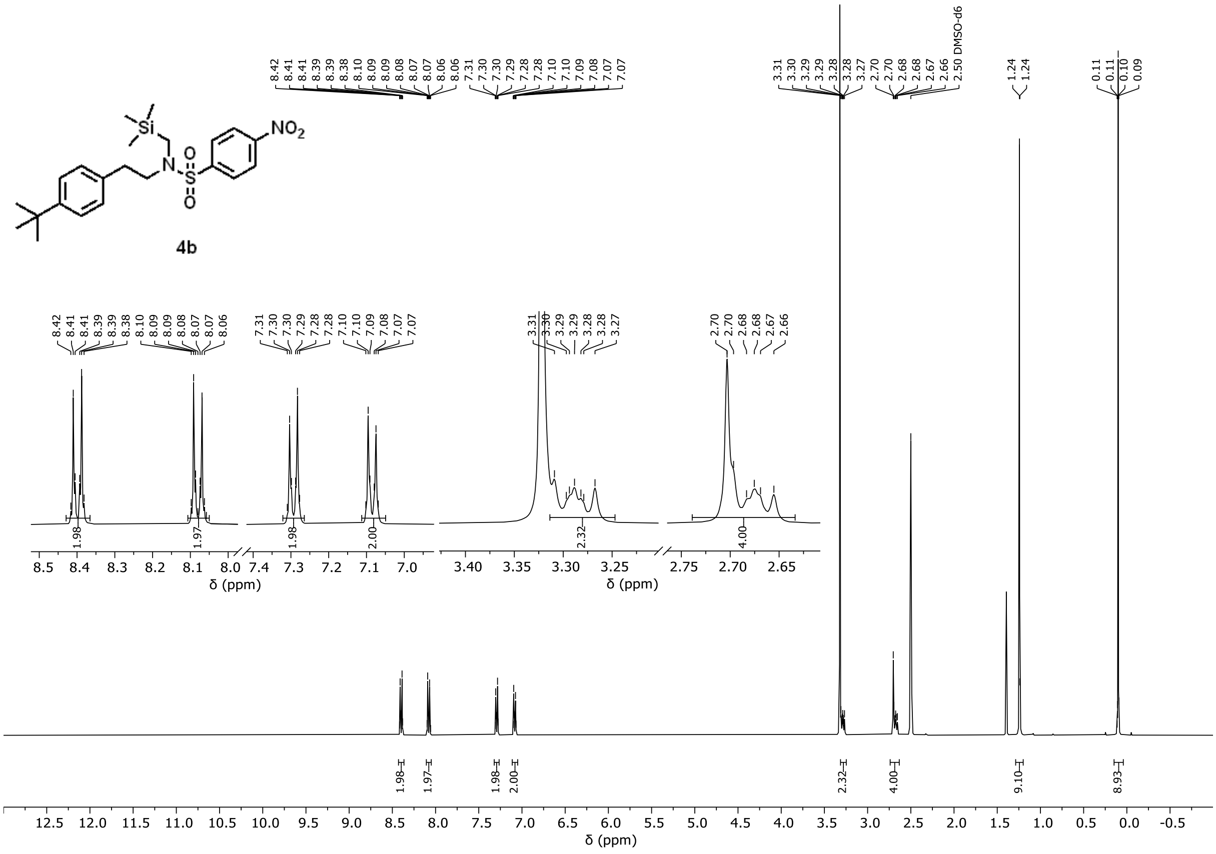

Figure **S7**: ^{1}H NMR (400 MHz, DMSO-d$_6$) of **4b**.

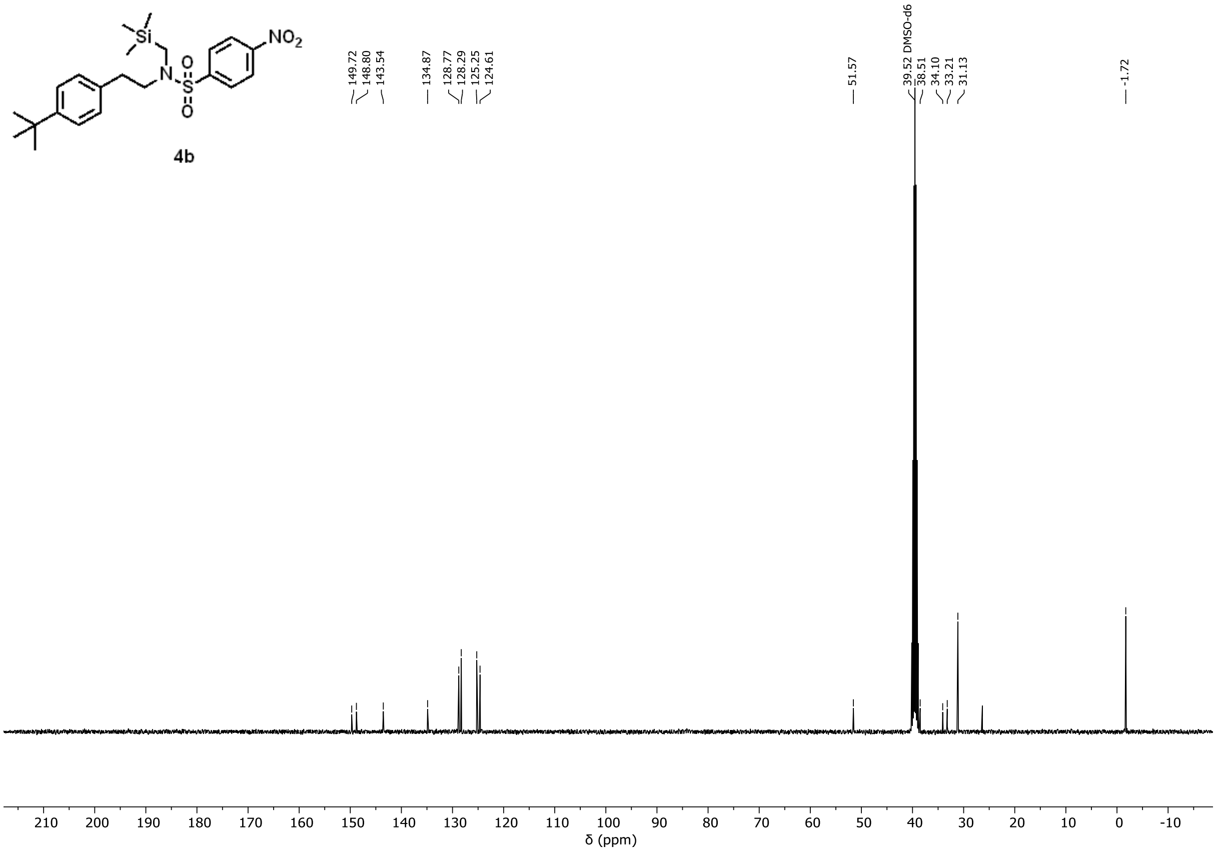

Figure **S8**: ^{13}C NMR (101 MHz, DMSO-d$_6$) of **4b**.

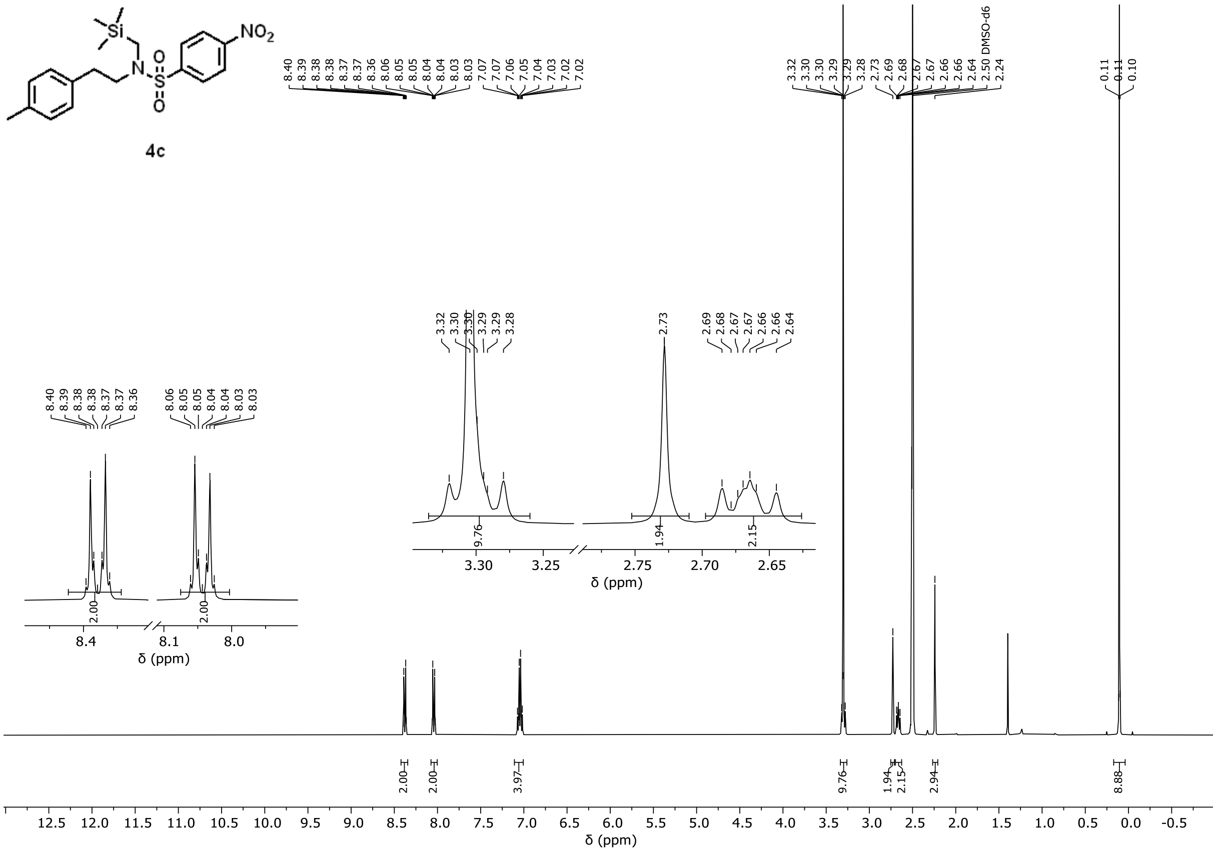

Figure **S9**: ^{1}H NMR (400 MHz, DMSO-d$_6$) of **4c**.

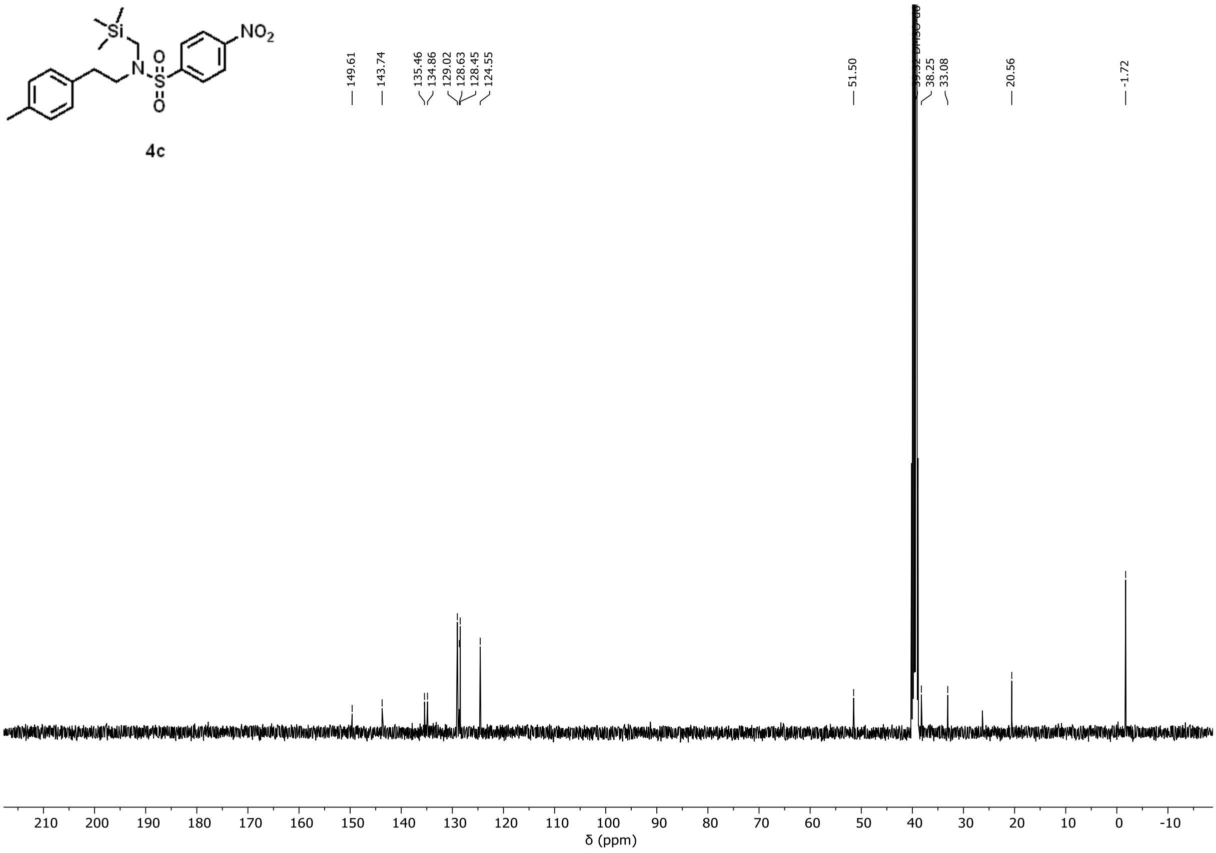

Figure **S10**: ^{13}C NMR (101 MHz, DMSO-d$_6$) of **4c**.

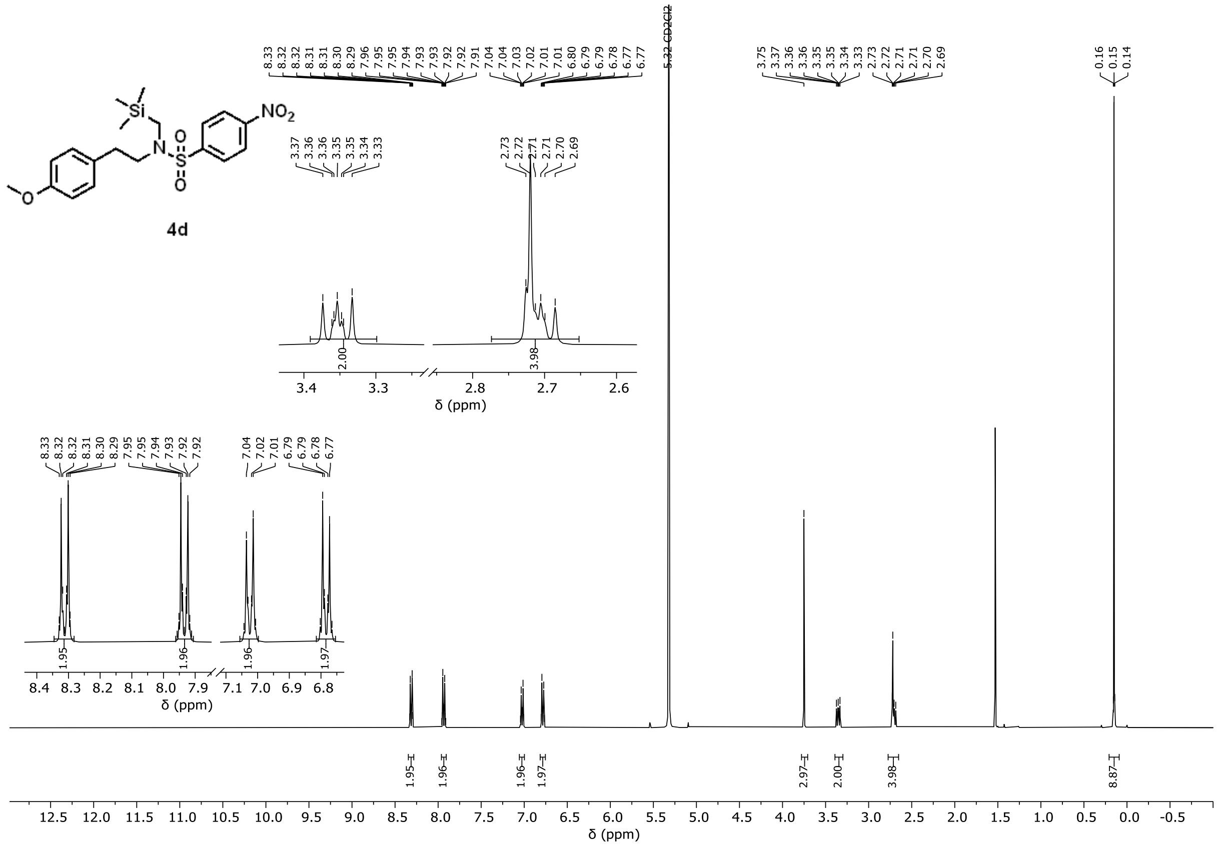

Figure **S11**: ^{1}H NMR (400 MHz, CD_2Cl_2) of **4d**.

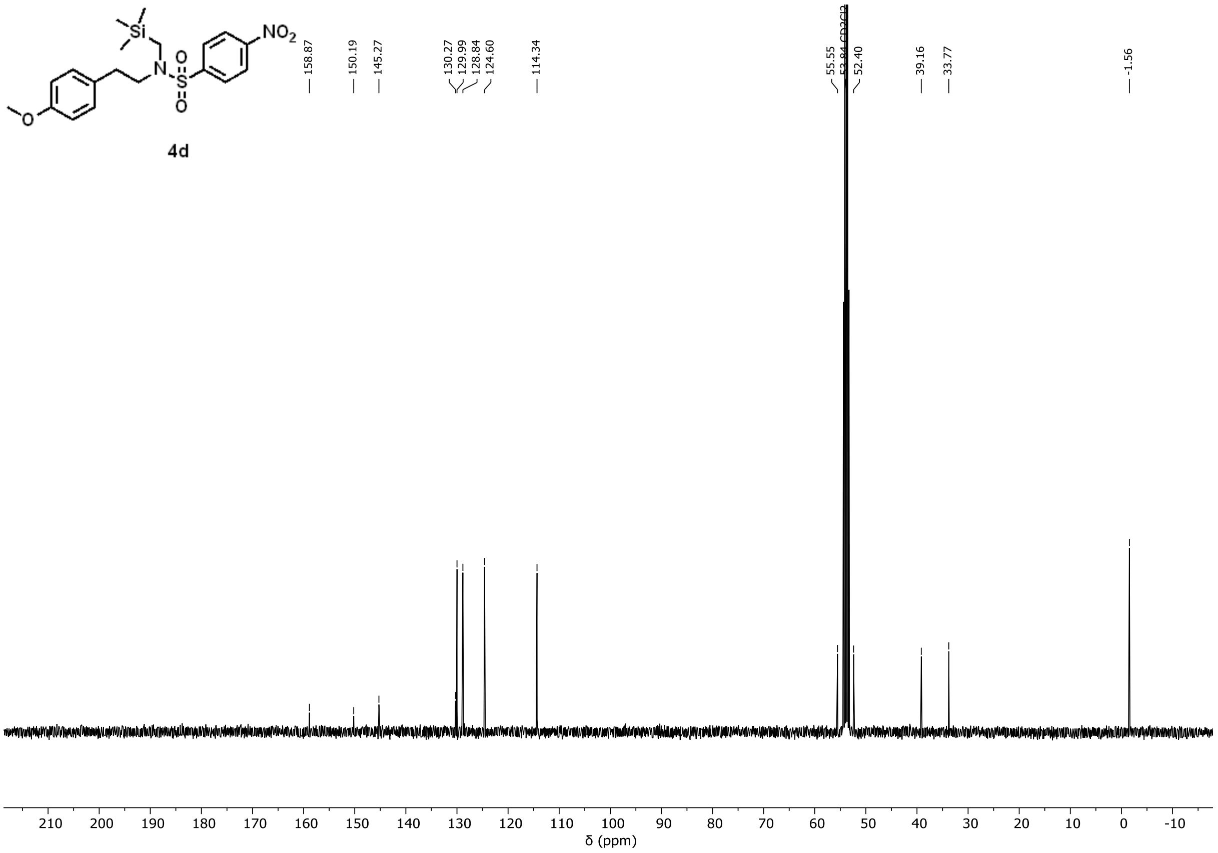

Figure **S12**: ^{13}C NMR (101 MHz, CD_2Cl_2) of **4d**.

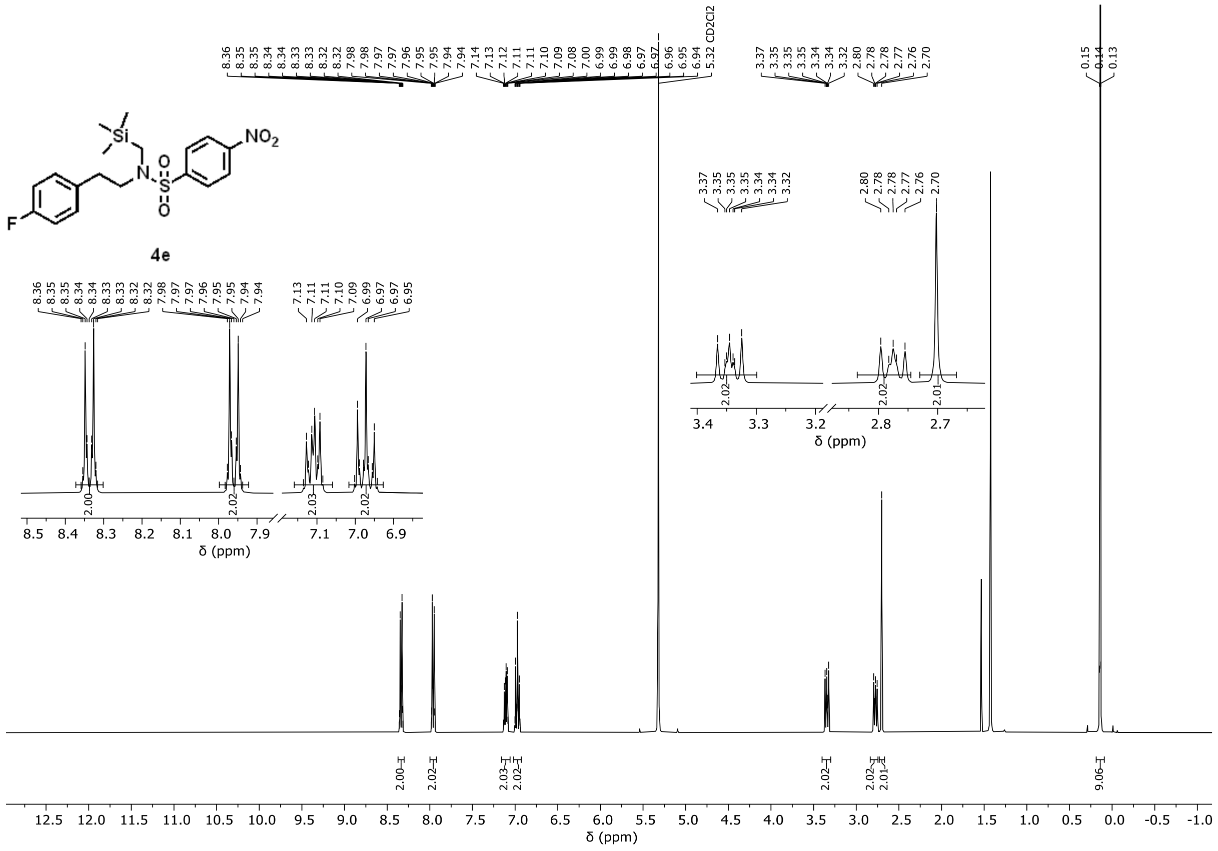

Figure **S13**: ^1H NMR (400 MHz, CD_2Cl_2) of **4e**.

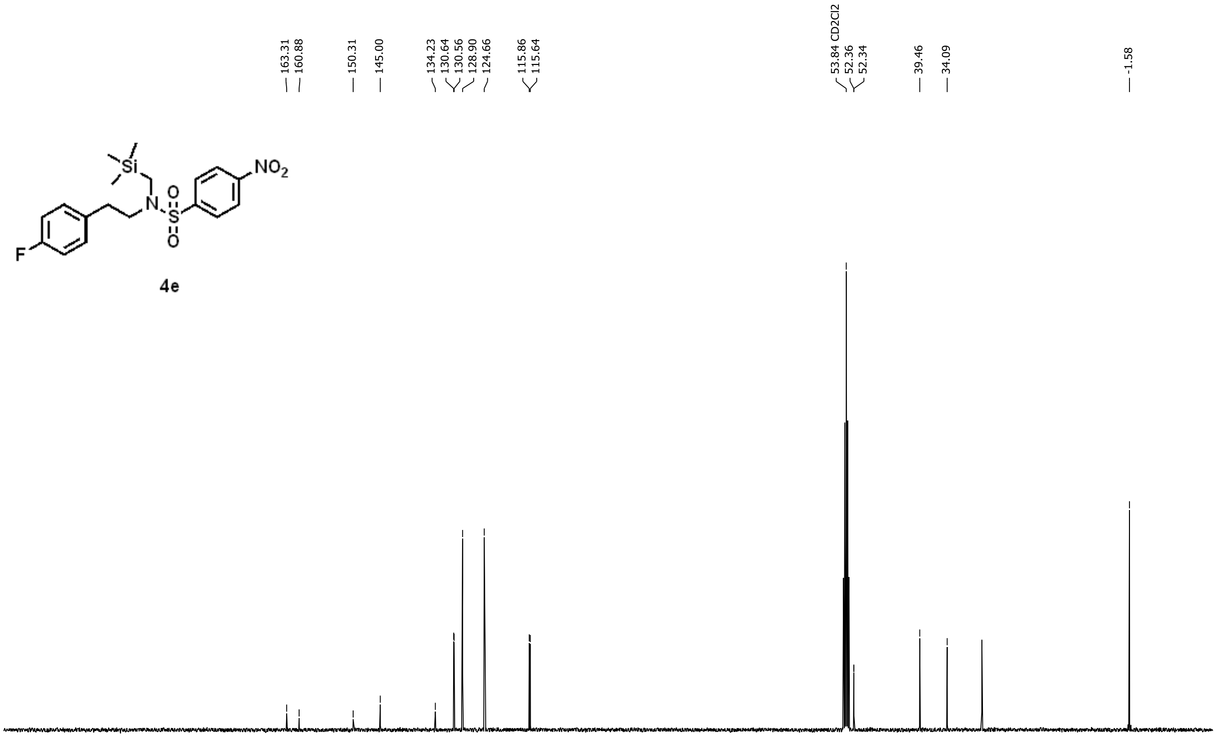

Figure **S14**: ^{13}C NMR (101 MHz, CD_2Cl_2) of **4e**.

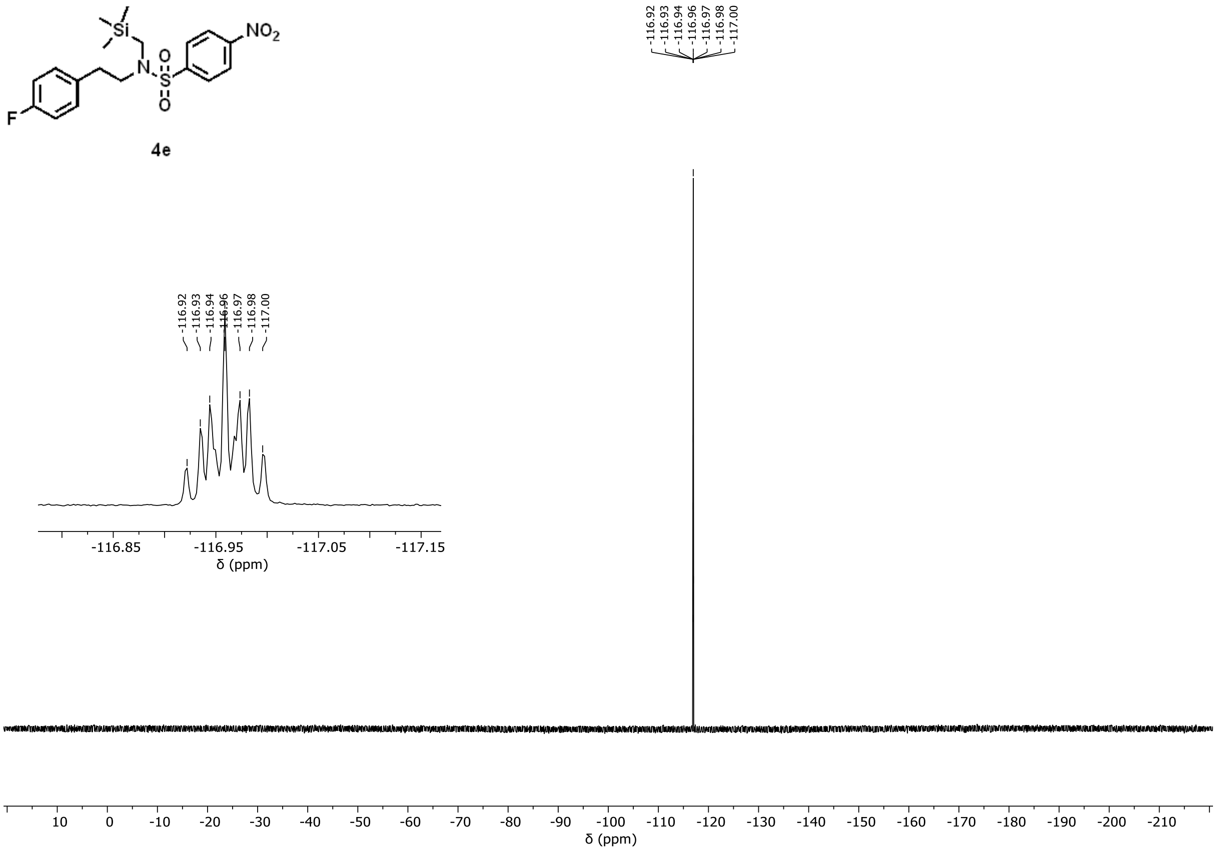

Figure **S15**: ^{19}F NMR (376 MHz, CD_2Cl_2) of **4e**.

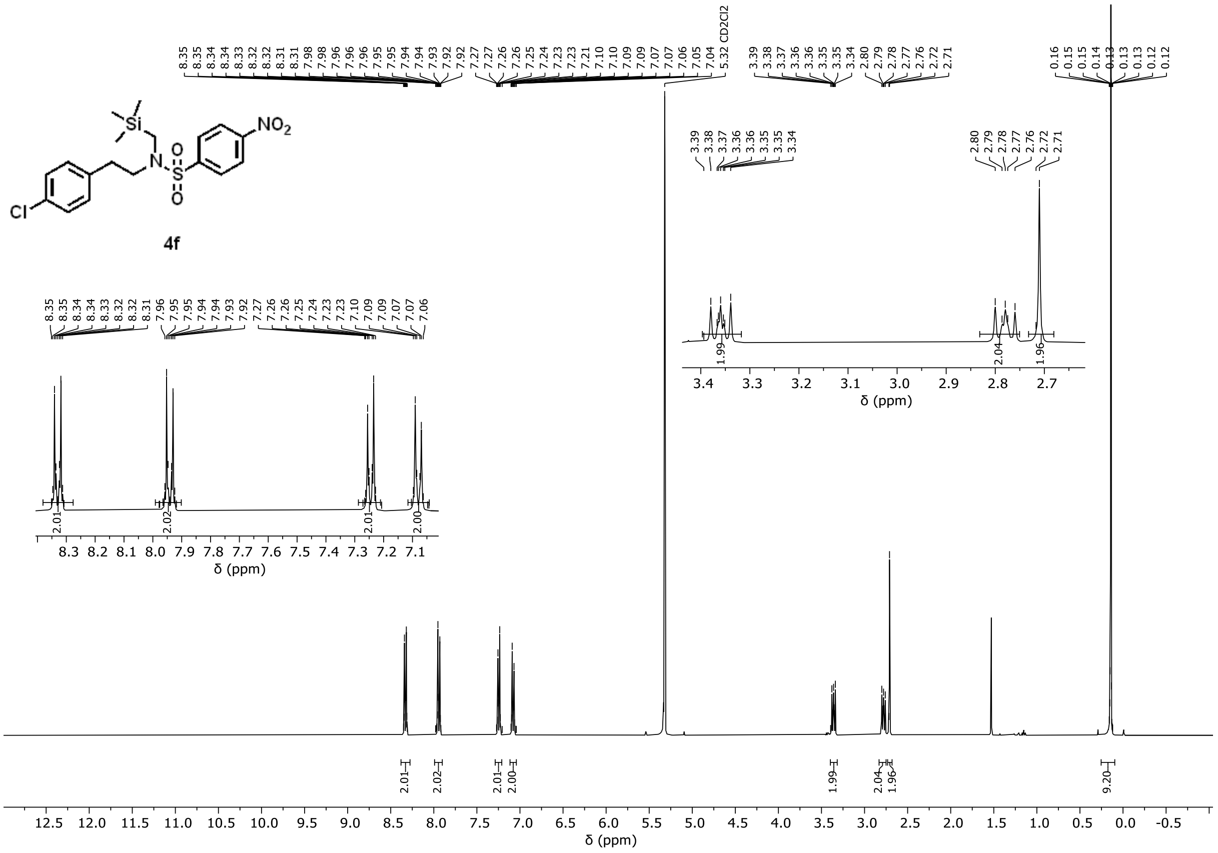

Figure **S16**: ^{1}H NMR (400 MHz, CD_2Cl_2) of **4f**.

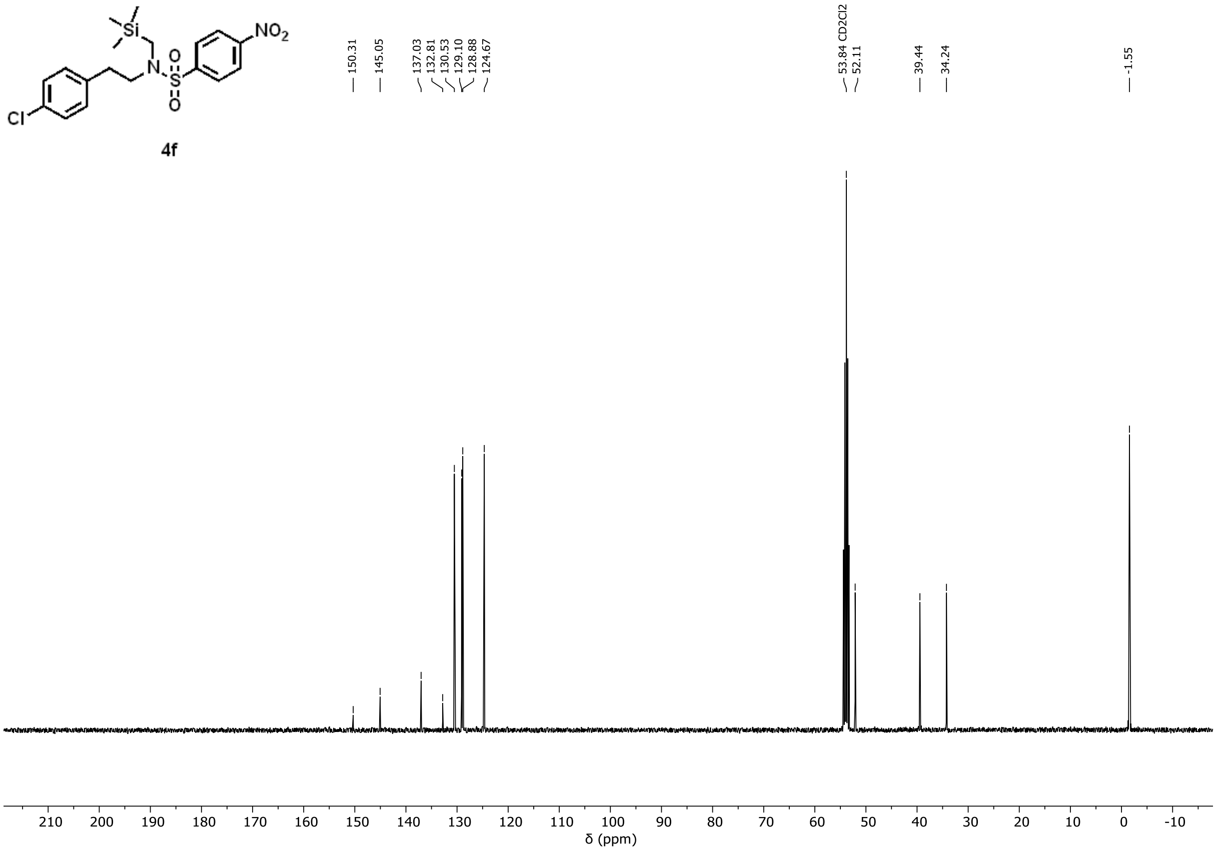

Figure **S17**: ^{13}C NMR (101 MHz, CD_2Cl_2) of **4f**.

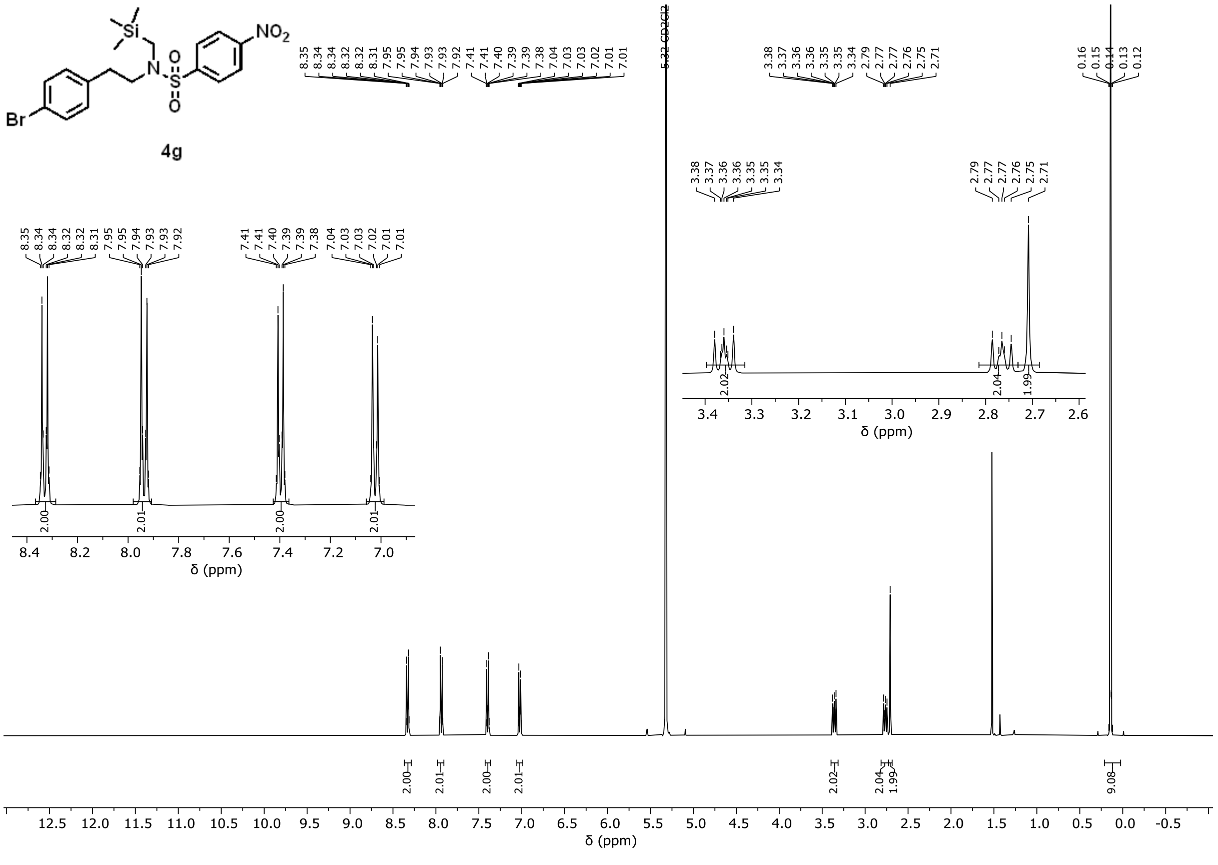

Figure **S18**: ^{1}H NMR (400 MHz, CD_2Cl_2) of **4g**.

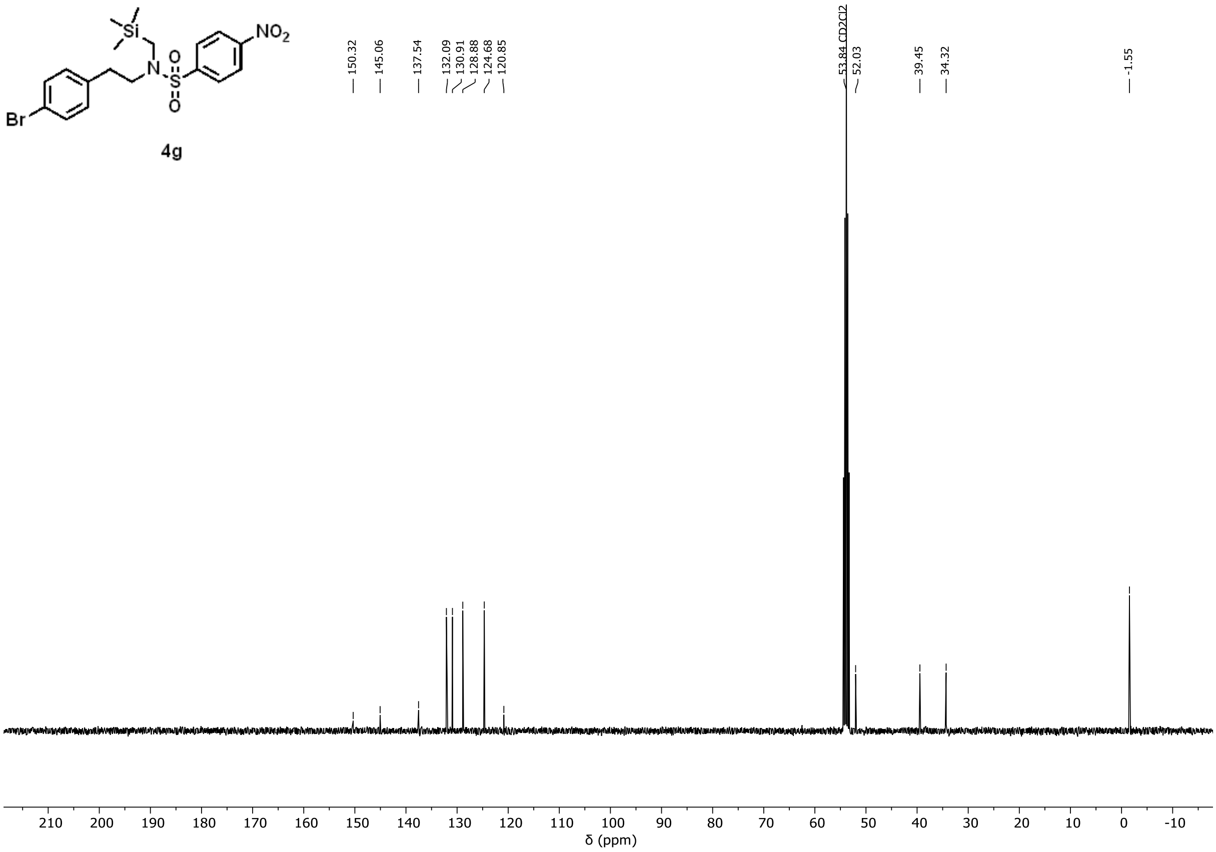

Figure **S19**: ^{13}C NMR (101 MHz, CD_2Cl_2) of **4g**.

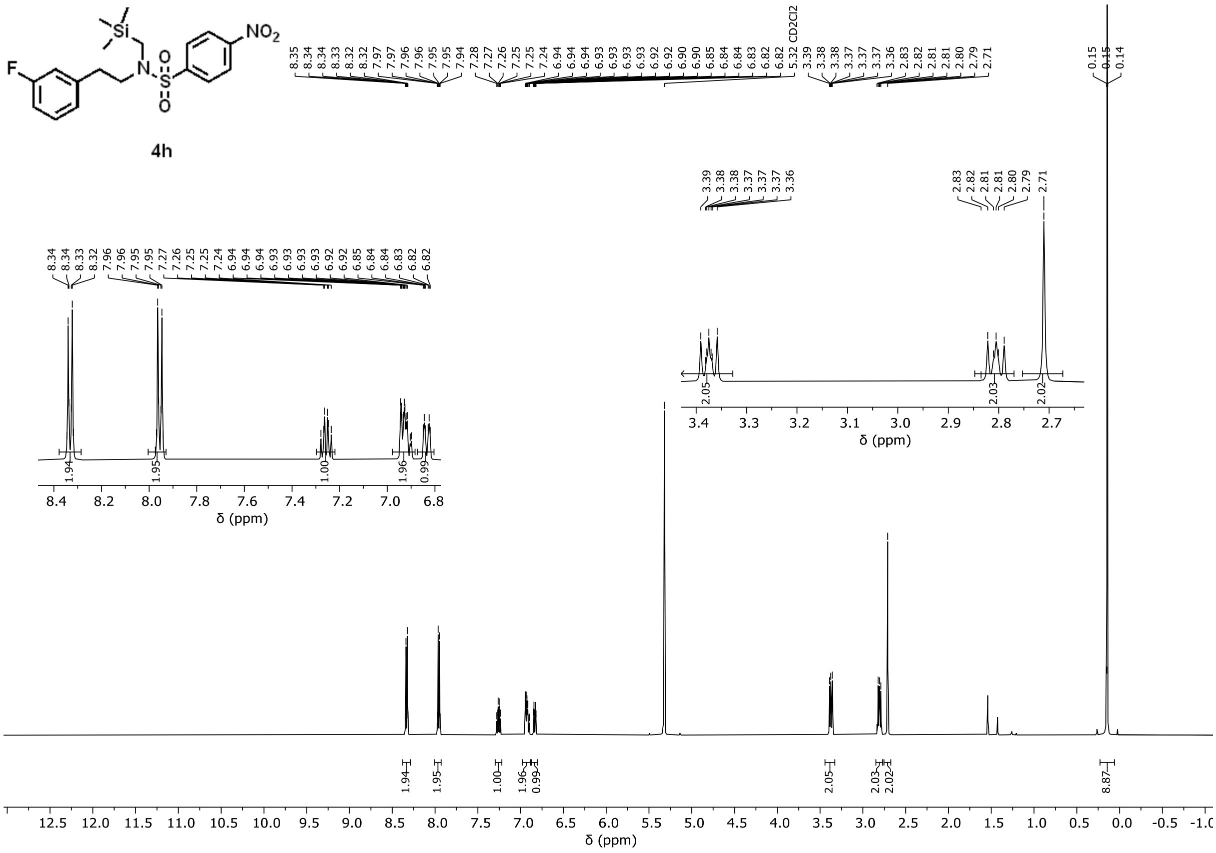

Figure **S20**: ^{1}H NMR (400 MHz, CD_2Cl_2) of **4h**.

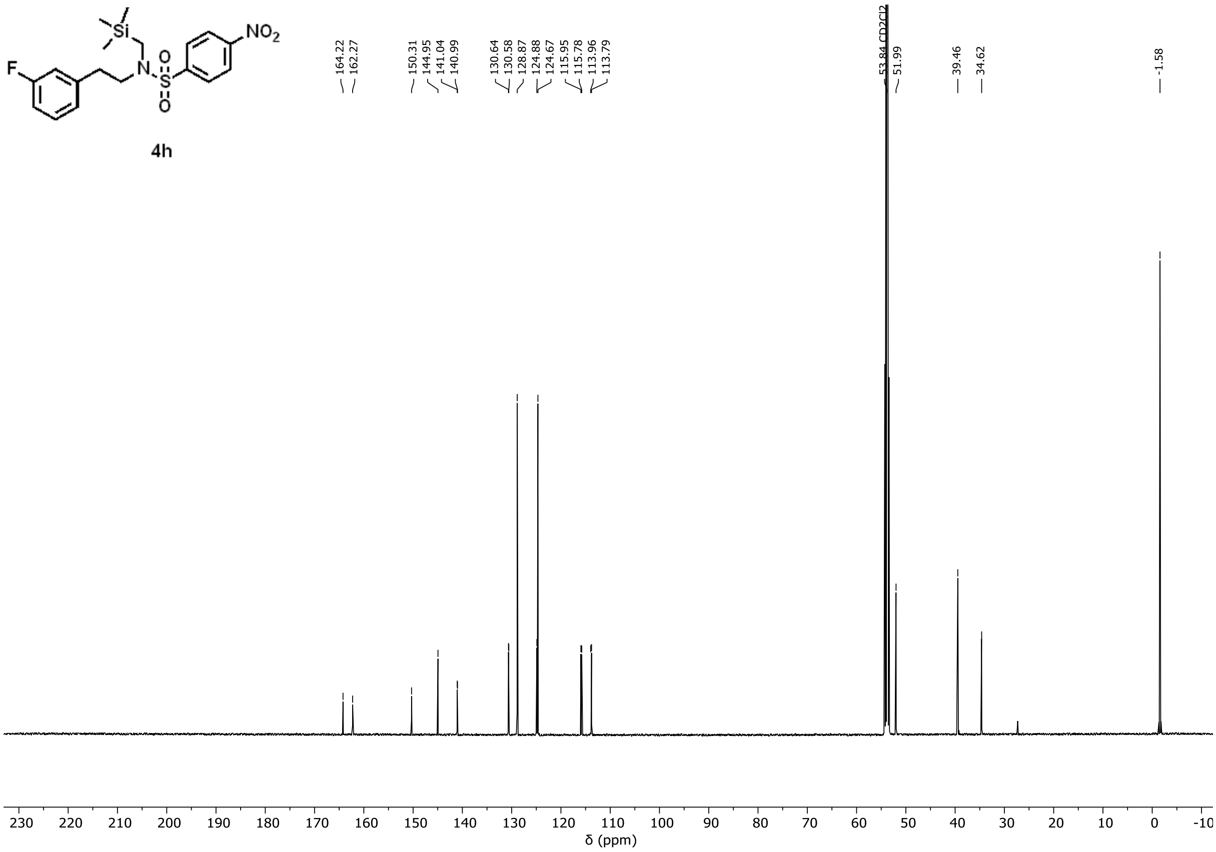

Figure **S21**: ^{13}C NMR (101 MHz, CD_2Cl_2) of **4h**.

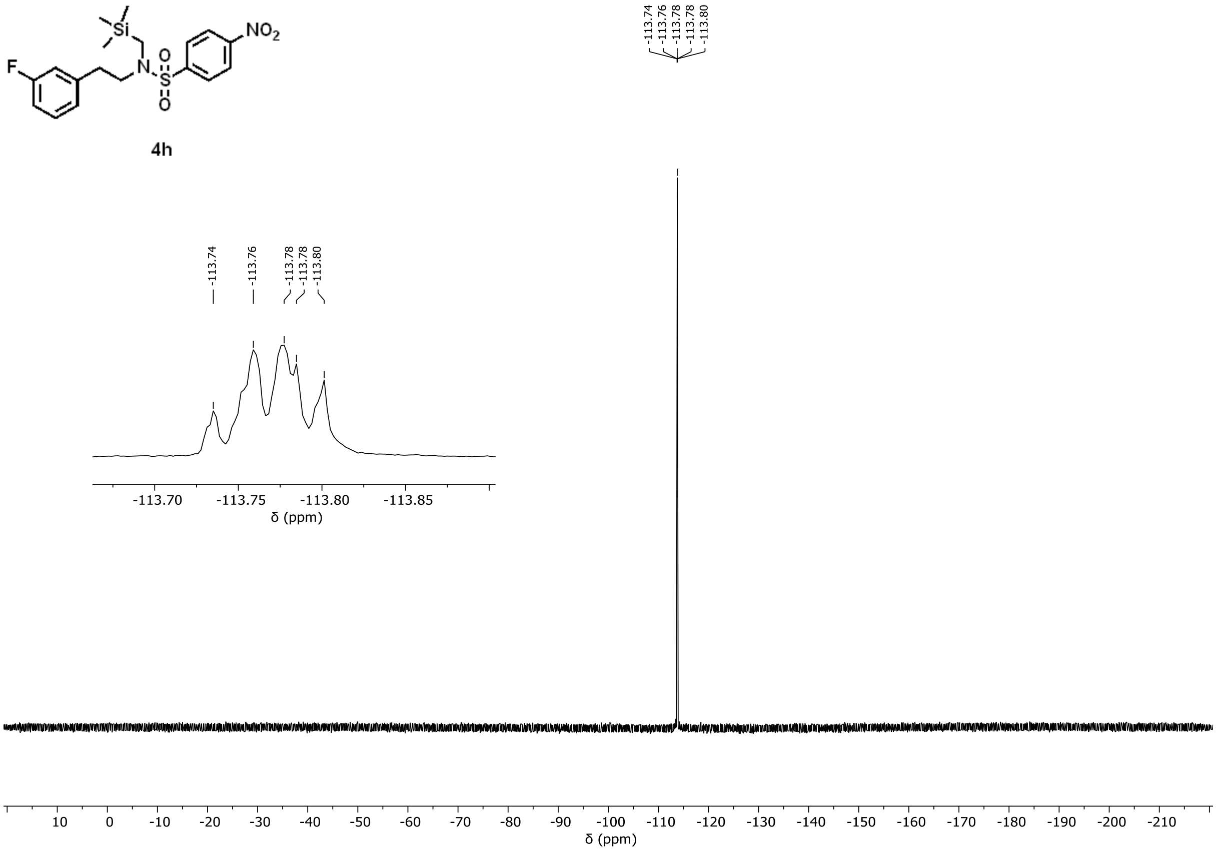

Figure **S22**: ^{19}F NMR (376 MHz, CD_2Cl_2) of **4h**.

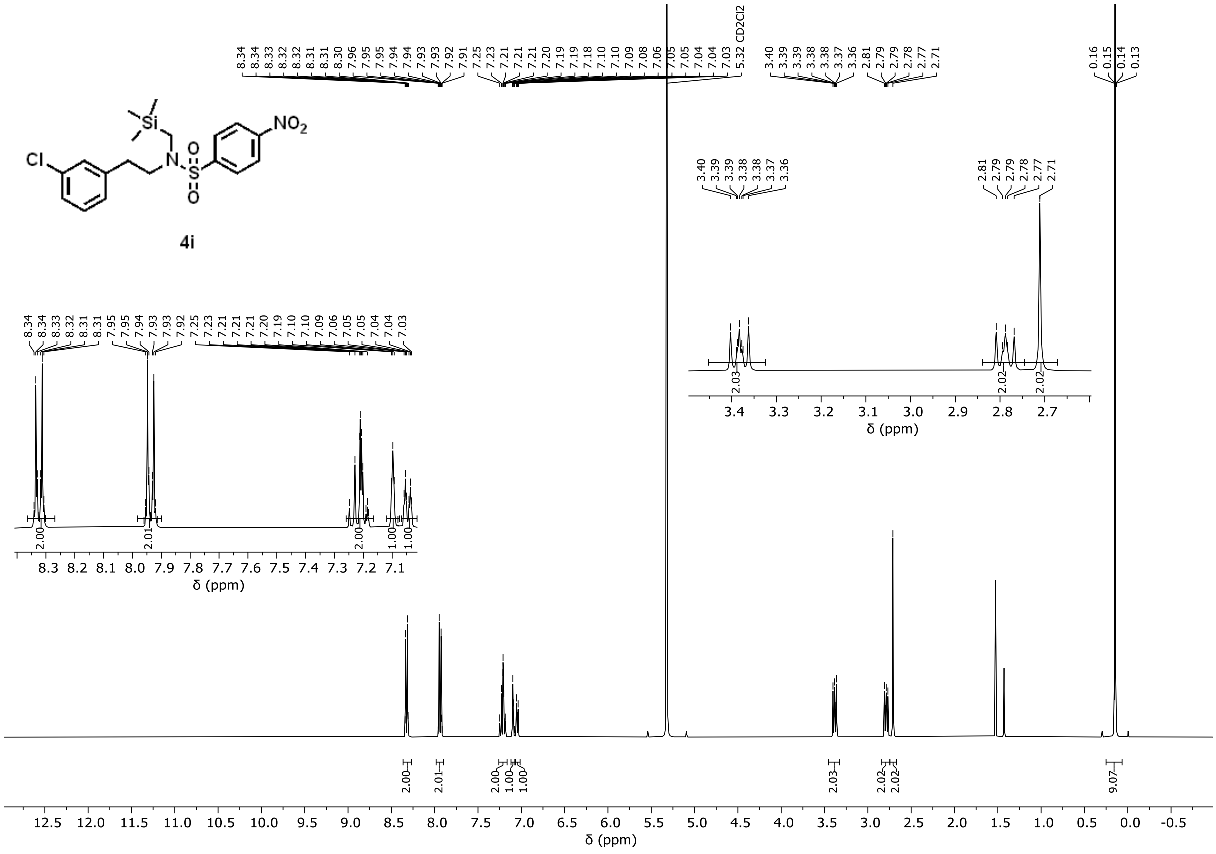

Figure **S23**: ^{1}H NMR (400 MHz, CD_2Cl_2) of **4i**.

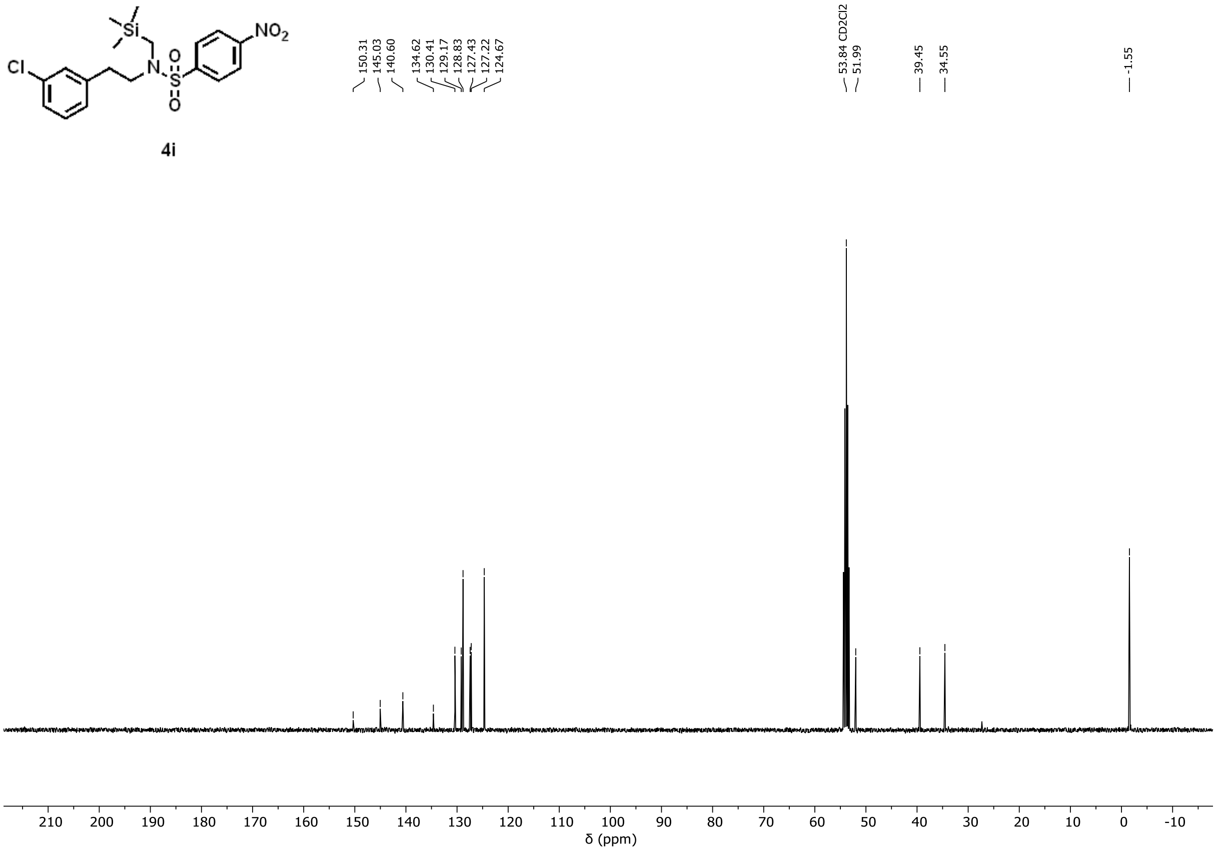

Figure **S24**: ^{13}C NMR (101 MHz, CD_2Cl_2) of **4i**.

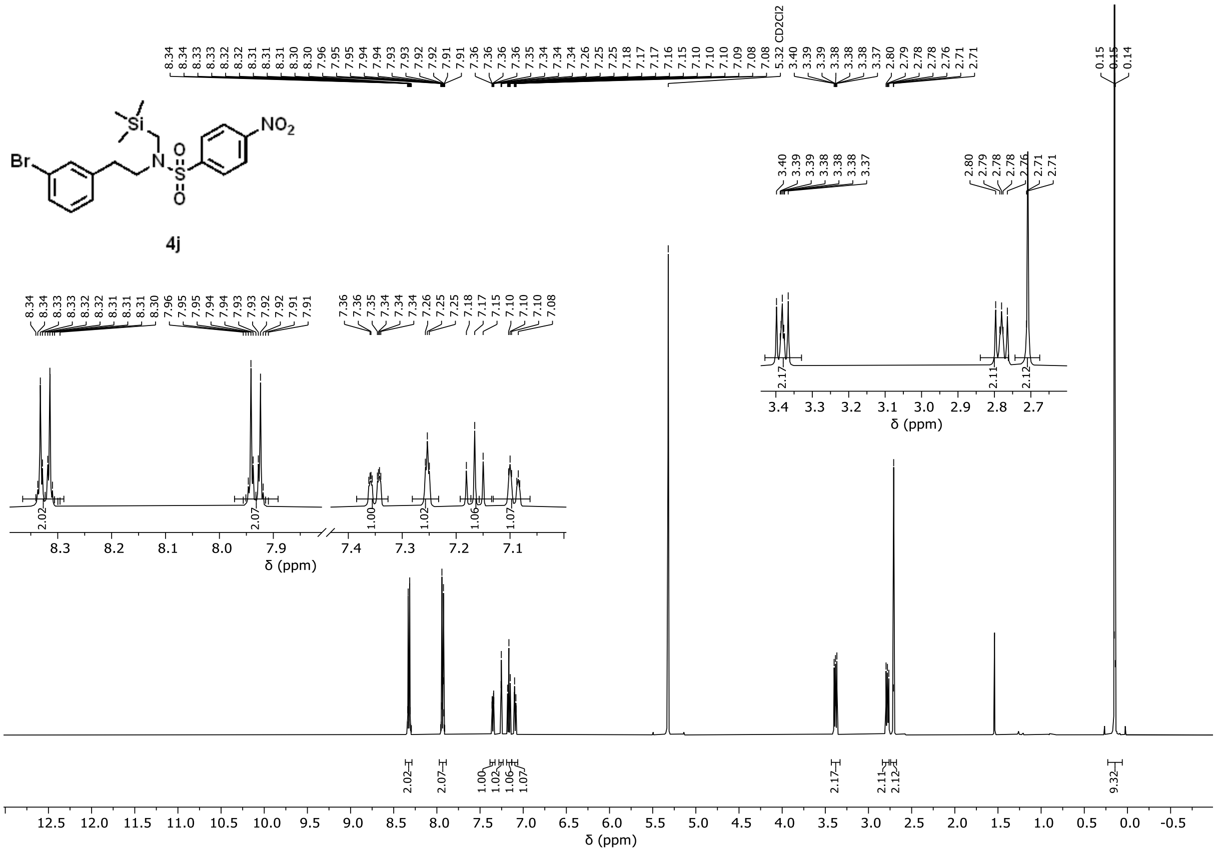

Figure **S25**: ^{1}H NMR (400 MHz, CD_2Cl_2) of **4j**.

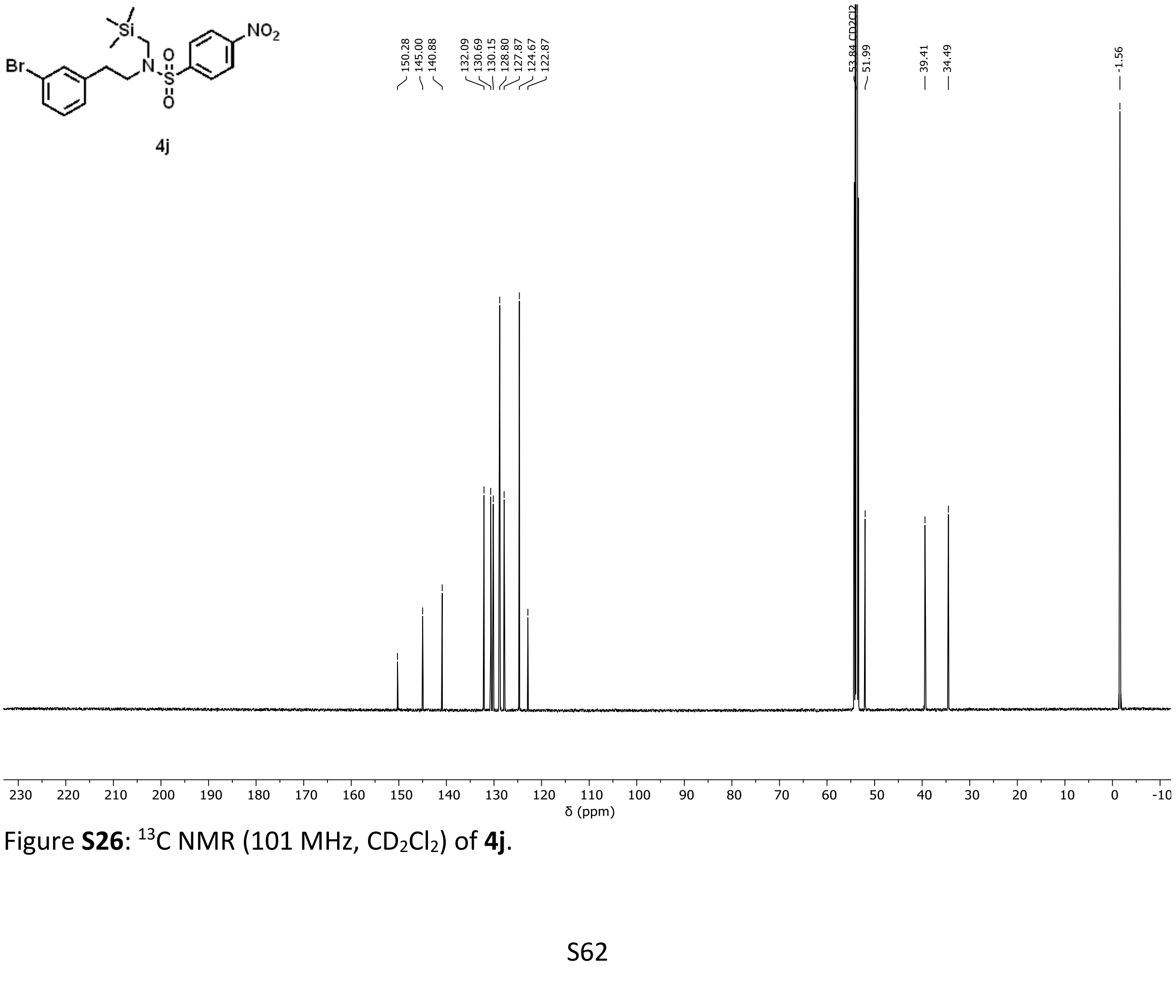

Figure **S26**: ^{13}C NMR (101 MHz, CD_2Cl_2) of **4j**.

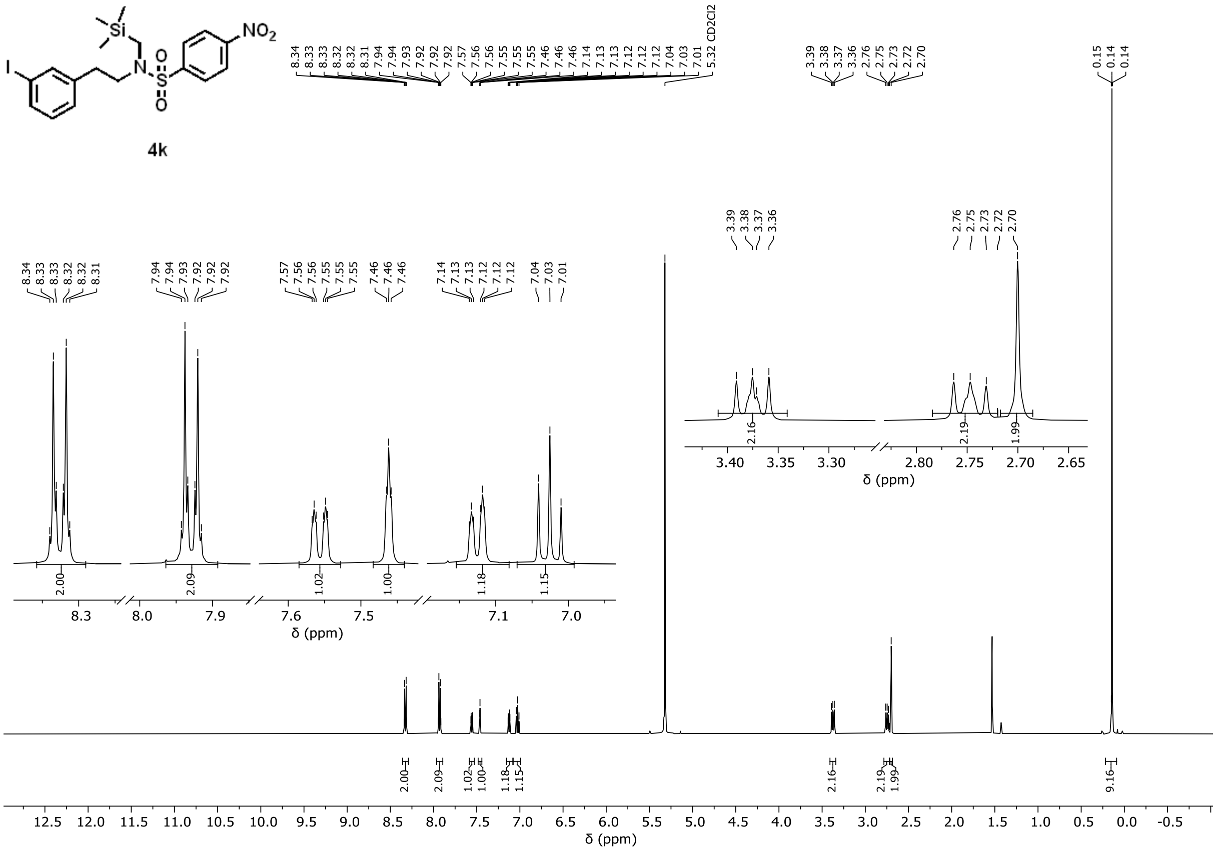

Figure **S27**: ^{1}H NMR (400 MHz, CD_2Cl_2) of **4k**.

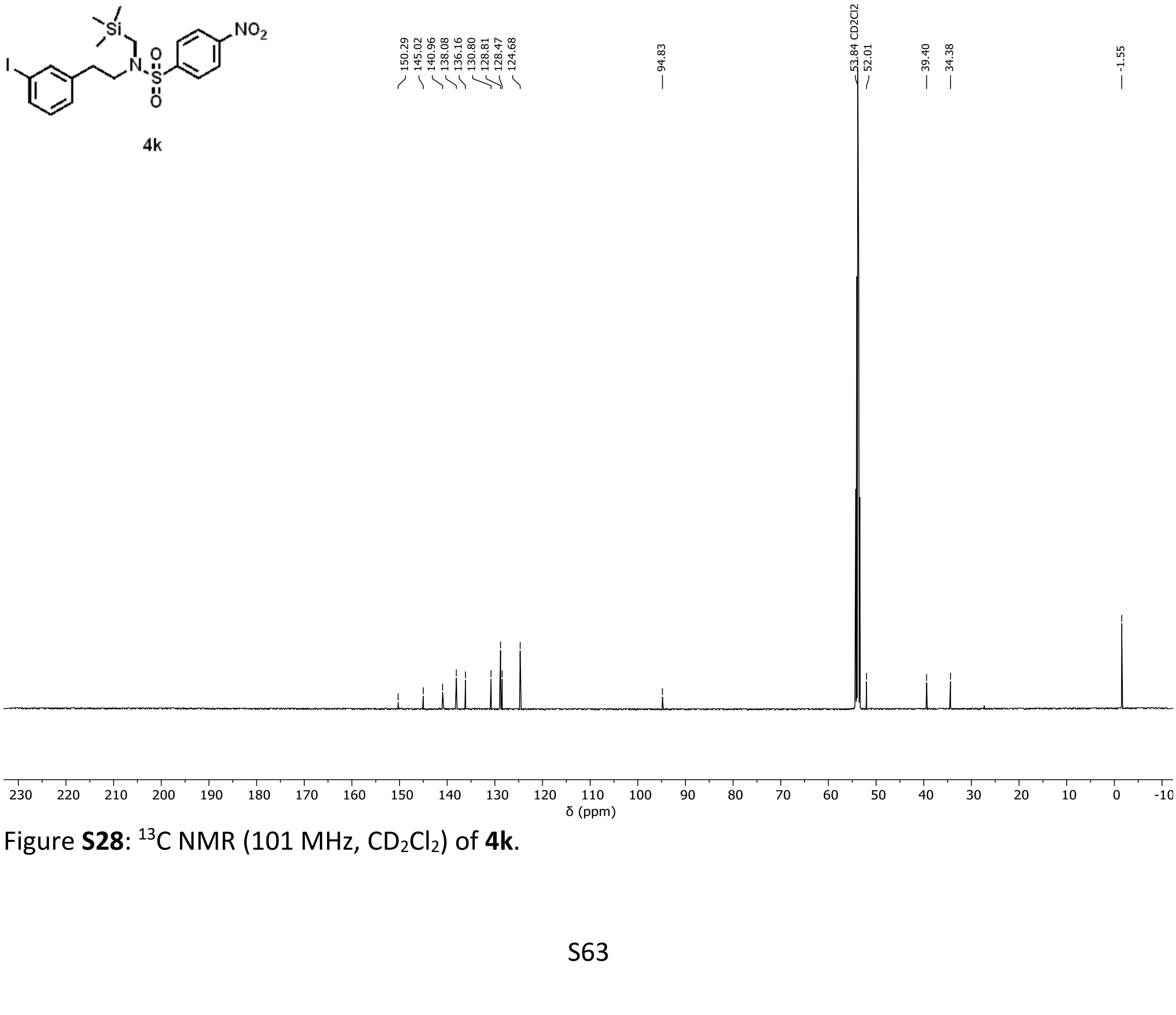

Figure **S28**: ^{13}C NMR (101 MHz, CD_2Cl_2) of **4k**.

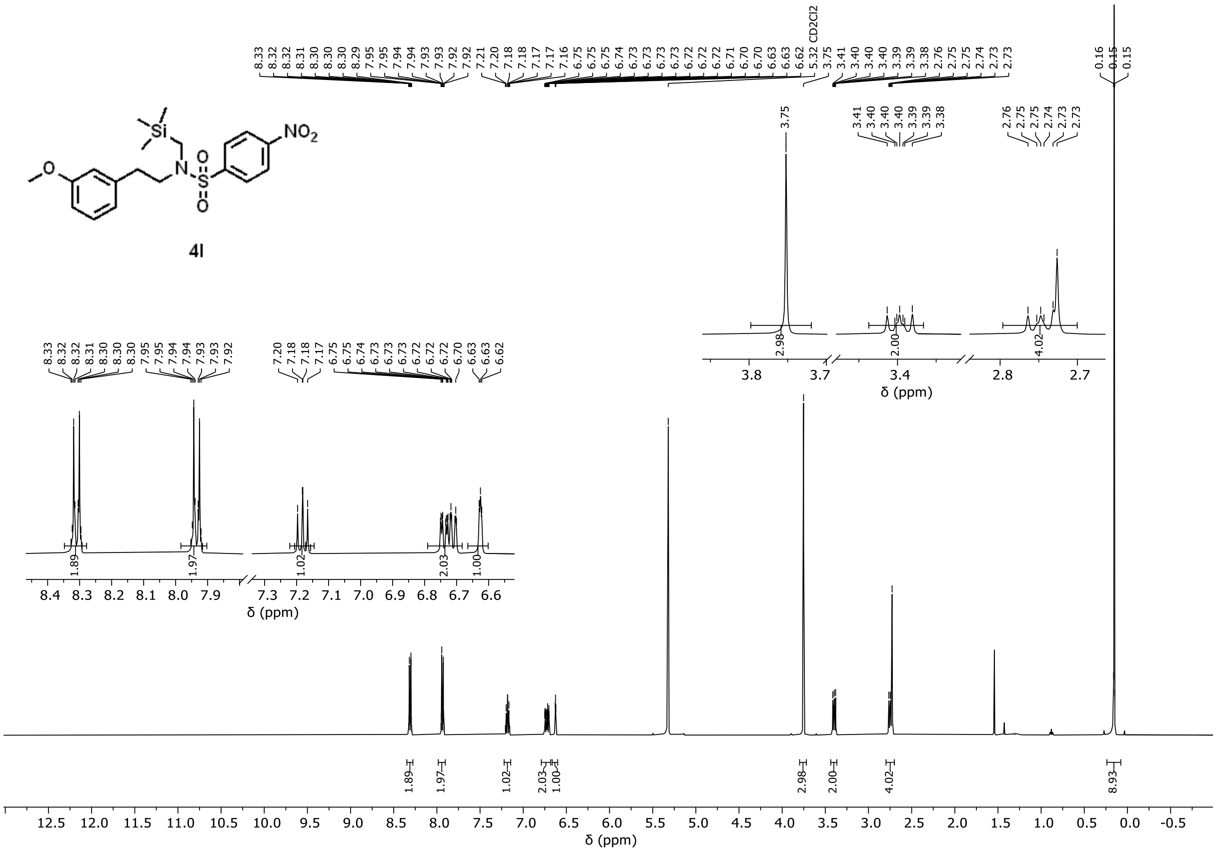

Figure **S29**: ^{1}H NMR (400 MHz, CD_2Cl_2) of **4l**.

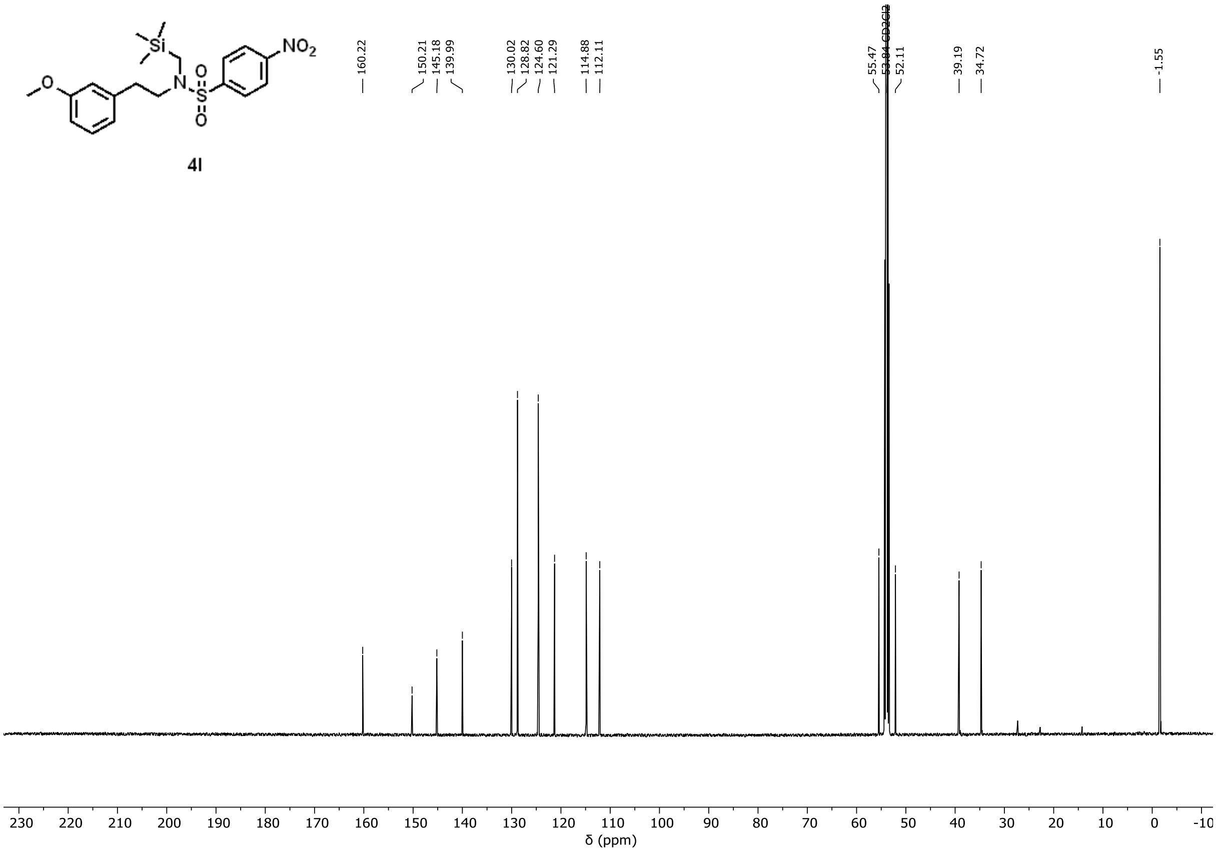

Figure **S30**: ^{13}C NMR (101 MHz, CD_2Cl_2) of **4l**.

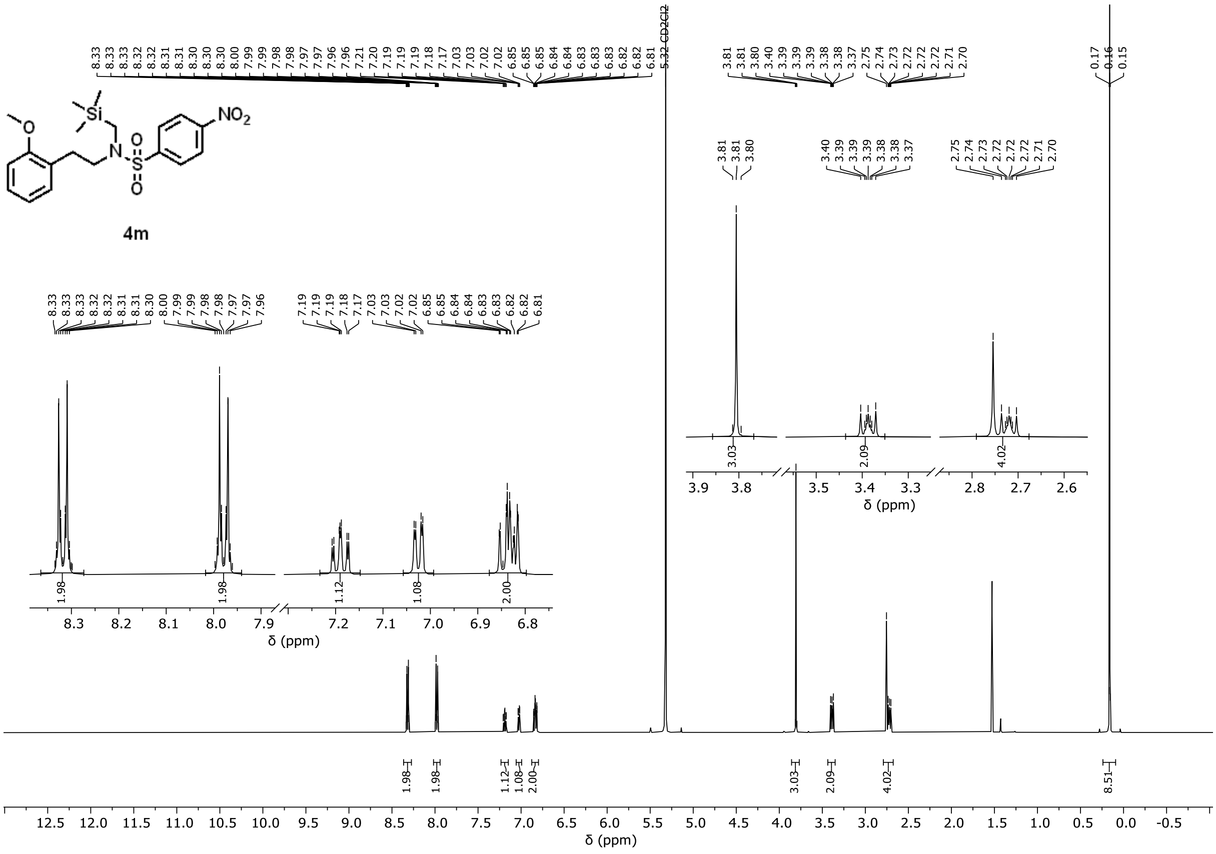

Figure **S31**: ^{1}H NMR (400 MHz, CD_2Cl_2) of **4m**.

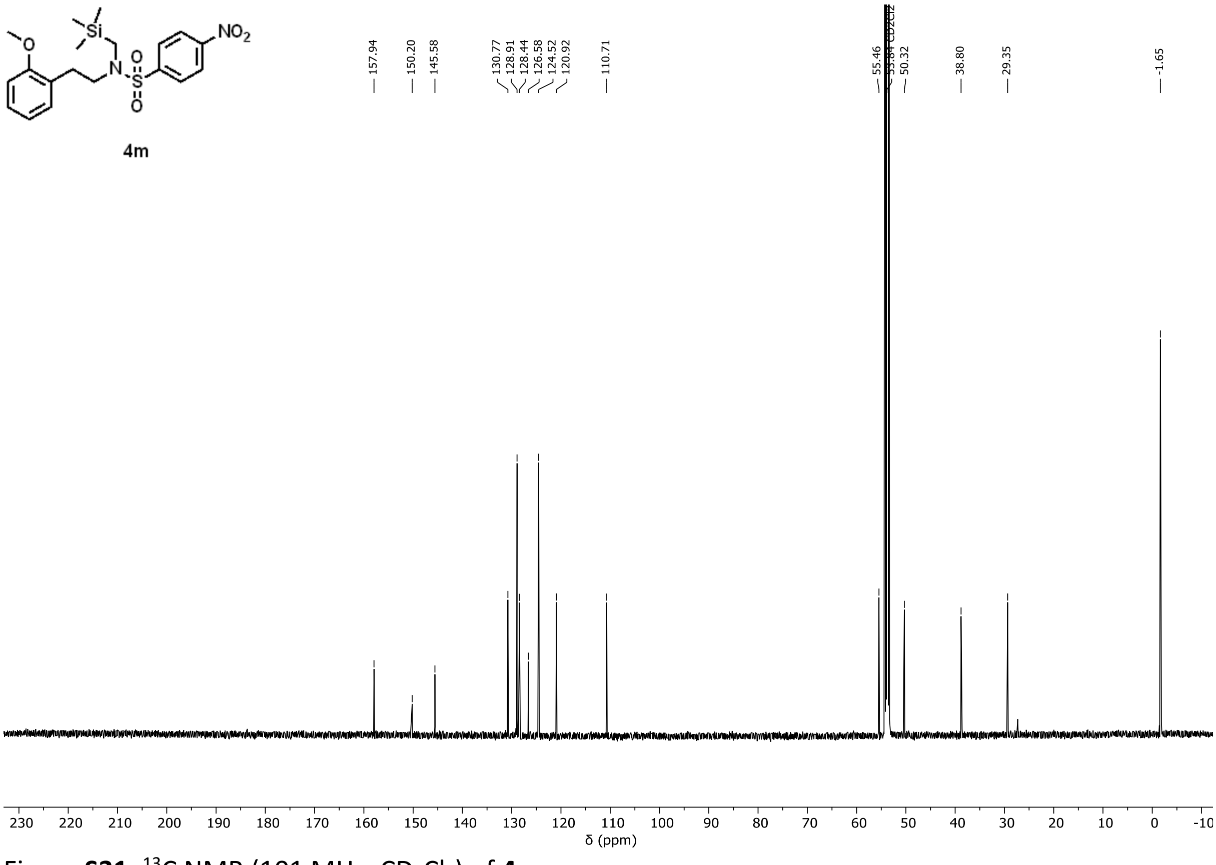

Figure **S31**: ^{13}C NMR (101 MHz, CD_2Cl_2) of **4m**.

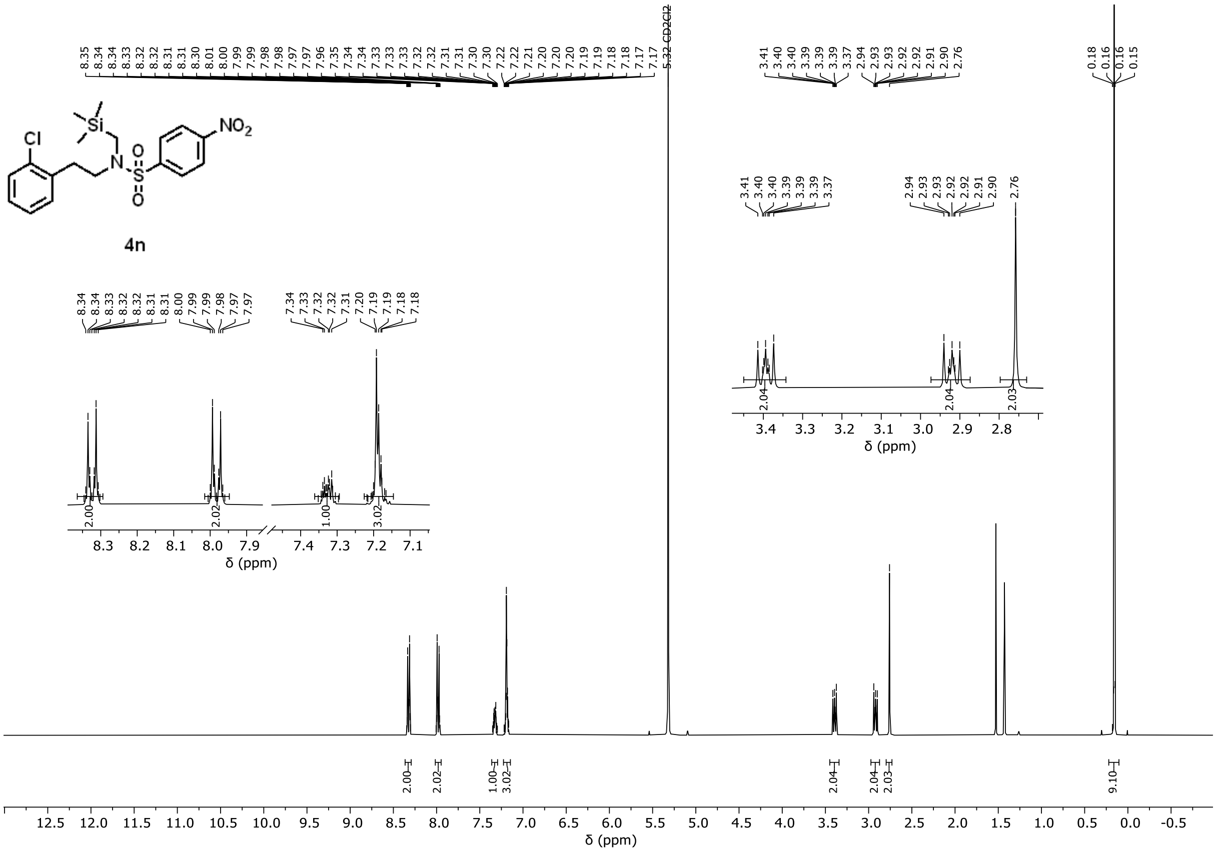

Figure **S32**: ^{1}H NMR (400 MHz, CD_2Cl_2) of **4n**.

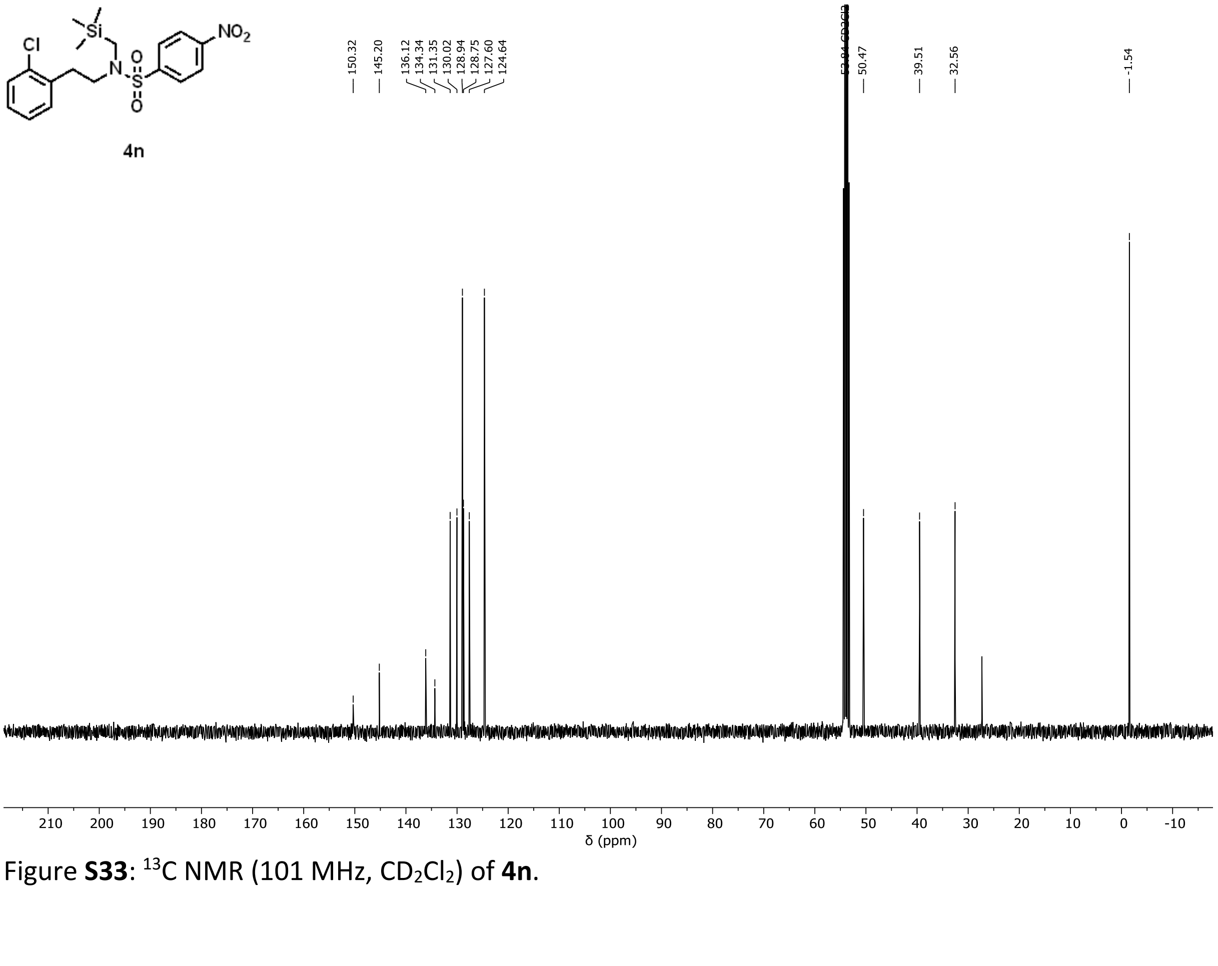

Figure **S33**: ^{13}C NMR (101 MHz, CD_2Cl_2) of **4n**.

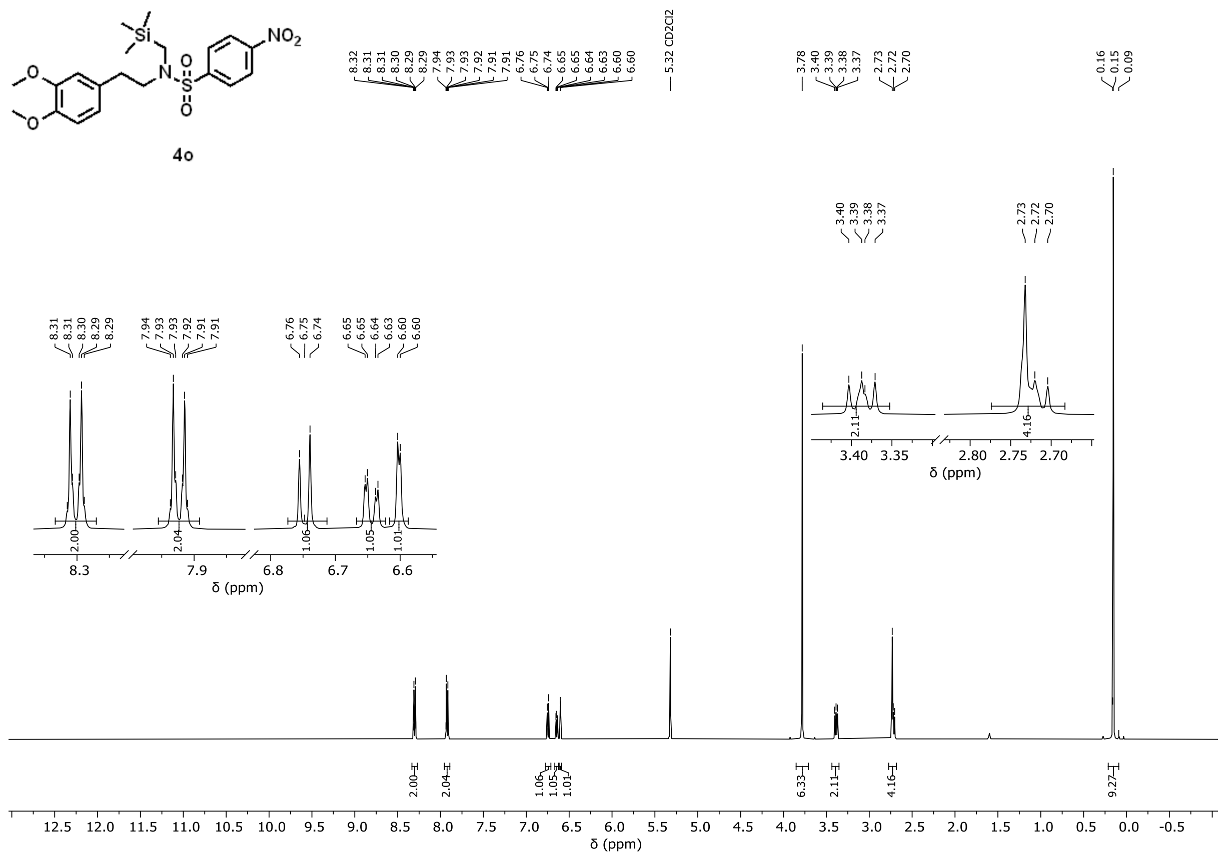

Figure **S34**: ^{1}H NMR (400 MHz, CD_2Cl_2) of **4o**.

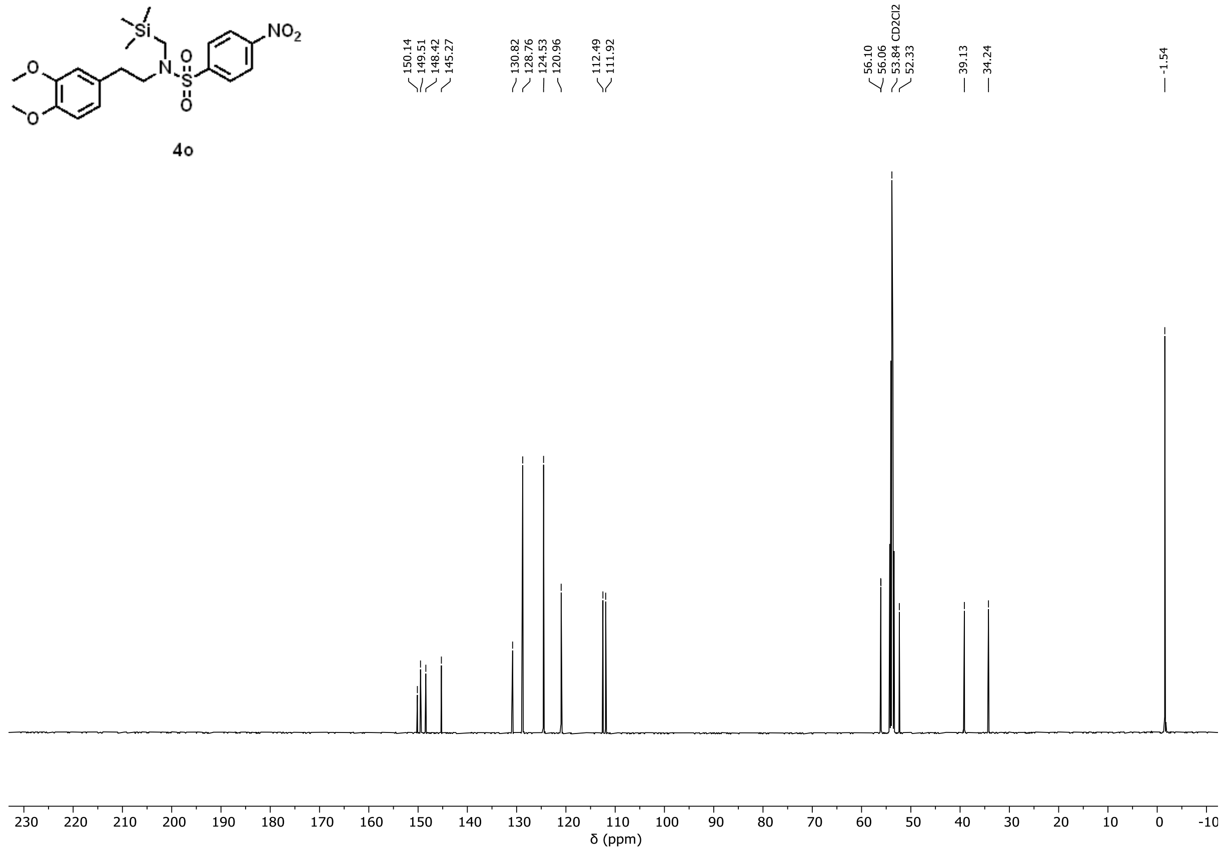

Figure **S35**: ^{13}C NMR (101 MHz, CD_2Cl_2) of **4o**.

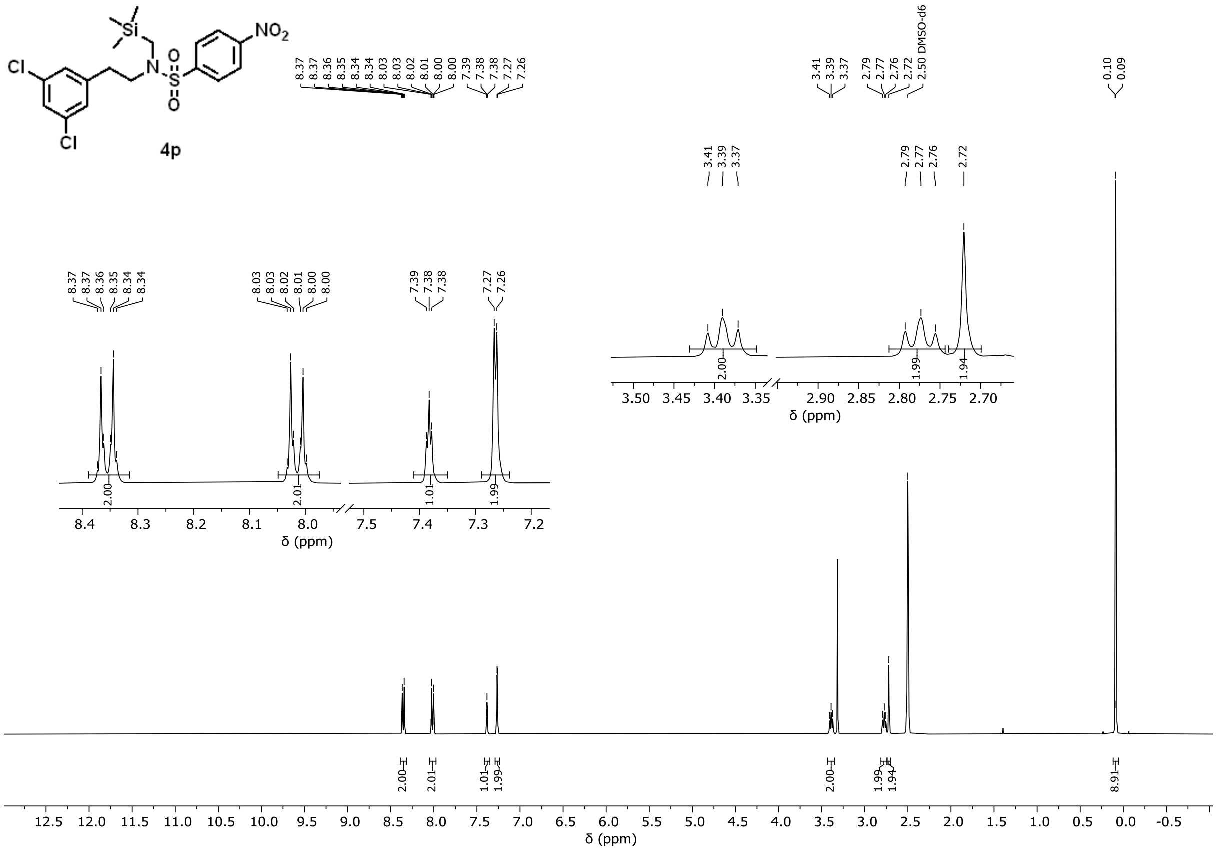

Figure **S36**: ^{1}H NMR (500 MHz, DMSO-d$_6$) of **4p**.

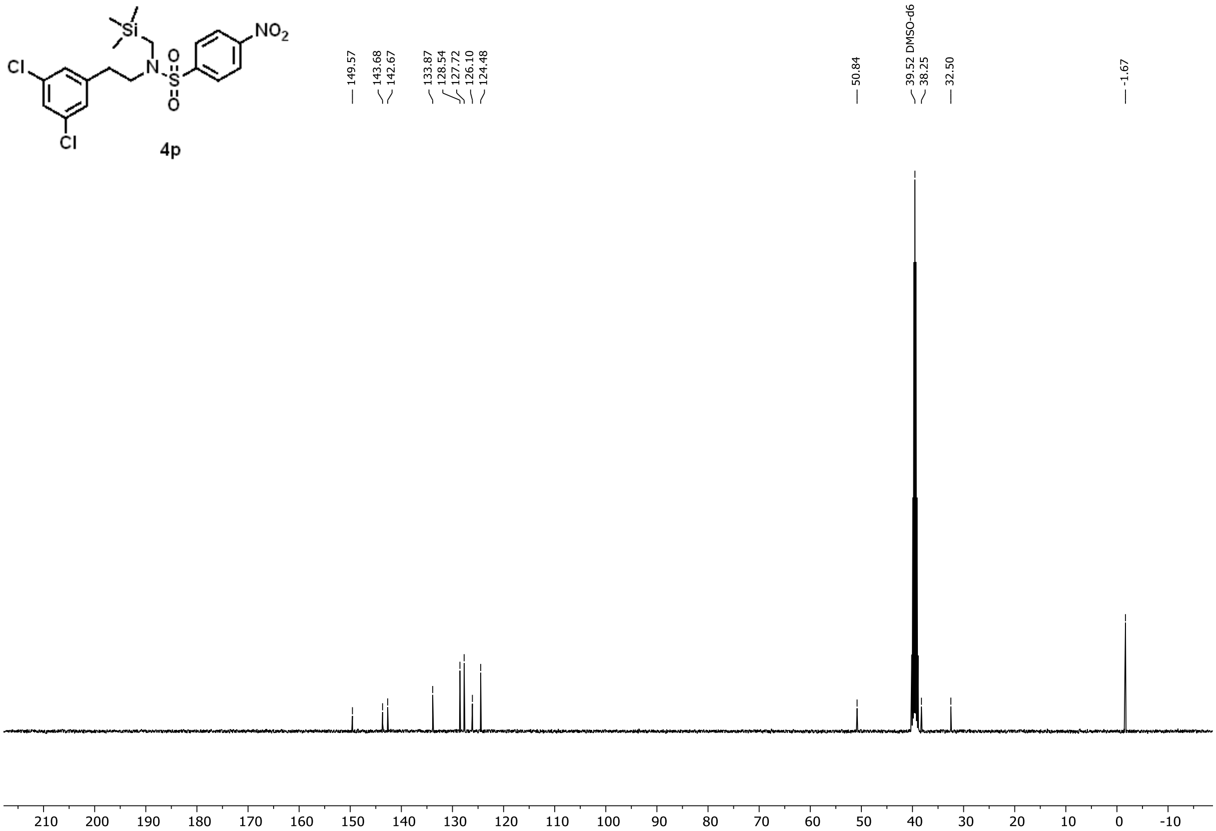

Figure **S37**: ^{13}C NMR (126 MHz, DMSO-d$_6$) of **4p**.

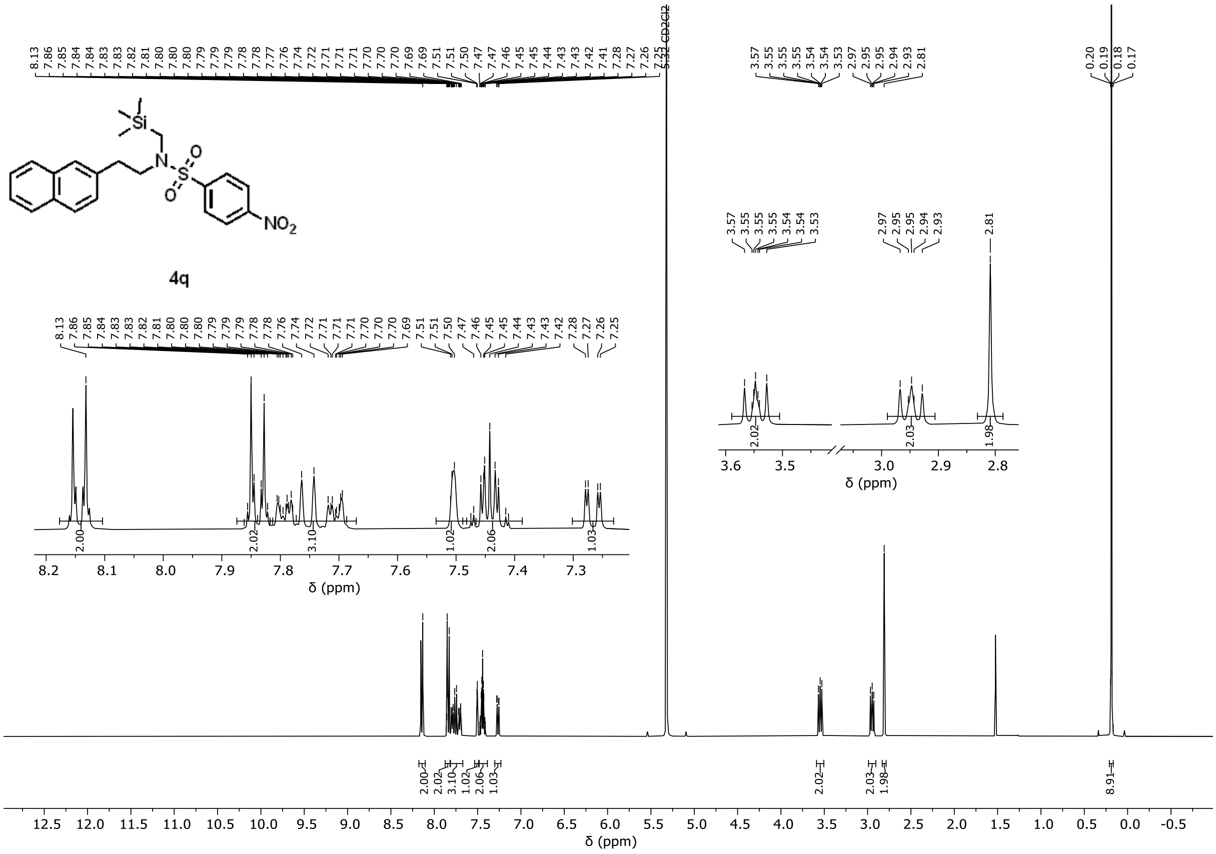

Figure **S38**: ^{1}H NMR (400 MHz, CD_2Cl_2) of **4q**.

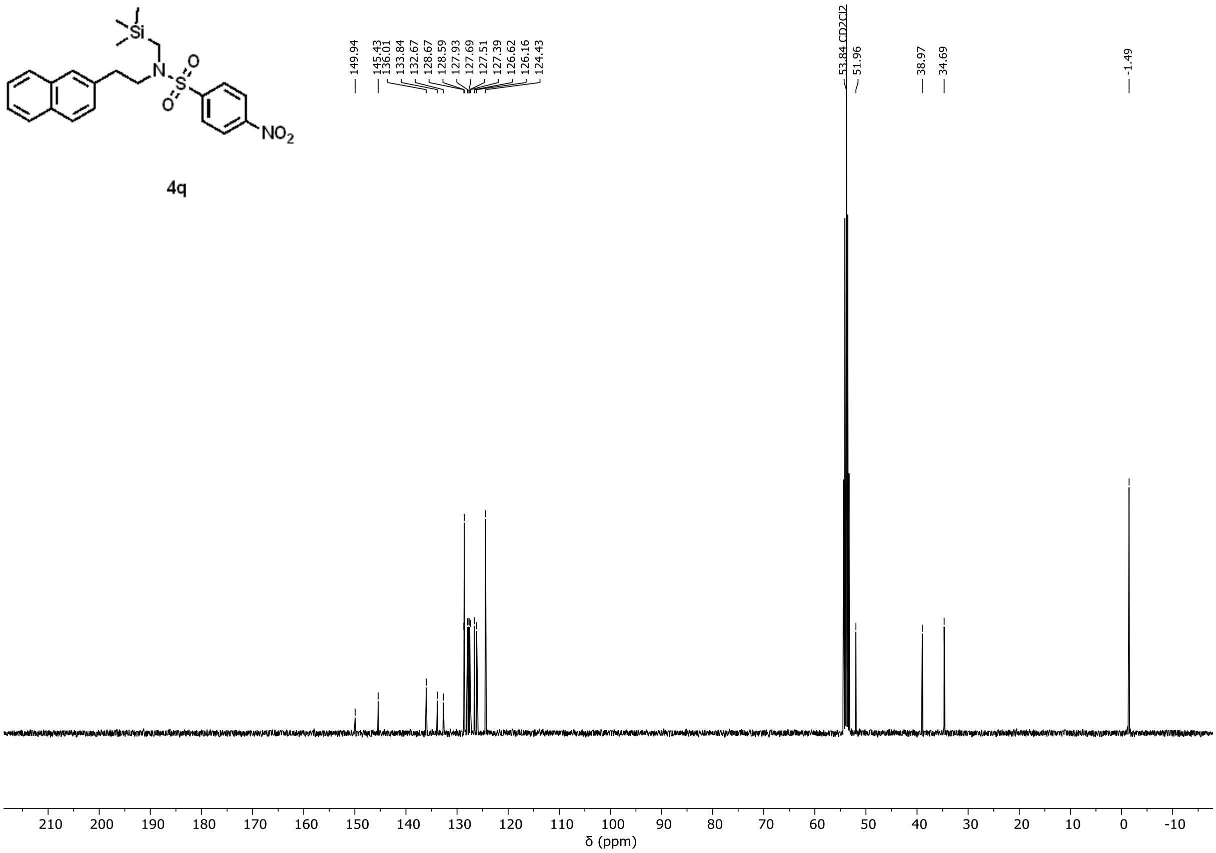

Figure **S39**: ^{13}C NMR (101 MHz, CD_2Cl_2) of **4q**.

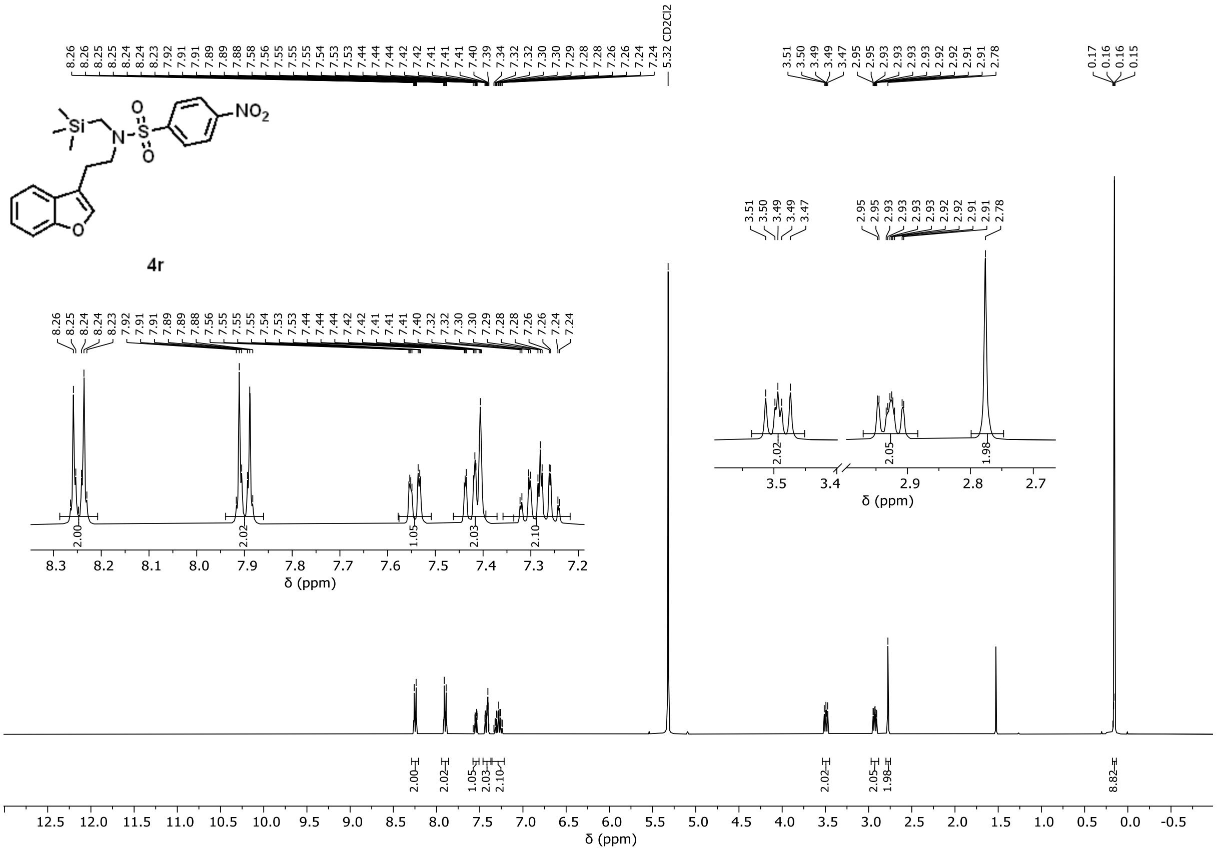

Figure **S40**: ^{1}H NMR (400 MHz, CD_2Cl_2) of **4r**.

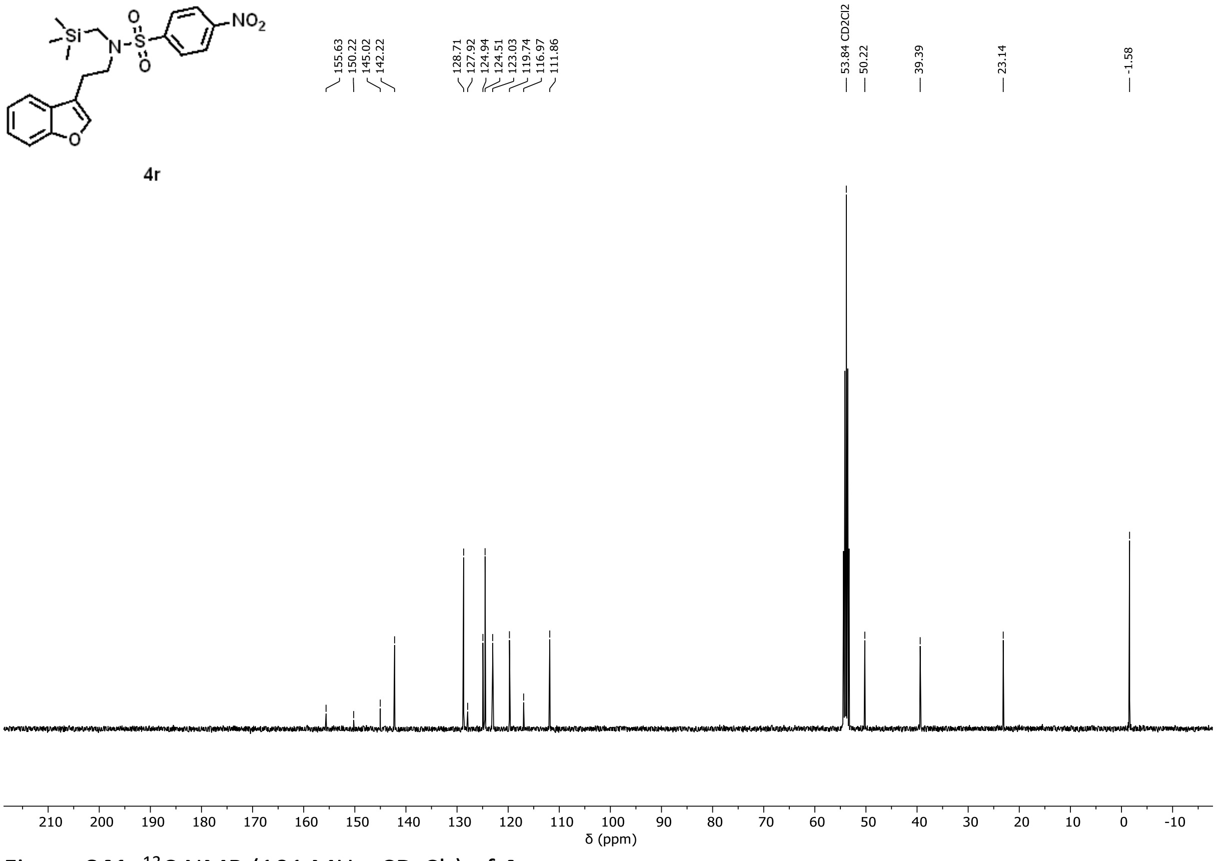

Figure **S41**: ^{13}C NMR (101 MHz, CD_2Cl_2) of **4r**.

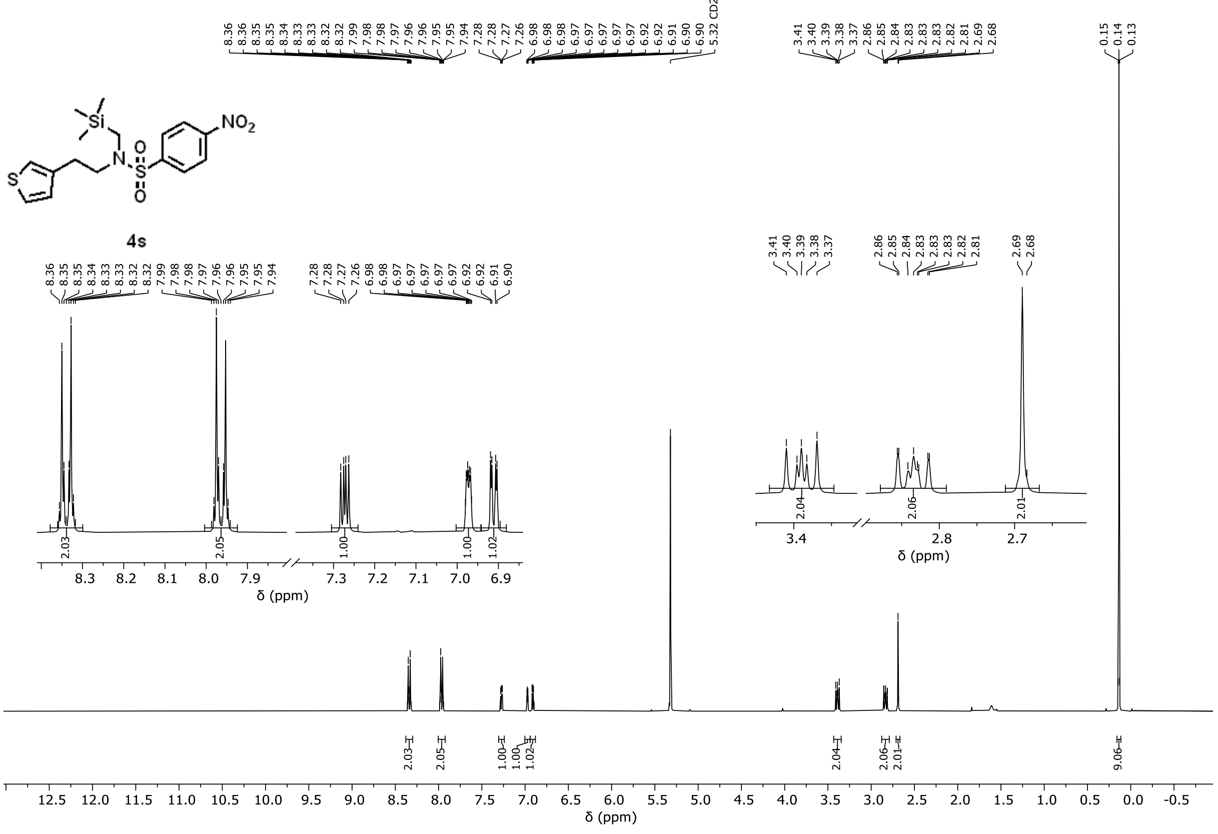

Figure **S42**: ^{1}H NMR (400 MHz, CD_2Cl_2) of **4s**.

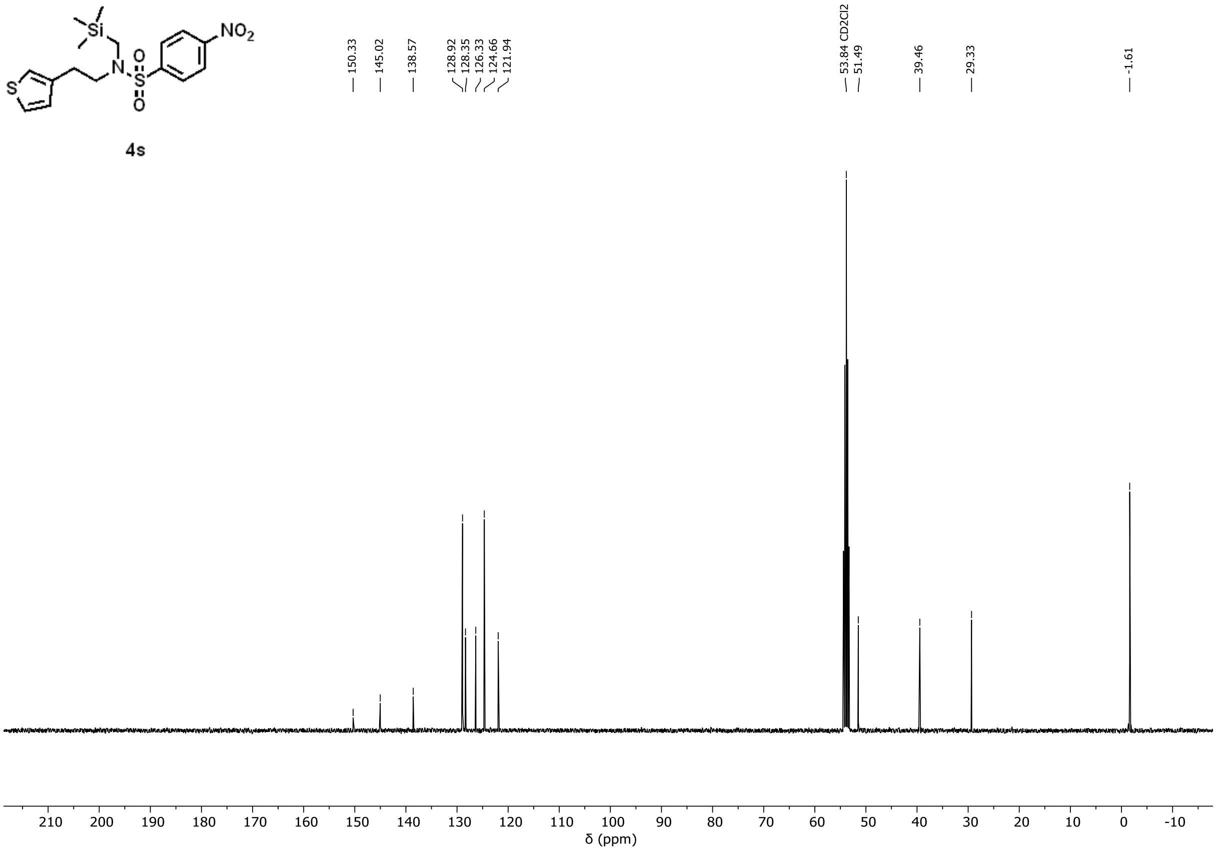

Figure **S43**: ^{13}C NMR (101 MHz, CD_2Cl_2) of **4s**.

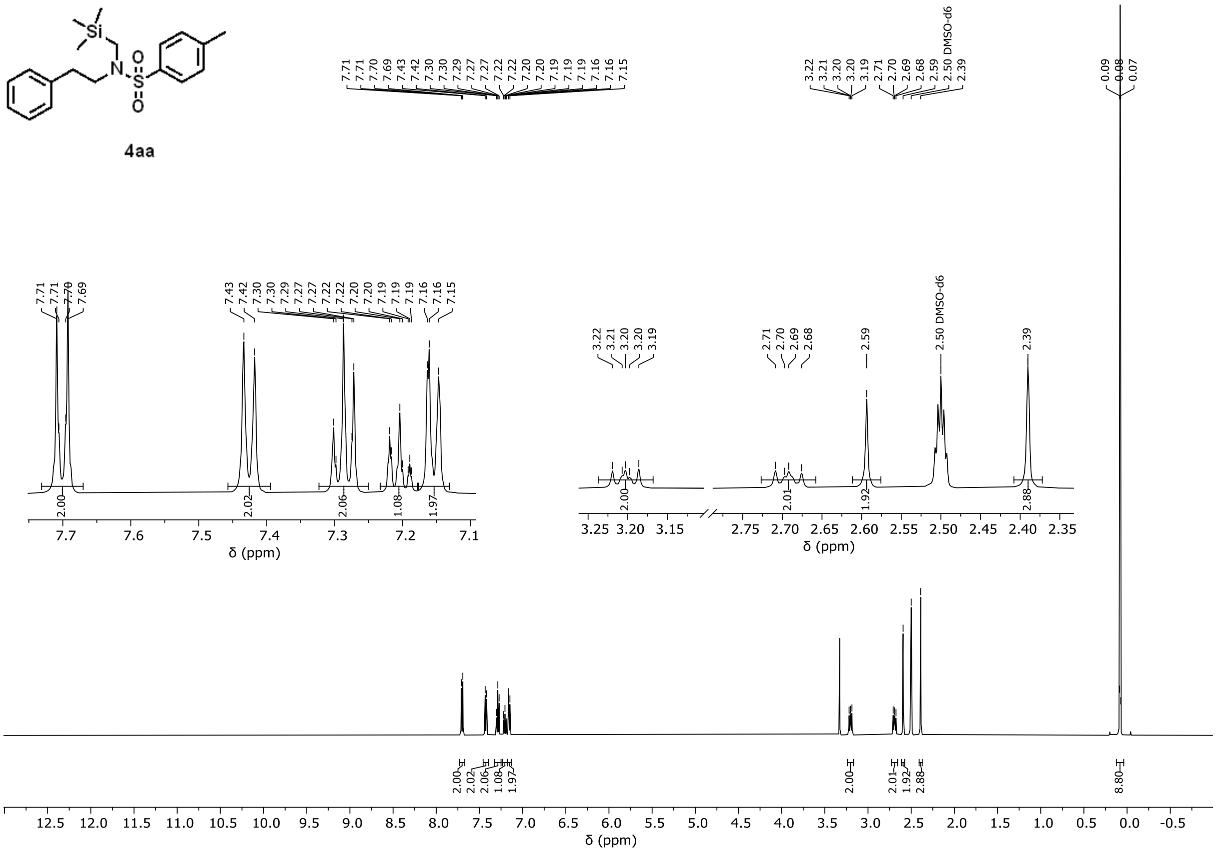

Figure **S44**: ^{1}H NMR (400 MHz, DMSO-d_6) of **4aa**.

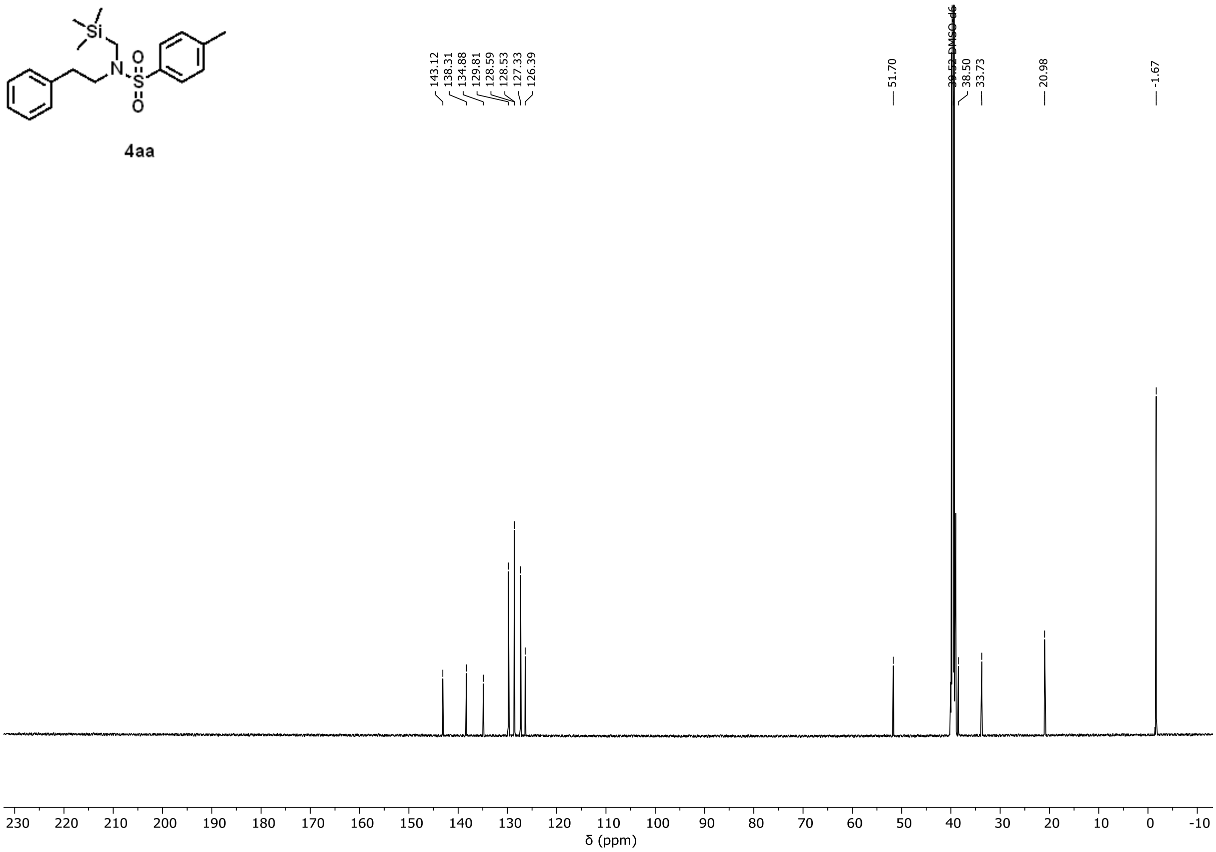

Figure **S45**: ^{13}C NMR (101 MHz, DMSO-d_6) of **4aa**.

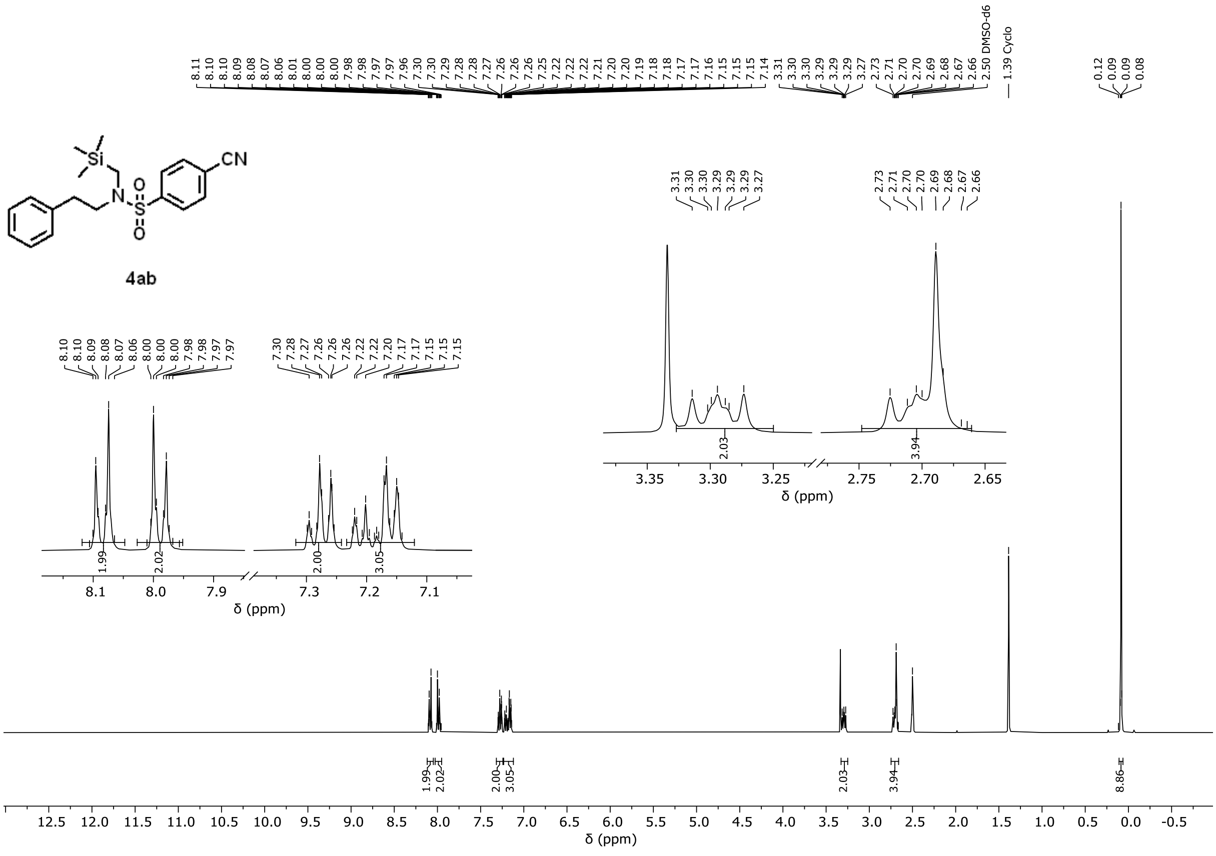

Figure **S46**: ^{1}H NMR (400 MHz, DMSO-d$_6$) of **4ab**.

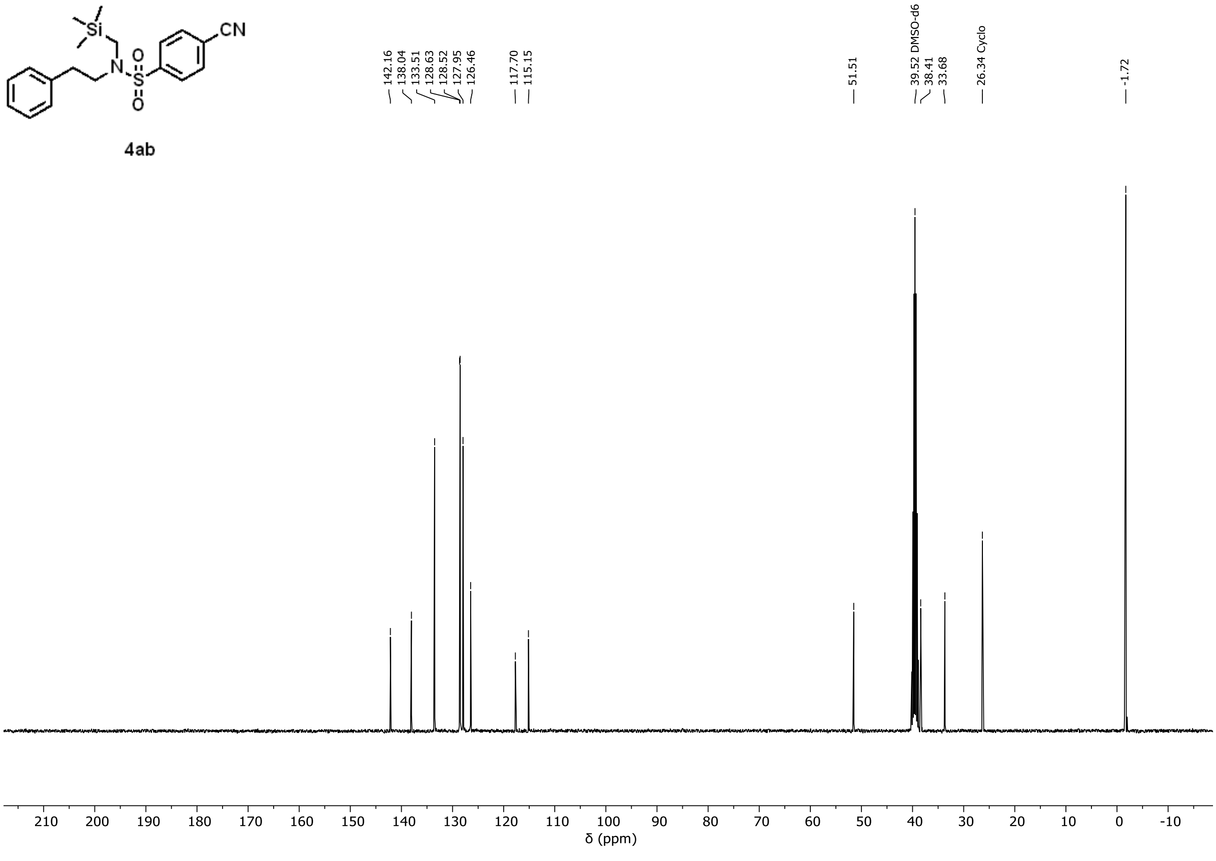

Figure **S47**: ^{13}C NMR (101 MHz, DMSO-d$_6$) of **4ab**.

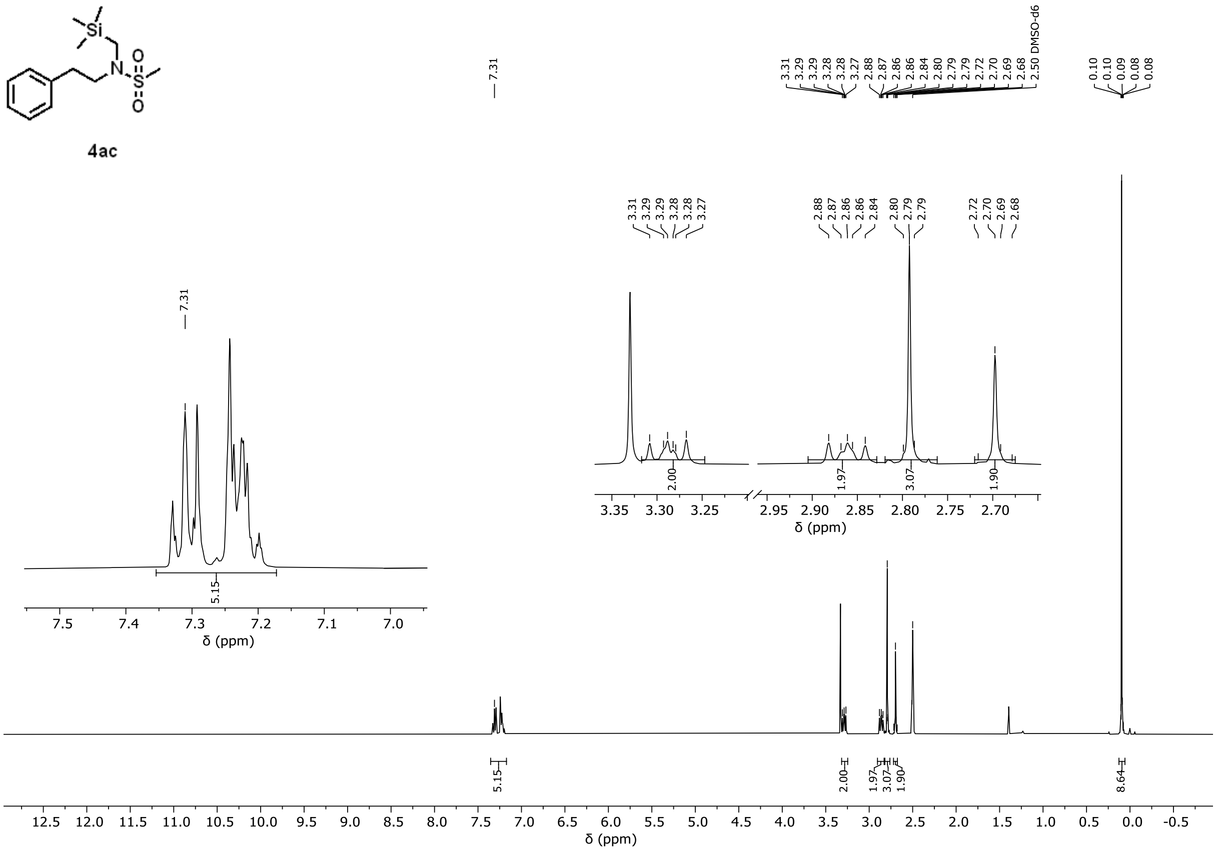

Figure **S48**: ^{1}H NMR (400 MHz, DMSO-d$_6$) of **4ac**.

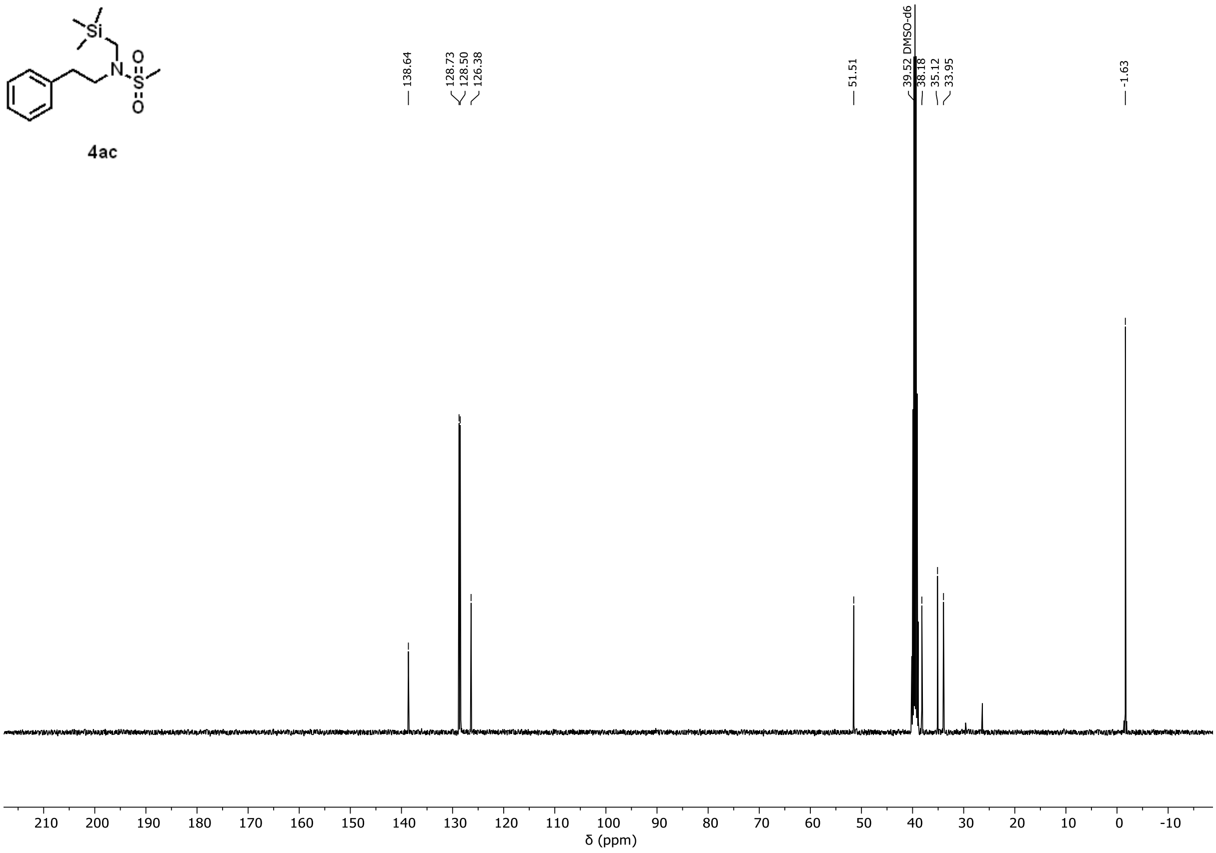

Figure **S49**: ^{13}C NMR (101 MHz, DMSO-d$_6$) of **4ac**.

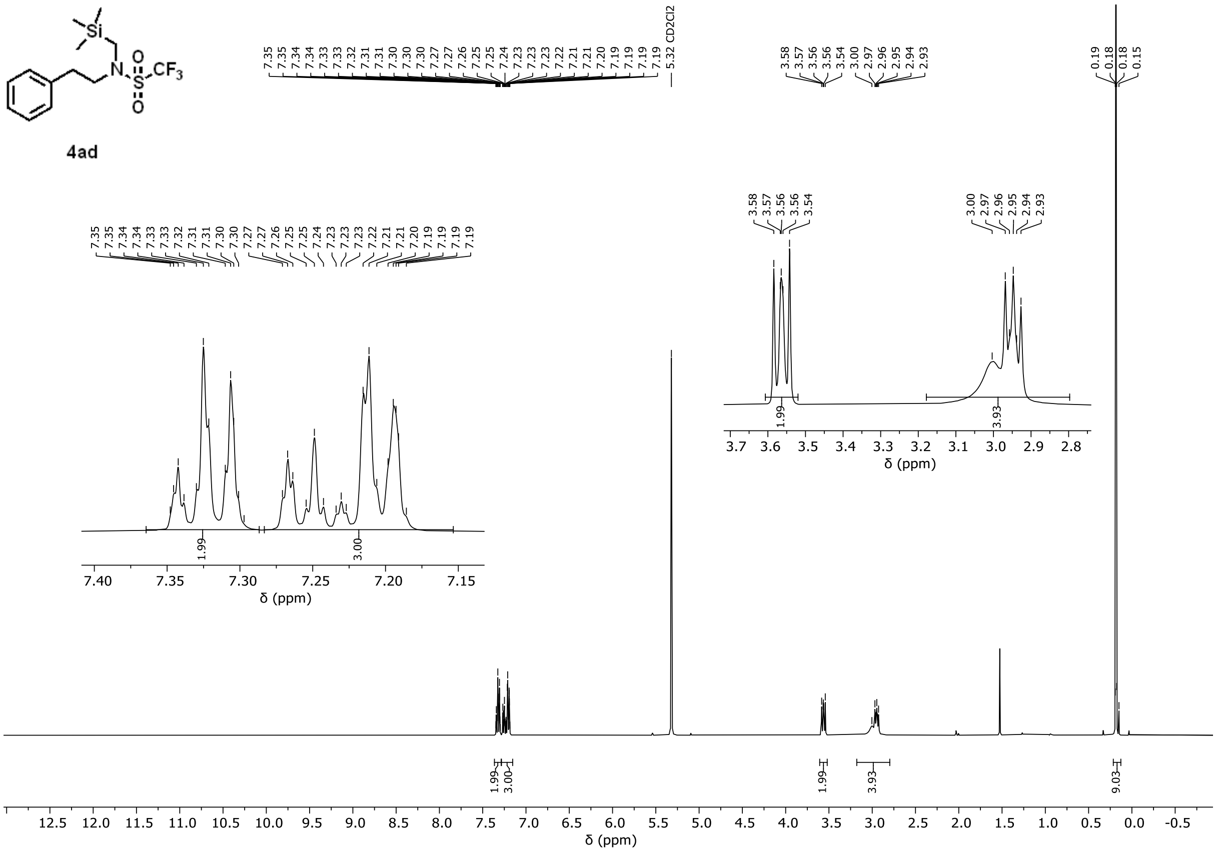

Figure **S50**: ^{1}H NMR (400 MHz, CD_2Cl_2) of **4ad**.

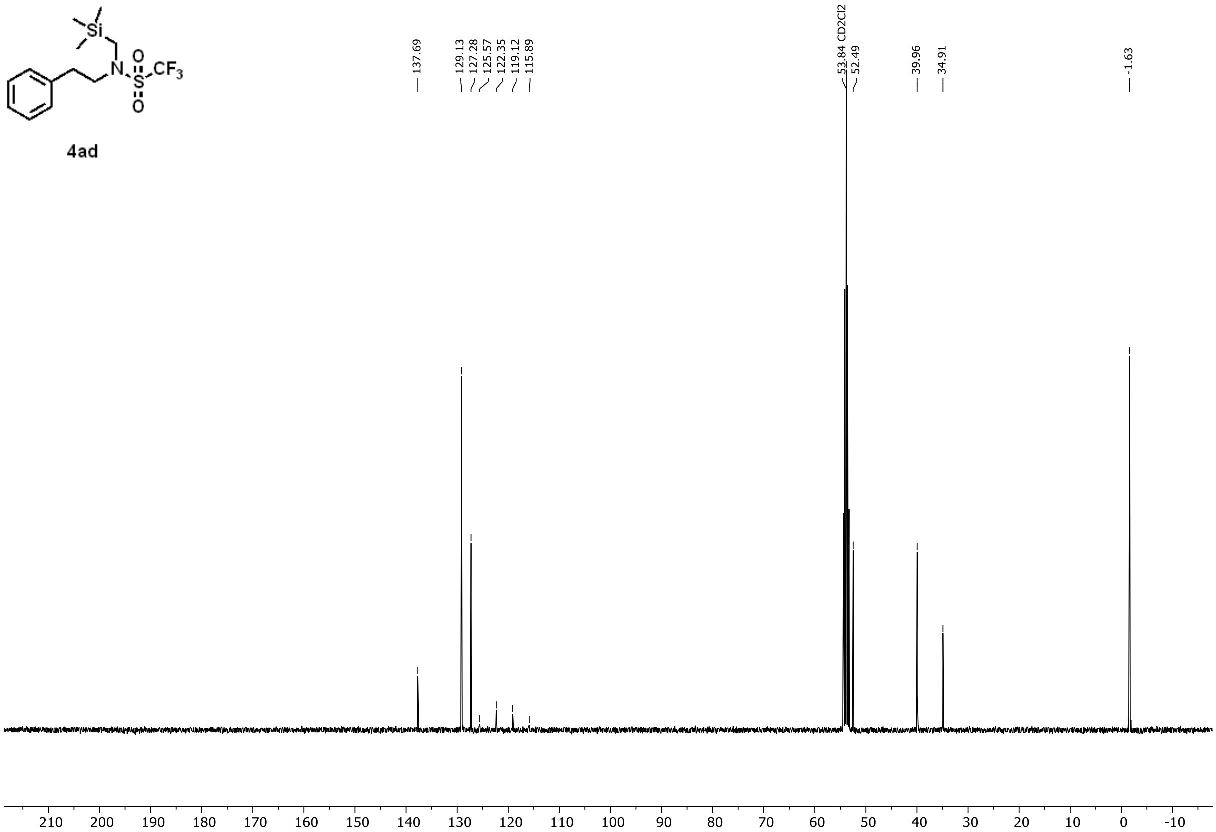

Figure **S51**: ^{13}C NMR (101 MHz, CD_2Cl_2) of **4ad**.

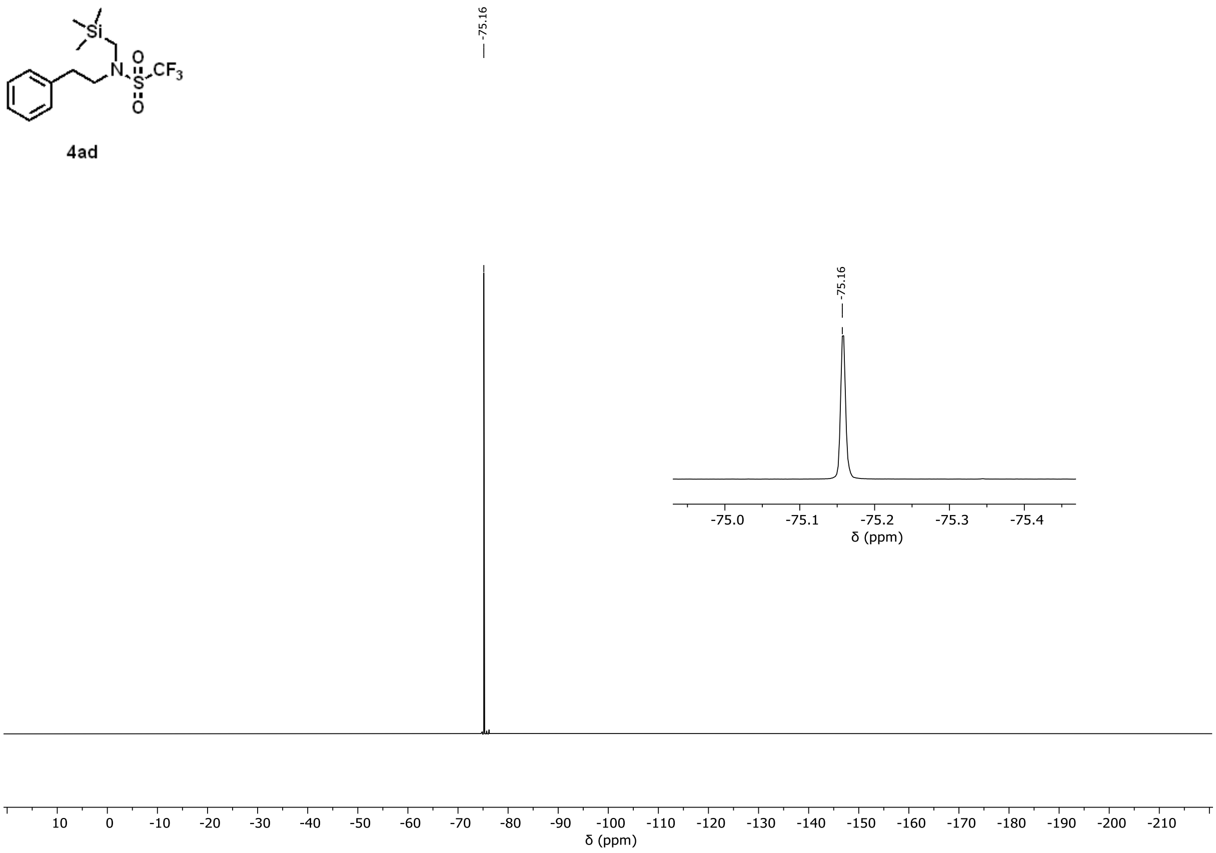

Figure **S52**: ^{19}F NMR (376 MHz, CD_2Cl_2) of **4ad**.

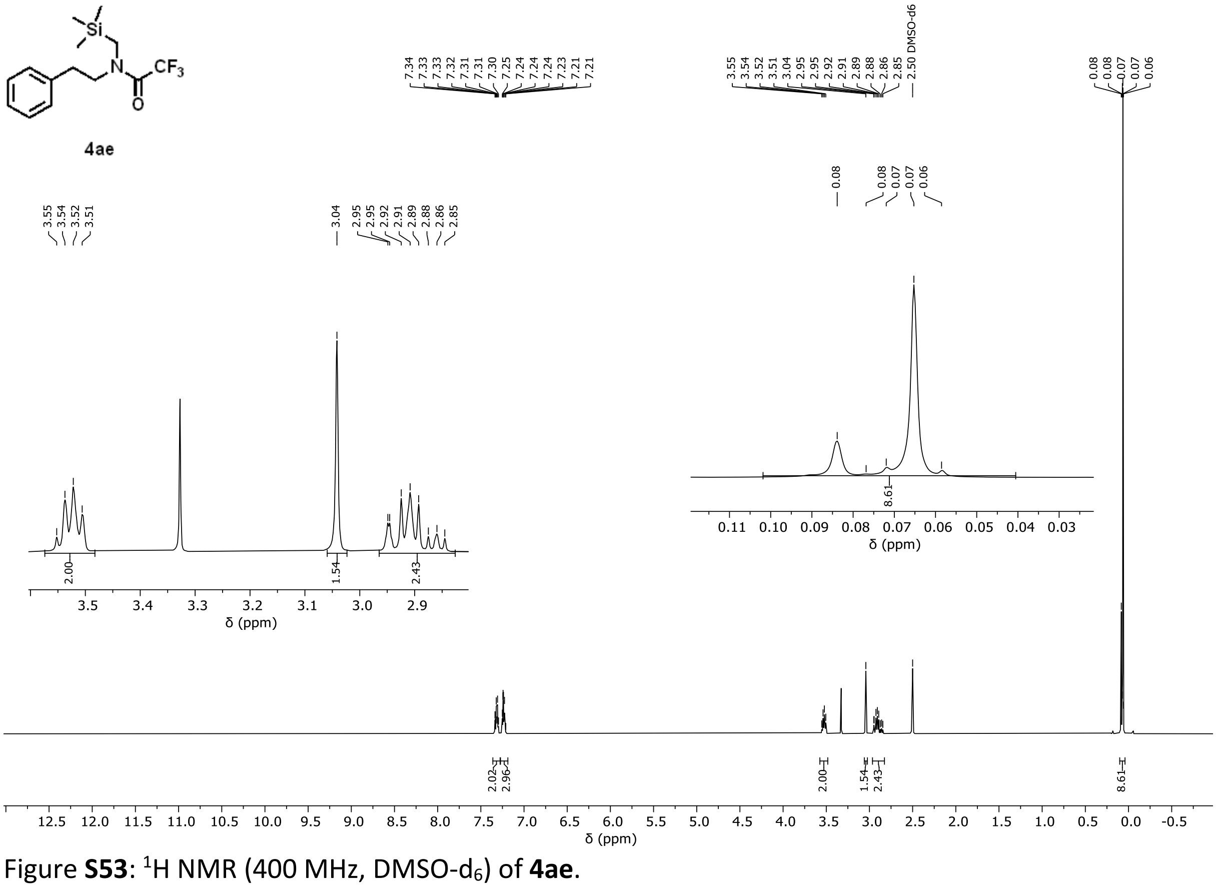

Figure **S53**: ^{1}H NMR (400 MHz, DMSO-d_6) of **4ae**.

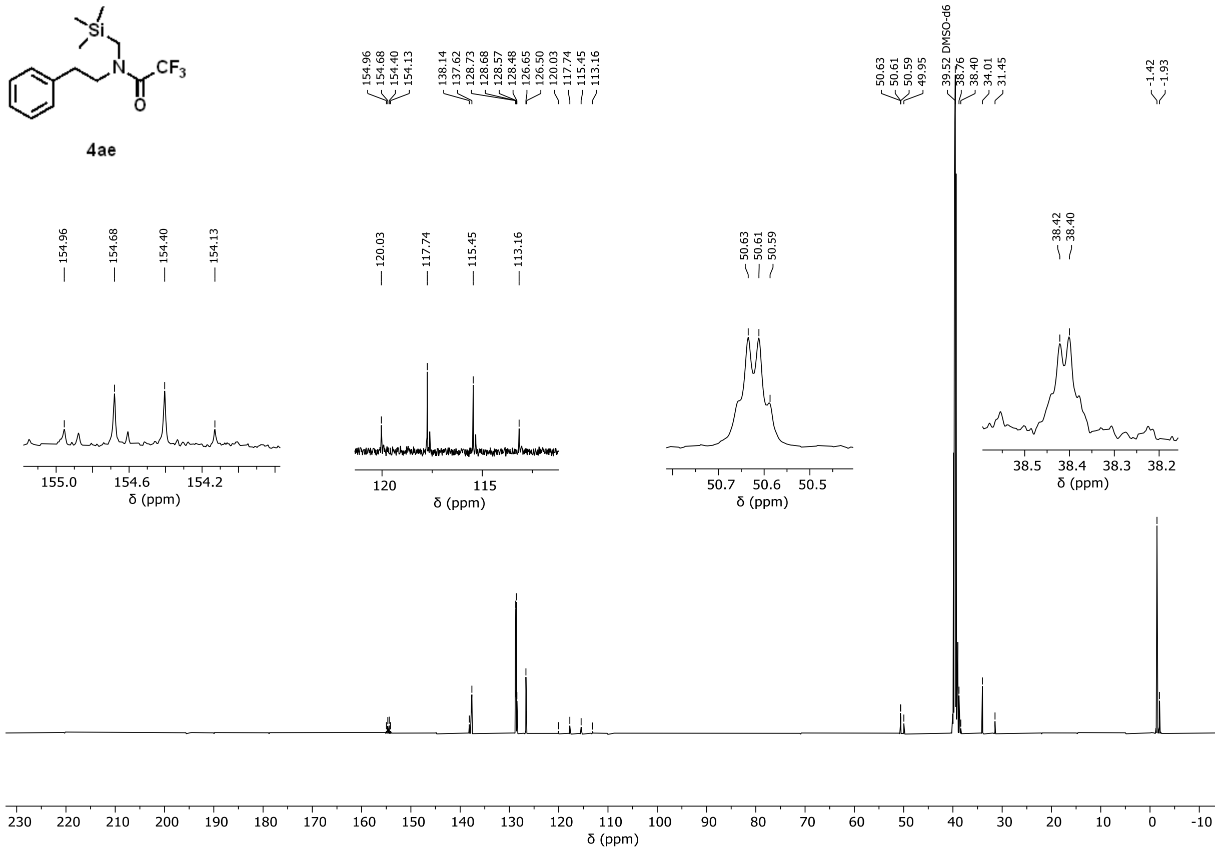

Figure **S54**: ^{13}C NMR (101 MHz, DMSO-d_6) of **4ae**.

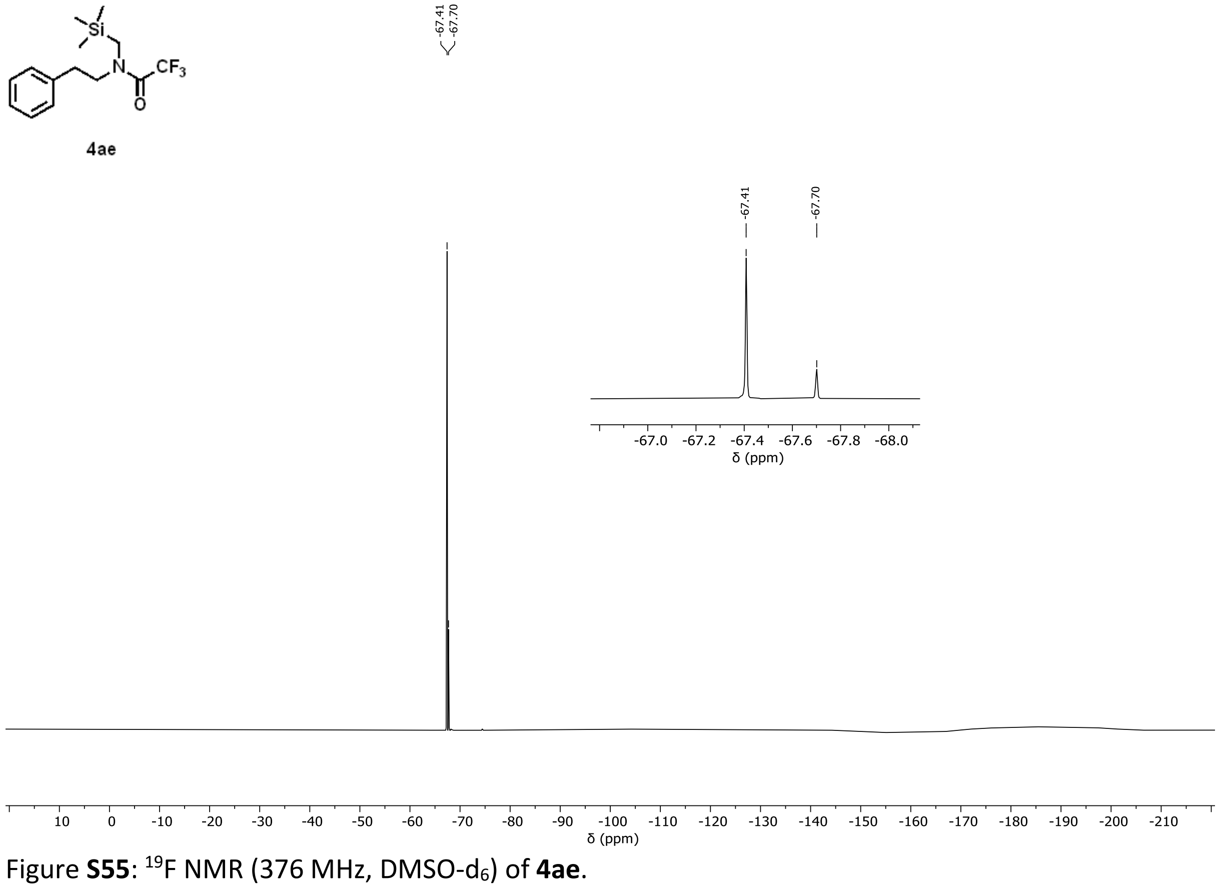

Figure **S55**: ^{19}F NMR (376 MHz, DMSO-d_6) of **4ae**.

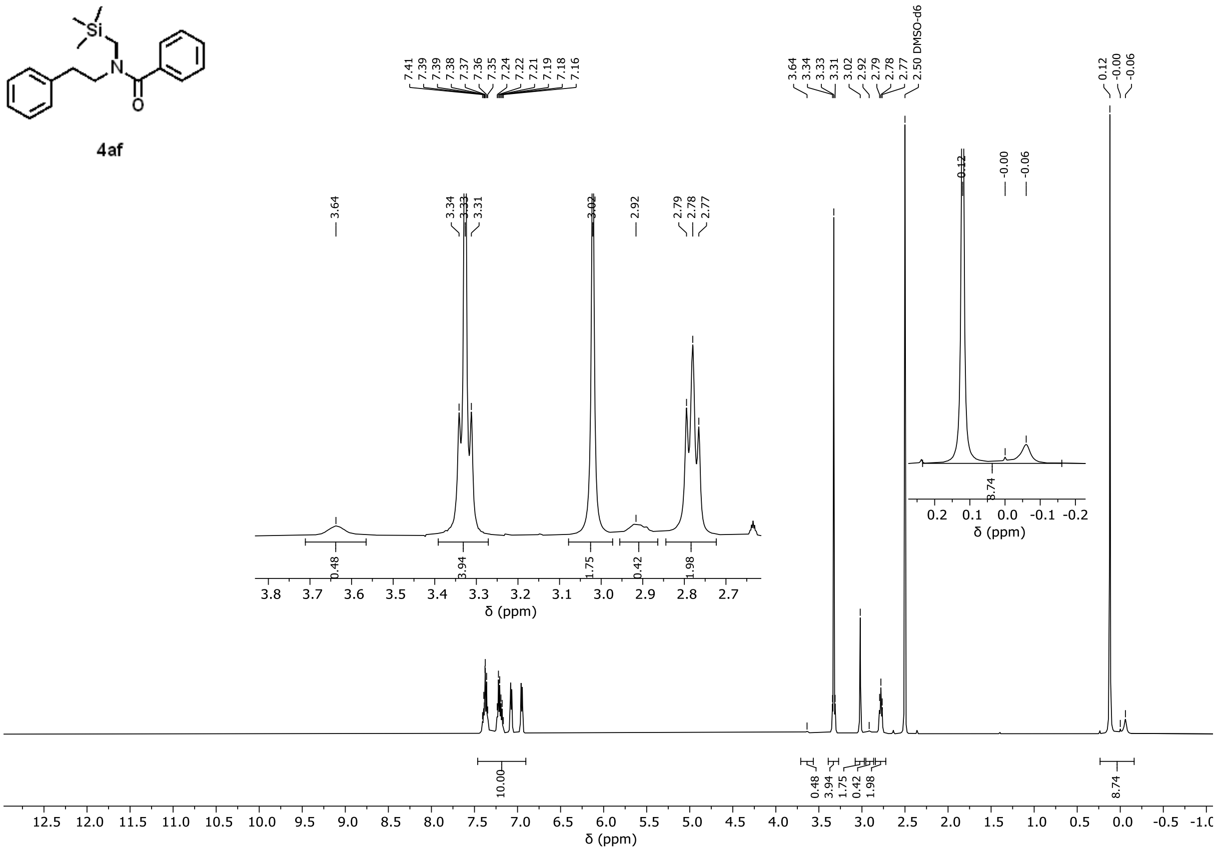

Figure **S56**: ^{1}H NMR (400 MHz, DMSO-d_6) of **4af**.

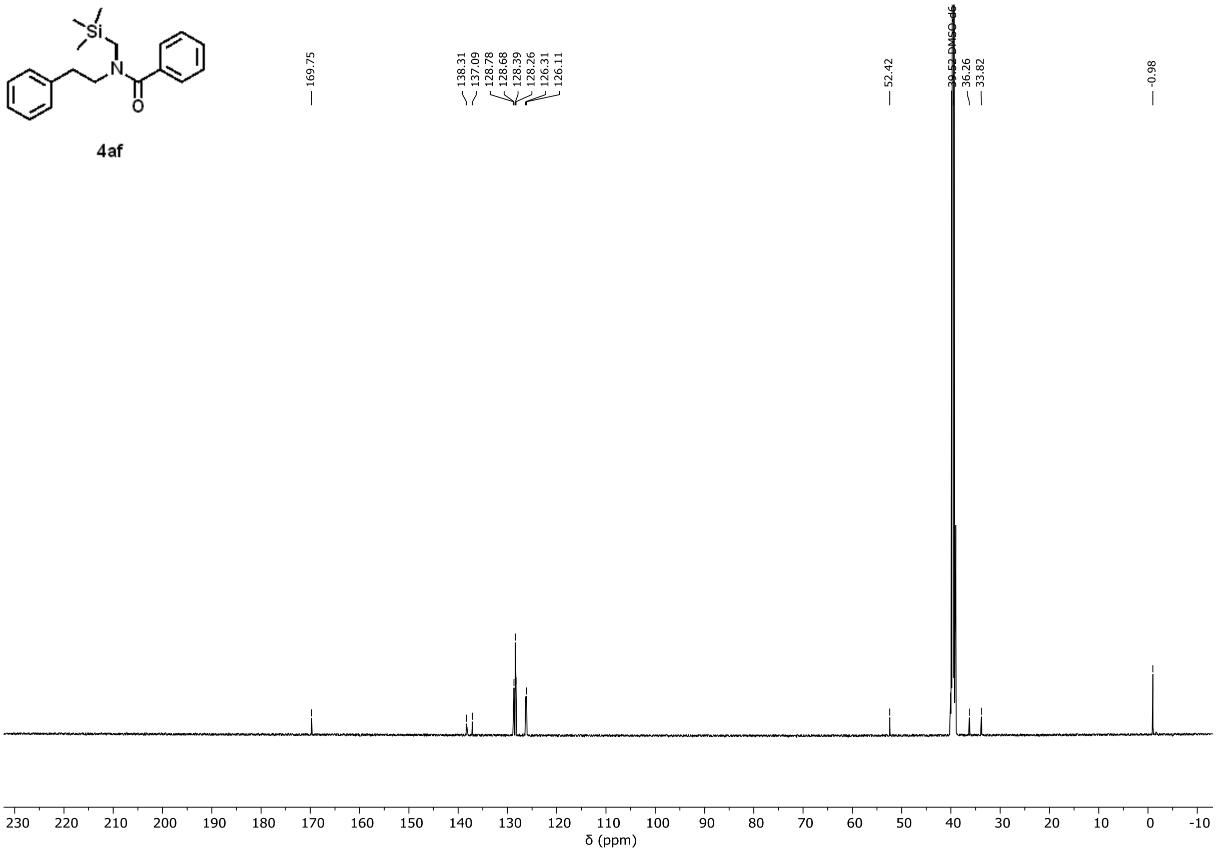

Figure **S57**: ^{13}C NMR (101 MHz, DMSO-d_6) of **4af**.

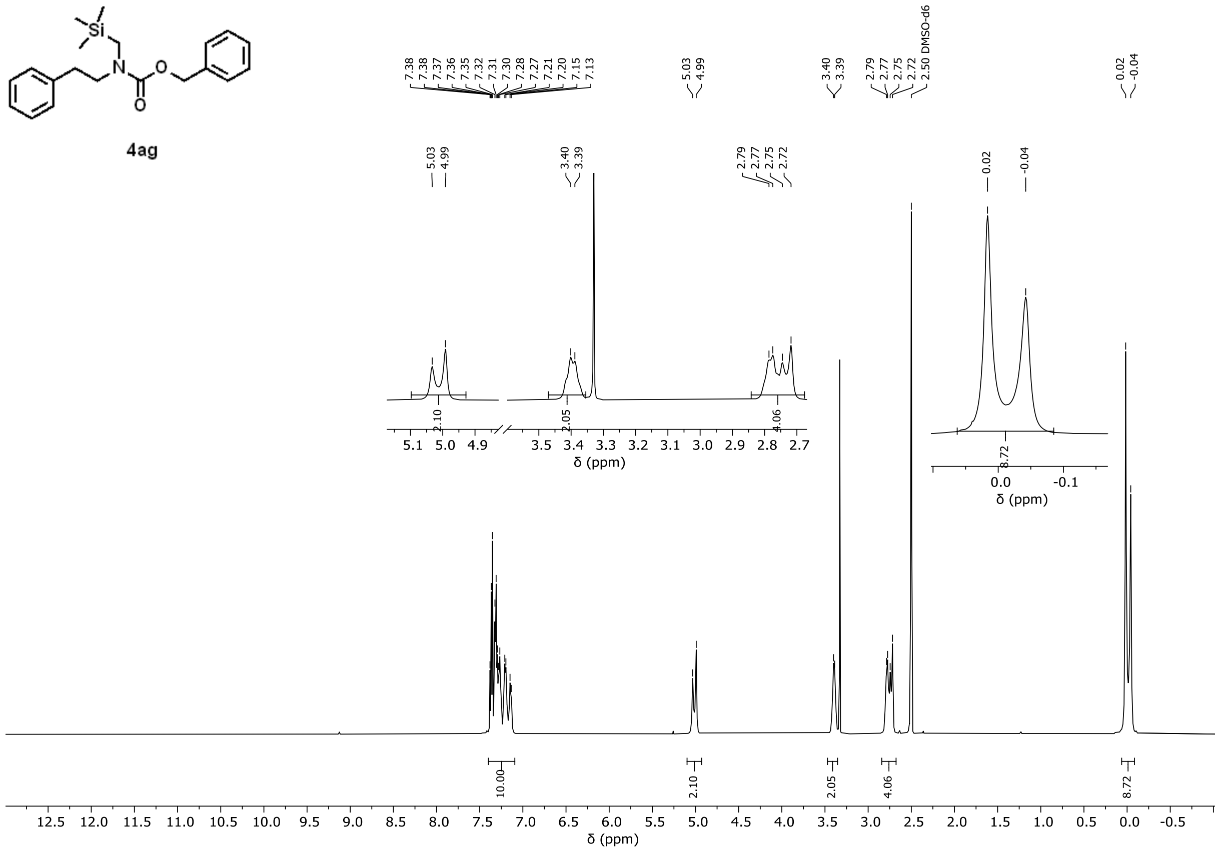

Figure **S58**: ^{1}H NMR (400 MHz, DMSO-d$_6$) of **4ag**.

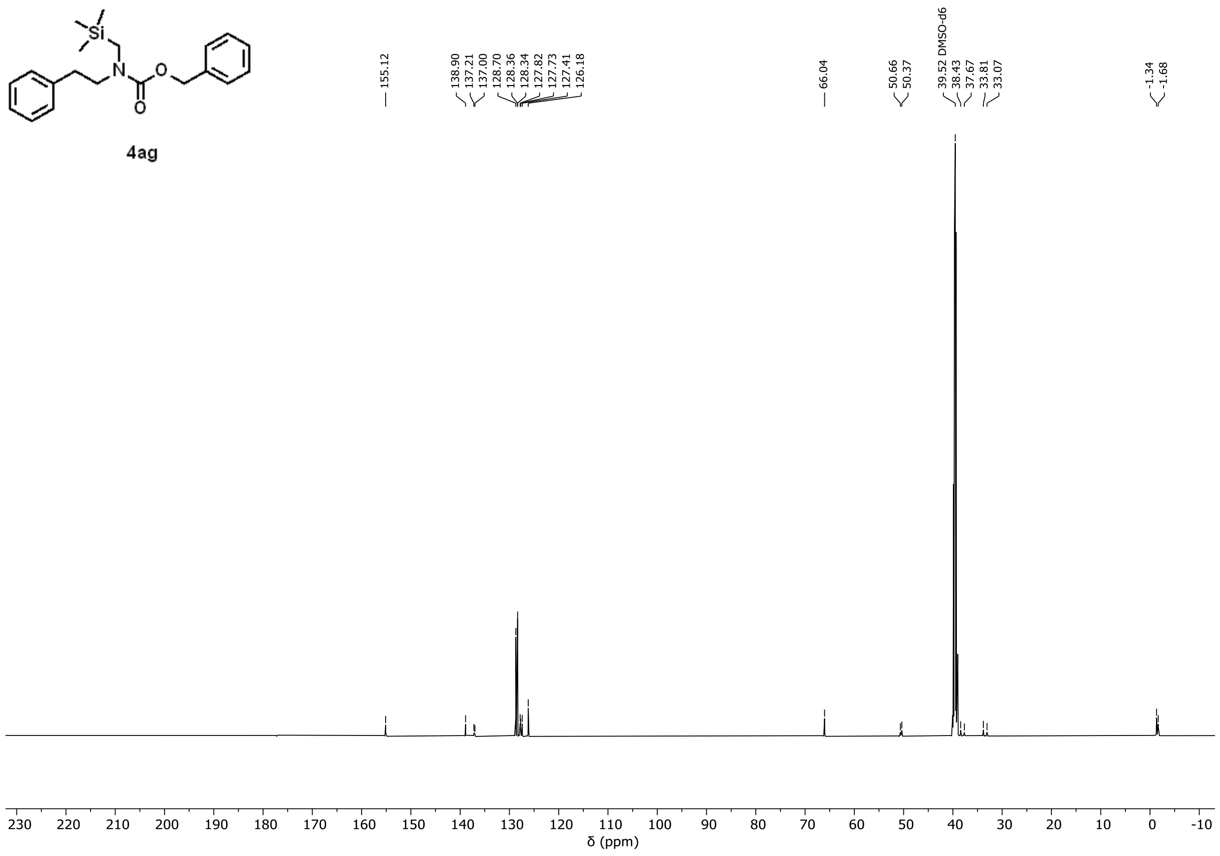

Figure **S59**: ^{13}C NMR (101 MHz, DMSO-d$_6$) of **4ag**.

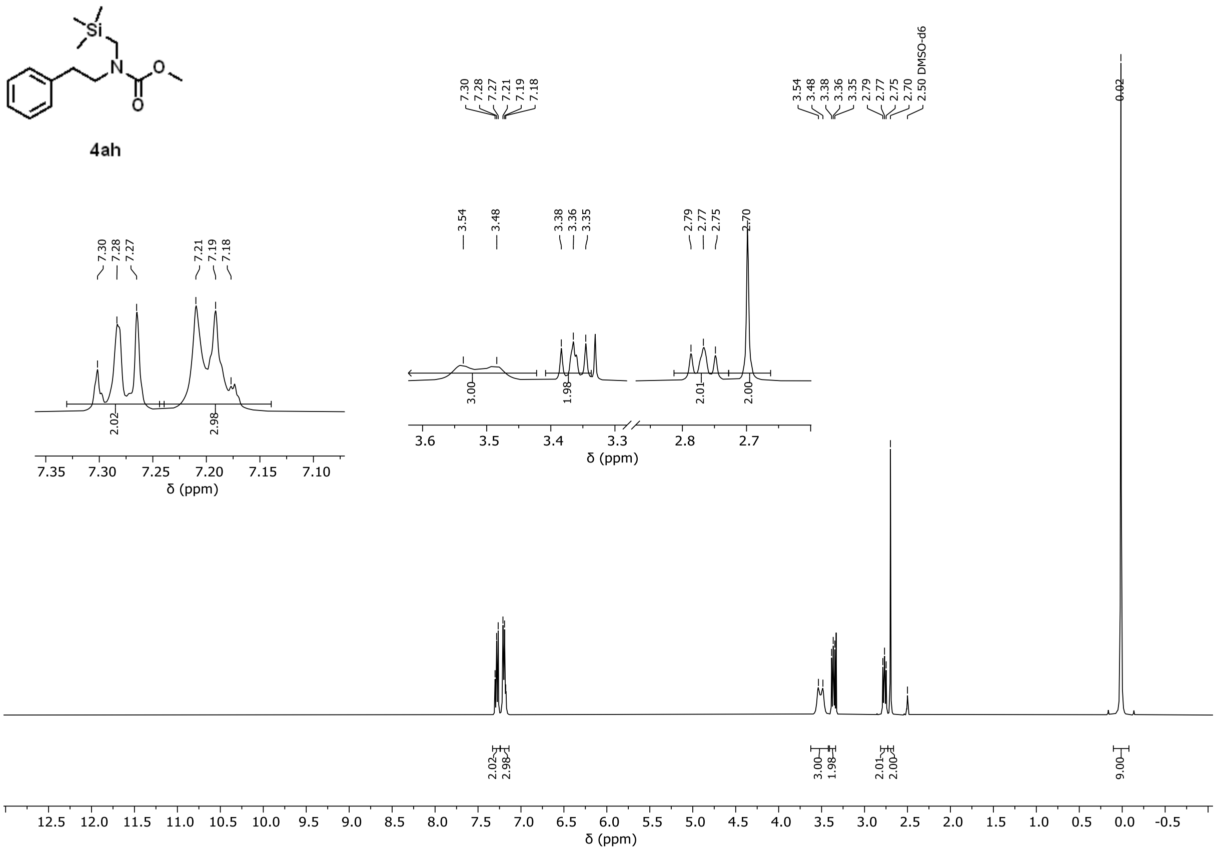

Figure **S60**: ^{1}H NMR (400 MHz, DMSO-d_6) of **4ah**.

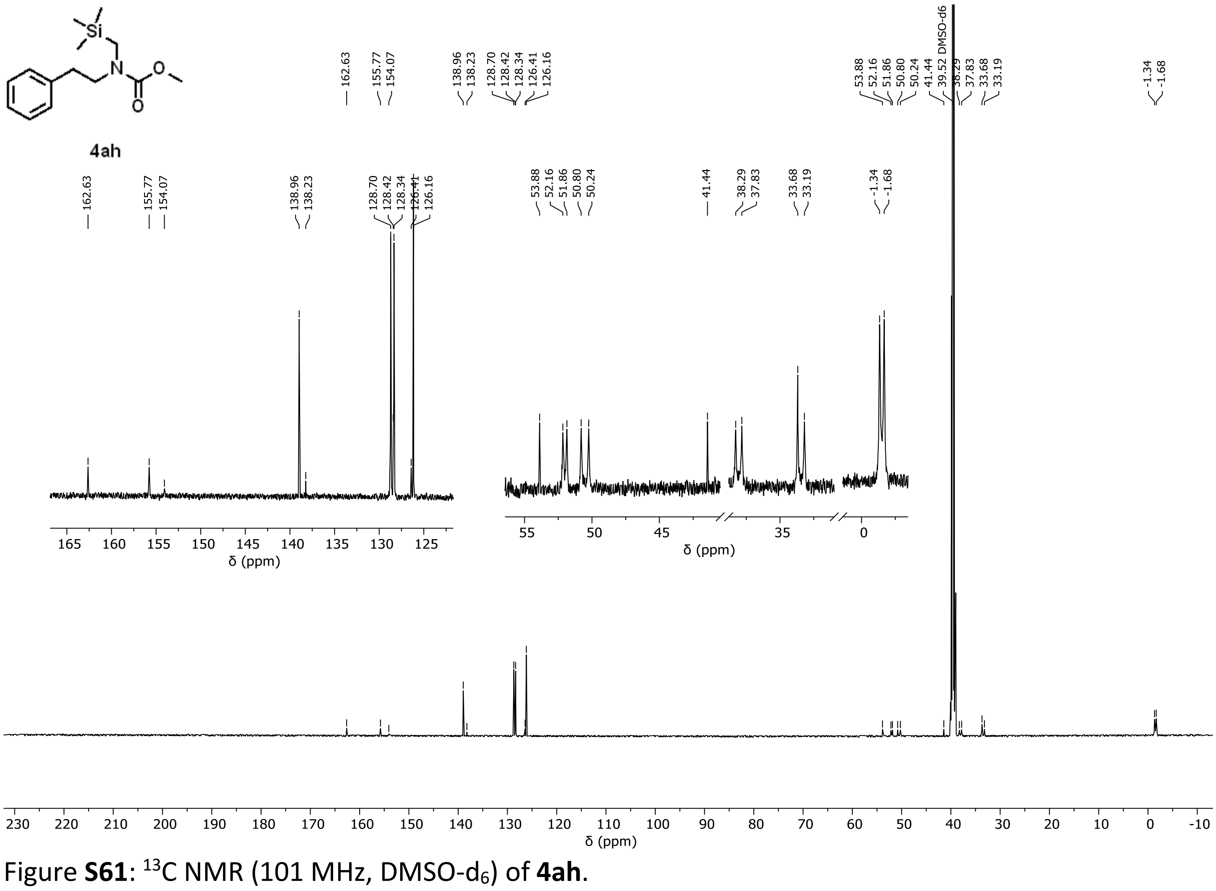

Figure **S61**: ^{13}C NMR (101 MHz, DMSO-d_6) of **4ah**.

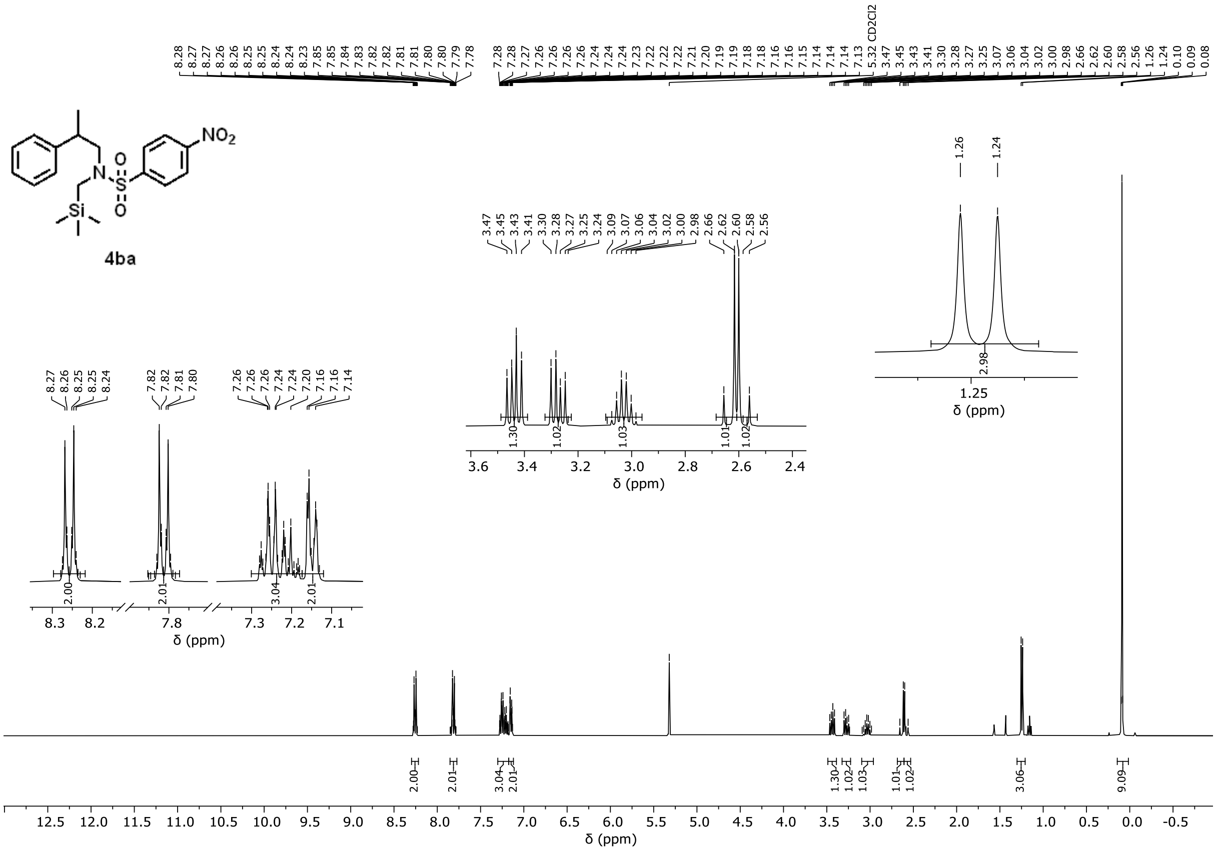

Figure **S62**: ^{1}H NMR (400 MHz, CD_2Cl_2) of **4ba**.

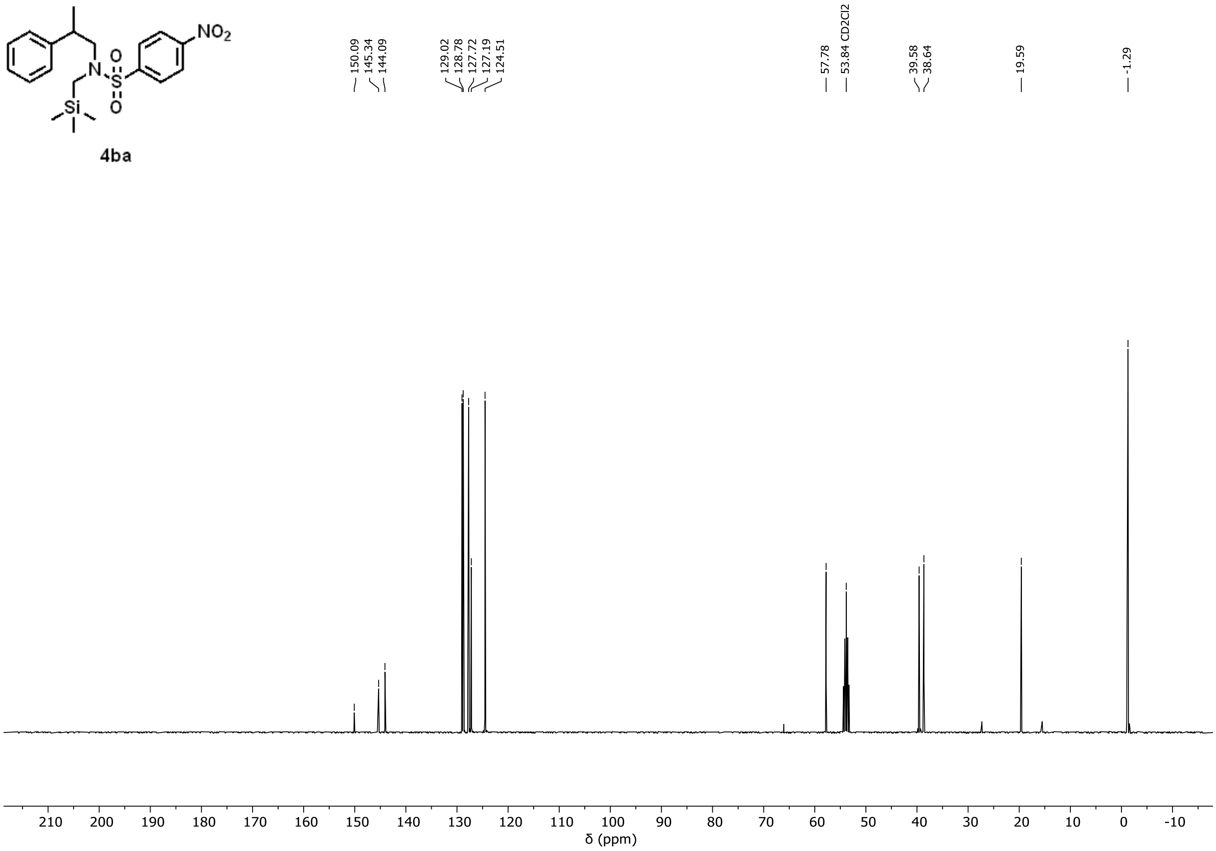

Figure **S63**: ^{13}C NMR (101 MHz, CD_2Cl_2) of **4ba**.

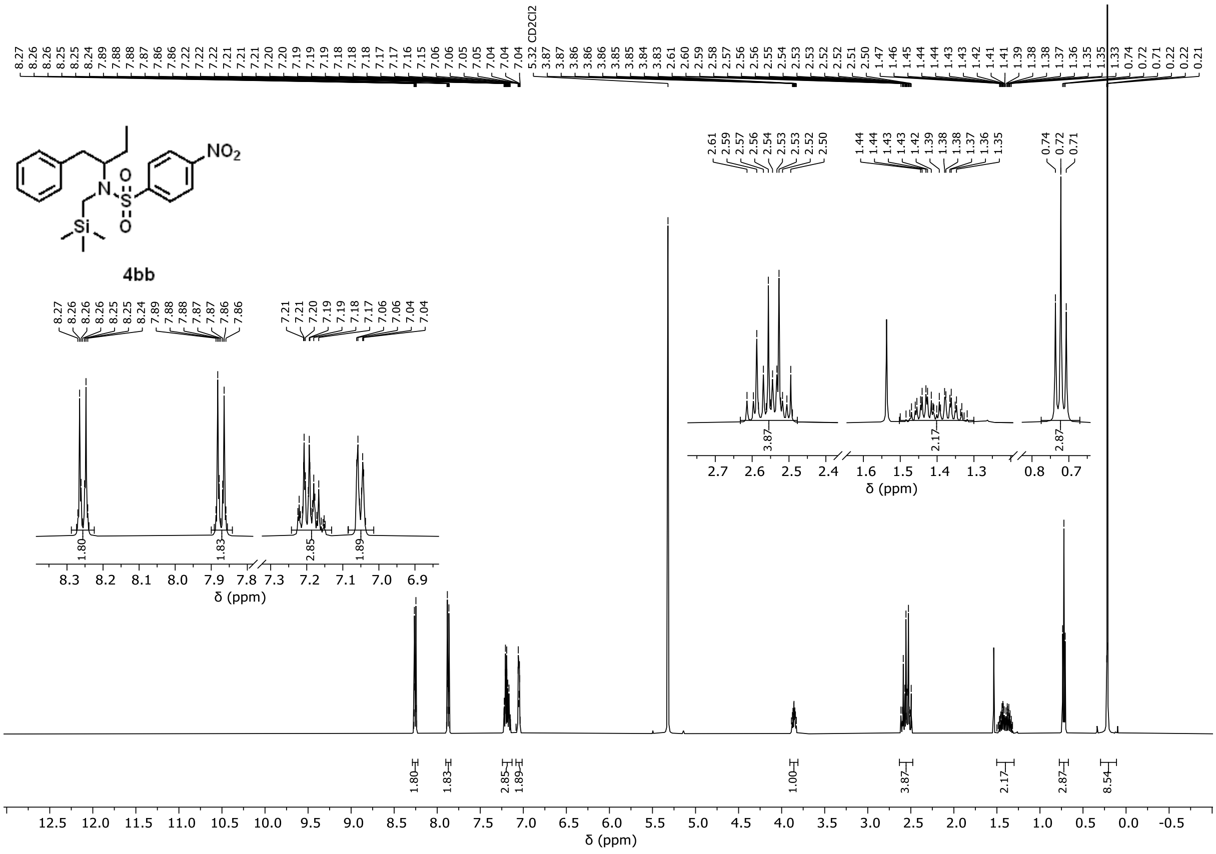

Figure **S64**: ^{1}H NMR (400 MHz, CD_2Cl_2) of **4bb**.

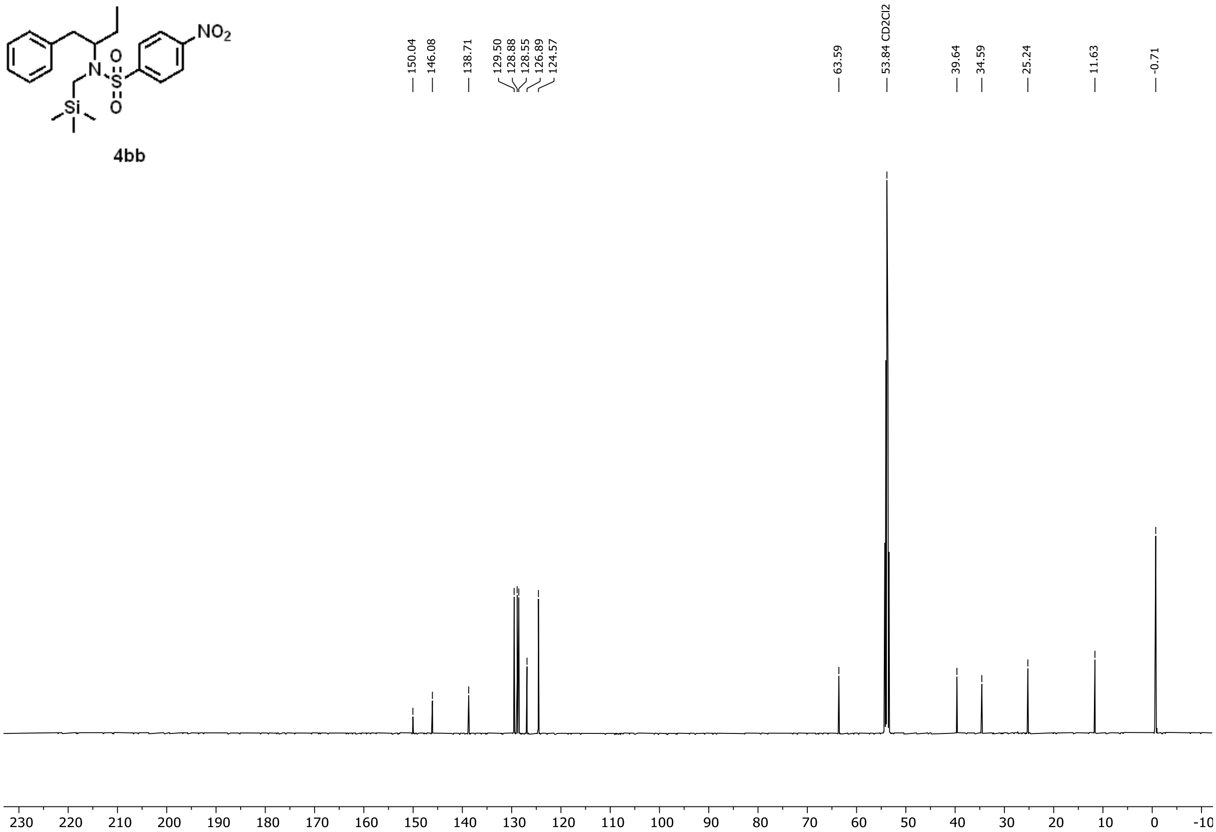

Figure **S65**: ^{13}C NMR (101 MHz, CD_2Cl_2) of **4bb**.

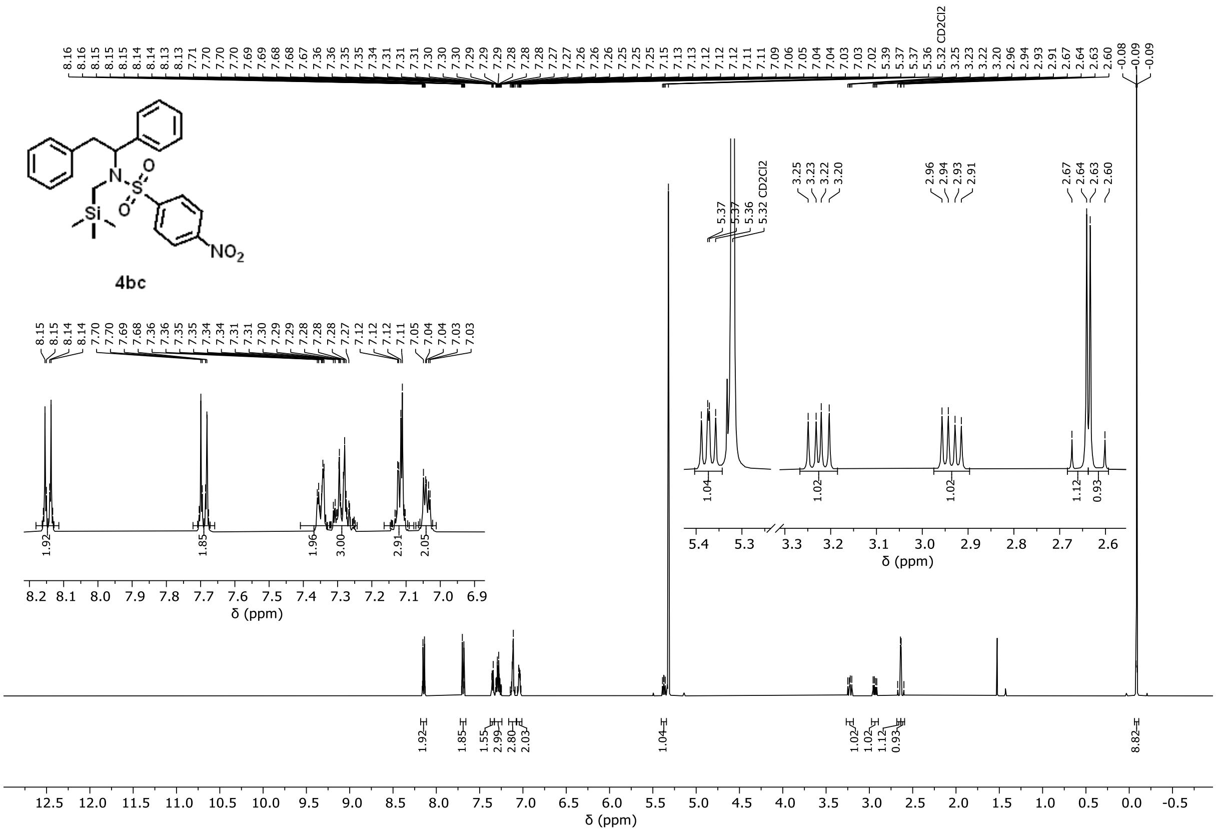

Figure **S66**: ^{1}H NMR (400 MHz, CD_2Cl_2) of **4bc**.

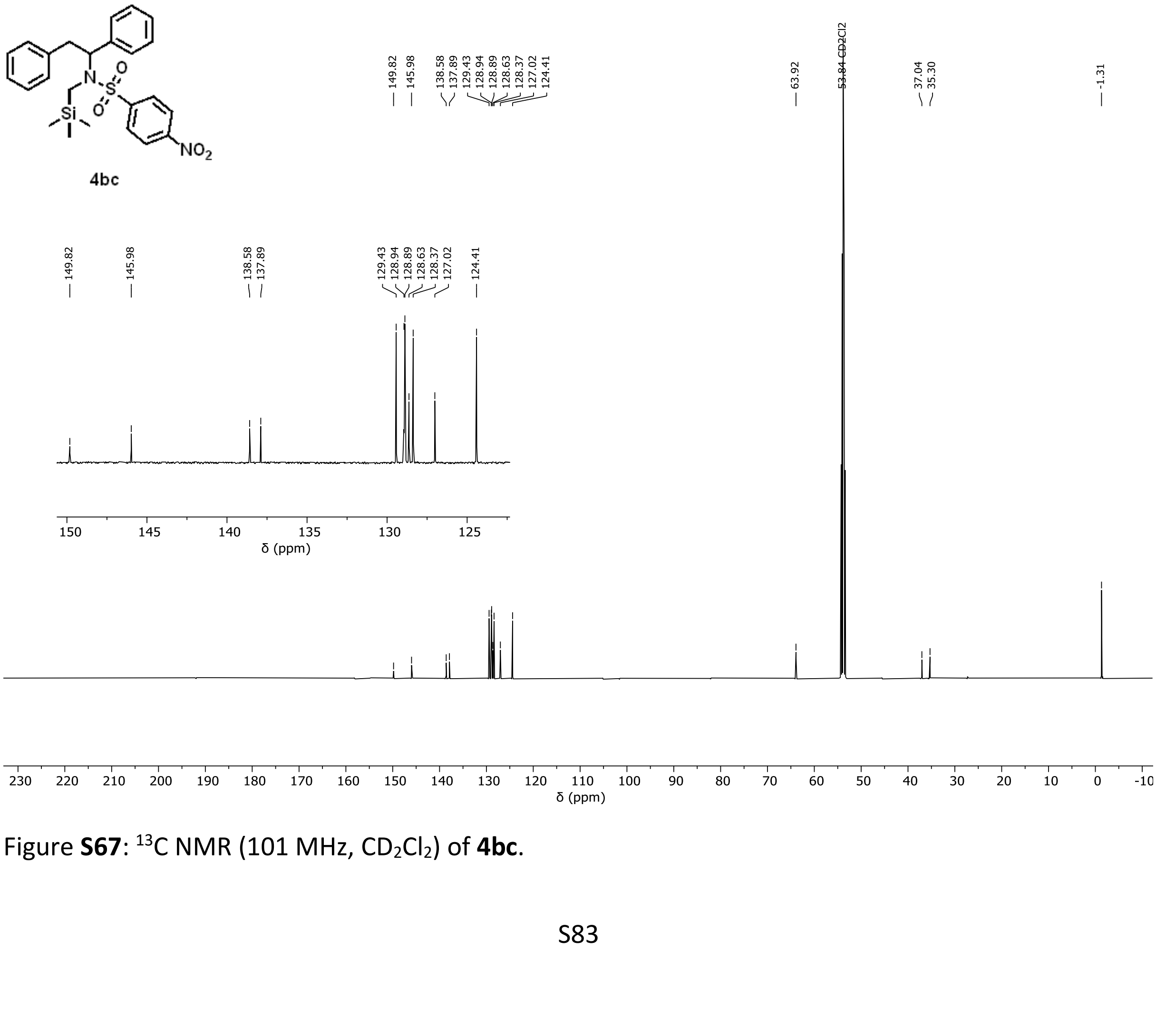

Figure **S67**: ^{13}C NMR (101 MHz, CD_2Cl_2) of **4bc**.

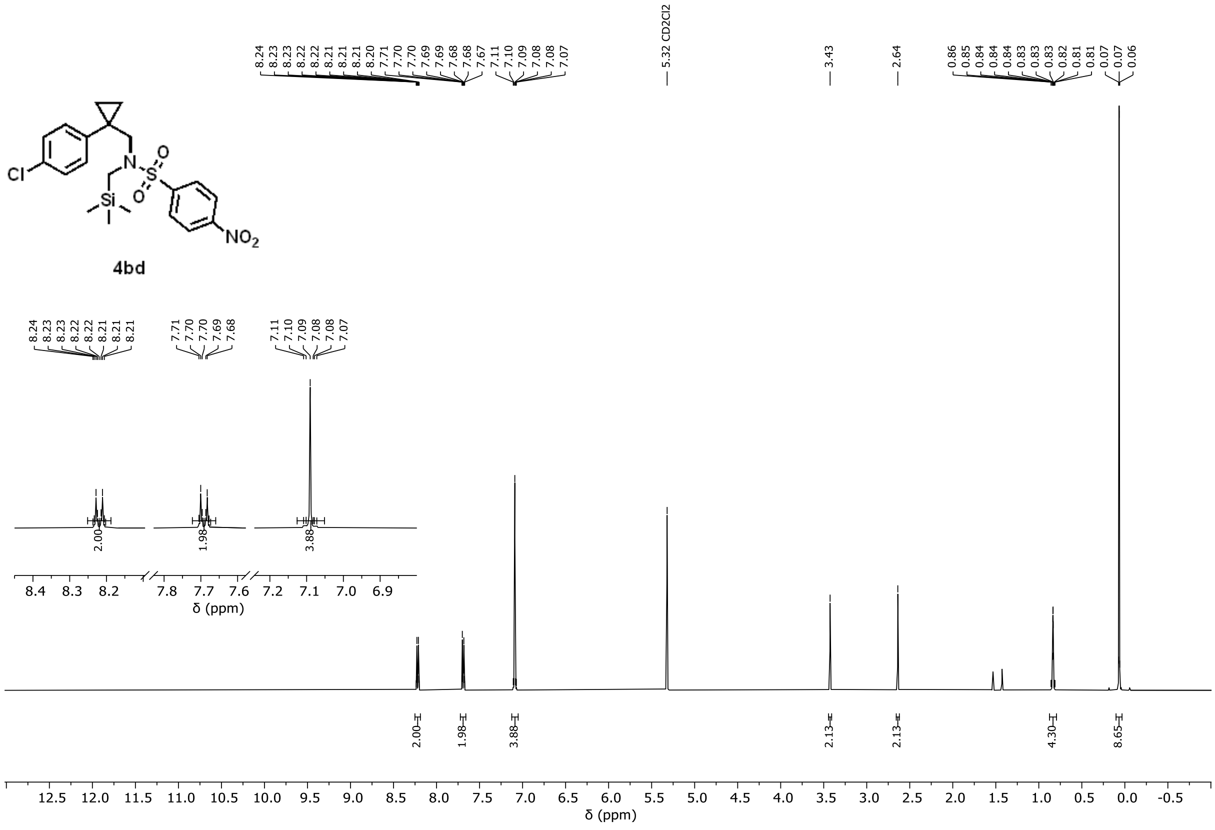

Figure **S68**: ^{1}H NMR (400 MHz, CD_2Cl_2) of **4bd**.

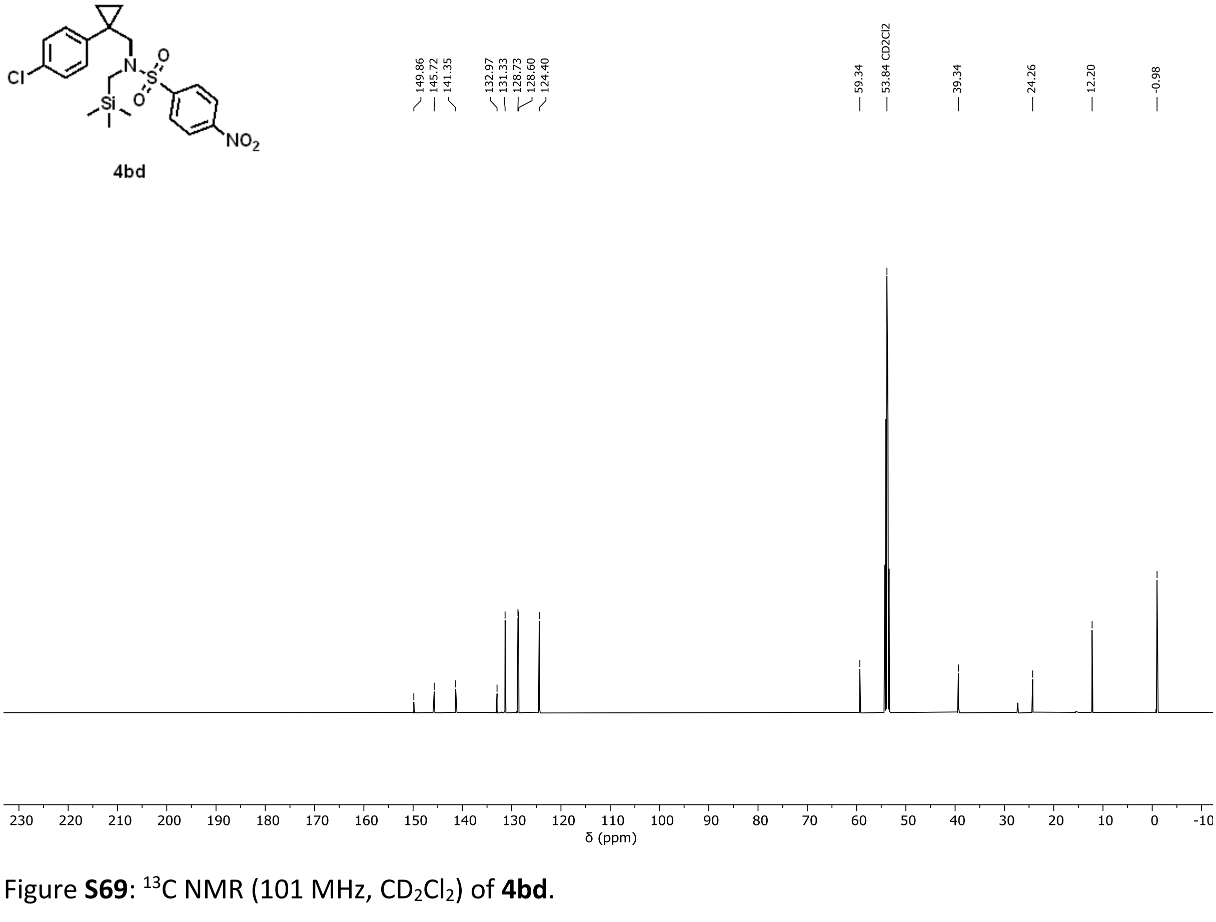

Figure **S69**: ^{13}C NMR (101 MHz, CD_2Cl_2) of **4bd**.

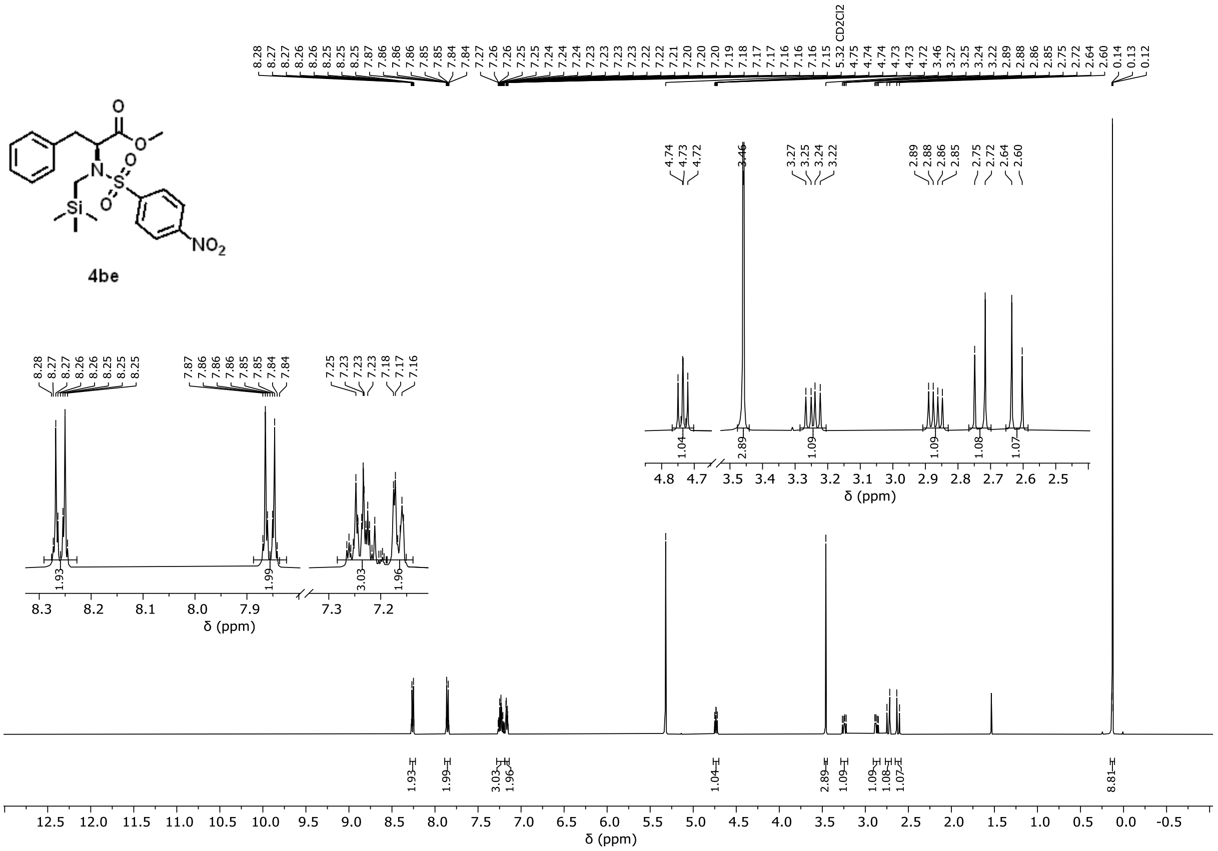

Figure **S70**: ^{1}H NMR (400 MHz, CD_2Cl_2) of **4be**.

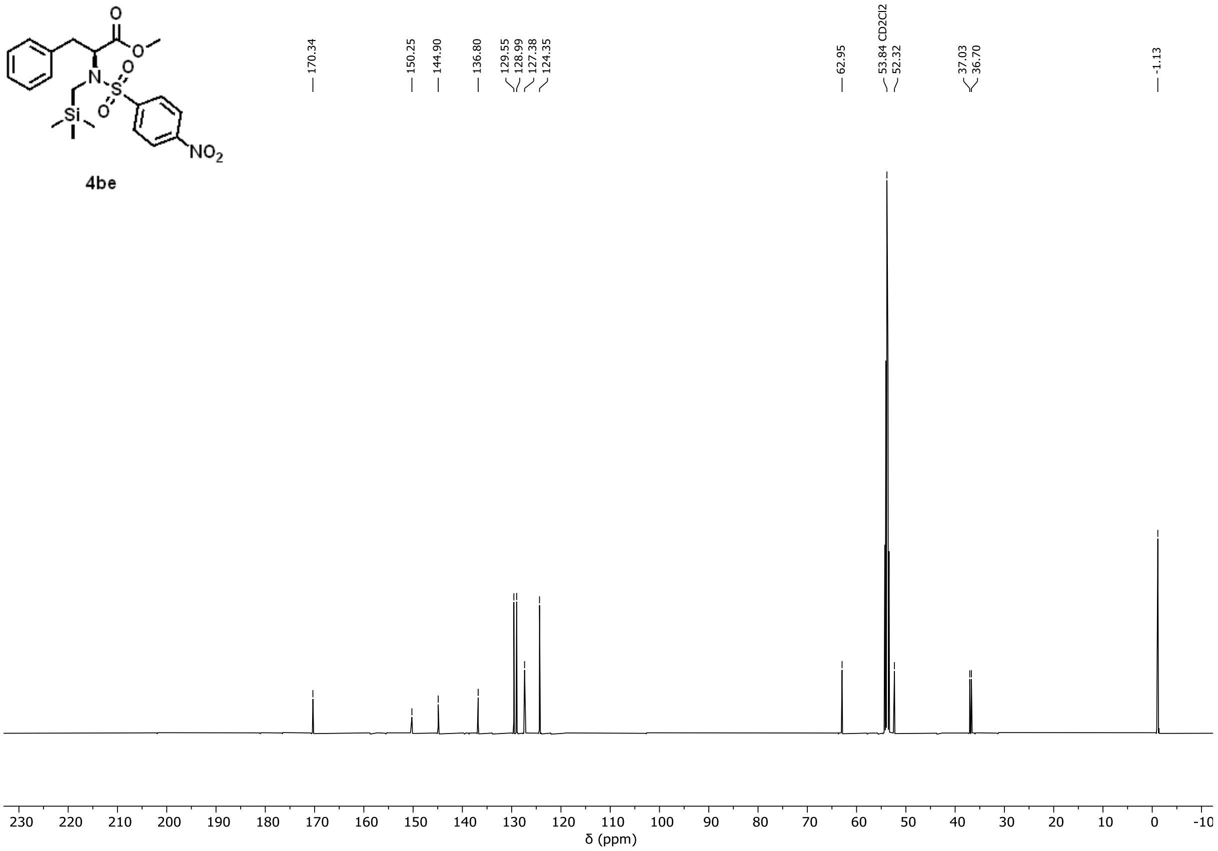

Figure **S71**: ^{13}C NMR (101 MHz, CD_2Cl_2) of **4be**.

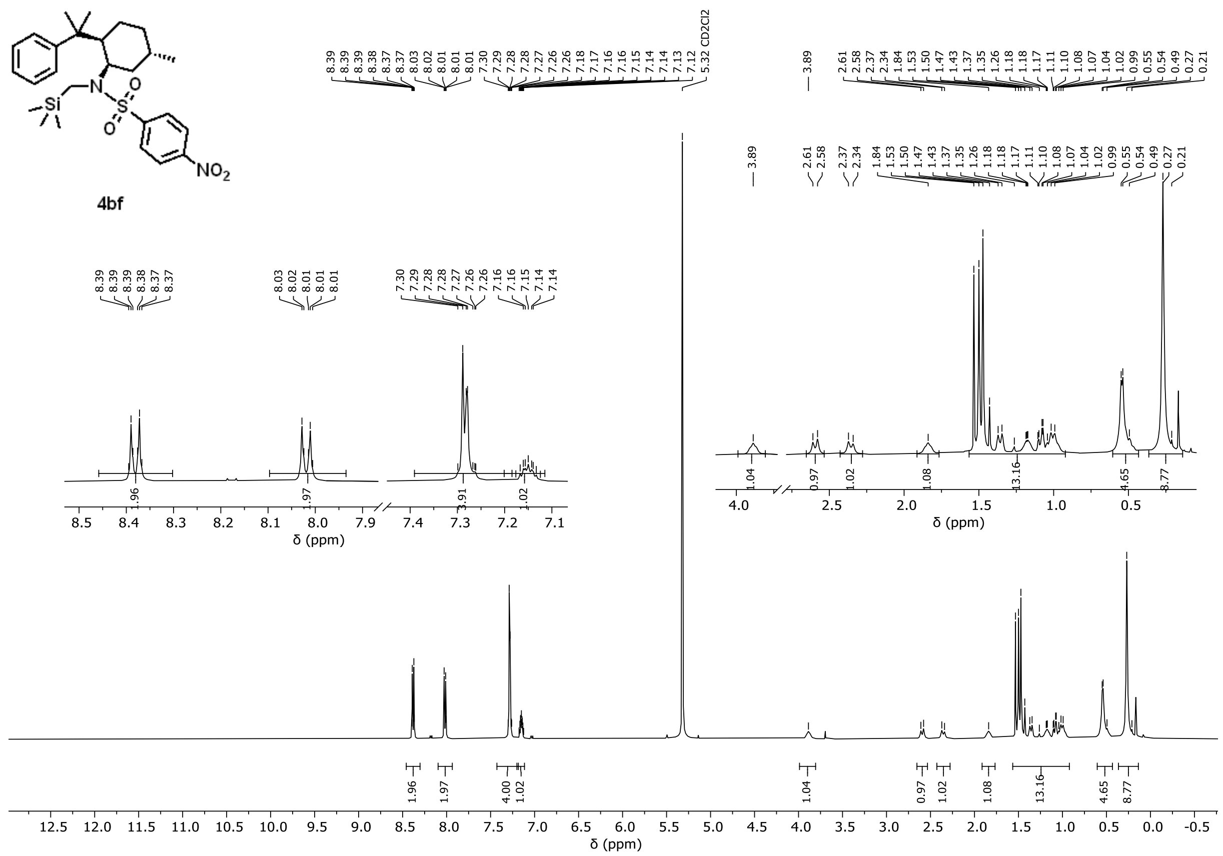

Figure **S72**: ^{1}H NMR (400 MHz, CD_2Cl_2) of **4bf**.

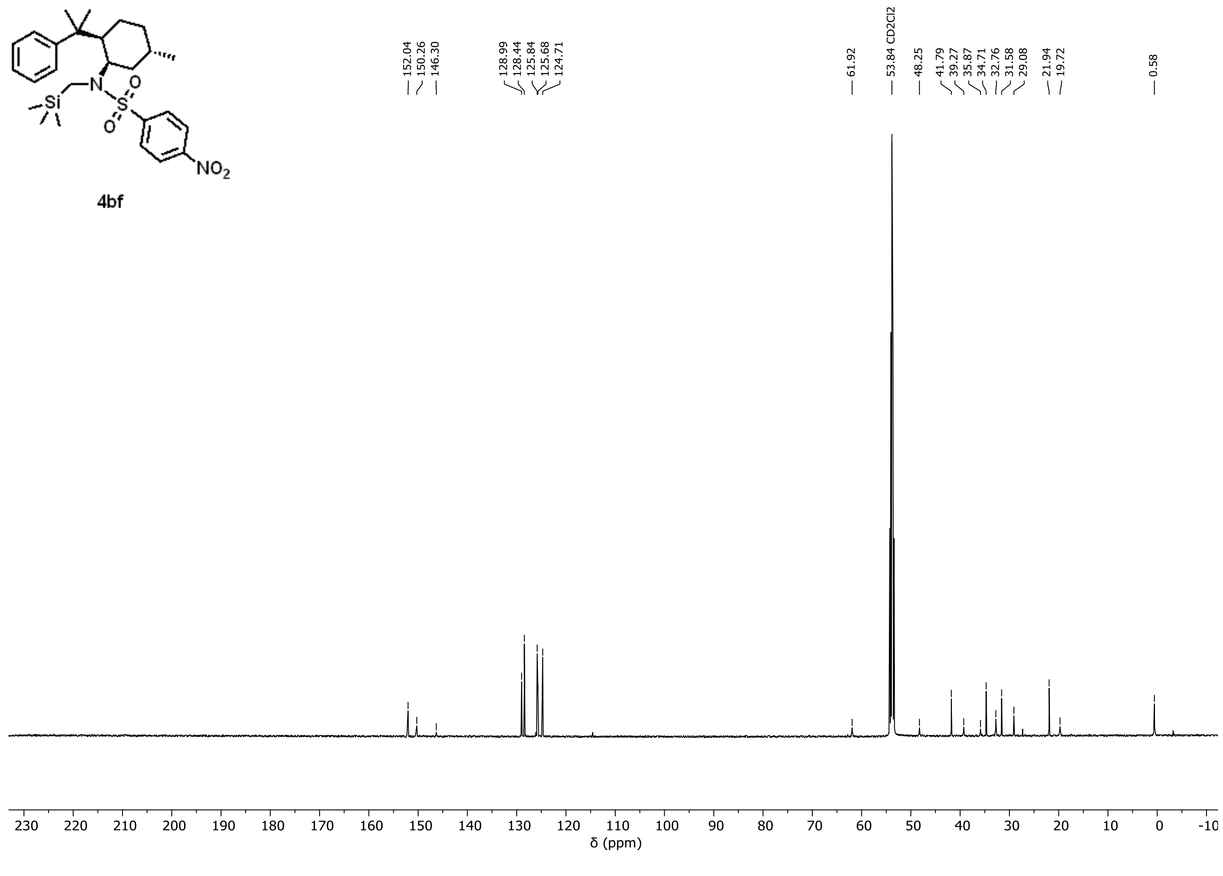

Figure **S73**: ^{13}C NMR (101 MHz, CD_2Cl_2) of **4bf**.

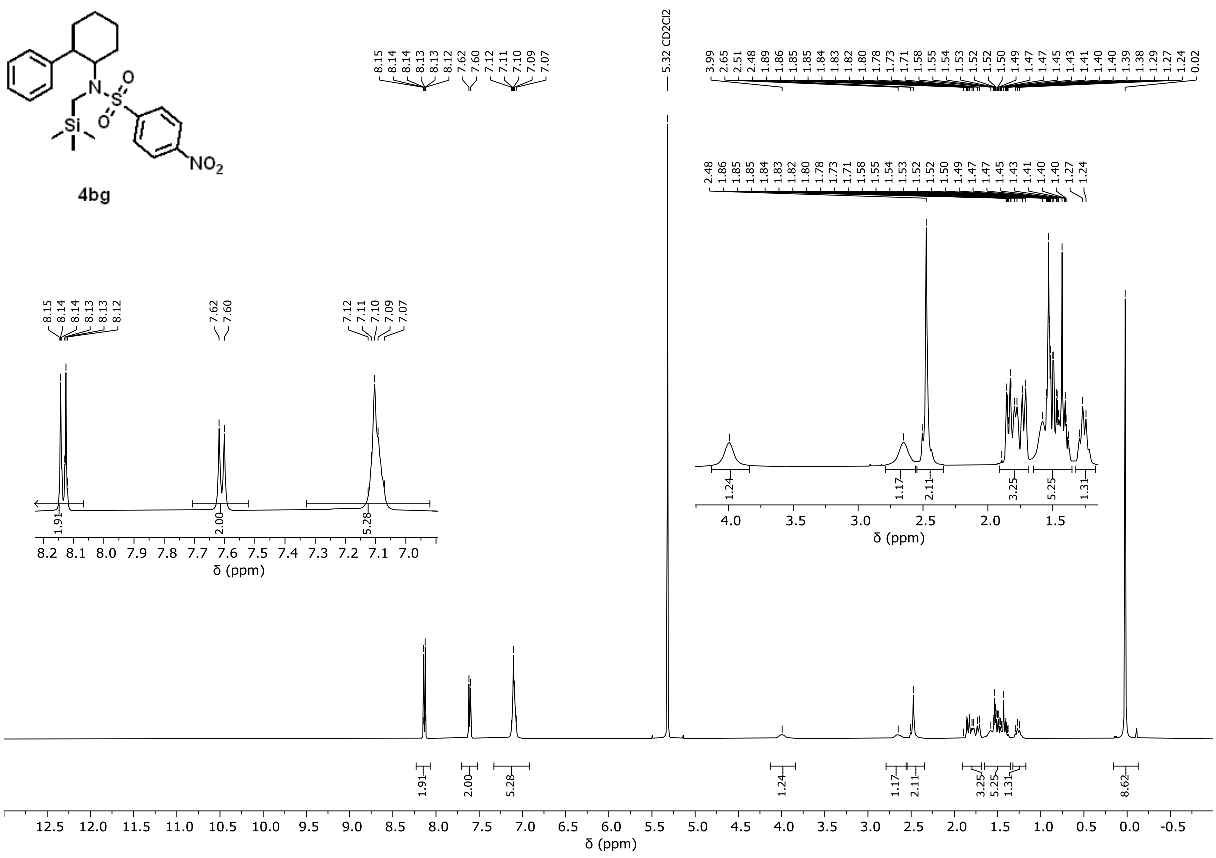

Figure **S74**: ^{1}H NMR (400 MHz, CD_2Cl_2) of **4bg**.

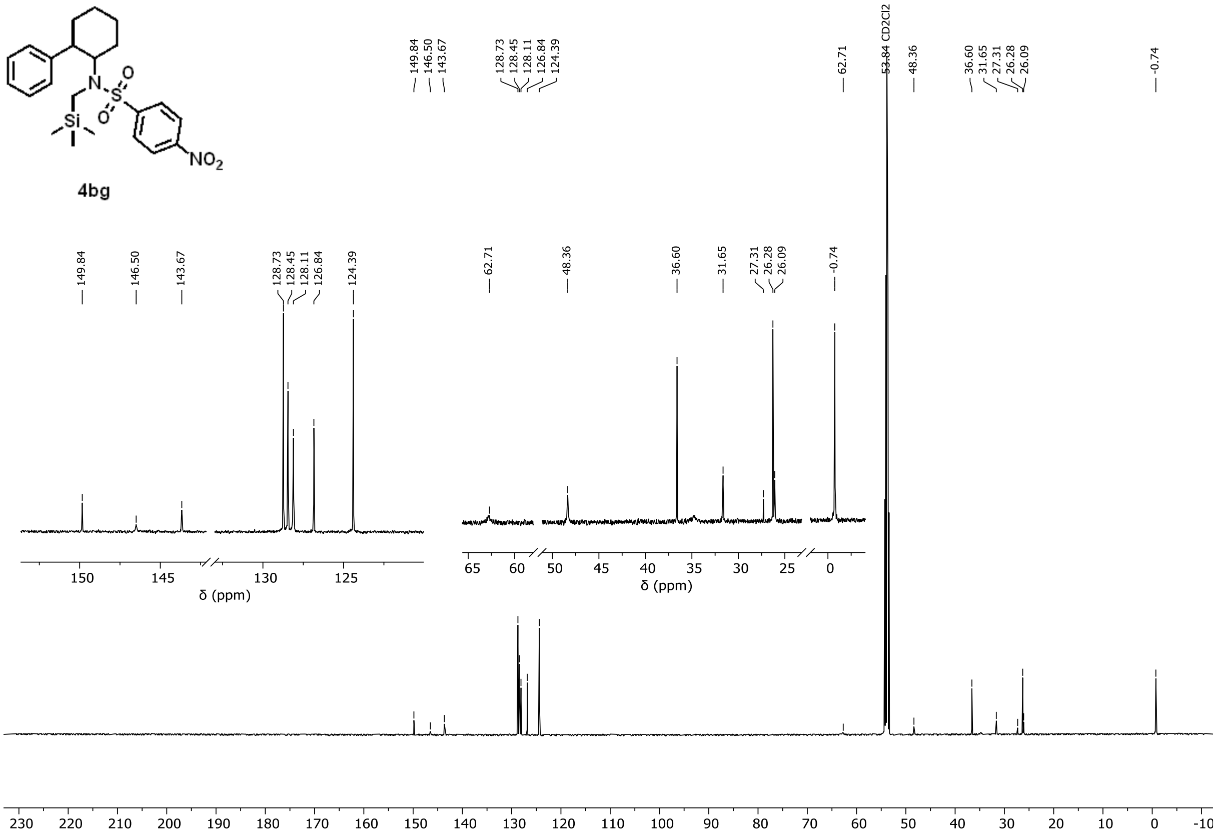

Figure **S75**: ^{13}C NMR (101 MHz, CD_2Cl_2) of **4bg**.

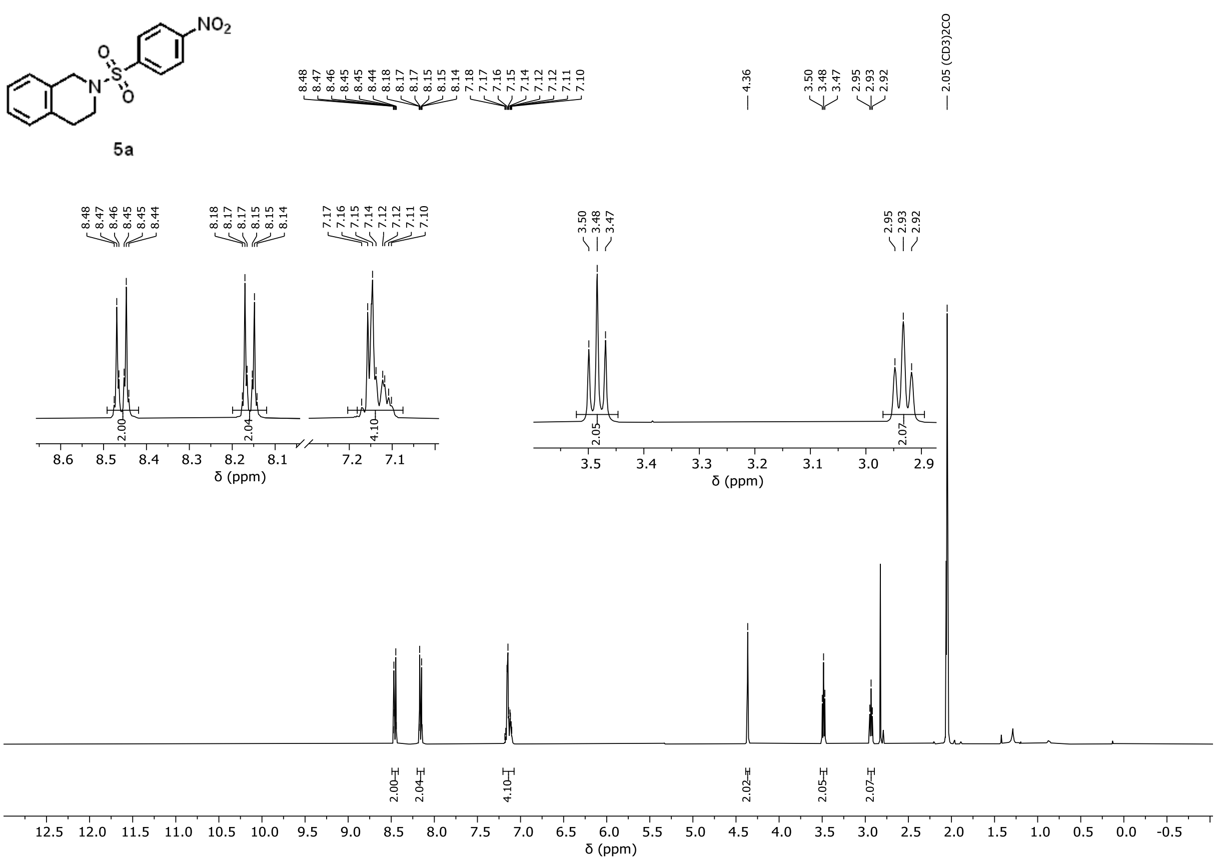

Figure **S76**: ^{1}H NMR (400 MHz, acetone-d_6) of **5a**.

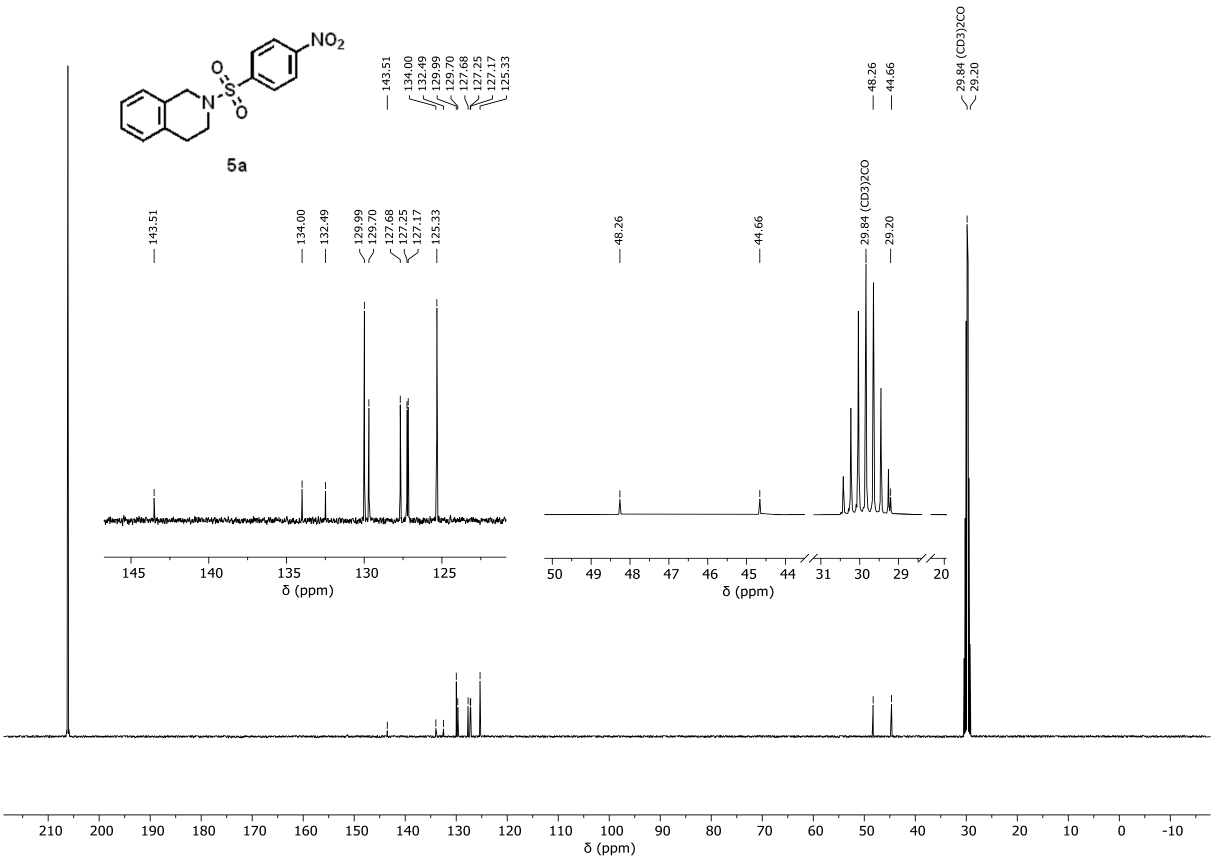

Figure **S77**: ^{13}C NMR (101 MHz, acetone-d_6) of **5a**.

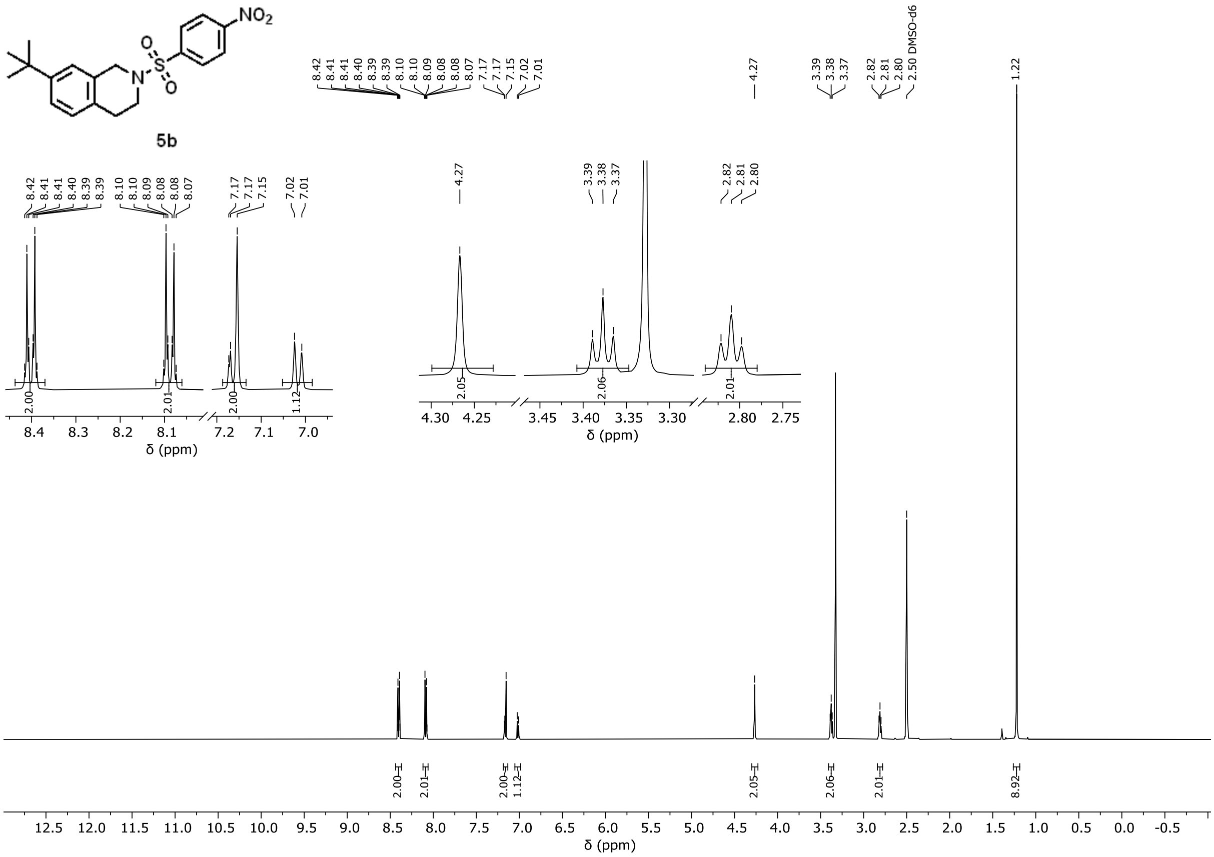

Figure **S78**: ^{1}H NMR (400 MHz, DMSO-d$_6$) of **5b**.

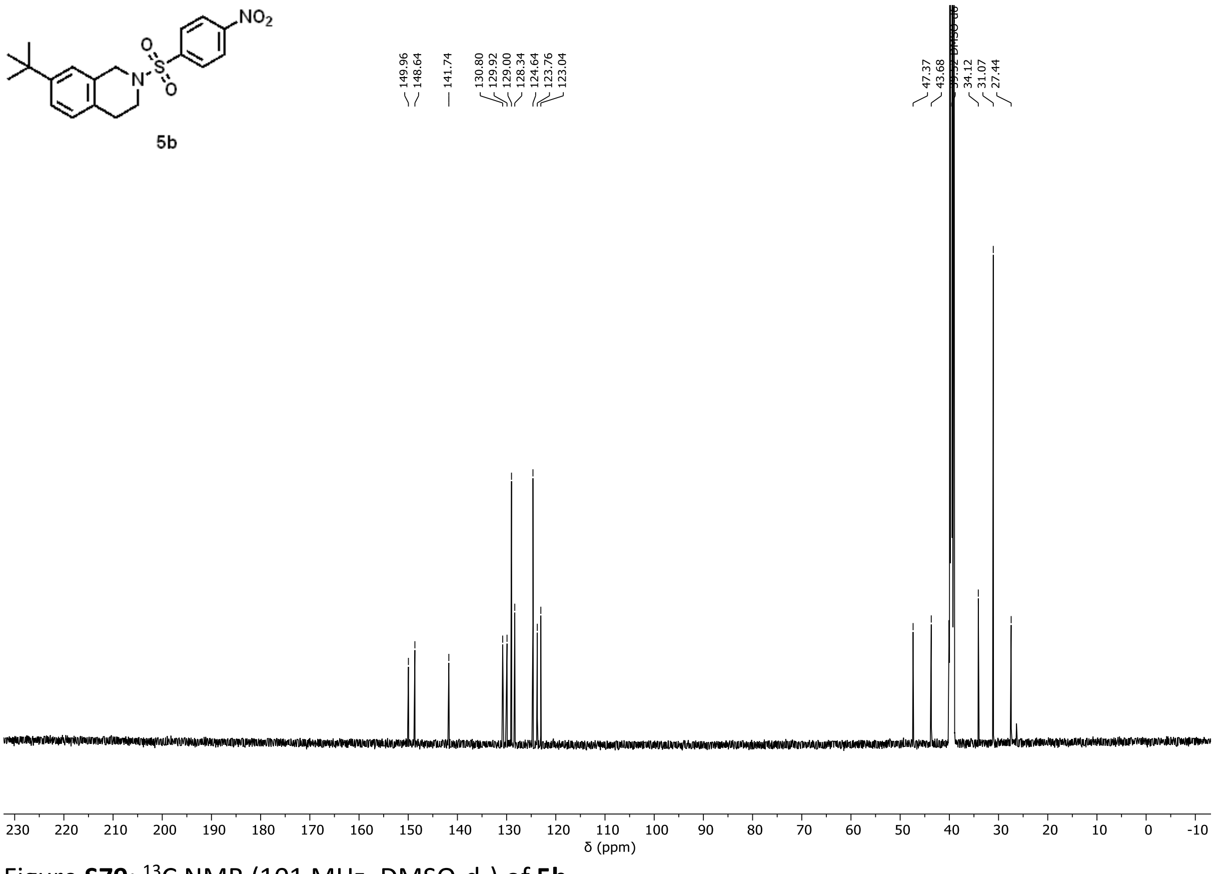

Figure **S79**: ^{13}C NMR (101 MHz, DMSO-d$_6$) of **5b**.

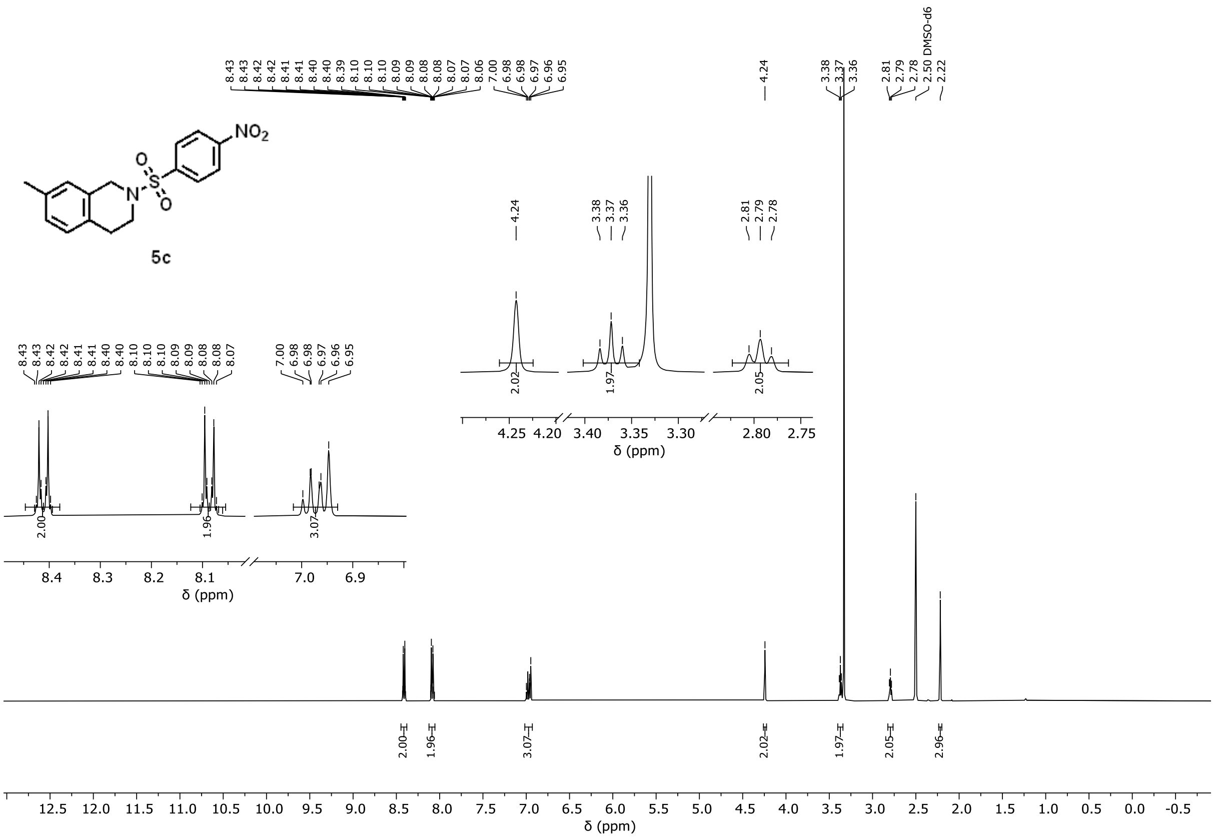

Figure **S80**: ^{1}H NMR (400 MHz, DMSO-d_6) of **5c**.

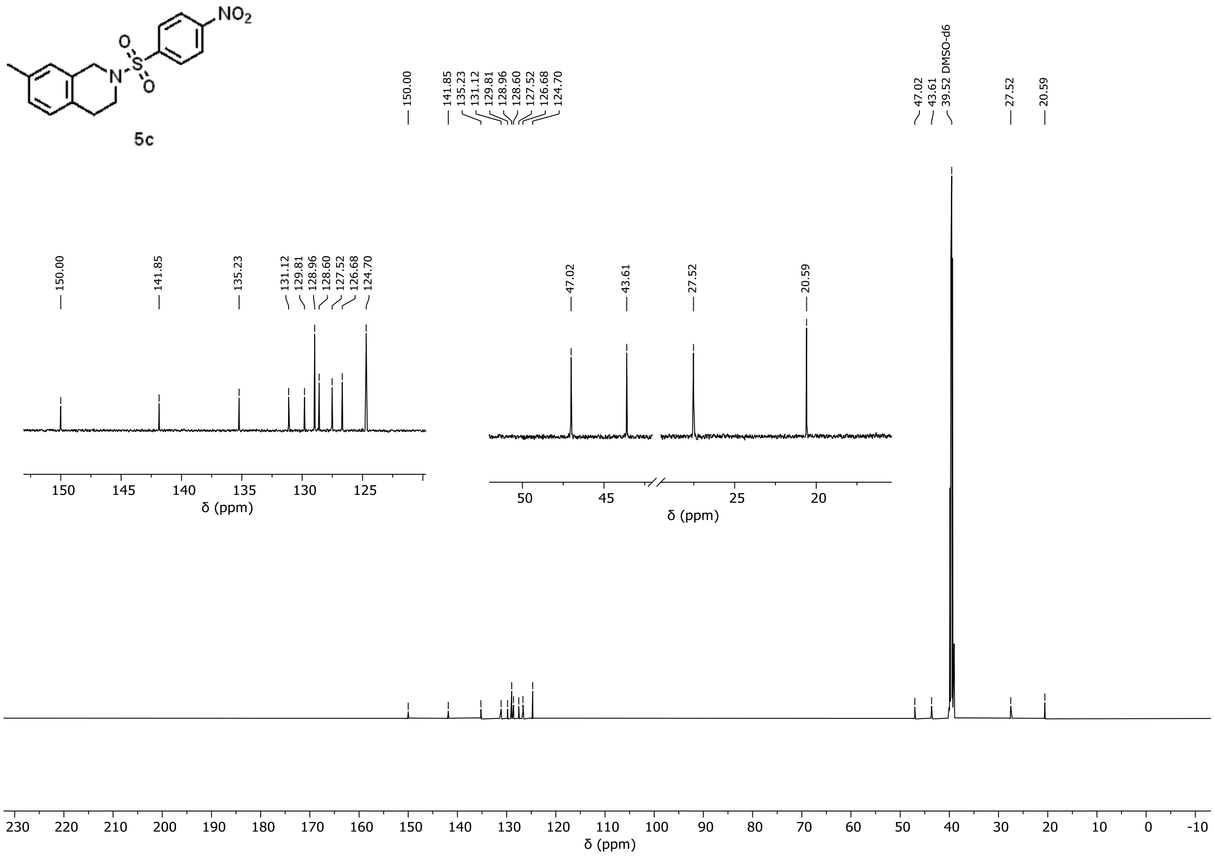

Figure **S81**: ^{13}C NMR (101 MHz, DMSO-d_6) of **5c**.

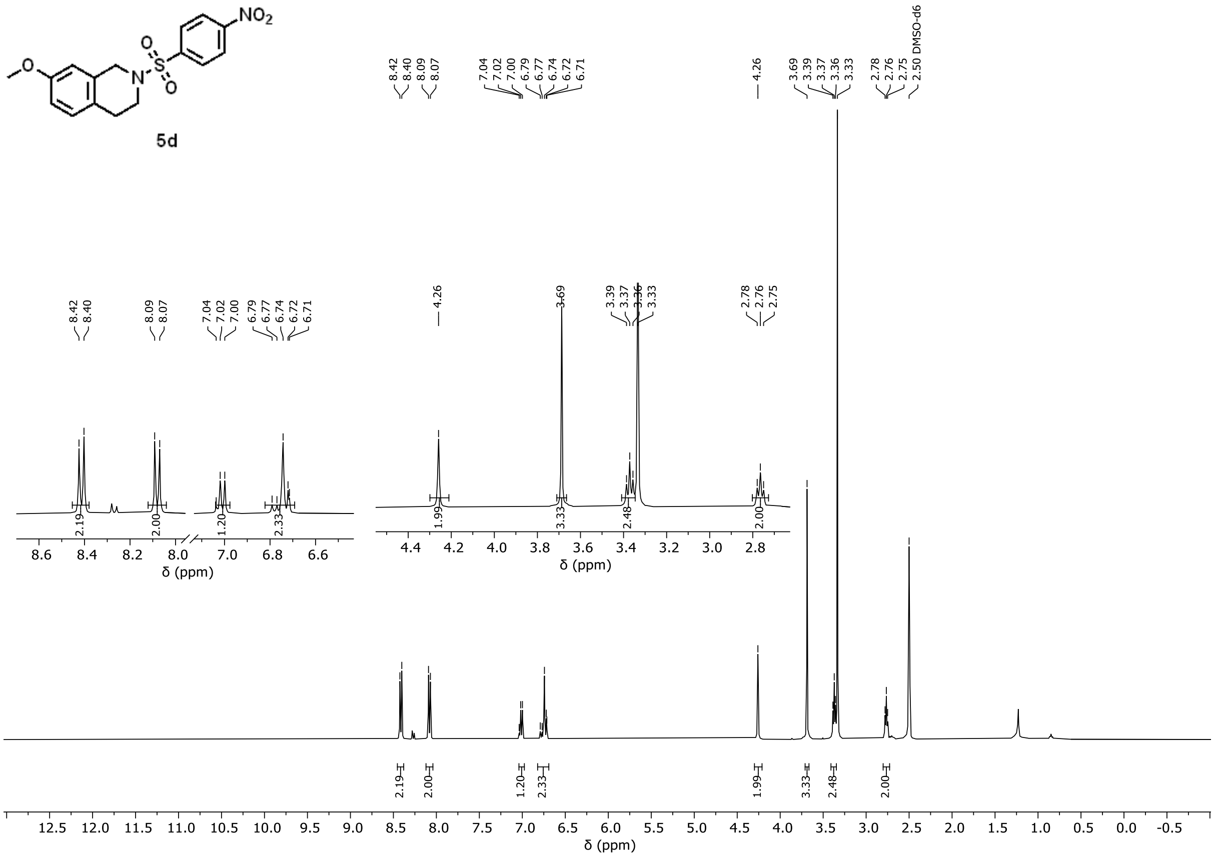

Figure **S82**: ^{1}H NMR (400 MHz, DMSO-d_6) of **5d**.

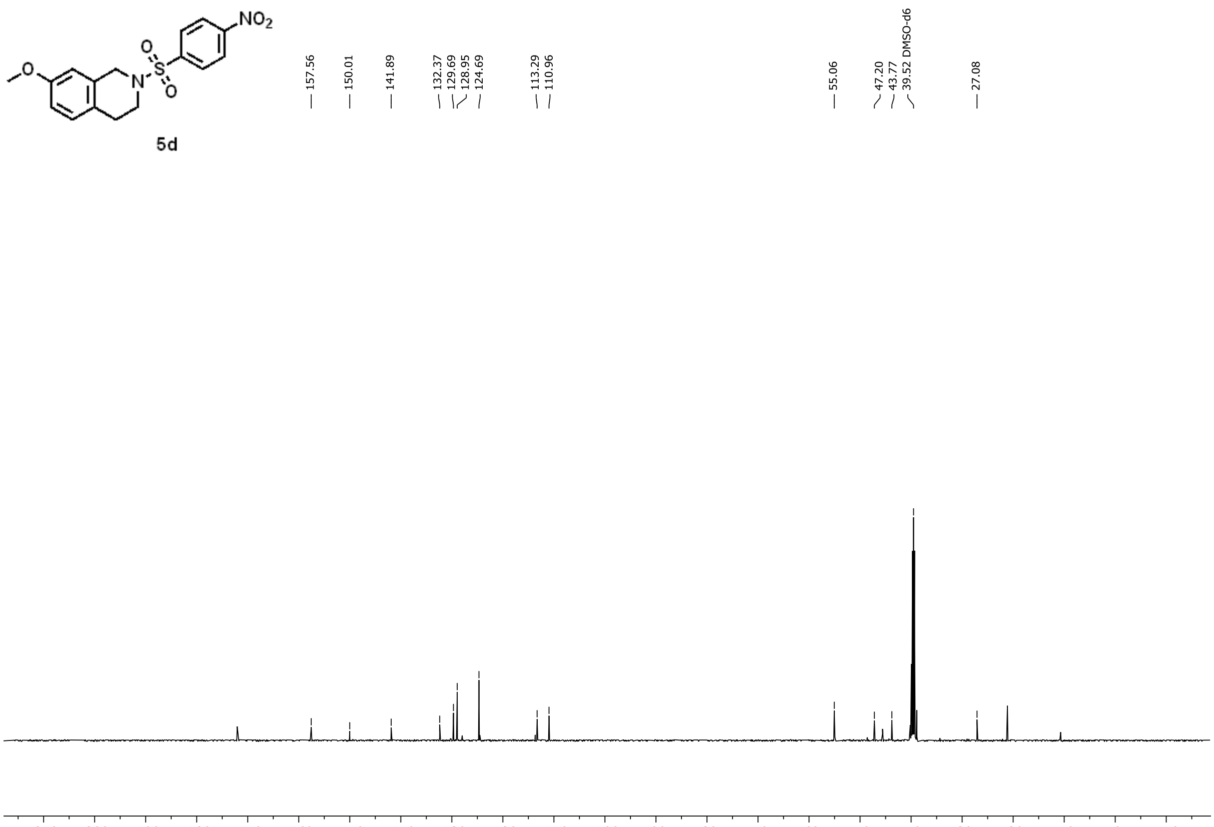

Figure **S83**: ^{13}C NMR (101 MHz, DMSO-d_6) of **5d**.

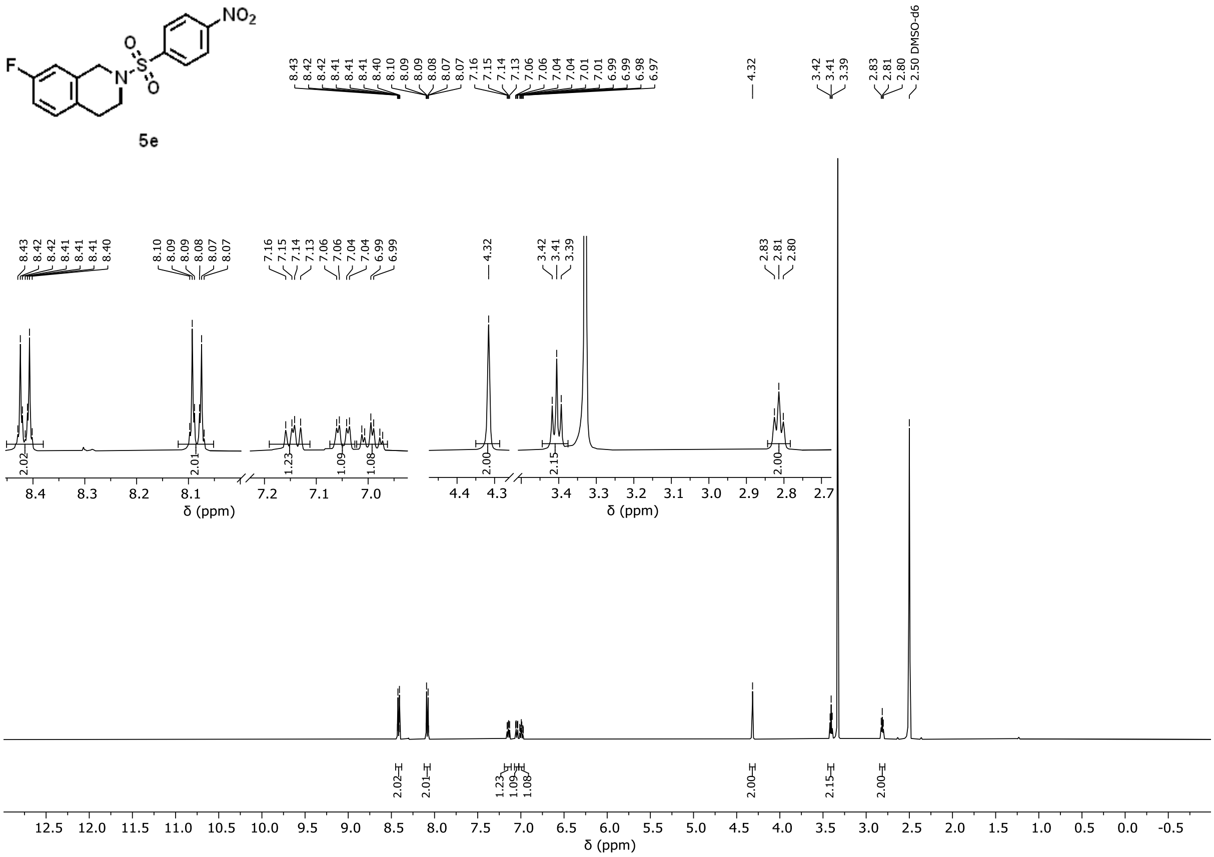

Figure **S84**: ^{1}H NMR (400 MHz, DMSO-d_6) of **5e**.

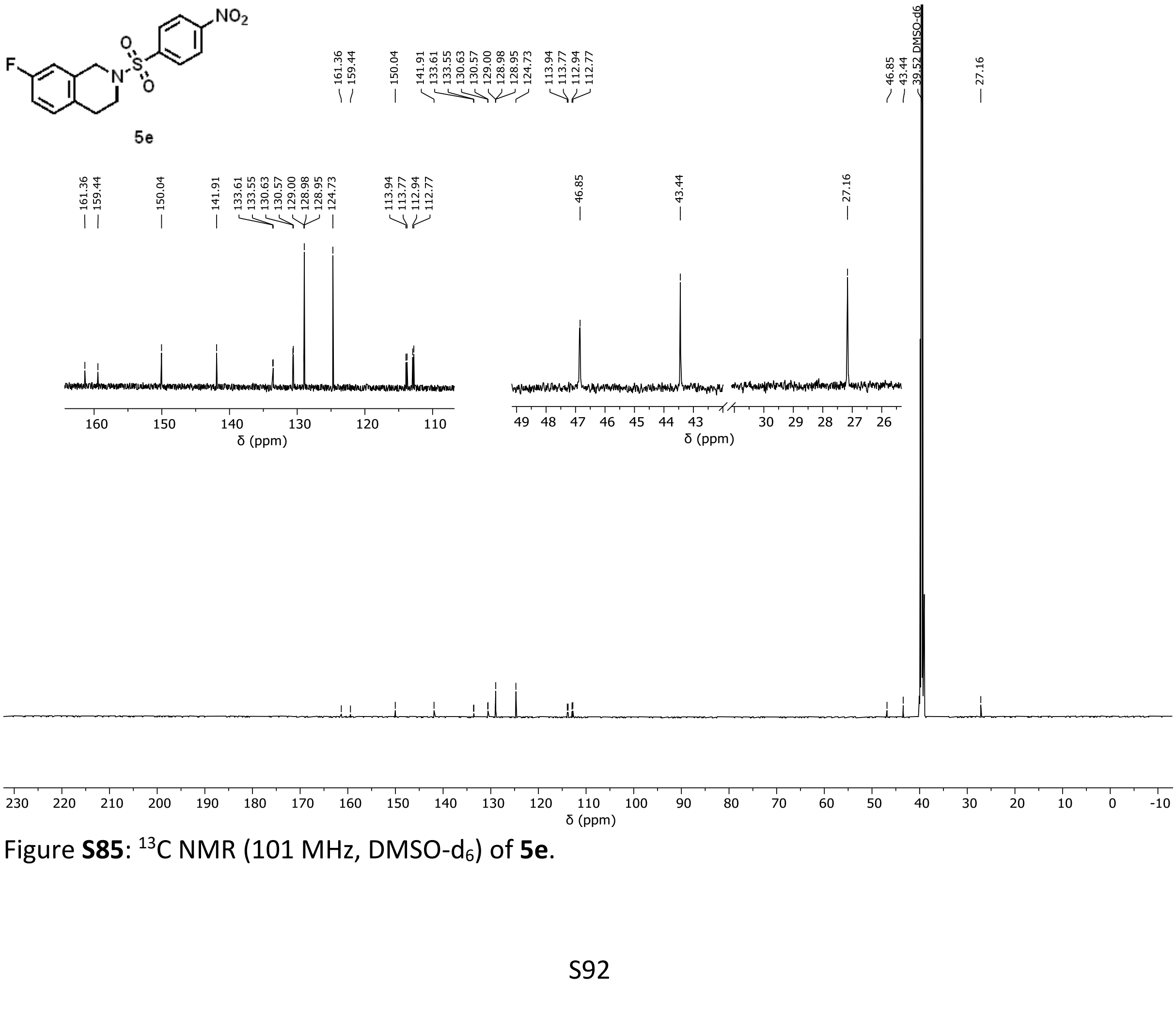

Figure **S85**: ^{13}C NMR (101 MHz, DMSO-d_6) of **5e**.

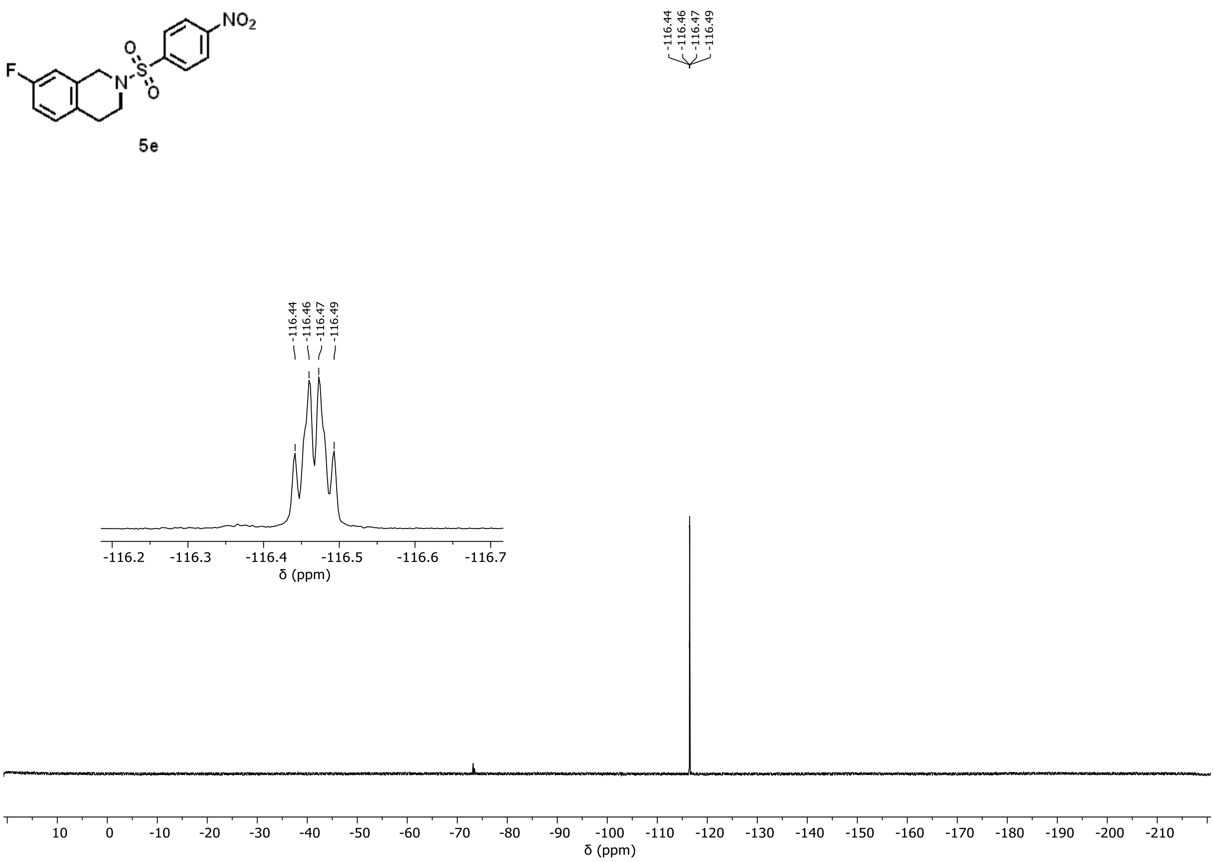

Figure **S86**: ^{19}F NMR (376 MHz, DMSO-d_6) of **5e**.

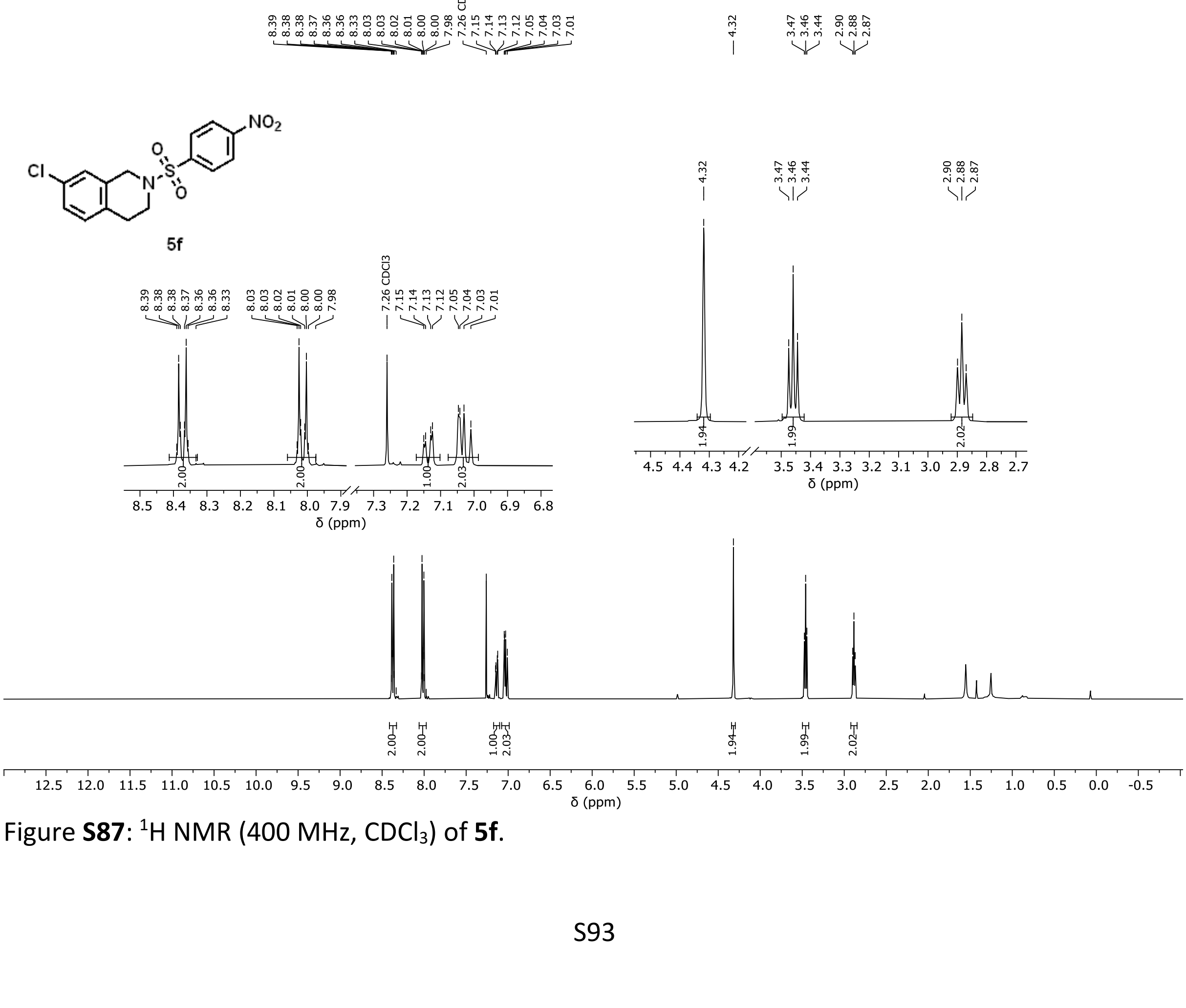

Figure **S87**: ^{1}H NMR (400 MHz, $CDCl_3$) of **5f**.

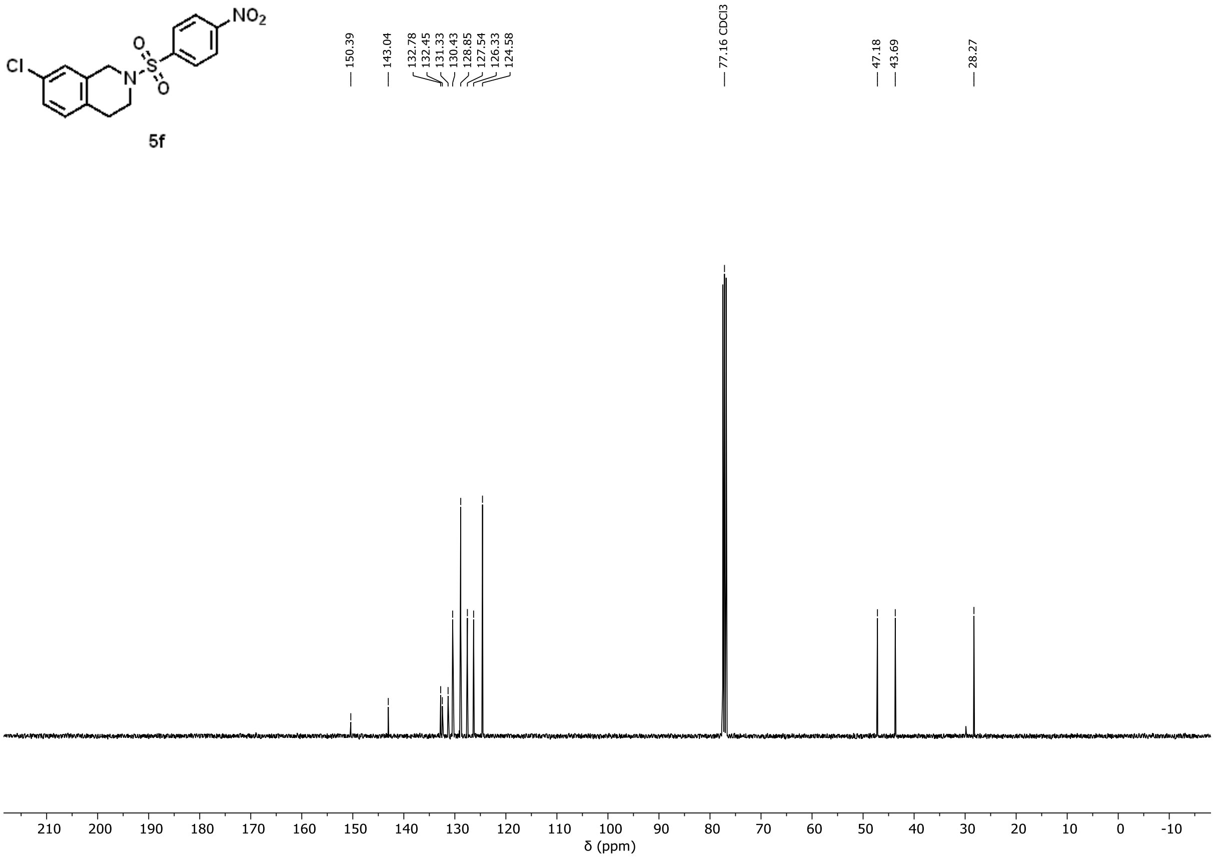

Figure **S88**: ^{13}C NMR (101 MHz, $CDCl_3$) of **5f**.

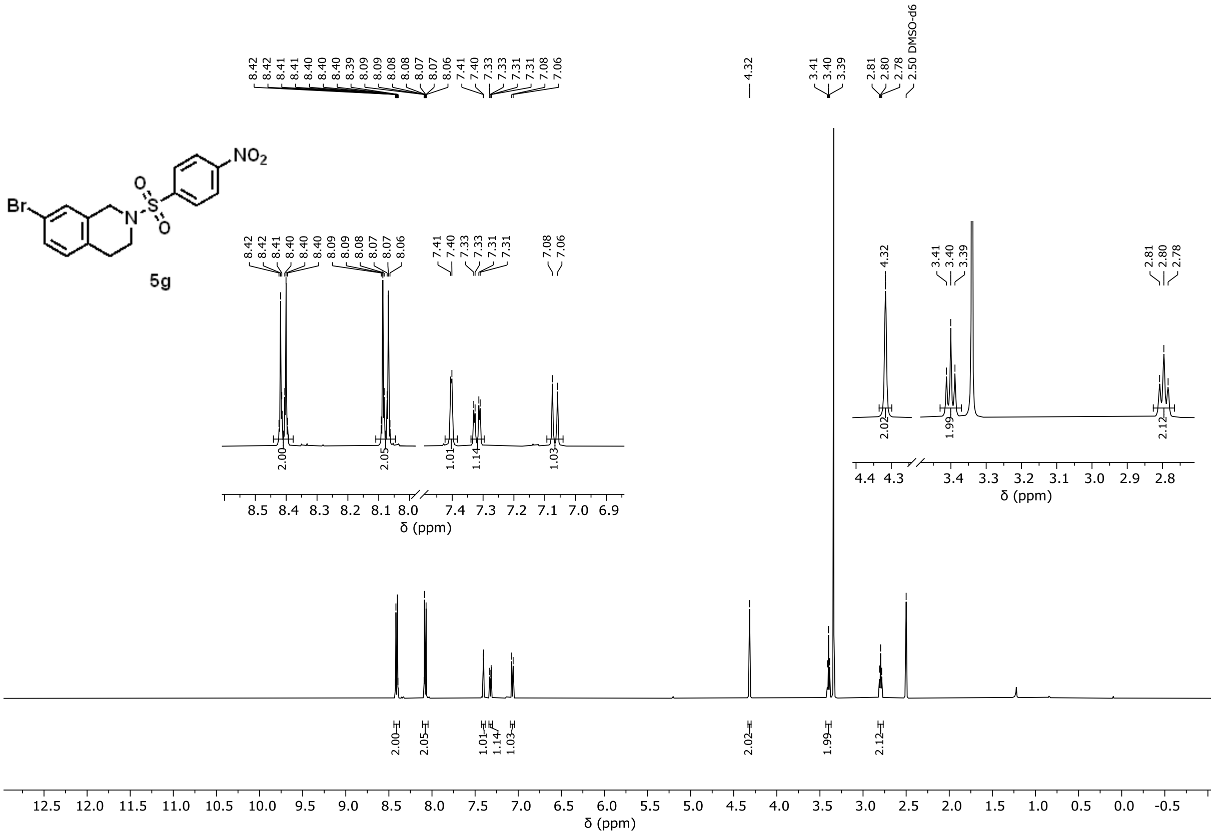

Figure **S89**: ^{1}H NMR (400 MHz, DMSO-d_6) of **5g**.

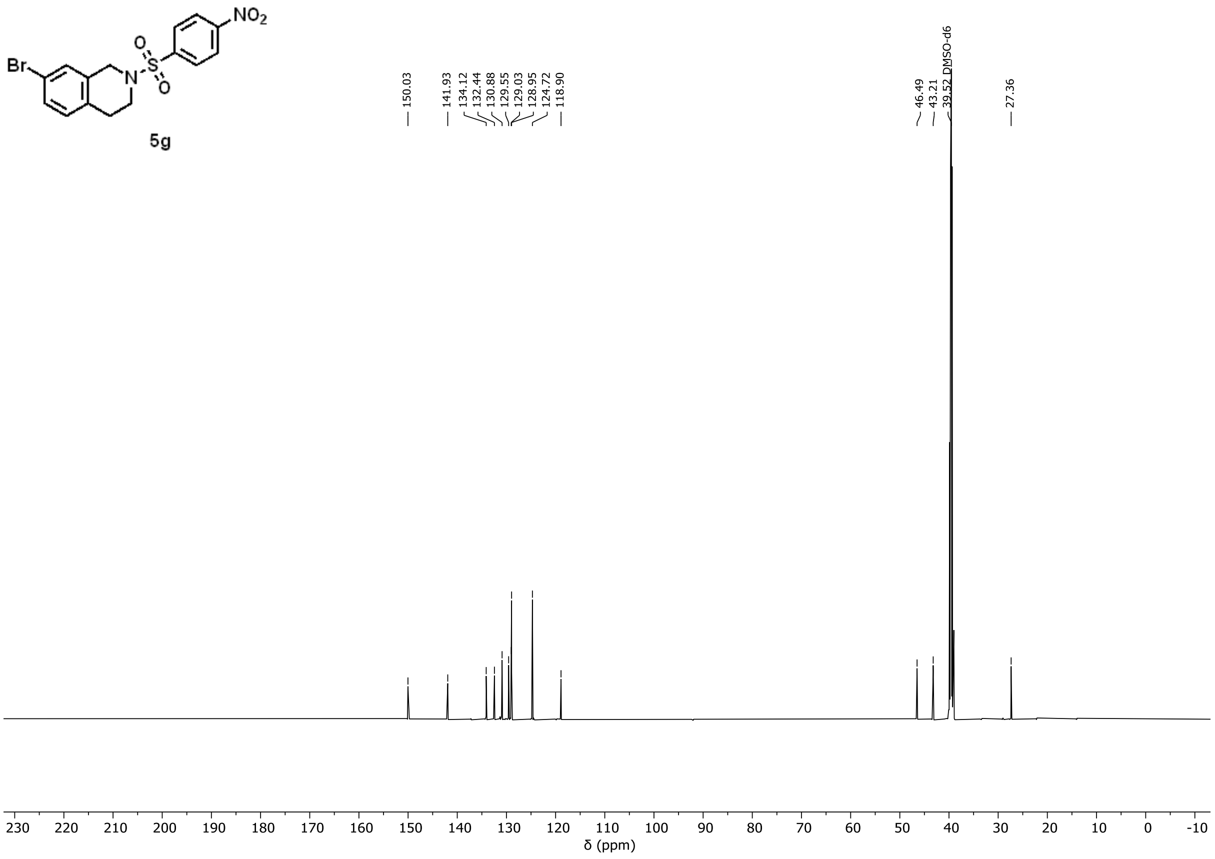

Figure **S90**: ^{13}C NMR (101 MHz, DMSO-d_6) of **5g**.

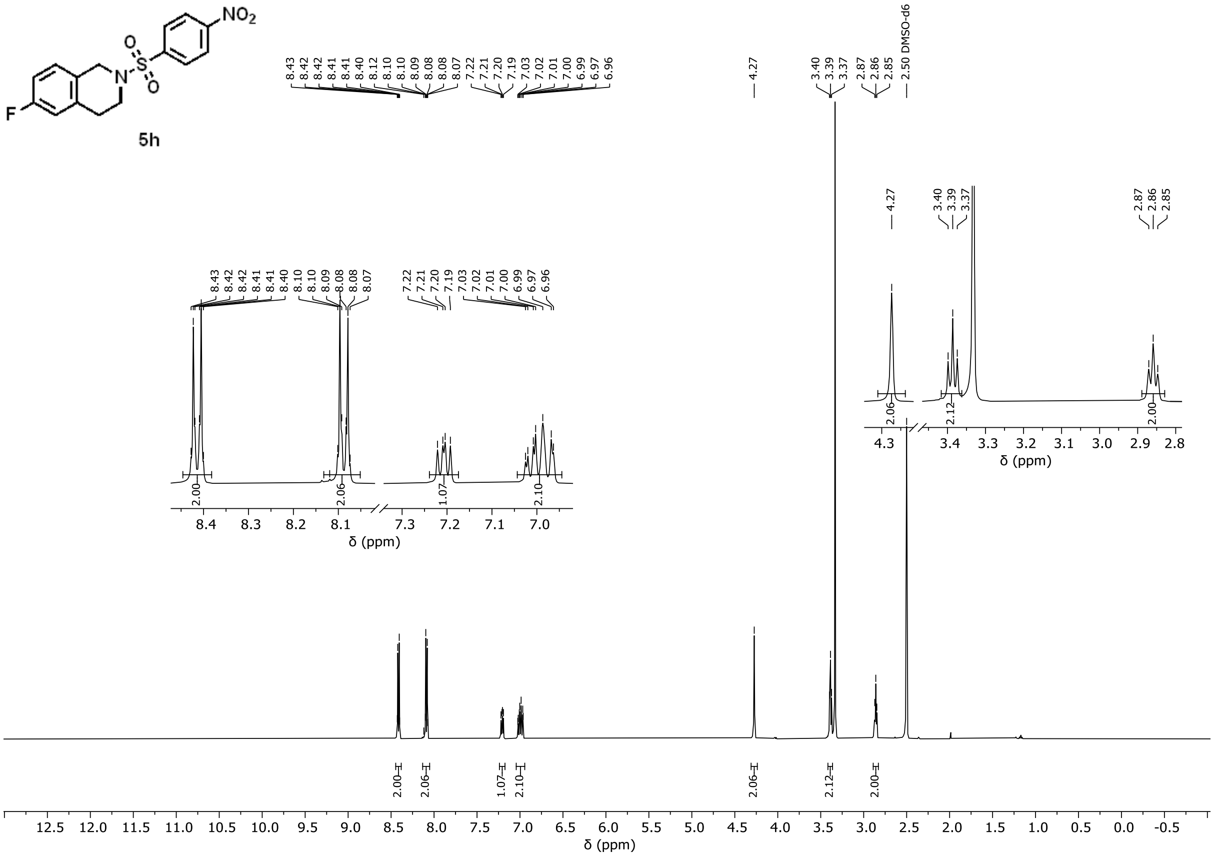

Figure **S91**: ^{1}H NMR (400 MHz, DMSO-d_6) of **5h**.

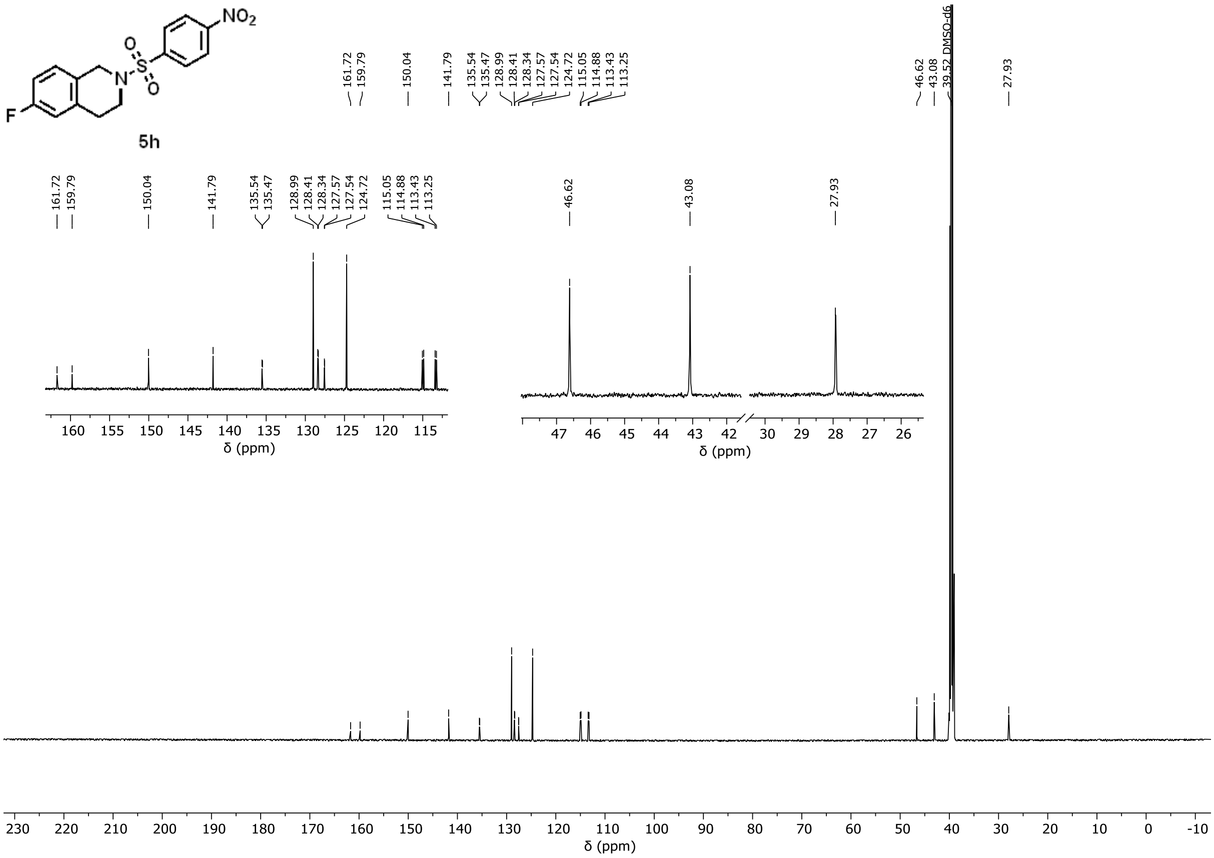

Figure **S92**: ^{13}C NMR (101 MHz, DMSO-d$_6$) of **5h**.

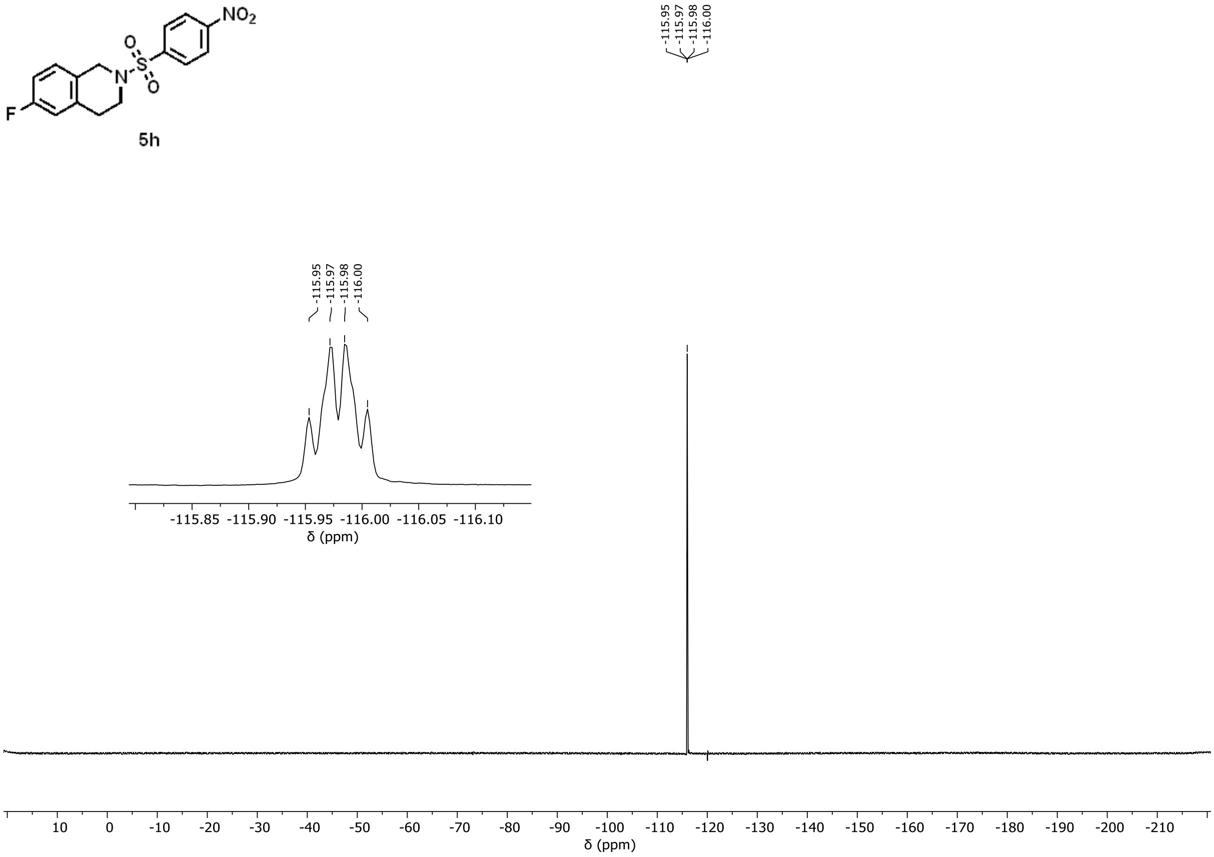

Figure **S93**: ^{19}F NMR (376 MHz, DMSO-d$_6$) of **5h**.

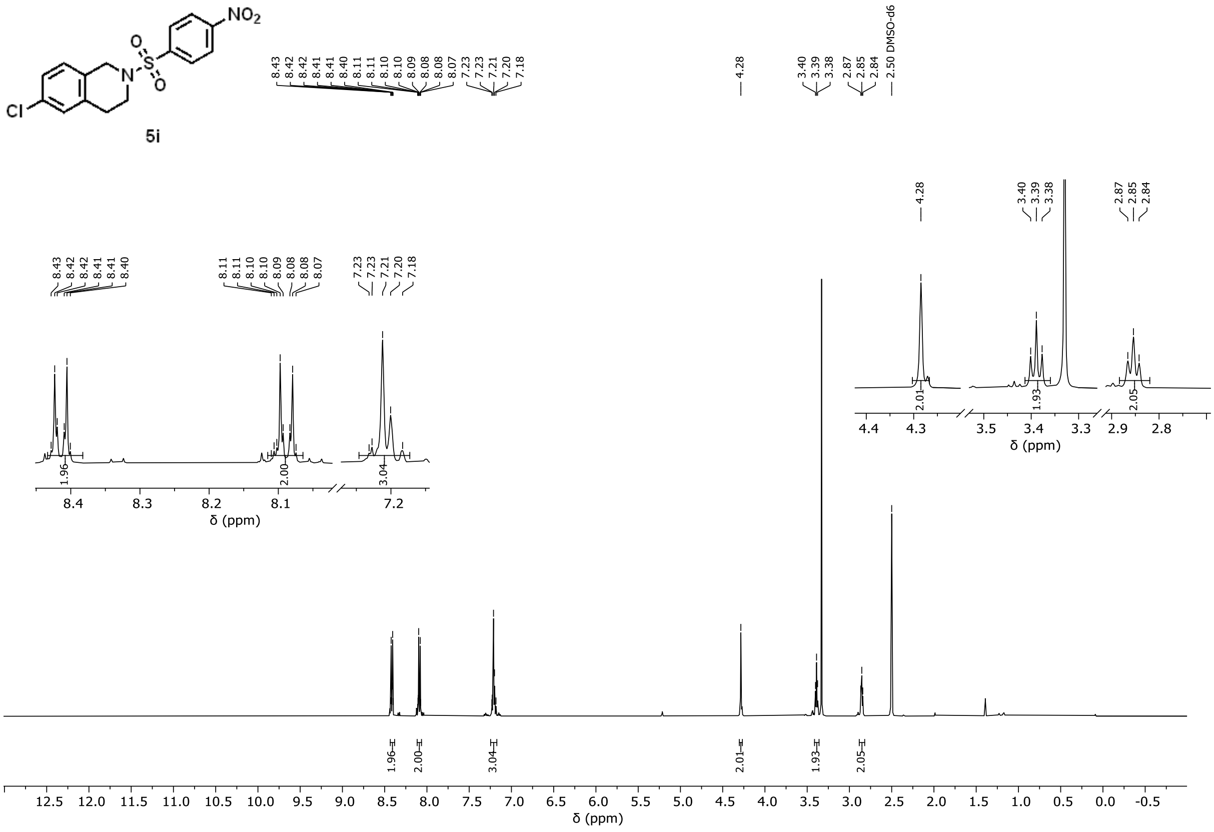

Figure **S94**: ^{1}H NMR (400 MHz, DMSO-d$_6$) of **5i**.

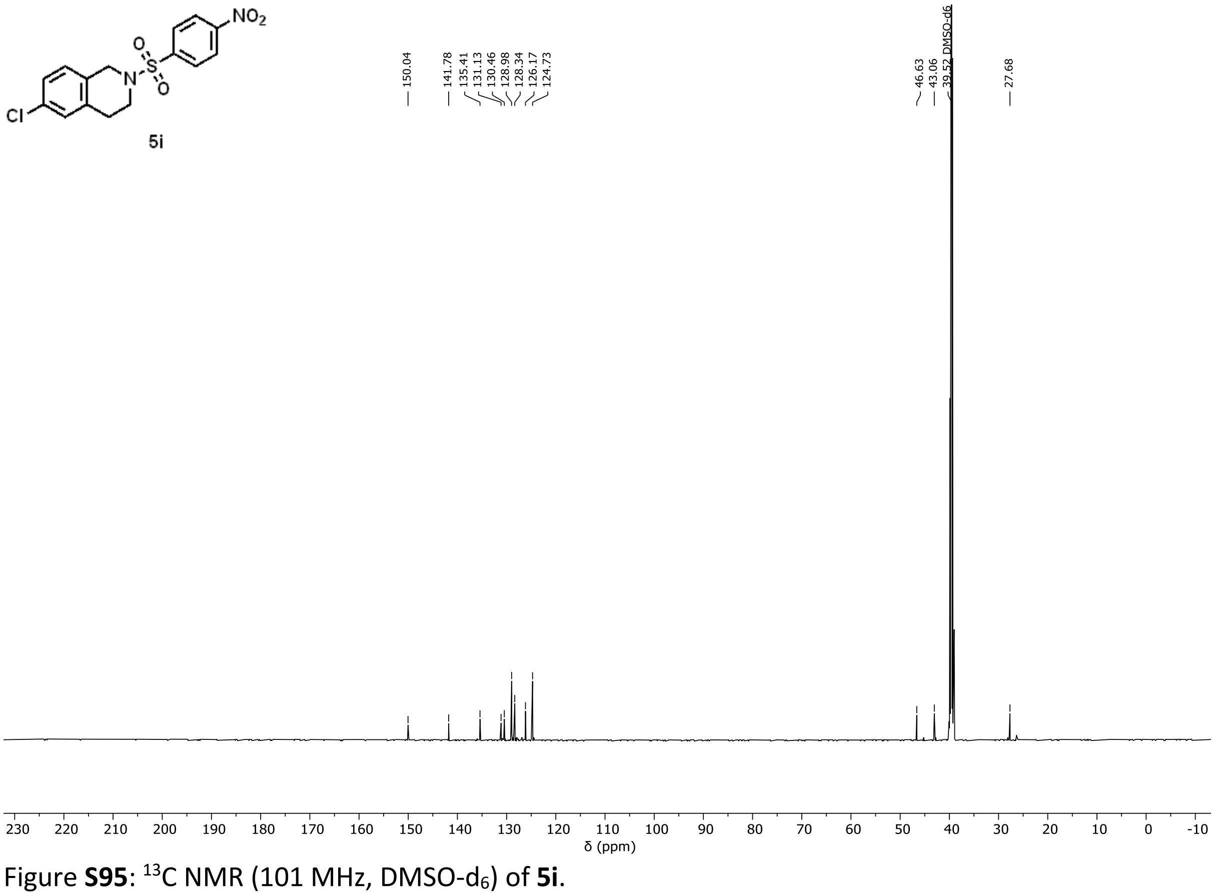

Figure **S95**: ^{13}C NMR (101 MHz, DMSO-d$_6$) of **5i**.

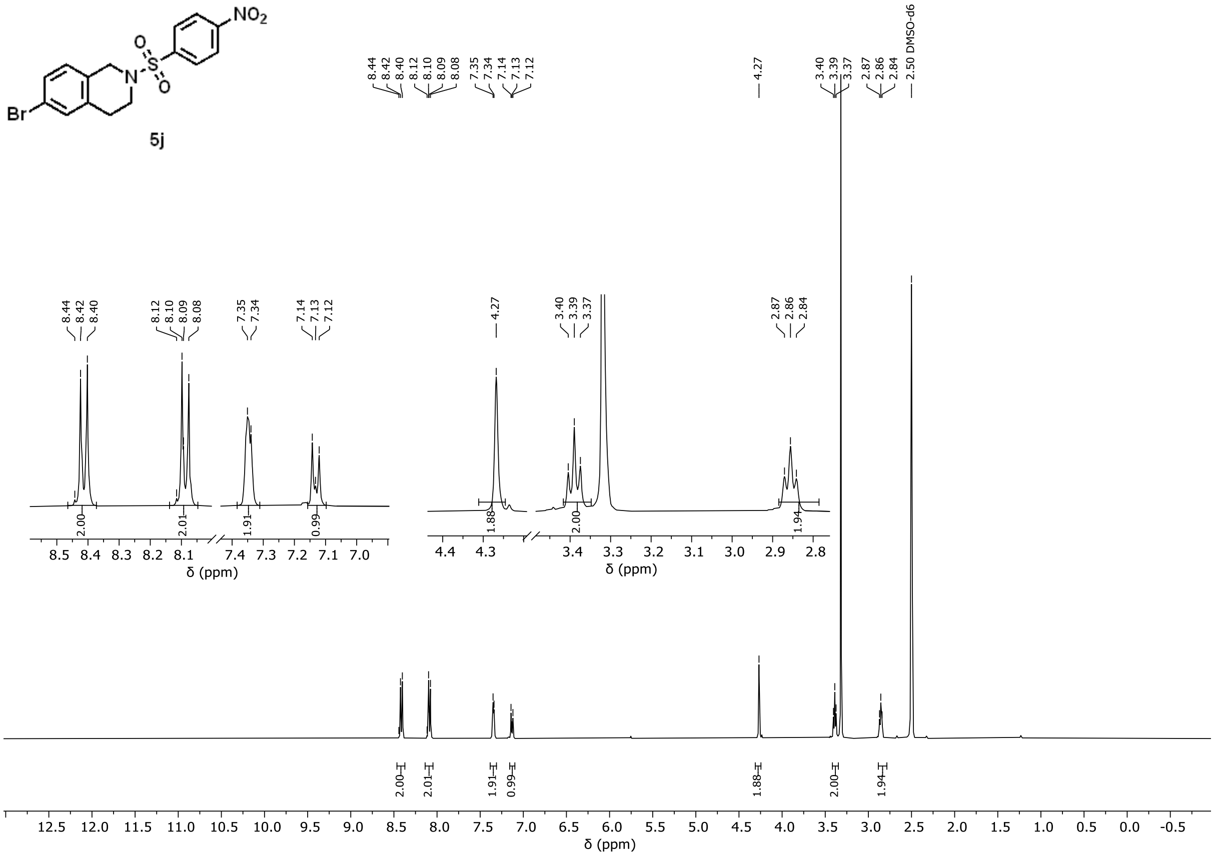

Figure **S96**: ^{1}H NMR (400 MHz, DMSO-d_6) of **5j**.

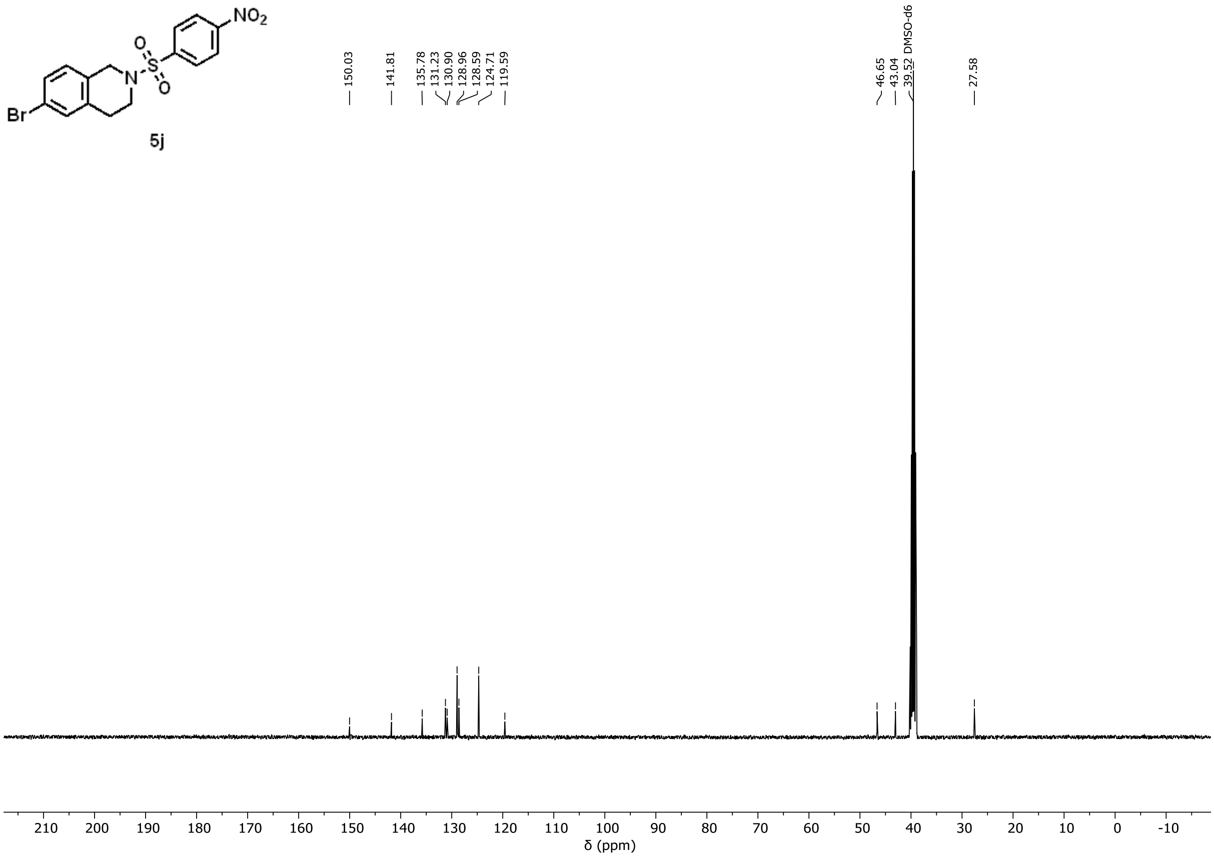

Figure **S97**: ^{13}C NMR (101 MHz, DMSO-d_6) of **5j**.

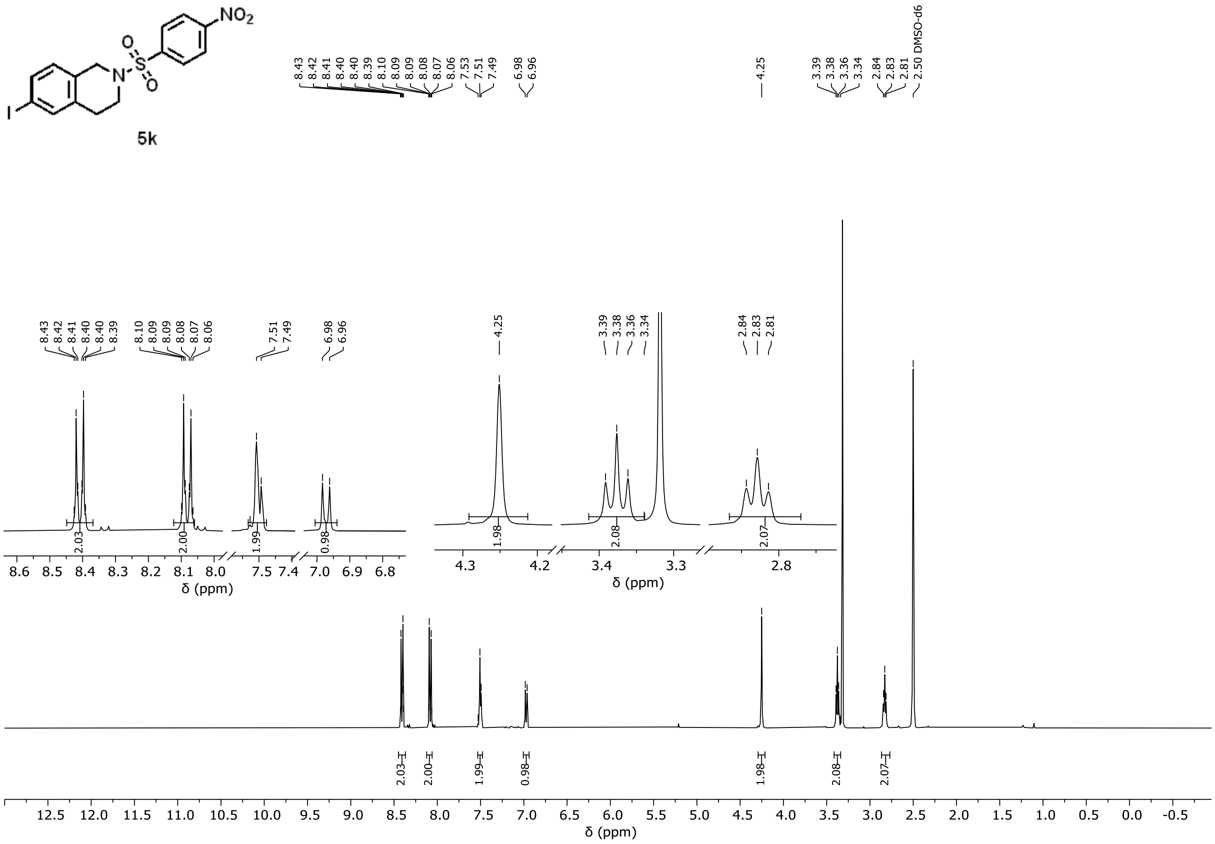

Figure **S98**: ^{1}H NMR (400 MHz, DMSO-d$_6$) of **5k**.

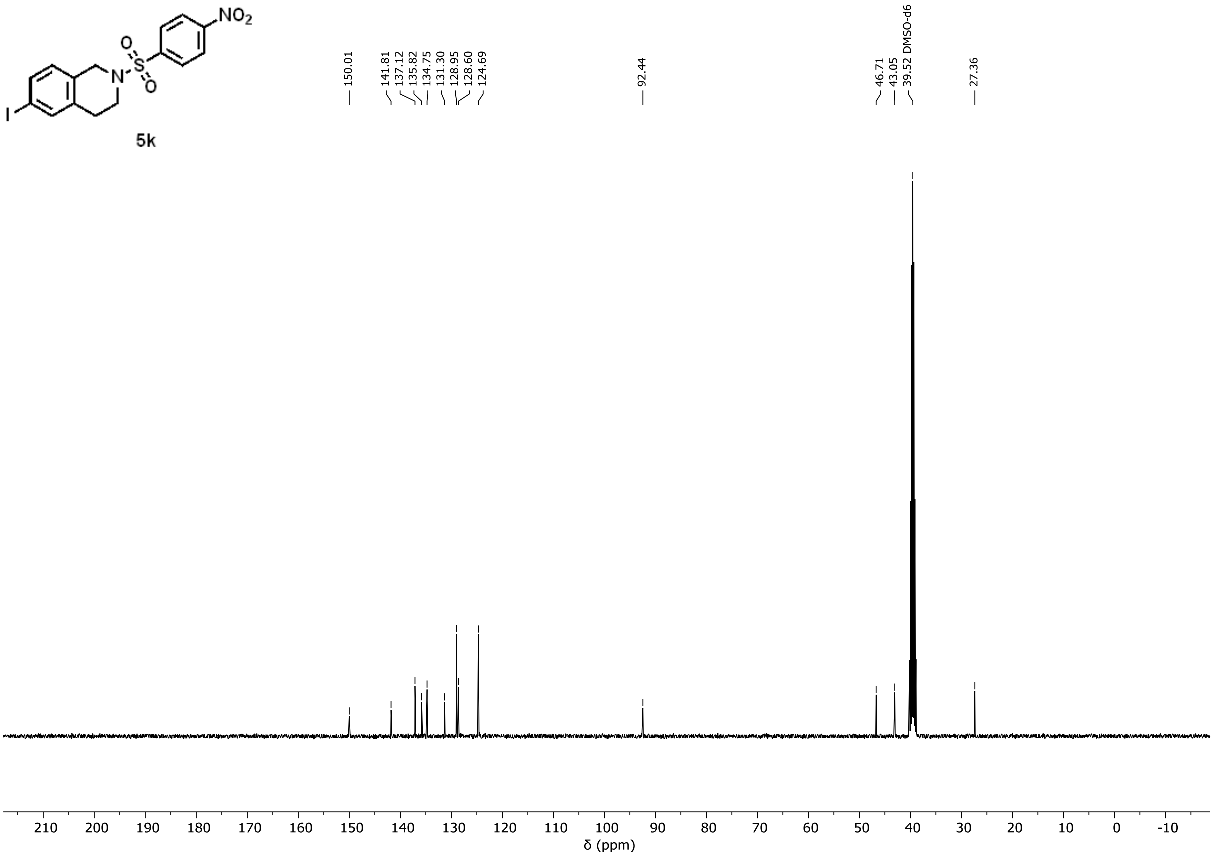

Figure **S99**: ^{13}C NMR (101 MHz, DMSO-d$_6$) of **5k**.

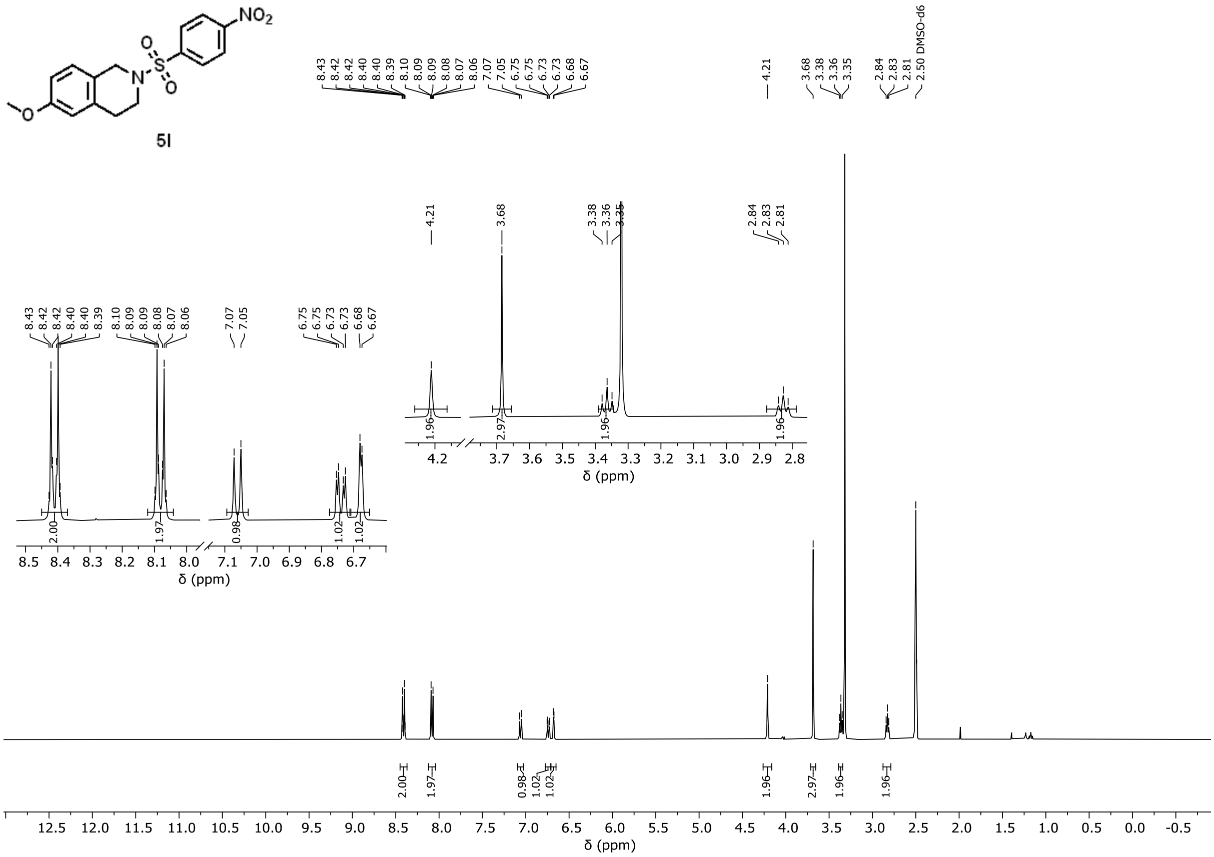

Figure **S100**: ^{1}H NMR (400 MHz, DMSO-d_6) of **5l**.

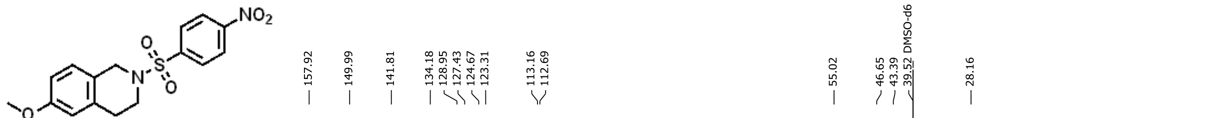

Figure **S101**: ^{13}C NMR (101 MHz, DMSO-d_6) of **5l**.

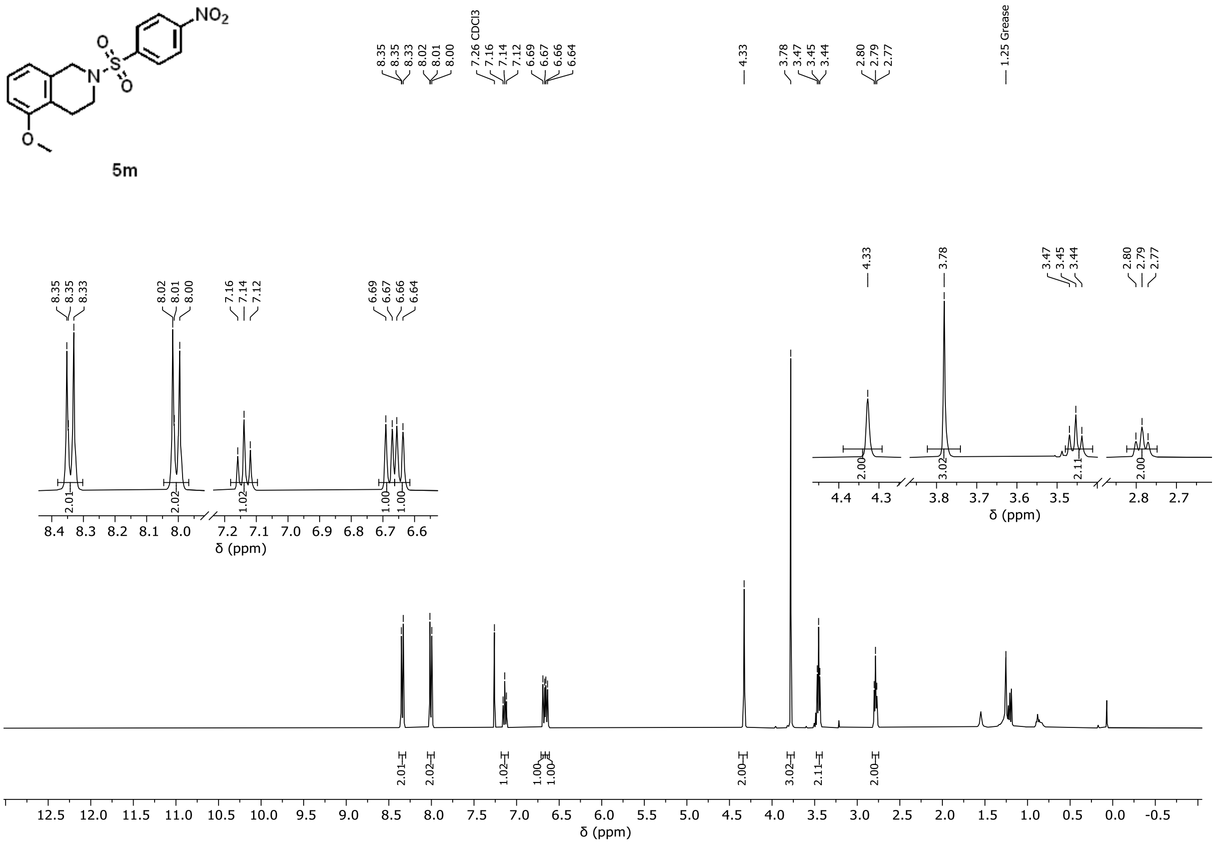

Figure **S102**: ^{1}H NMR (400 MHz, $CDCl_3$) of **5m**.

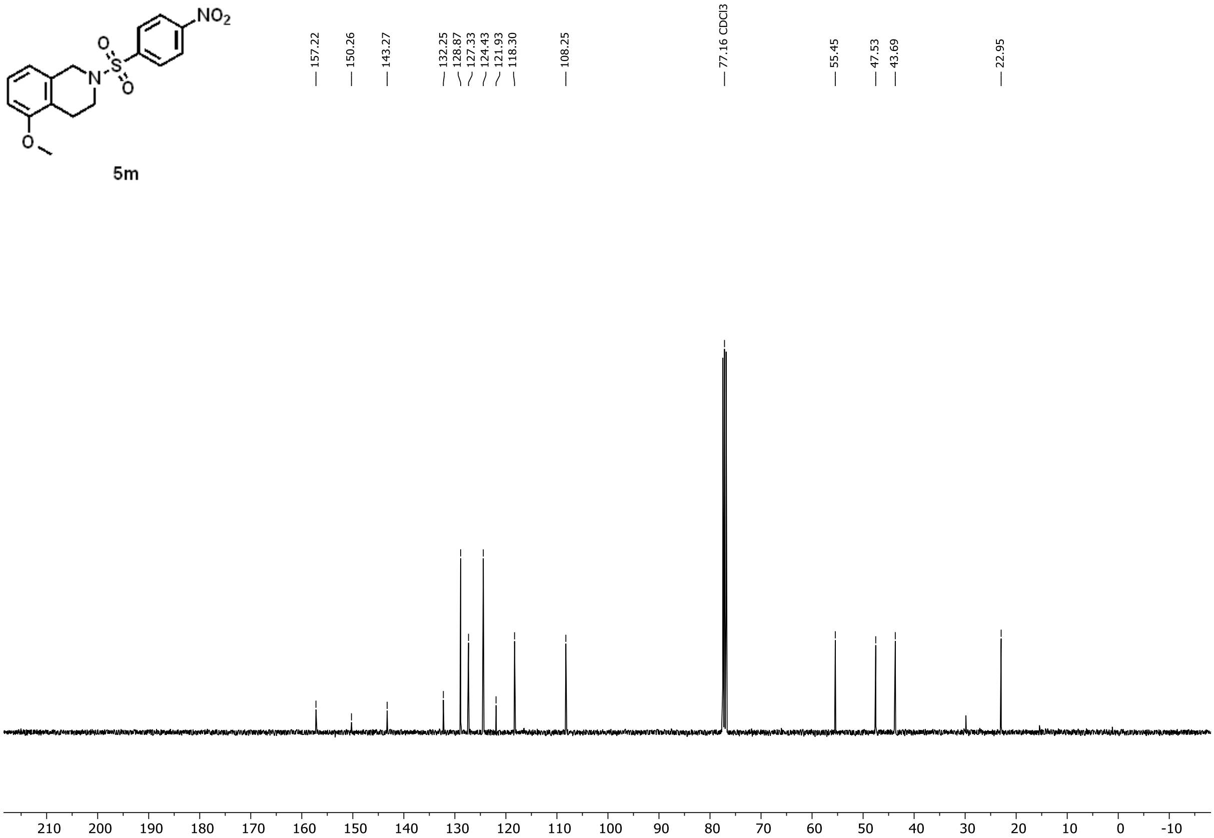

Figure **S103**: ^{13}C NMR (101 MHz, $CDCl_3$) of **5m**.

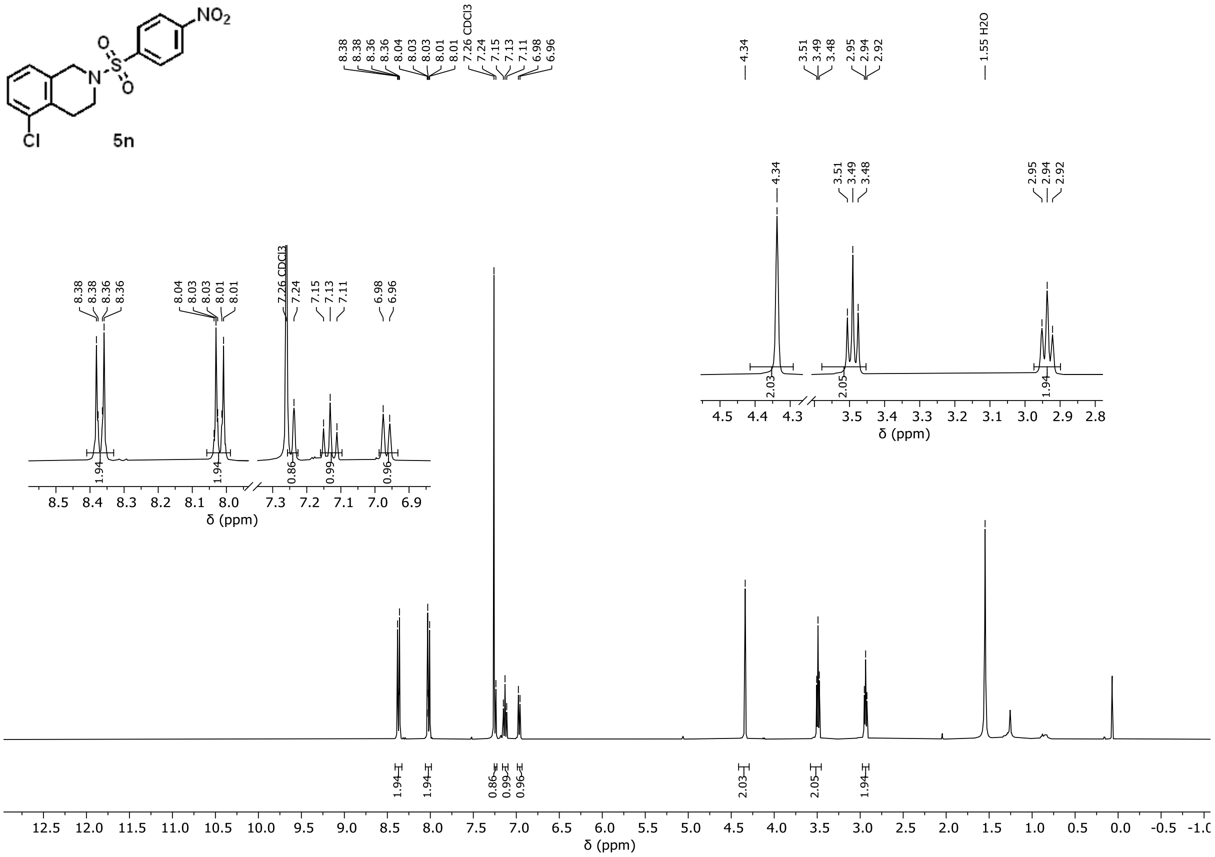

Figure **S104**: ^{1}H NMR (400 MHz, $CDCl_3$) of **5n**.

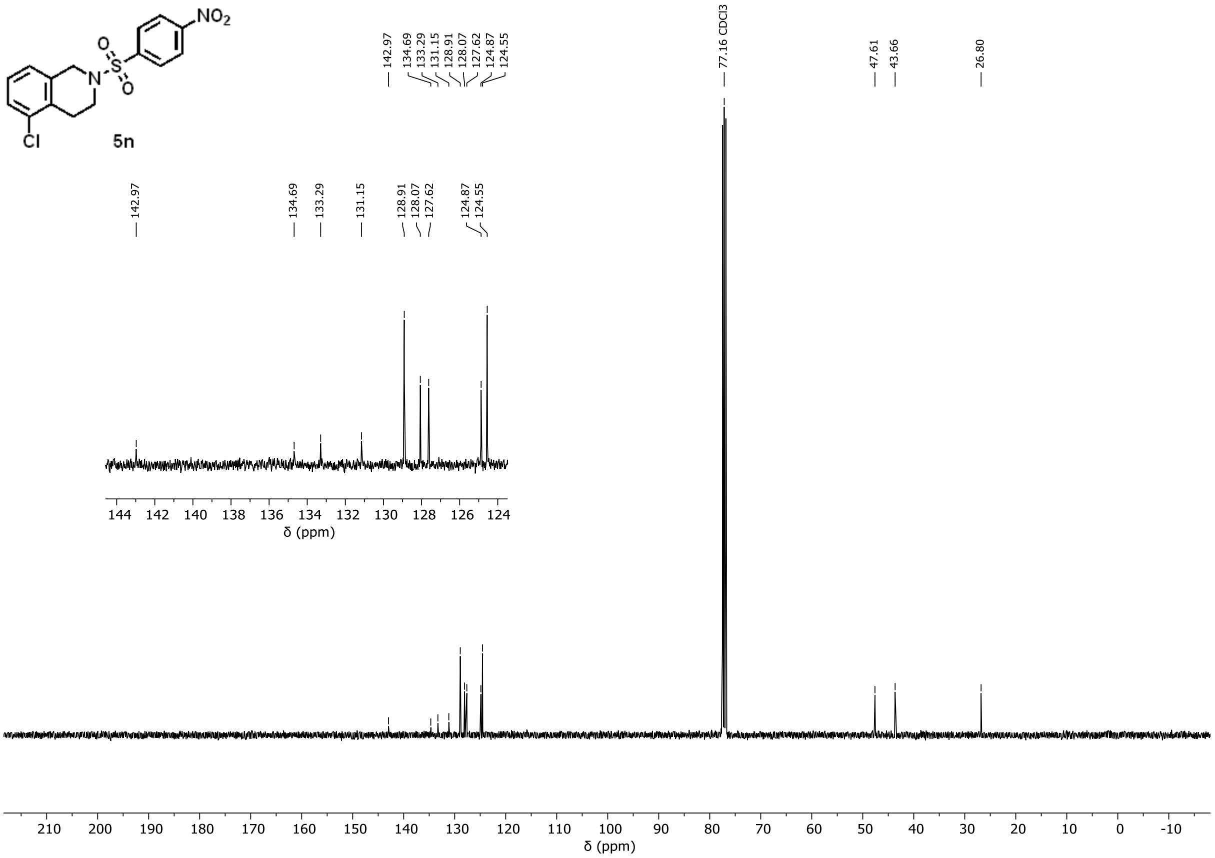

Figure **S105**: ^{13}C NMR (101 MHz, $CDCl_3$) of **5n**.

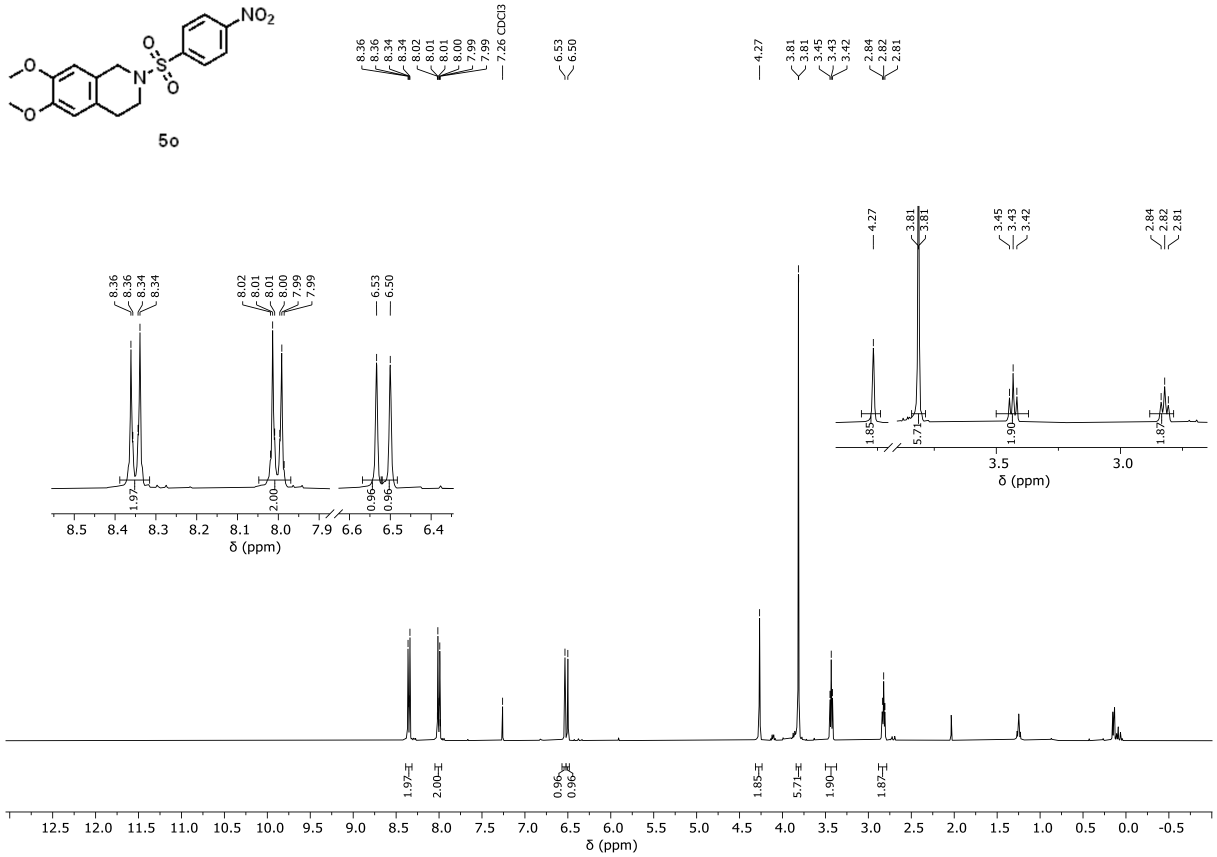

Figure **S106**: ^{1}H NMR (400 MHz, $CDCl_3$) of **5o**.

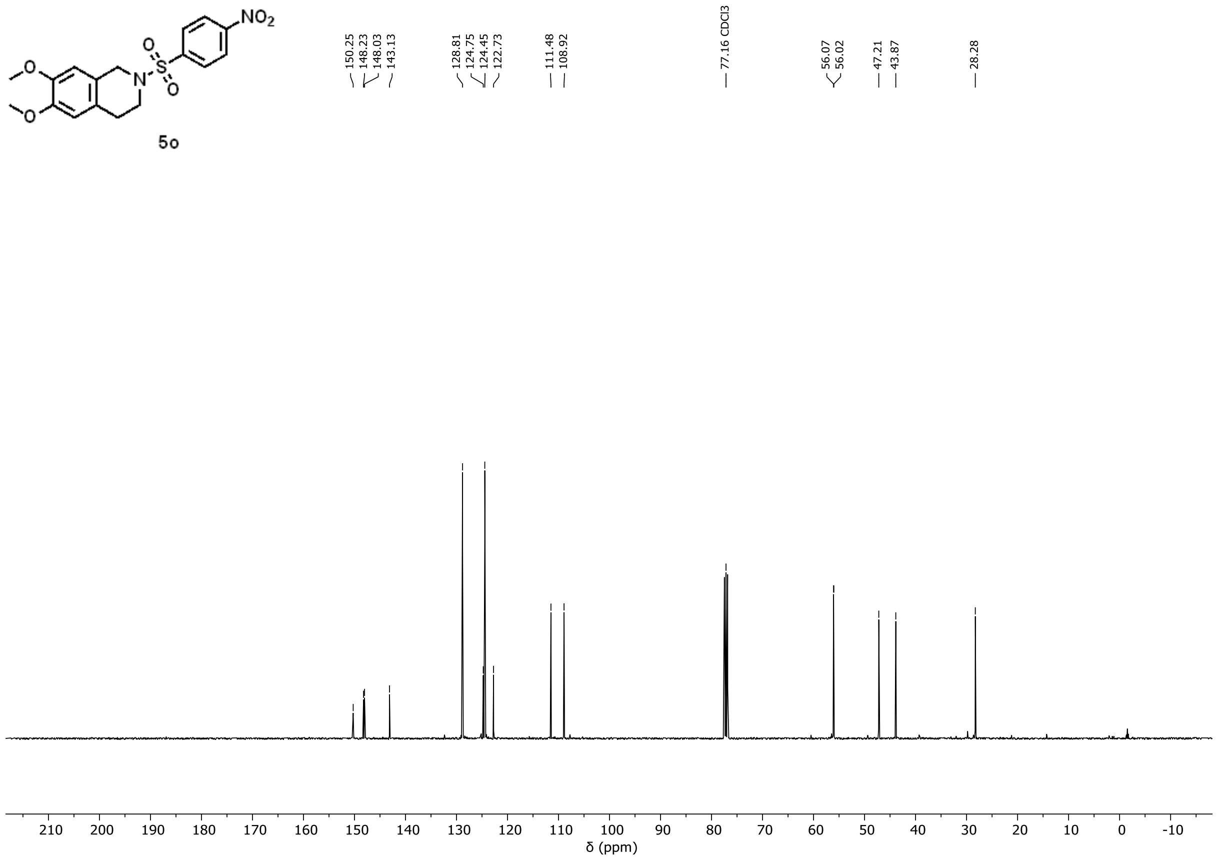

Figure **S107**: ^{13}C NMR (101 MHz, $CDCl_3$) of **5o**.

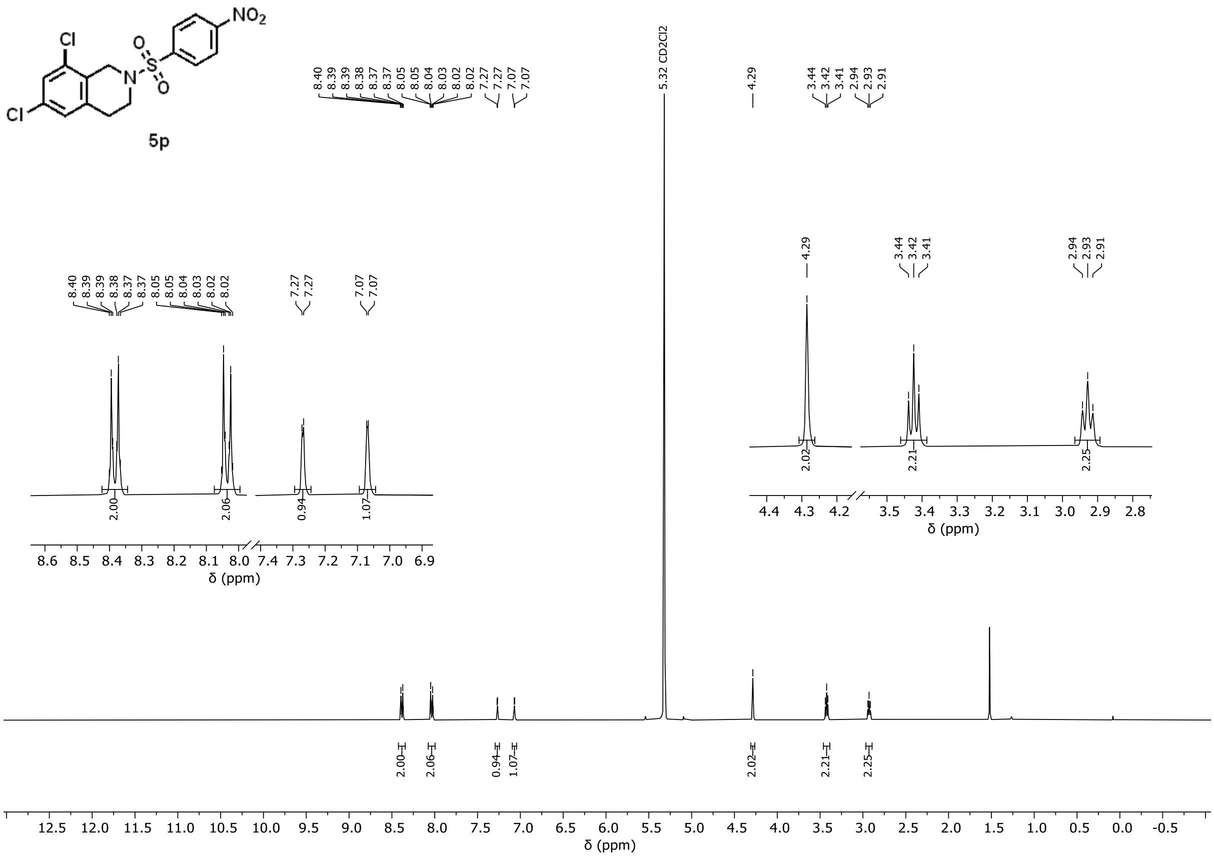

Figure **S108**: ^{1}H NMR (400 MHz, CD_2Cl_2) of **5p**.

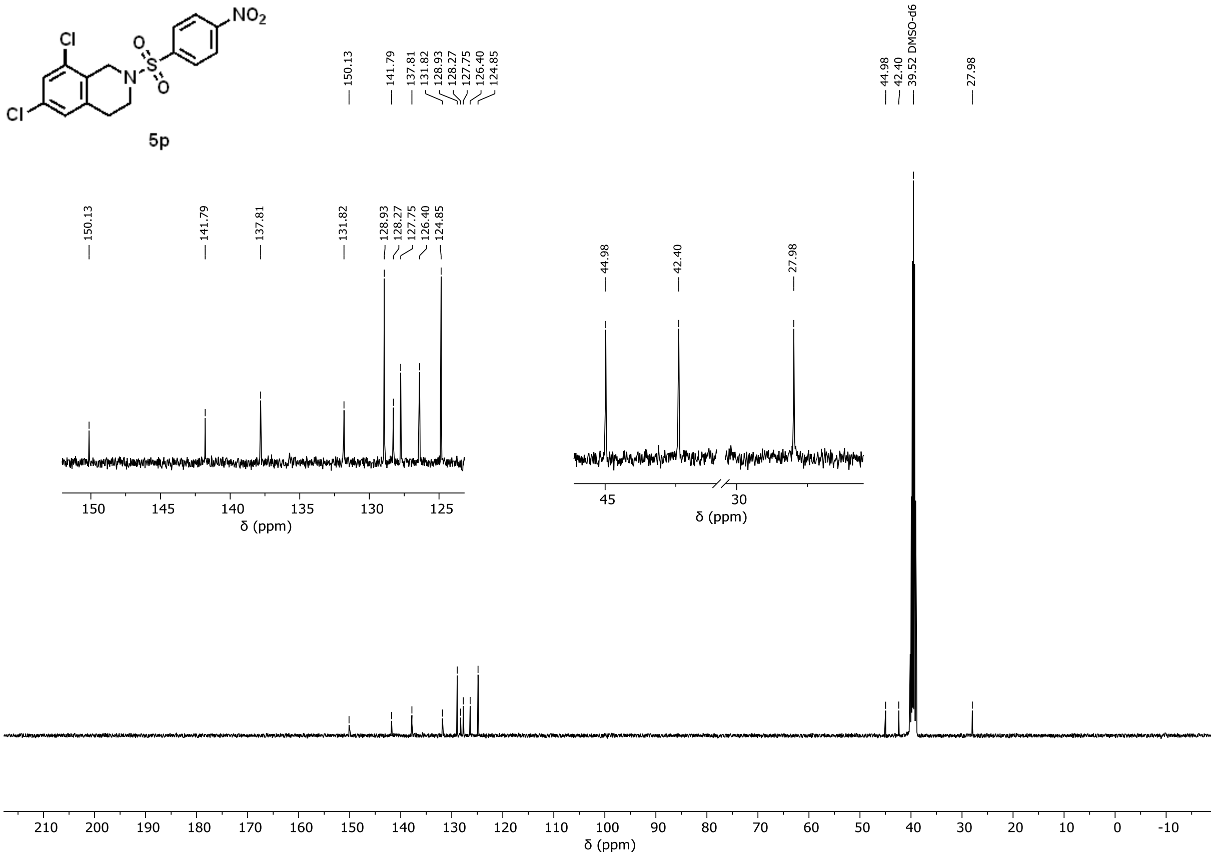

Figure **S109**: ^{13}C NMR (101 MHz, DMSO-d_6) of **5p**.

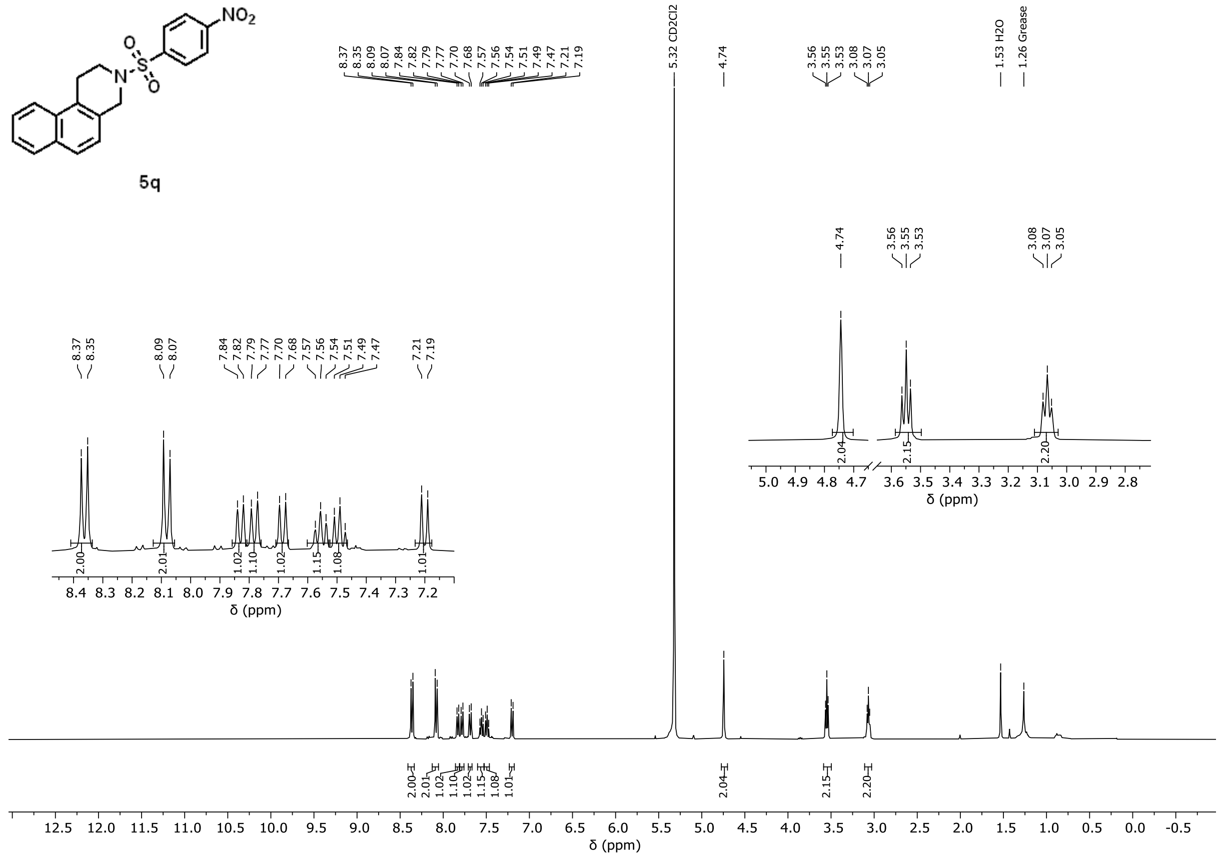

Figure **S110**: ^{1}H NMR (400 MHz, CD_2Cl_2) of **5q**.

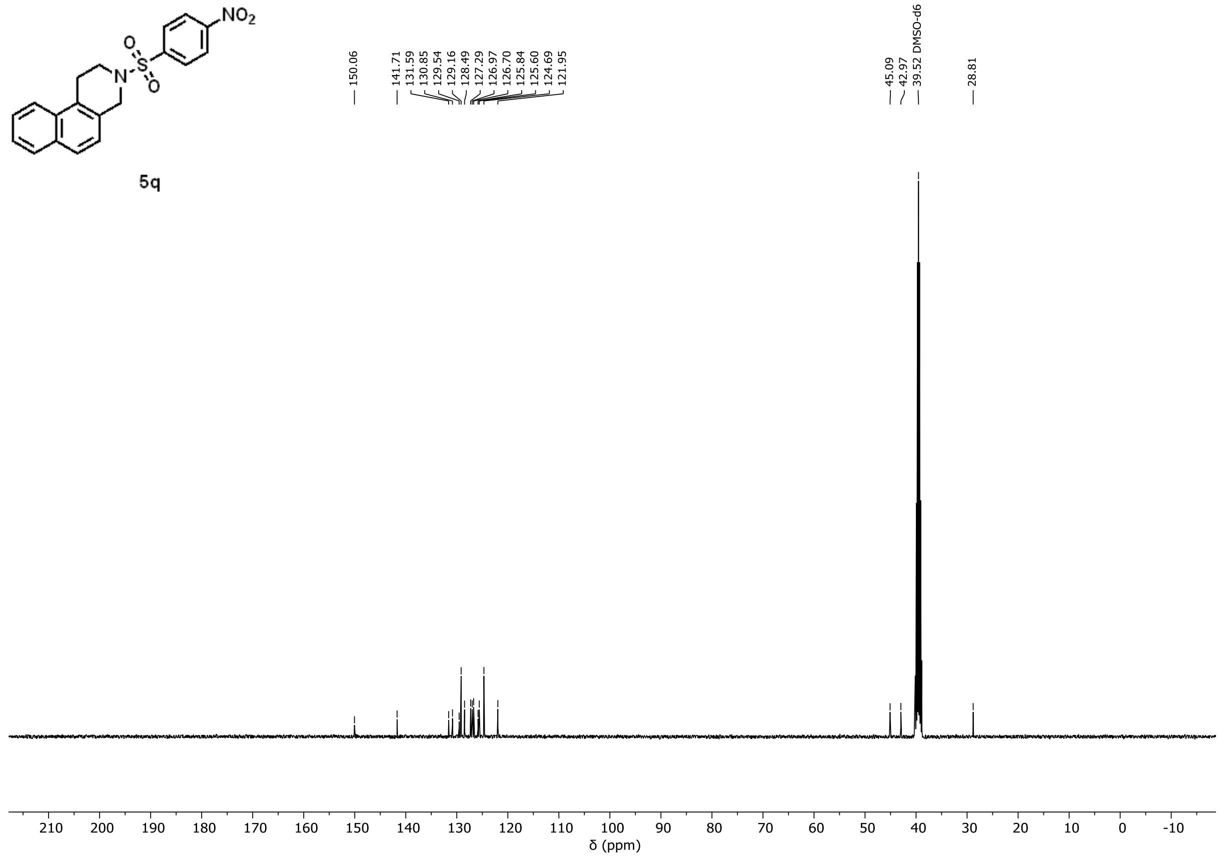

Figure **S111**: ^{13}C NMR (101 MHz, DMSO-d_6) of **5q**.

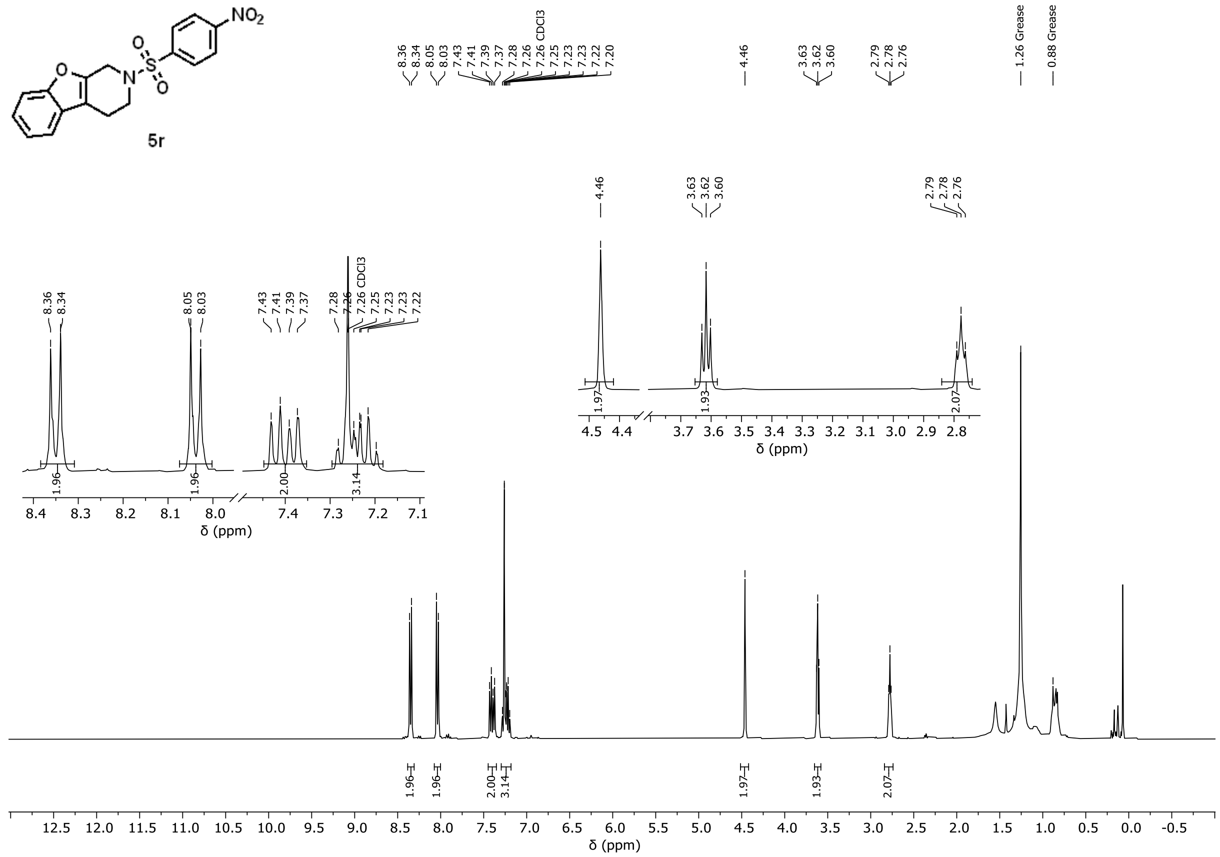

Figure **S112**: ^{1}H NMR (400 MHz, $CDCl_3$) of **5r**.

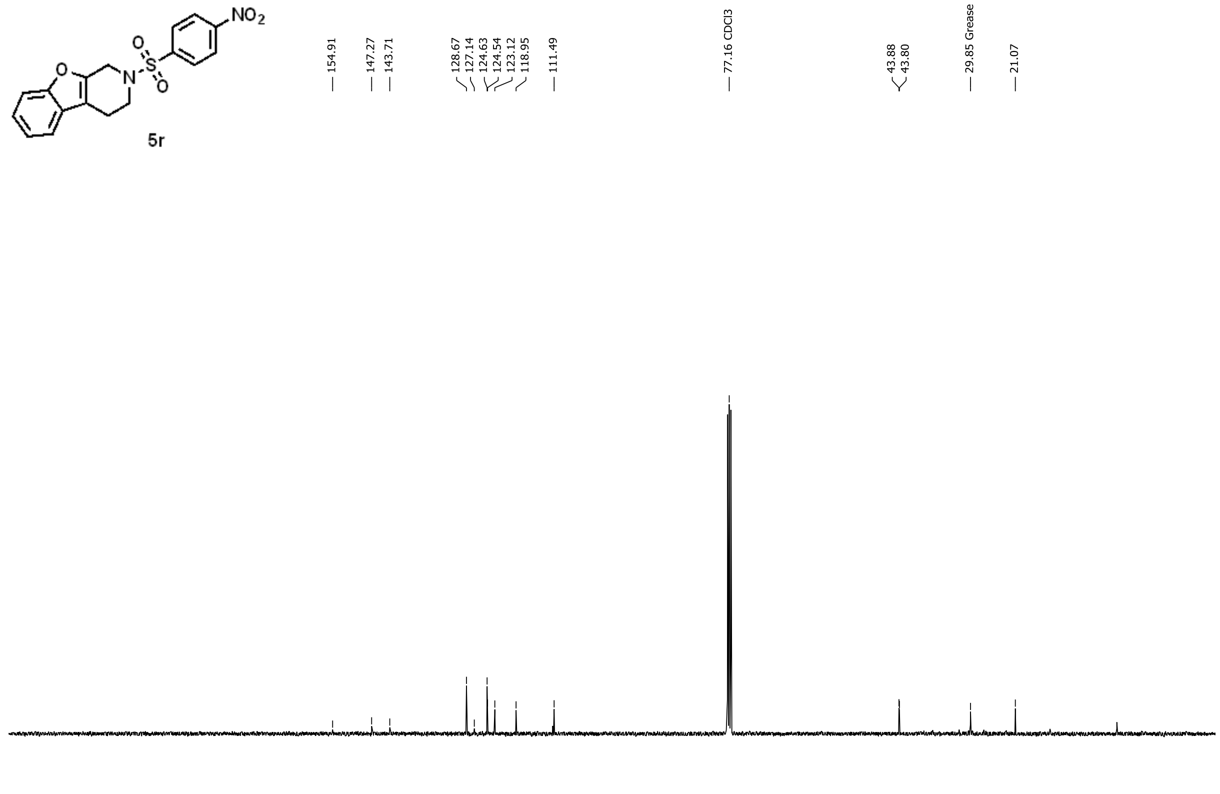

Figure **S113**: ^{13}C NMR (101 MHz, $CDCl_3$) of **5r**.

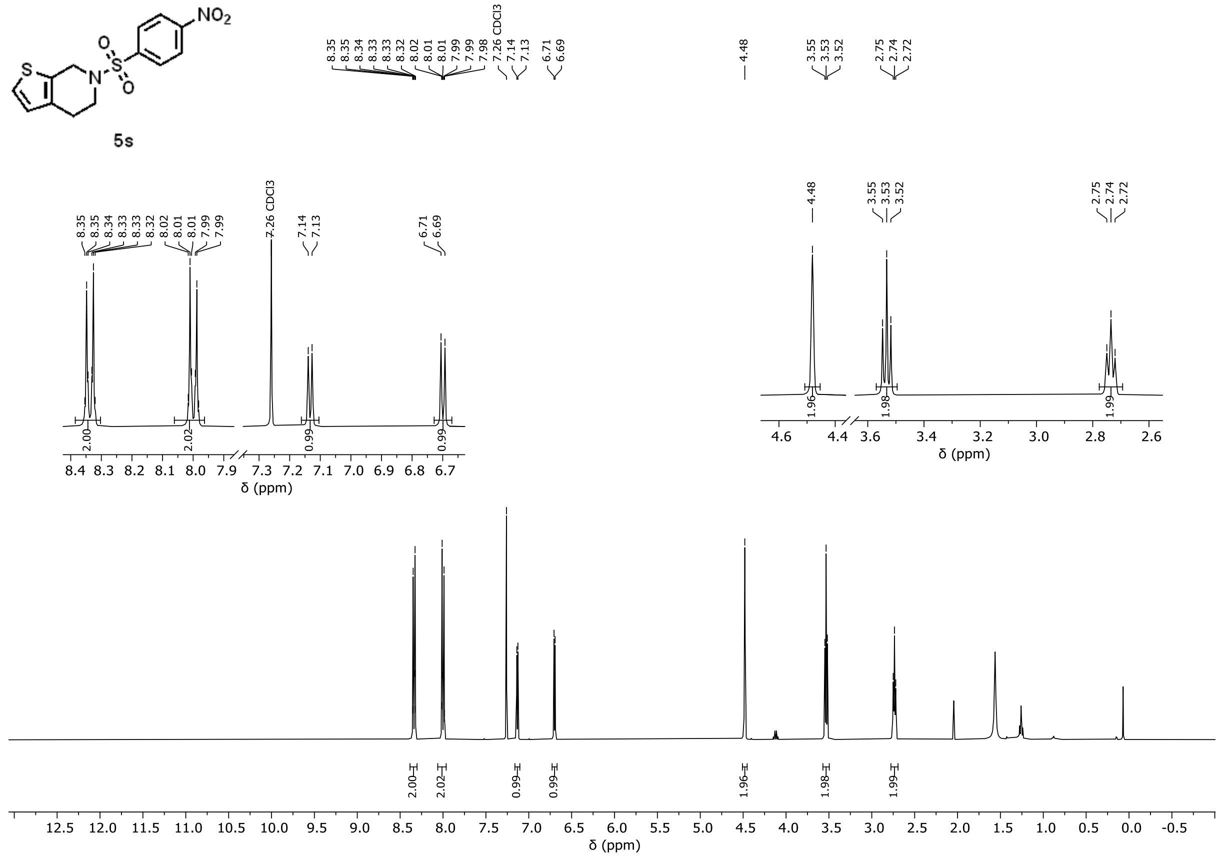

Figure **S114**: ^{1}H NMR (400 MHz, $CDCl_3$) of **5s**.

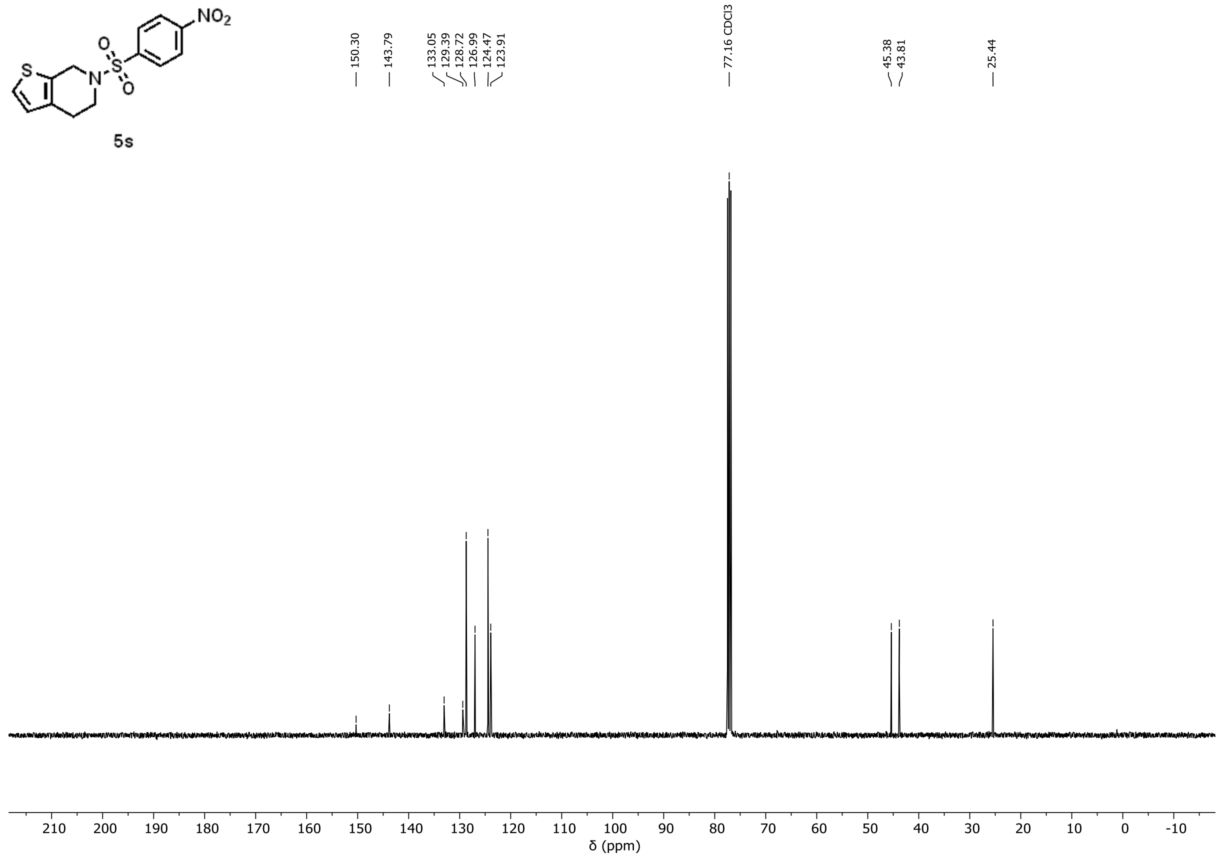

Figure **S115**: ^{13}C NMR (101 MHz, $CDCl_3$) of **5s**.

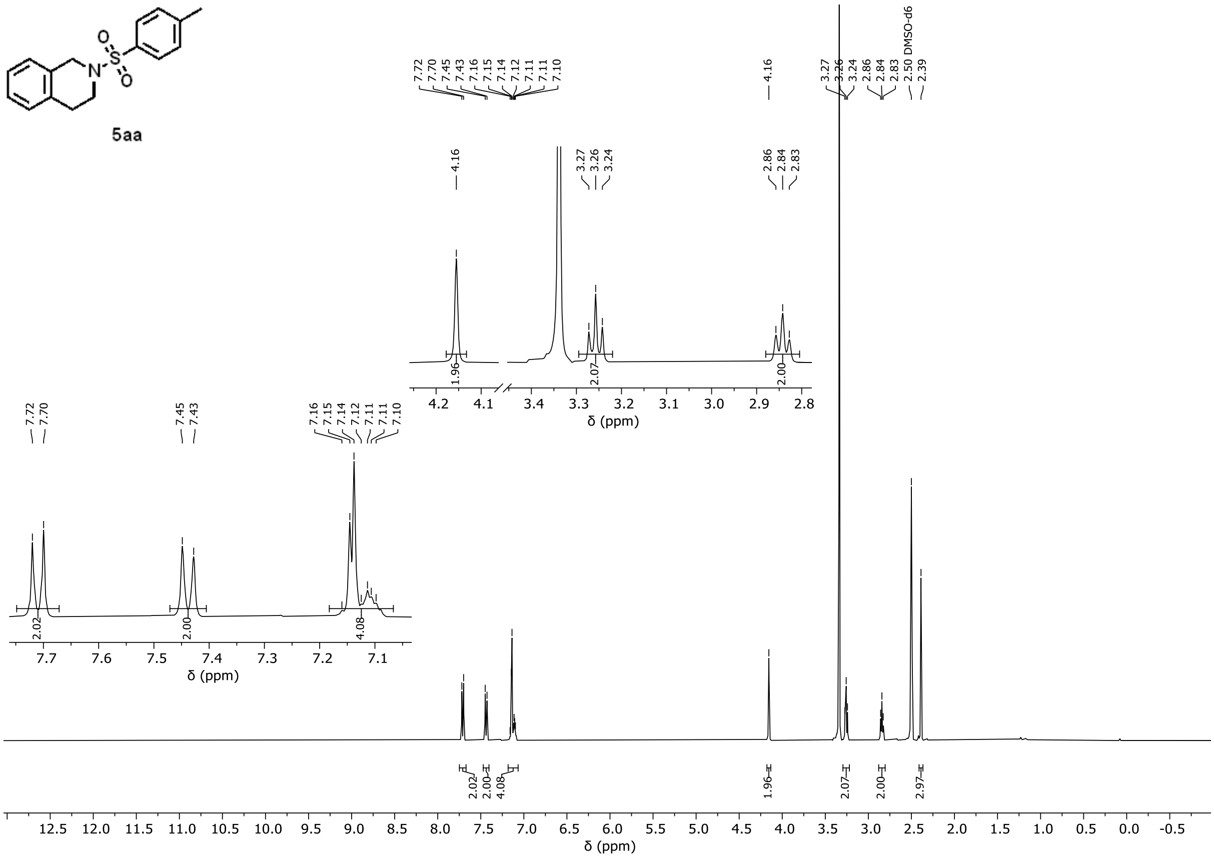

Figure **S116**: ^{1}H NMR (400 MHz, DMSO-d_6) of **5aa**.

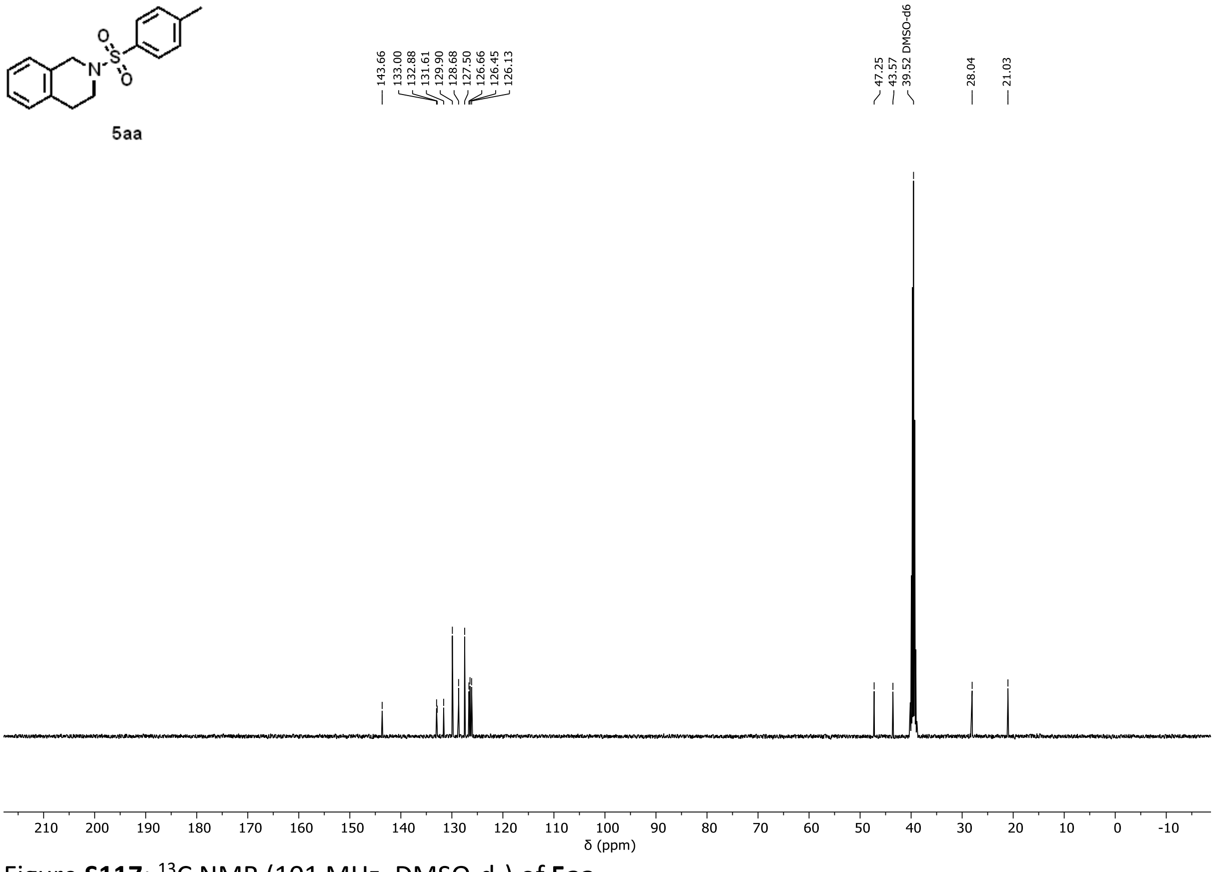

Figure **S117**: ^{13}C NMR (101 MHz, DMSO-d_6) of **5aa**.

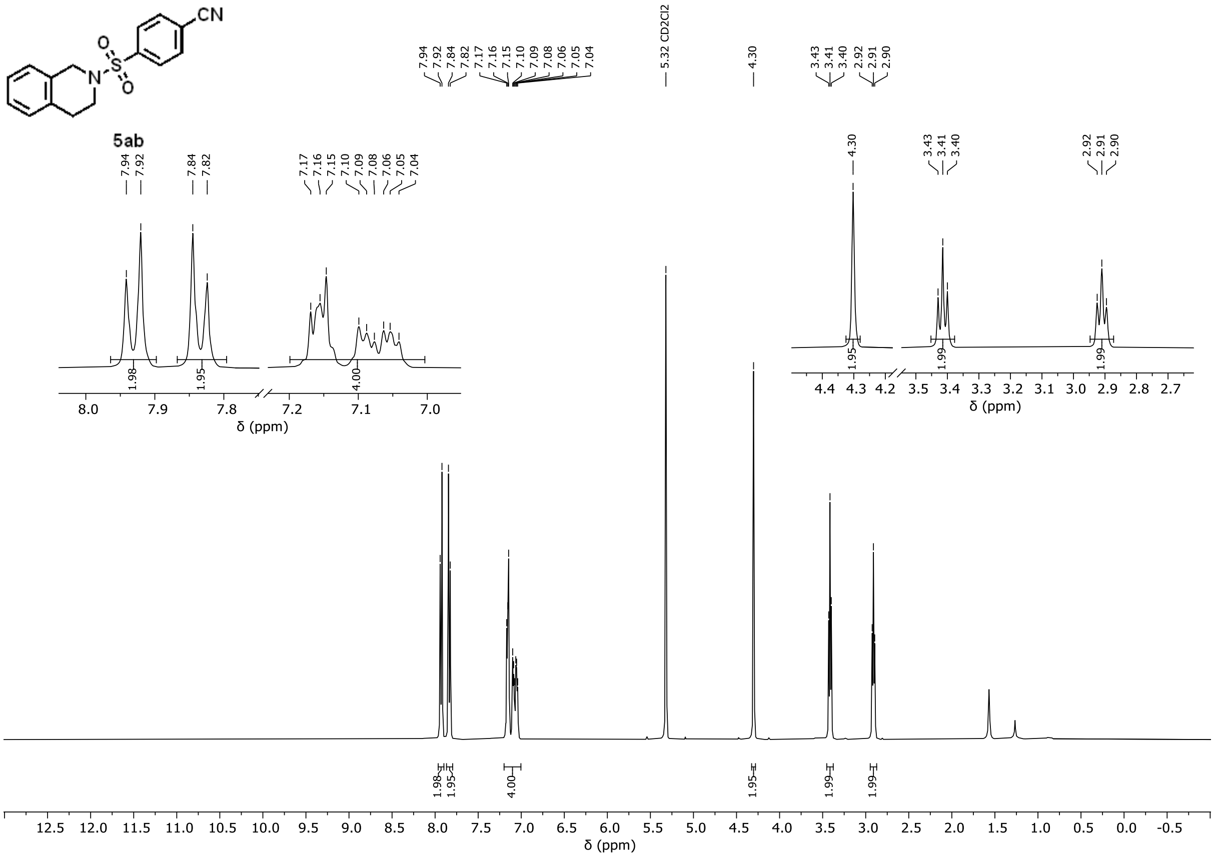

Figure **S118**: ^{1}H NMR (400 MHz, CD_2Cl_2) of **5ab**.

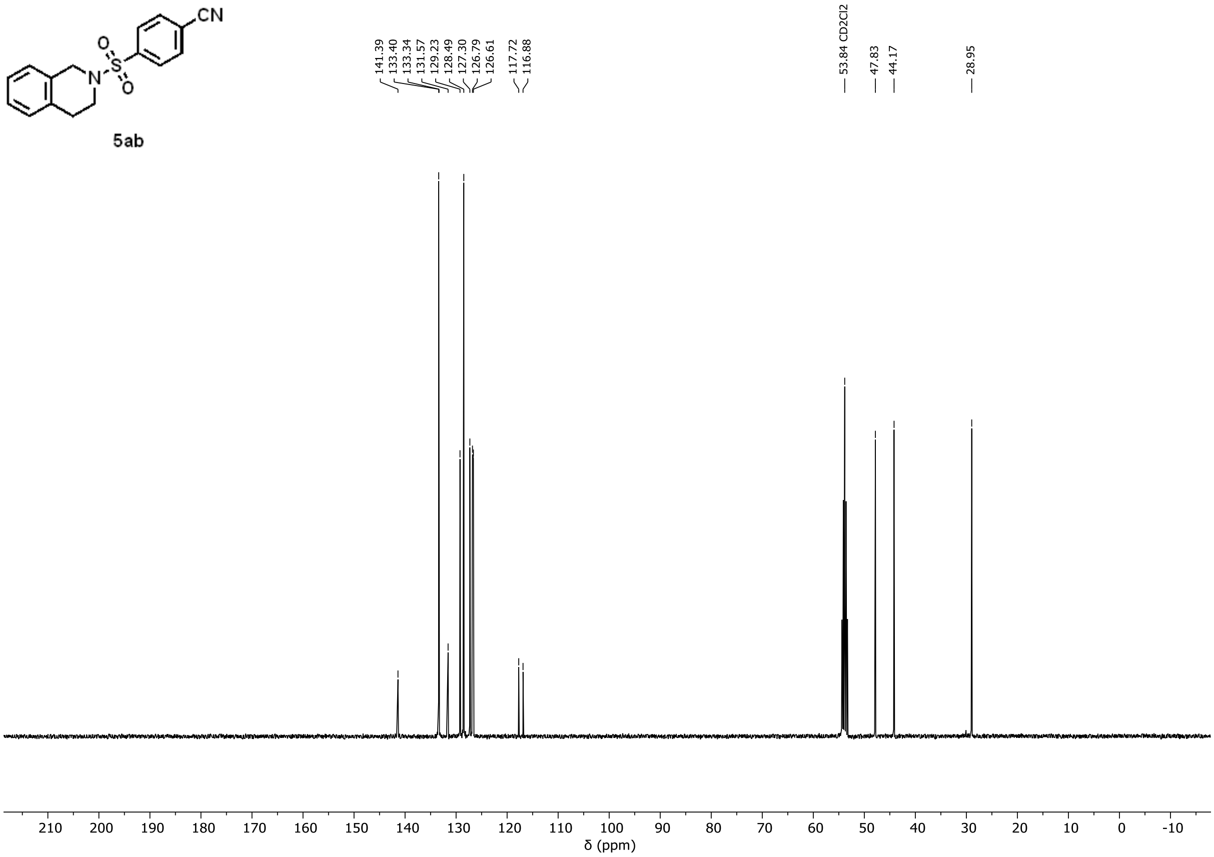

Figure **S119**: ^{13}C NMR (101 MHz, CD_2Cl_2) of **5ab**.

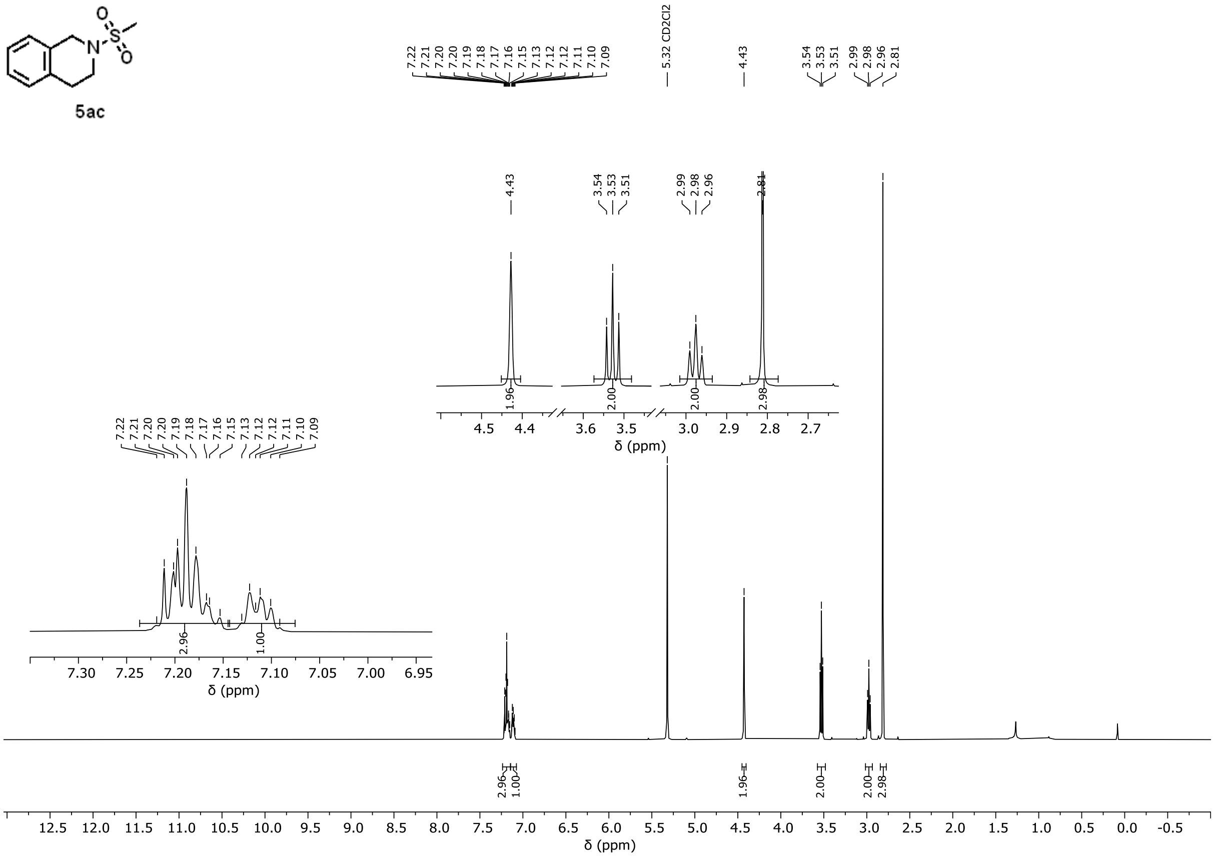

Figure **S120**: ^{1}H NMR (400 MHz, CD_2Cl_2) of **5ac**.

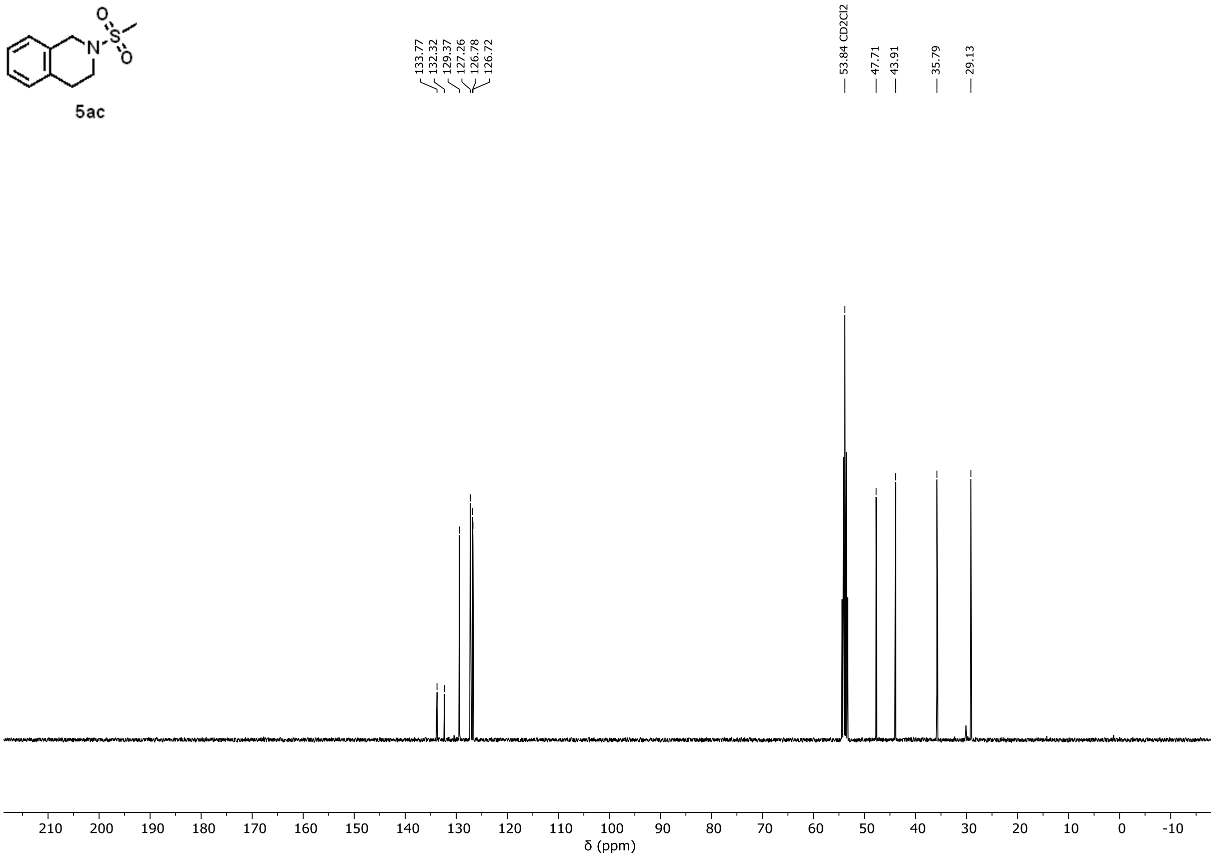

Figure **S121**: ^{13}C NMR (101 MHz, CD_2Cl_2) of **5ac**.

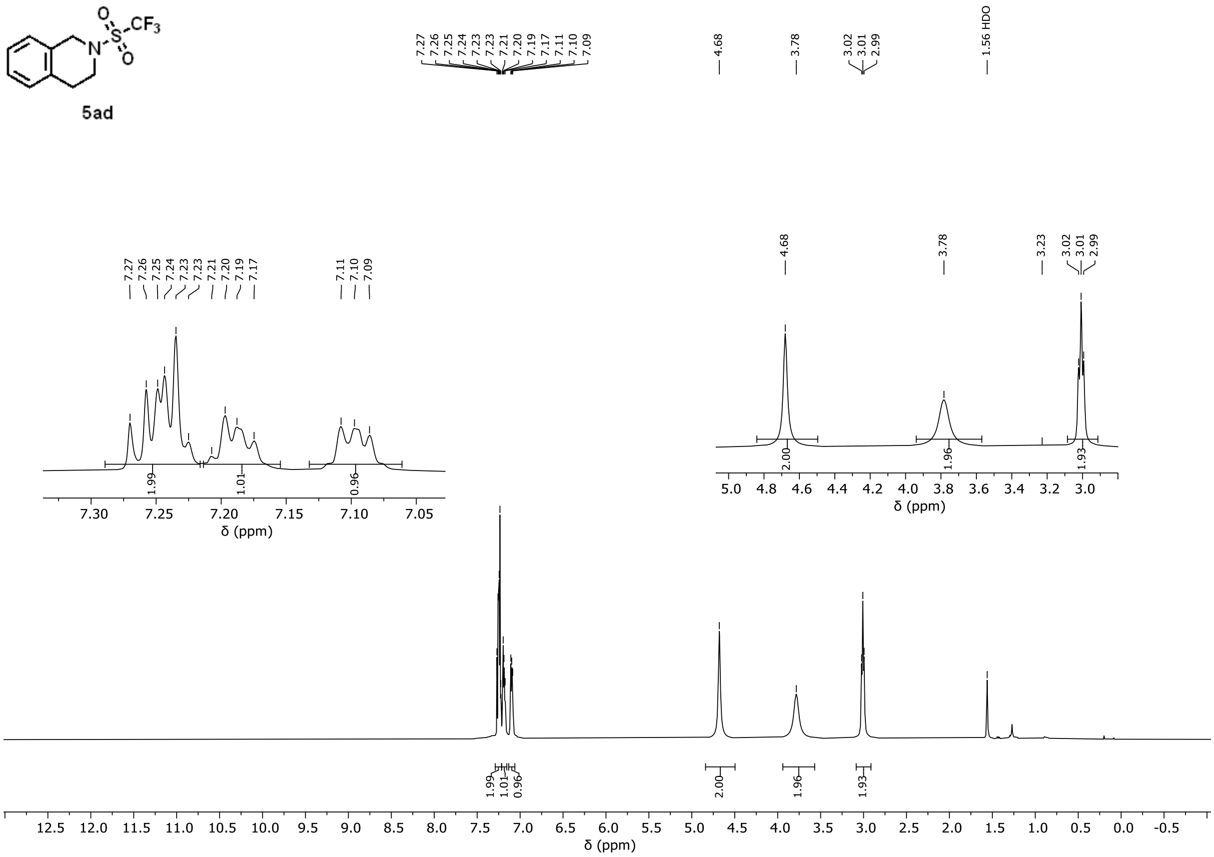

Figure **S122**: ^{1}H NMR (400 MHz, $CDCl_3$) of **5ad**.

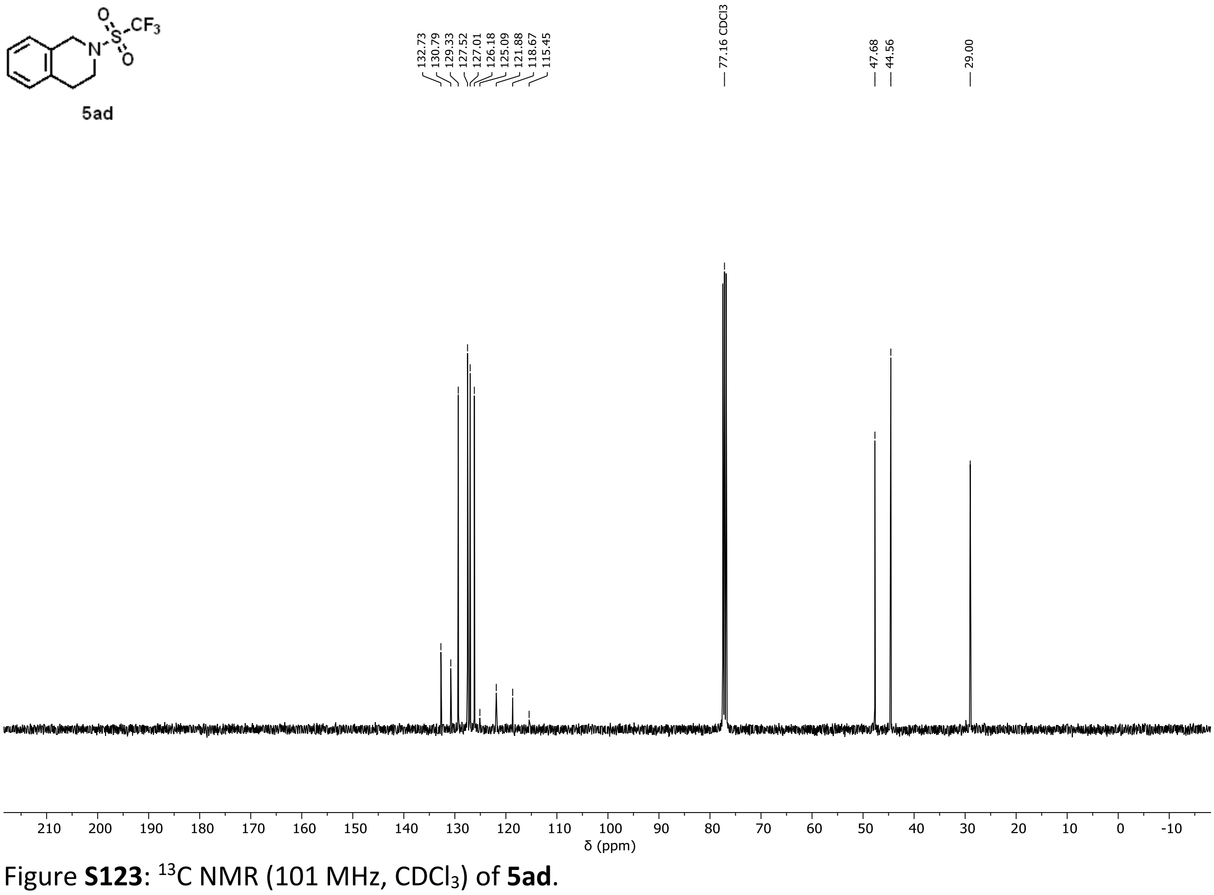

Figure **S123**: ^{13}C NMR (101 MHz, $CDCl_3$) of **5ad**.

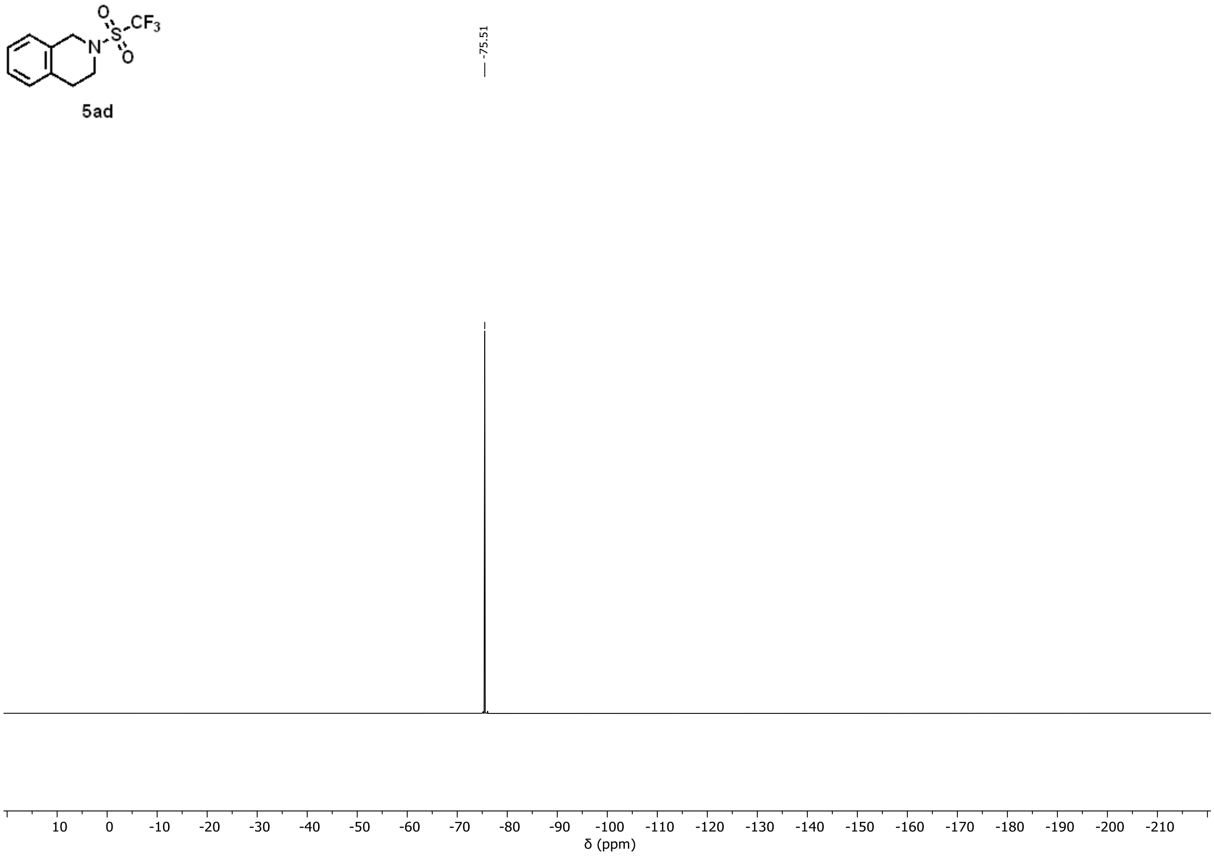

Figure **S124**: ^{19}F NMR (376 MHz, $CDCl_3$) of **5ad**.

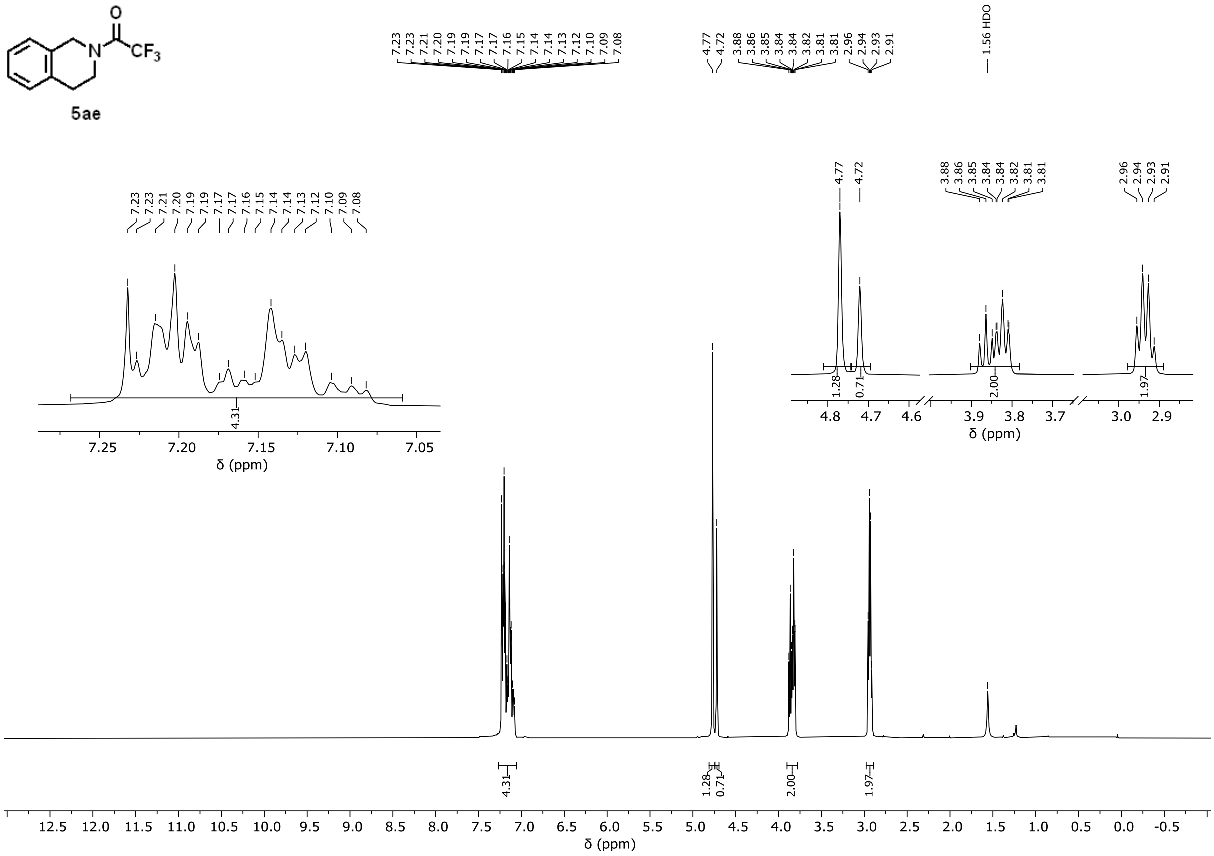

Figure **S125**: ^{1}H NMR (400 MHz, $CDCl_3$) of **5ae**.

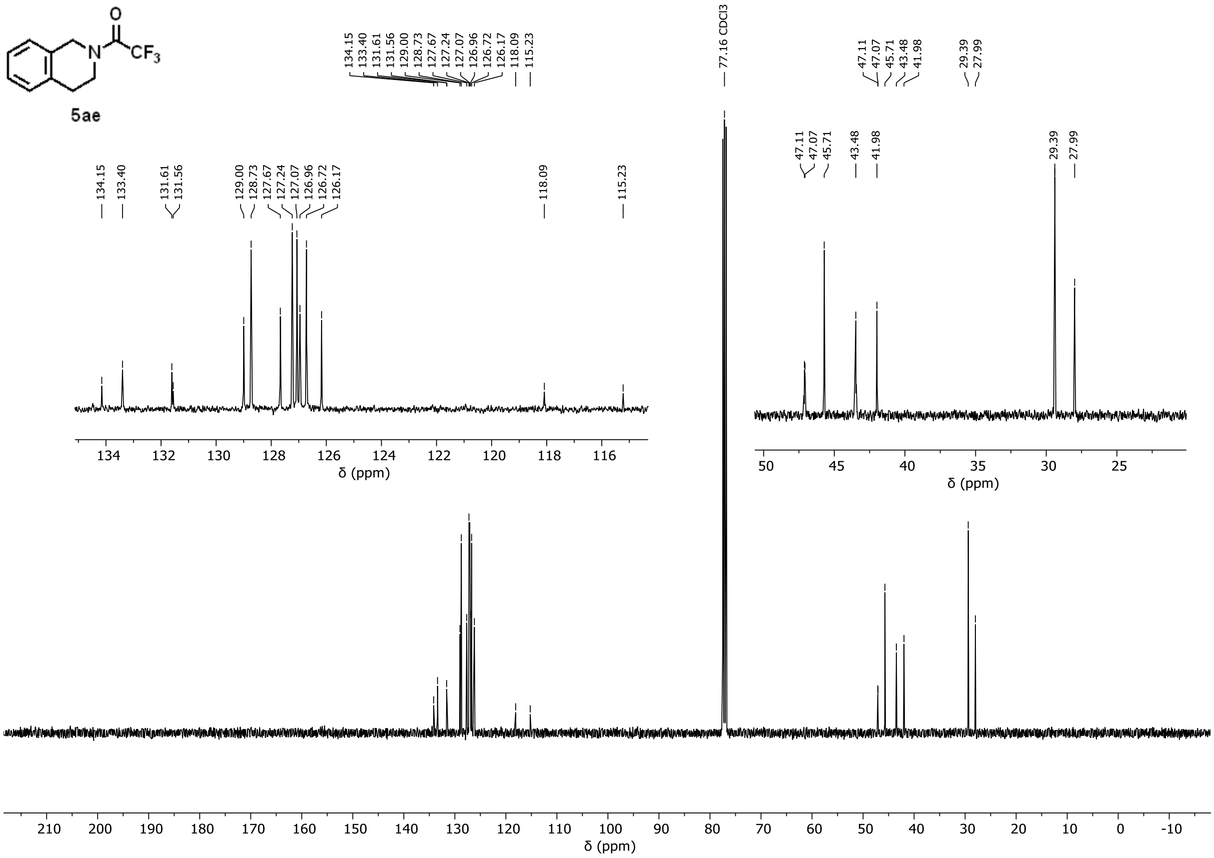

Figure **S126**: ^{13}C NMR (101 MHz, $CDCl_3$) of **5ae**.

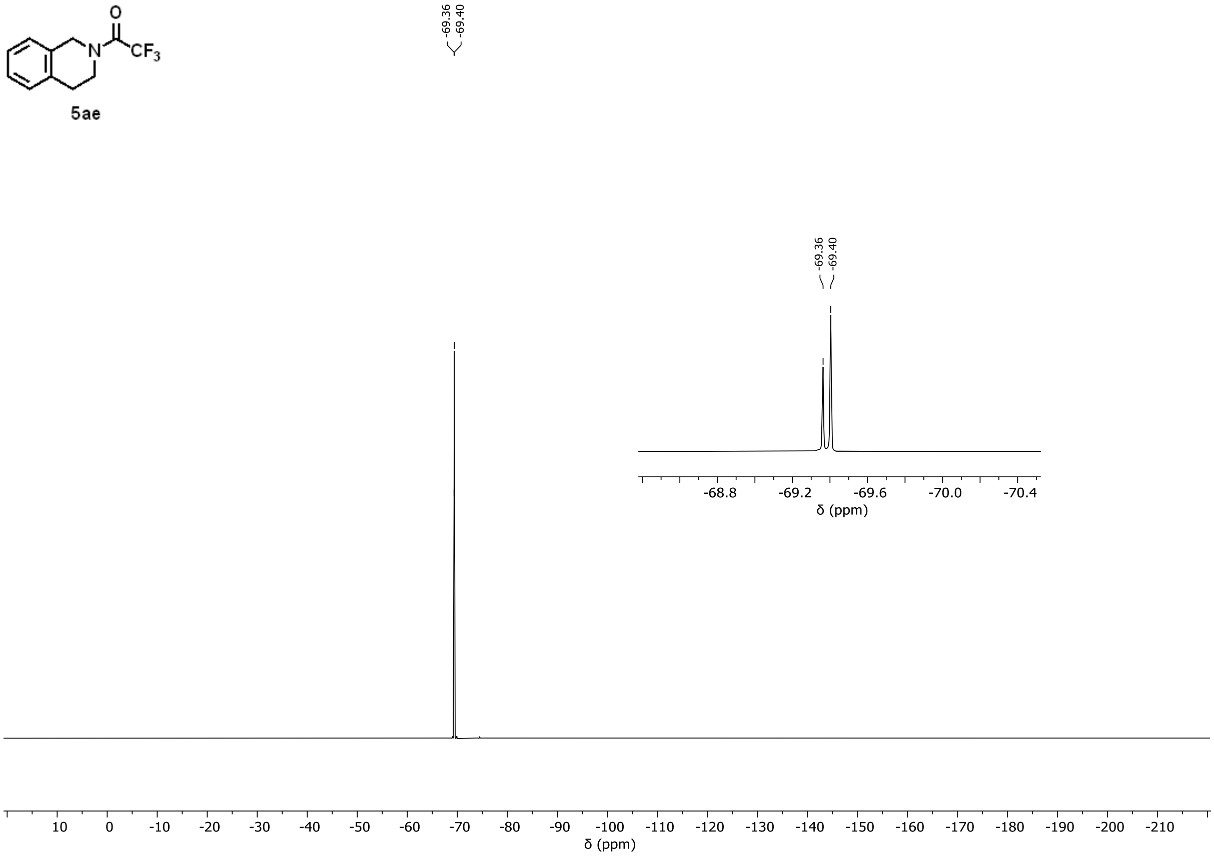

Figure **S127**: ^{19}F NMR (376 MHz, $CDCl_3$) of **5ae**.

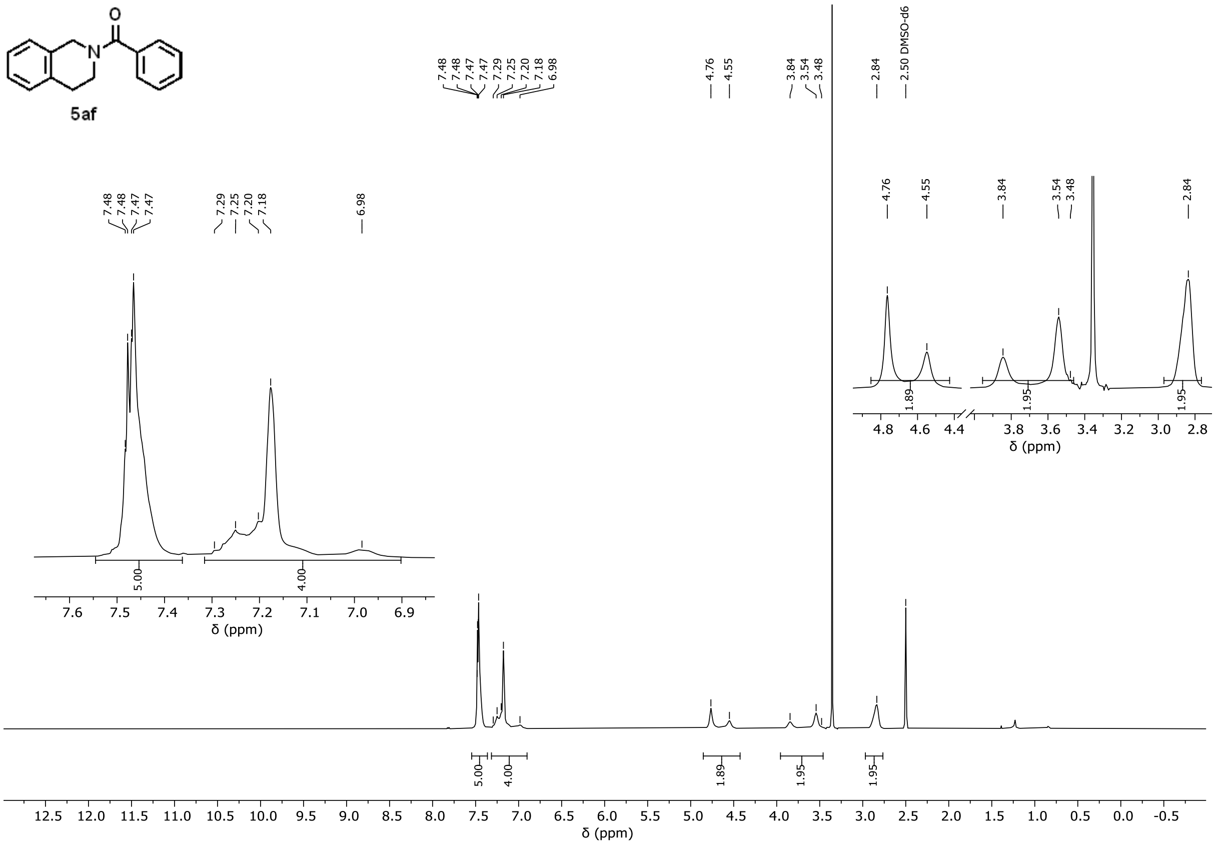

Figure **S128**: ^{1}H NMR (400 MHz, DMSO-d_6) of **5af**.

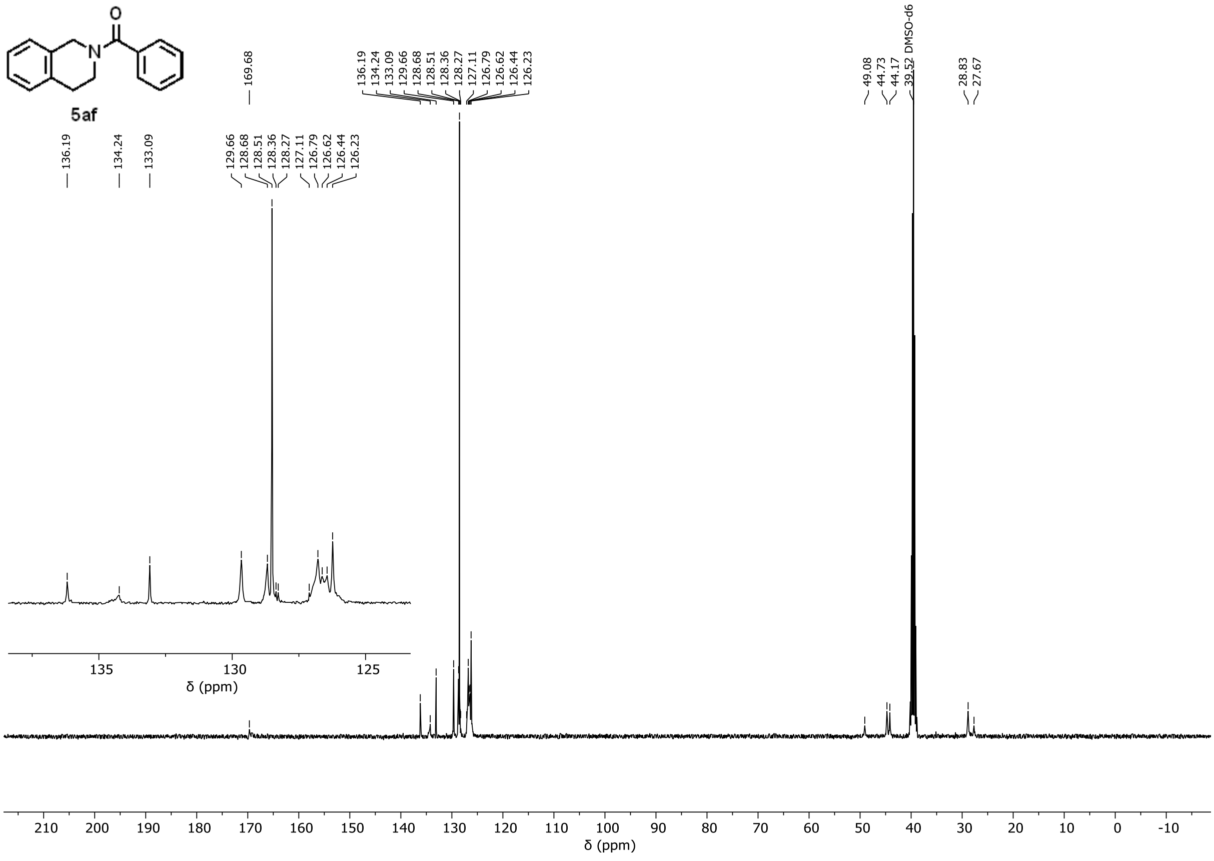

Figure **S129**: ^{13}C NMR (101 MHz, DMSO-d_6) of **5af**.

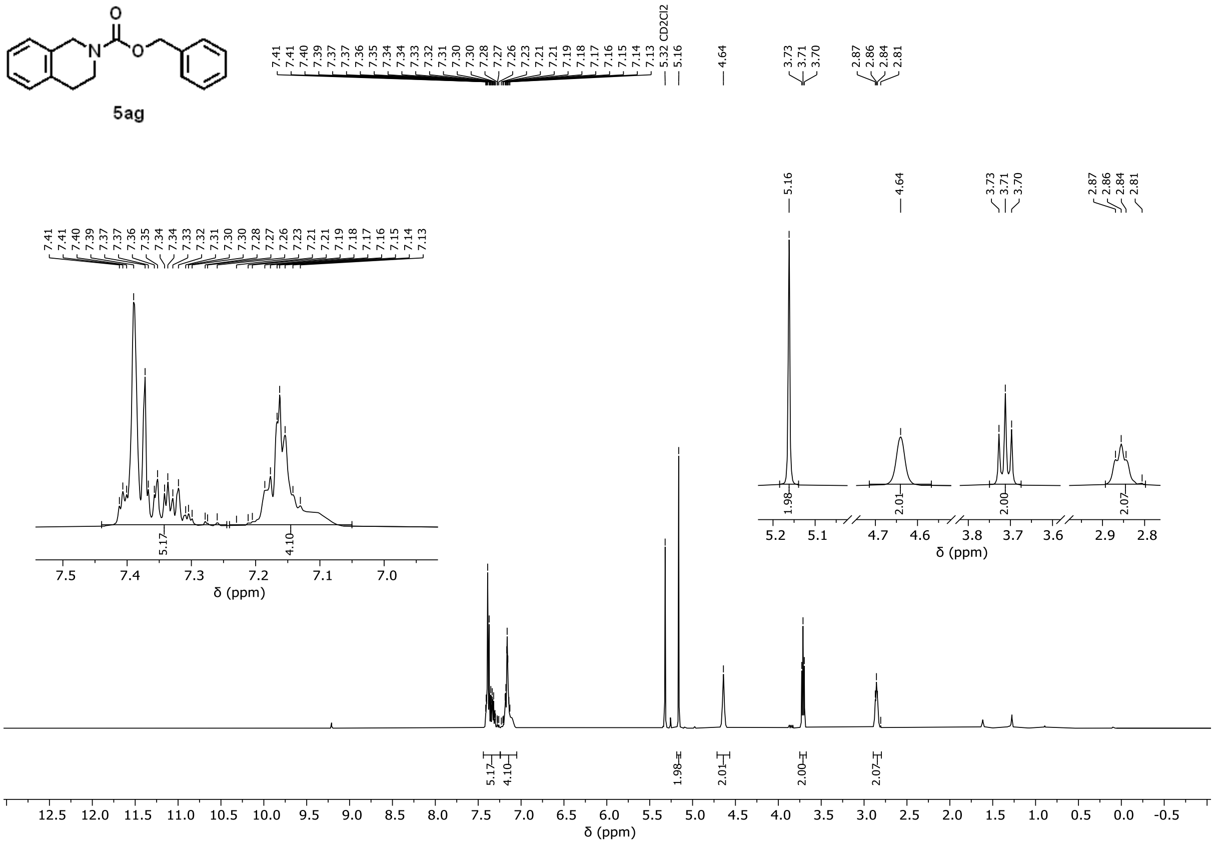

Figure **S130**: ^{1}H NMR (400 MHz, CD_2Cl_2) of **5ag**.

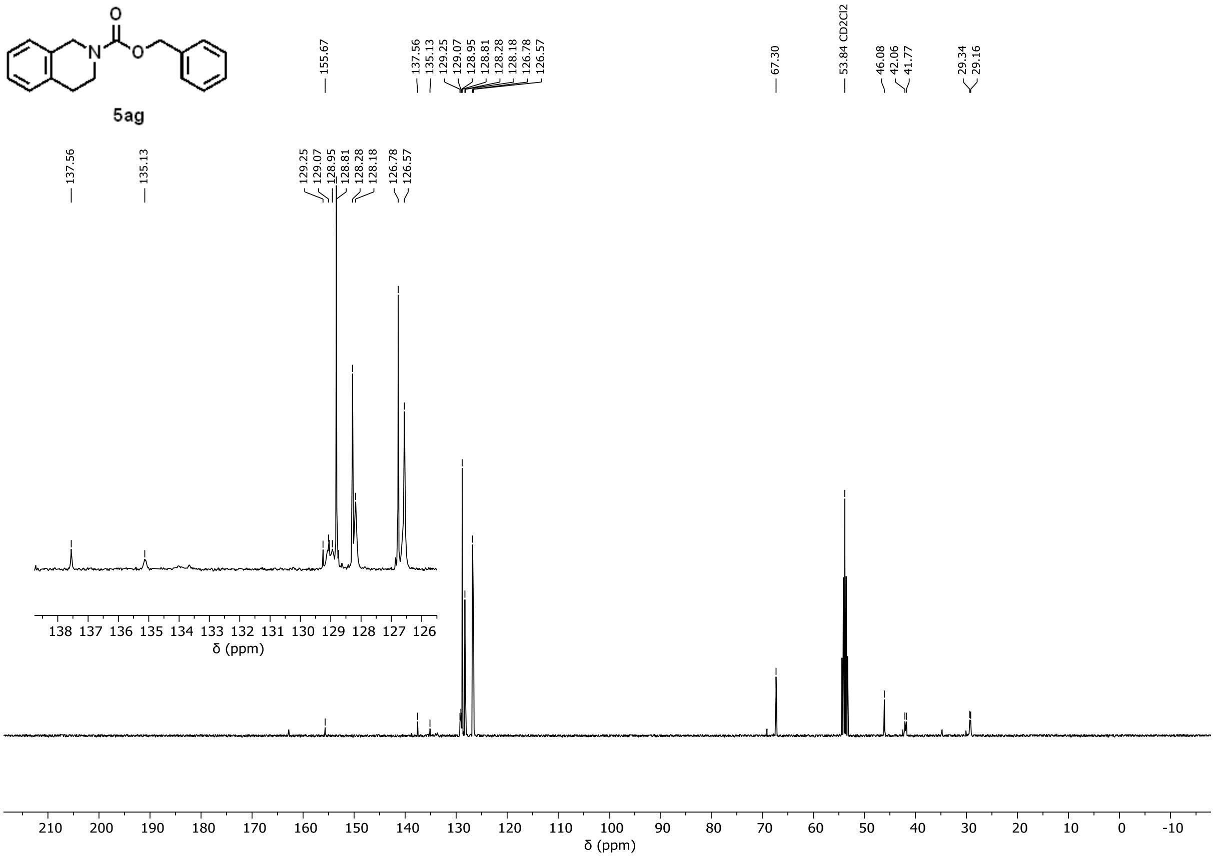

Figure **S131**: ^{13}C NMR (101 MHz, CD_2Cl_2) of **5ag**.

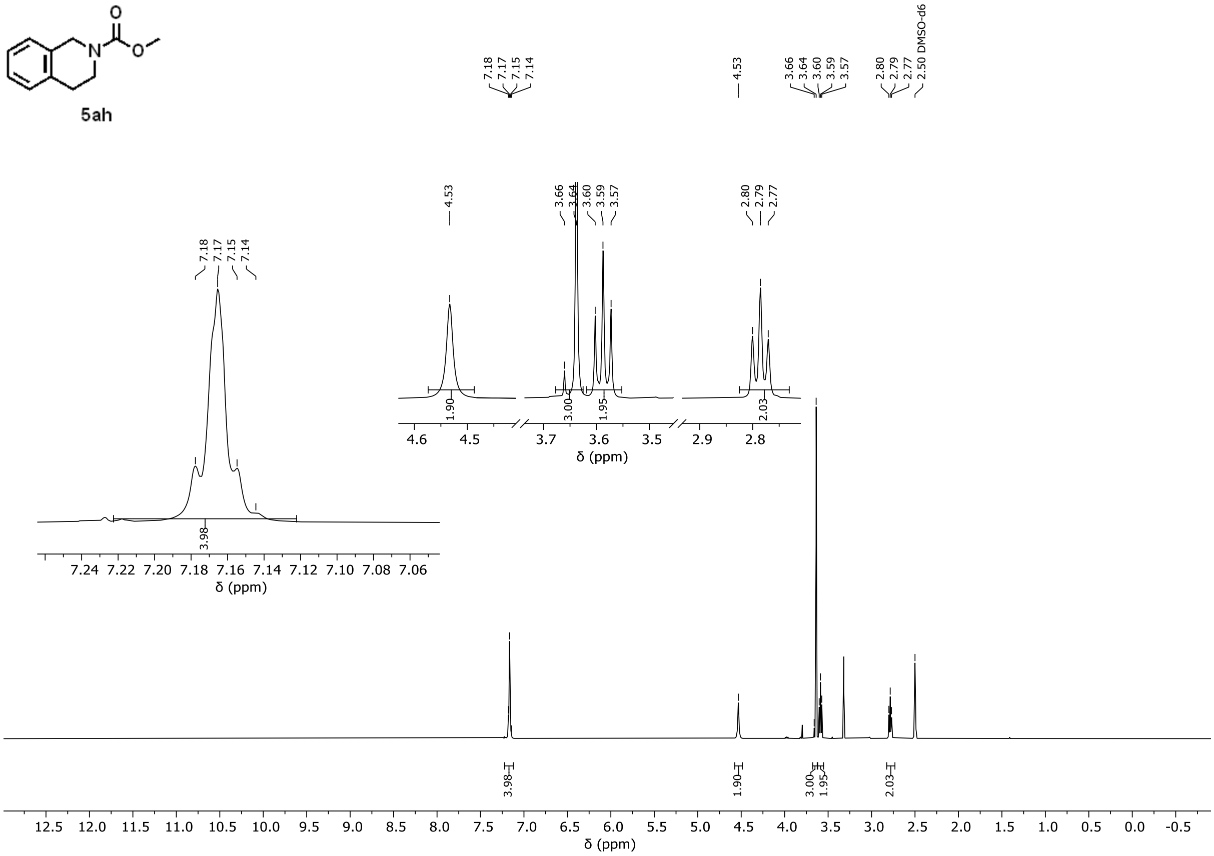

Figure **S132**: ^{1}H NMR (400 MHz, DMSO-d$_6$) of **5ah**.

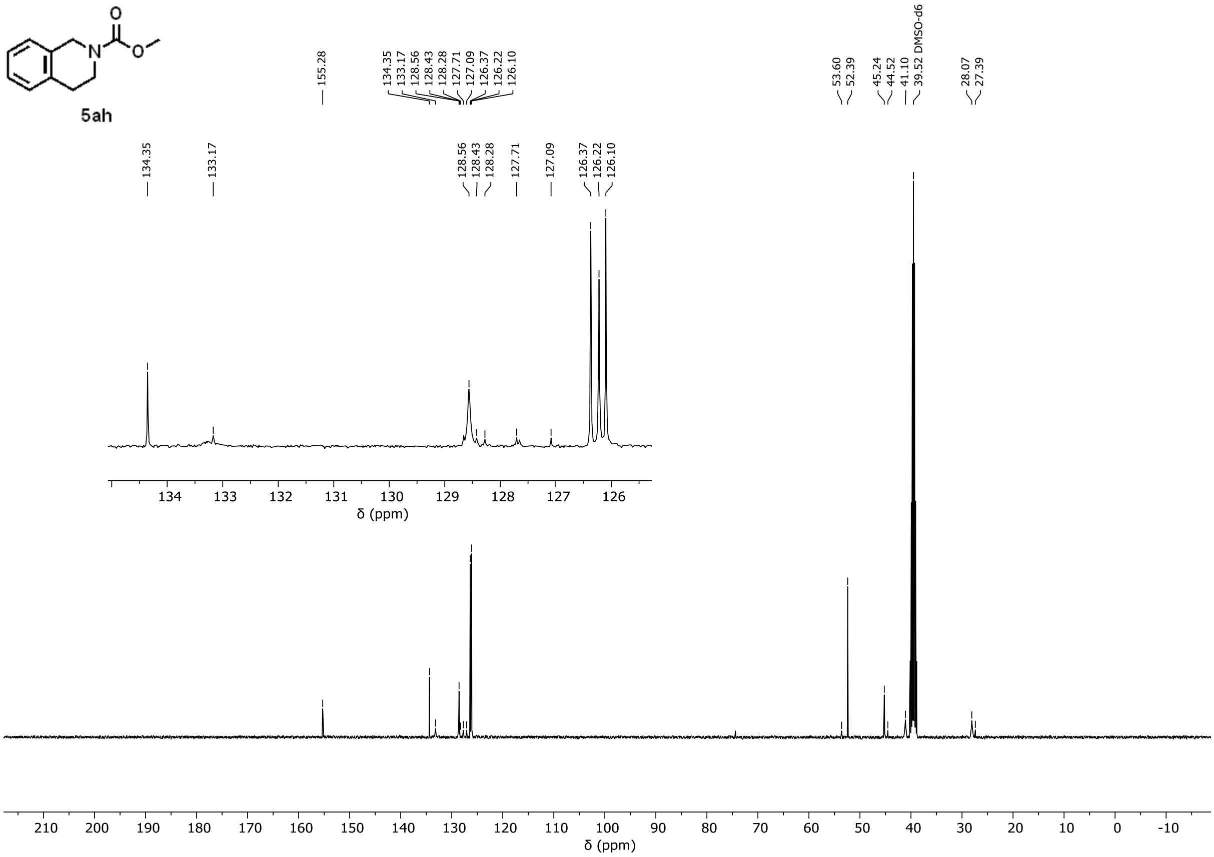

Figure **S133**: ^{13}C NMR (101 MHz, DMSO-d$_6$) of **5ah**.

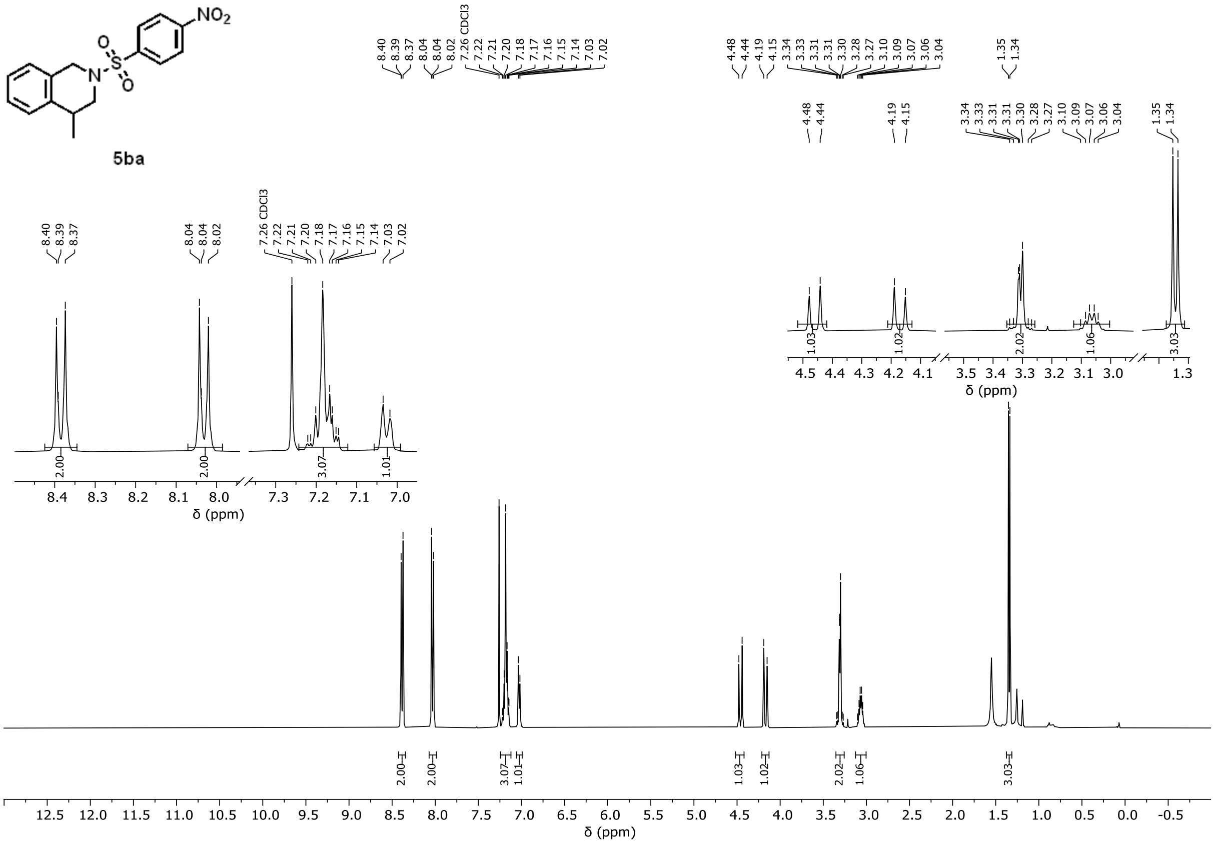

Figure **S134**: ^{1}H NMR (400 MHz, $CDCl_3$) of **5ba**.

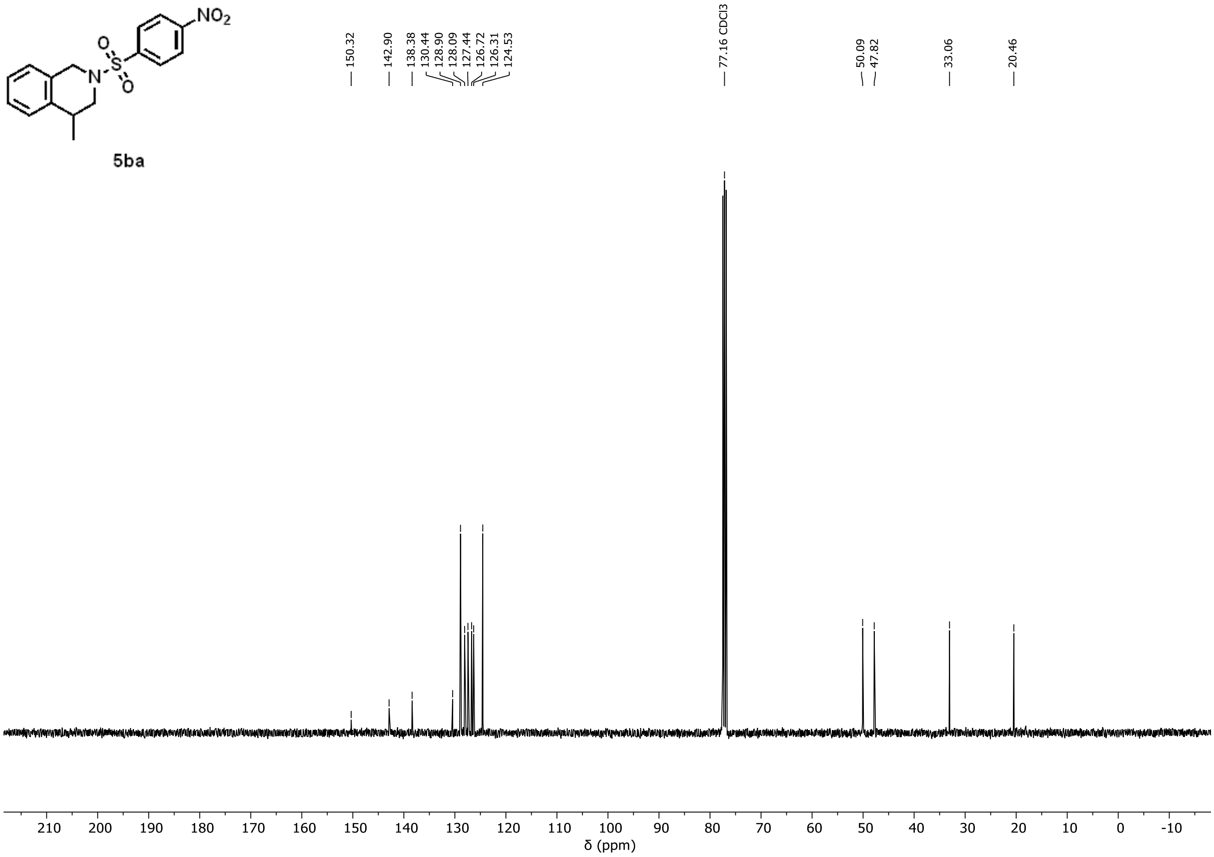

Figure **S135**: ^{13}C NMR (101 MHz, $CDCl_3$) of **5ba**.

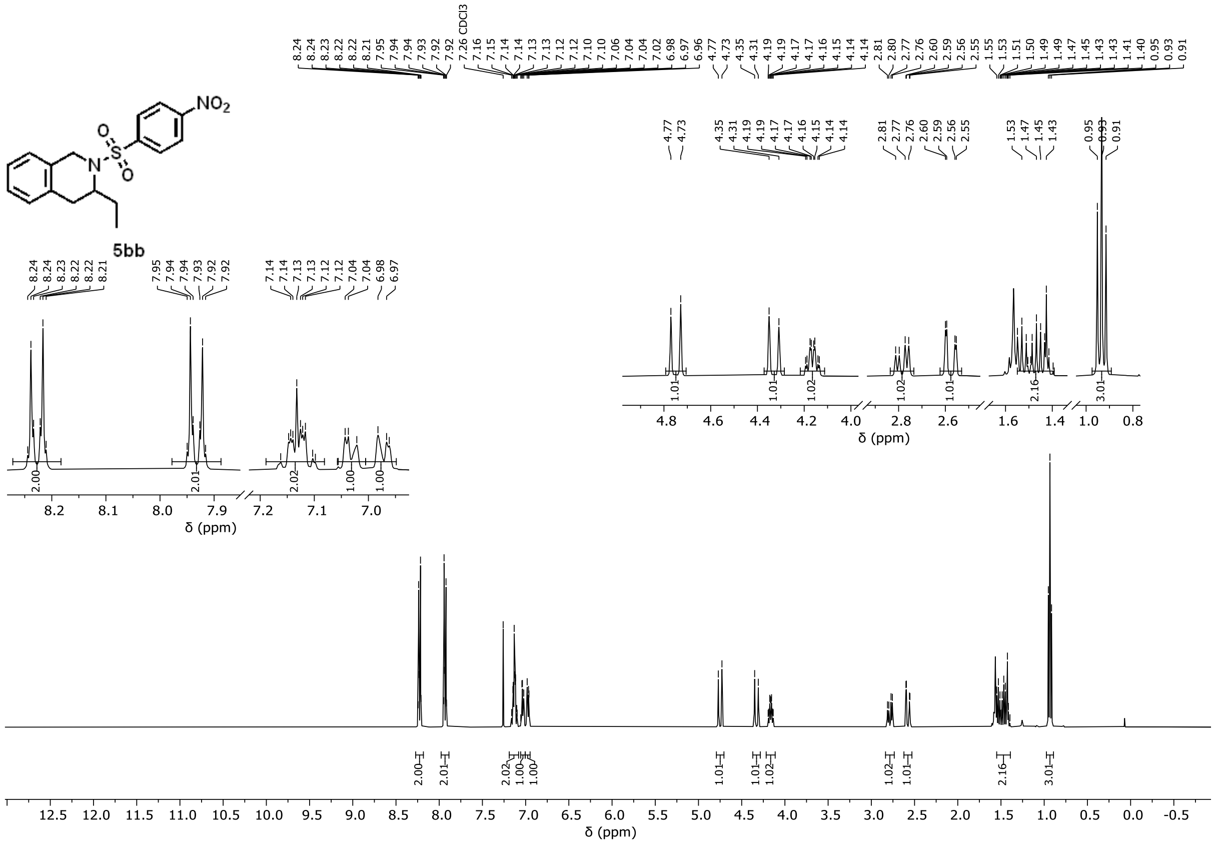

Figure **S136**: ^{1}H NMR (400 MHz, $CDCl_3$) of **5bb**.

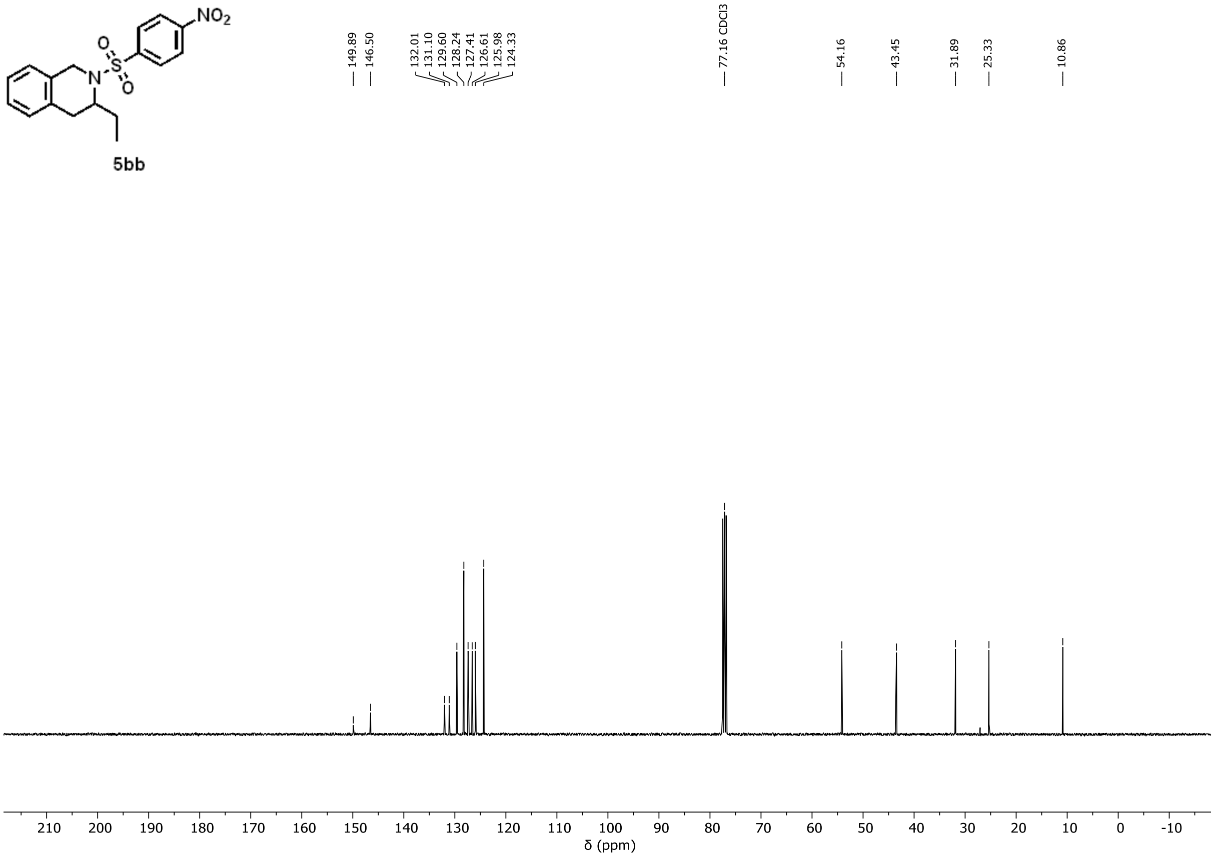

Figure **S137**: ^{13}C NMR (101 MHz, $CDCl_3$) of **5bb**.

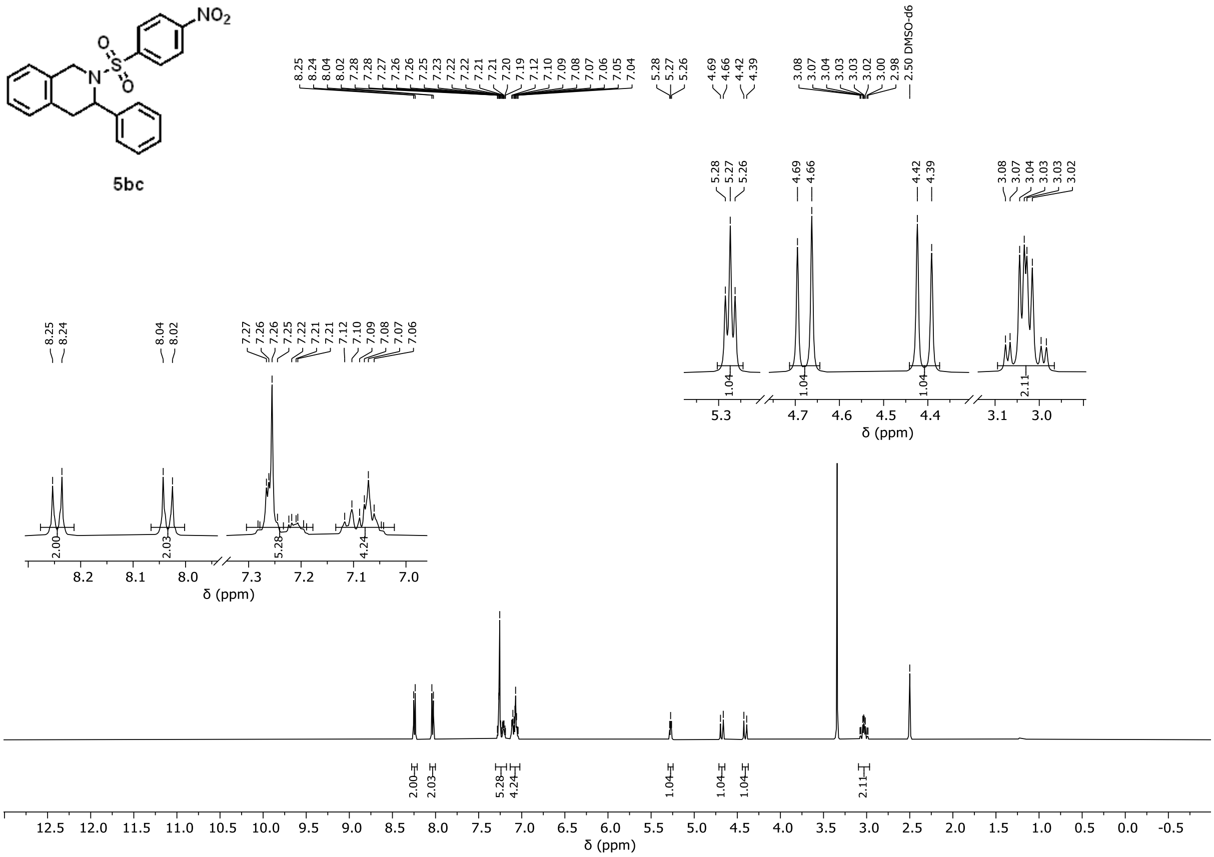

Figure **S138**: ^{1}H NMR (400 MHz, DMSO-d$_6$) of **5bc**.

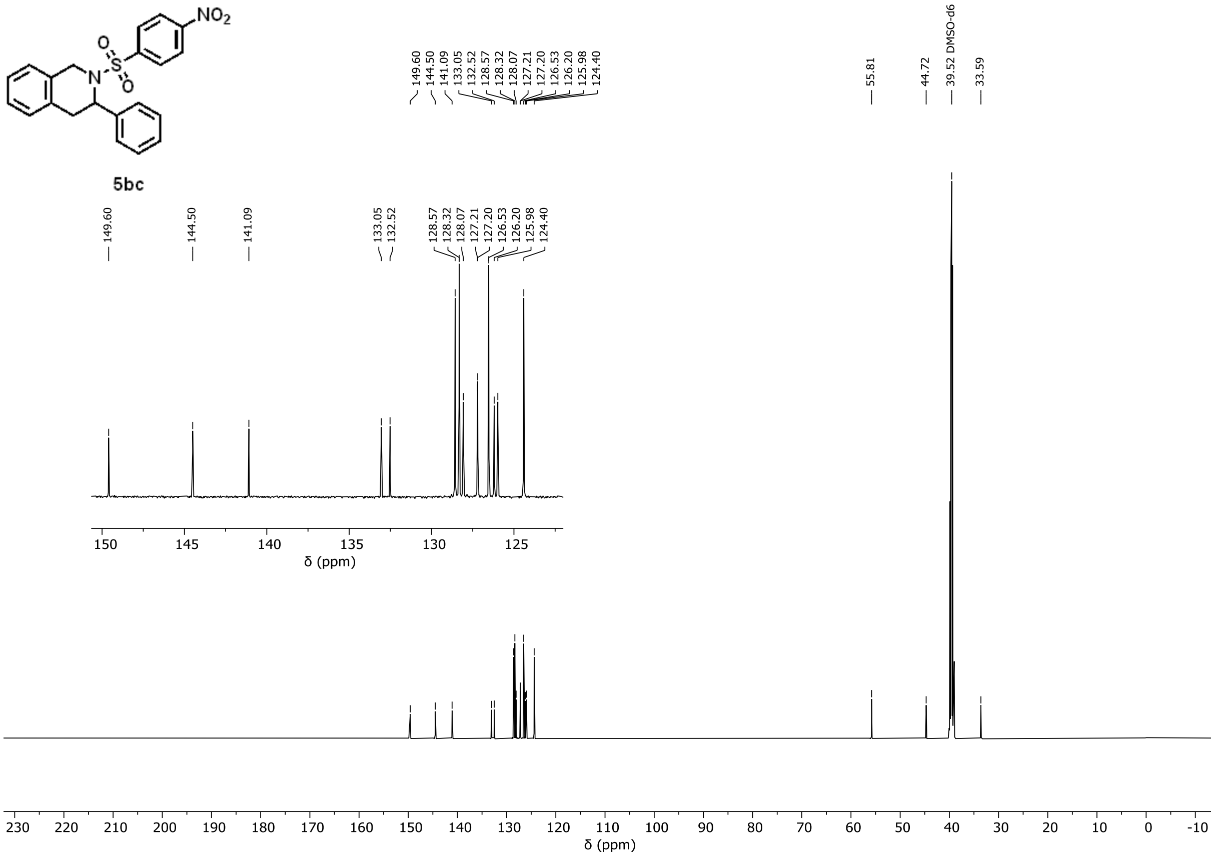

Figure **S139**: ^{13}C NMR (101 MHz, DMSO-d$_6$) of **5bc**.

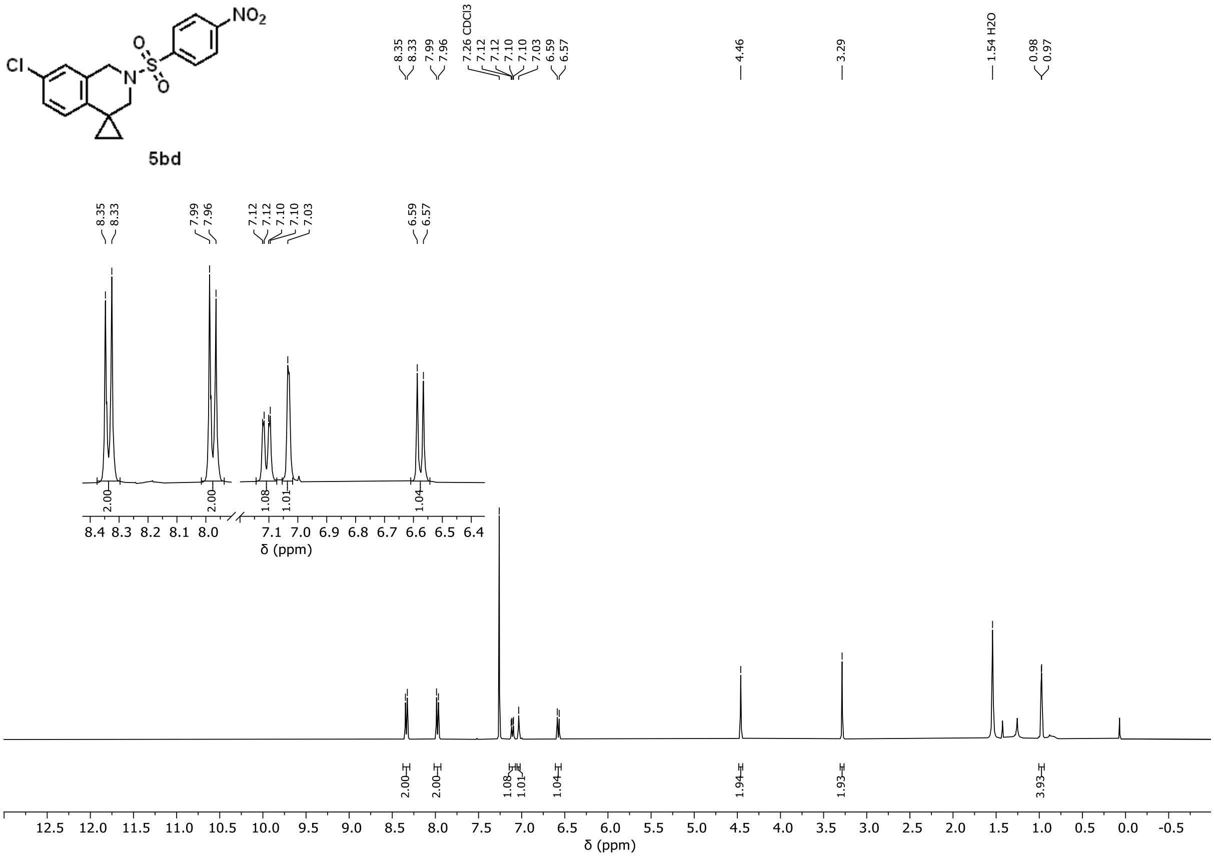

Figure **S140**: ^{1}H NMR (400 MHz, $CDCl_3$) of **5bd**.

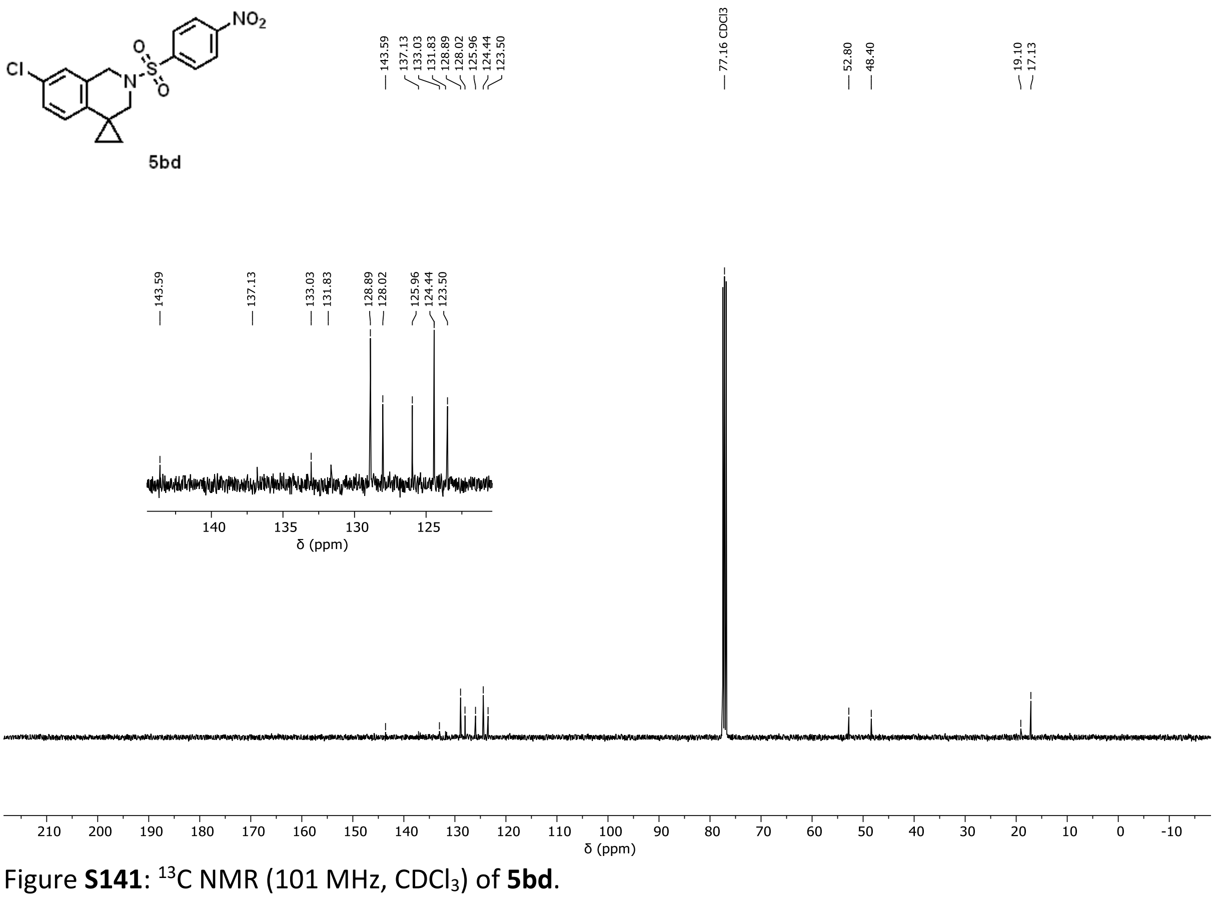

Figure **S141**: ^{13}C NMR (101 MHz, $CDCl_3$) of **5bd**.

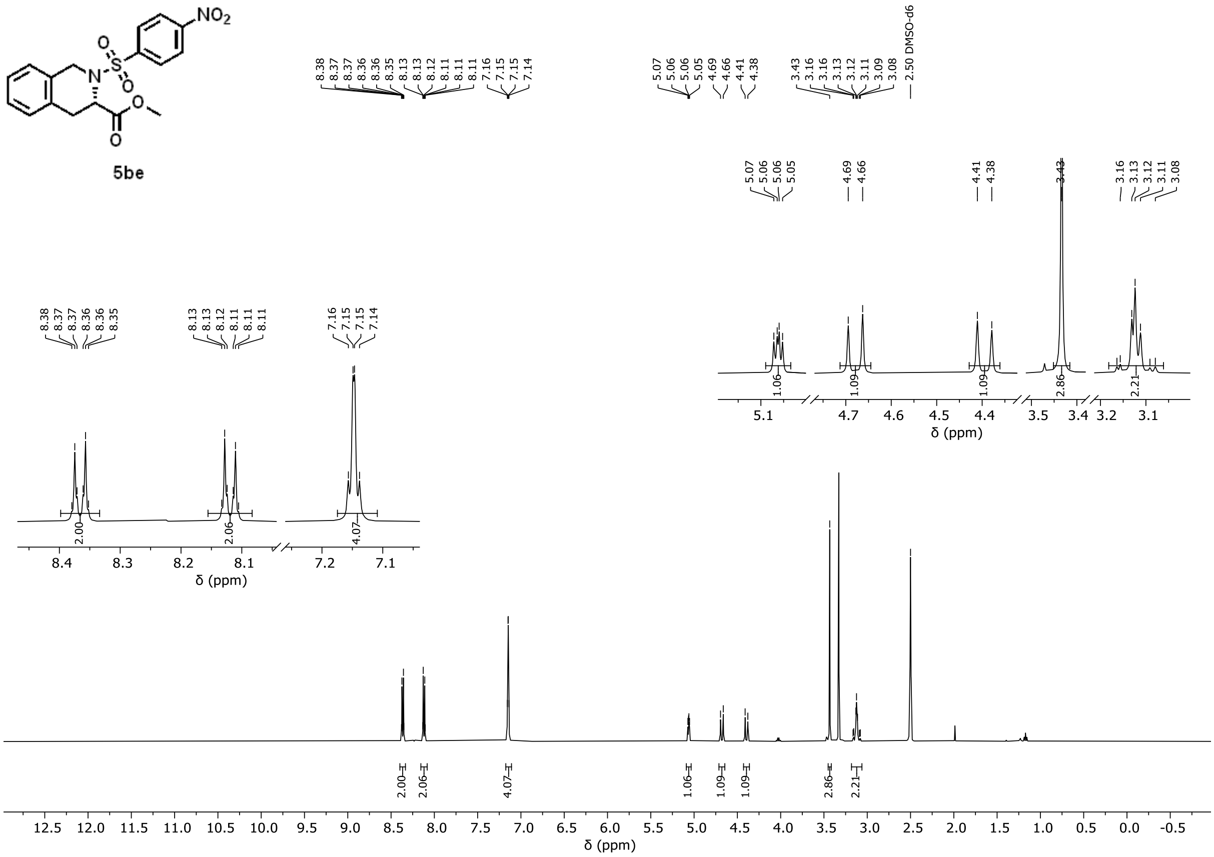

Figure **S142**: ^{1}H NMR (400 MHz, DMSO-d_6) of **5be**.

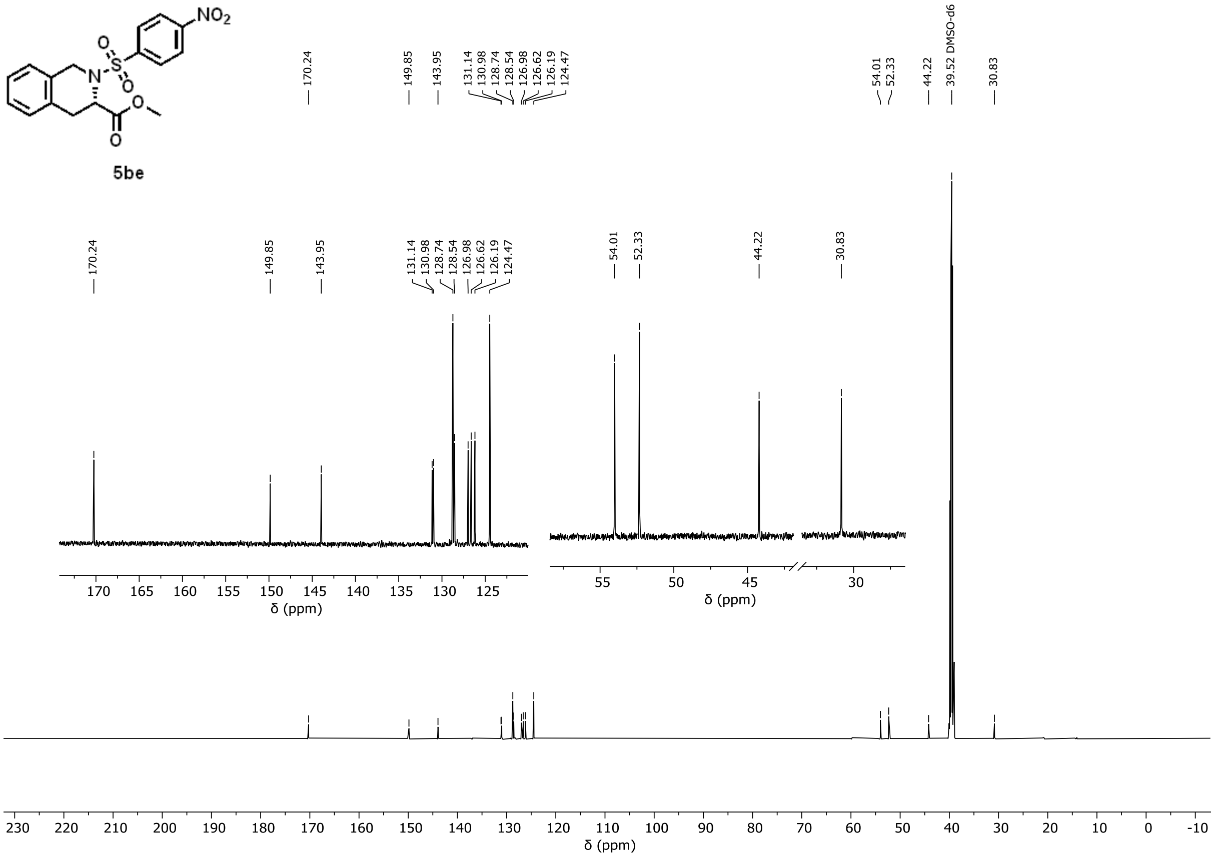

Figure **S143**: ^{13}C NMR (101 MHz, DMSO-d_6) of **5be**.

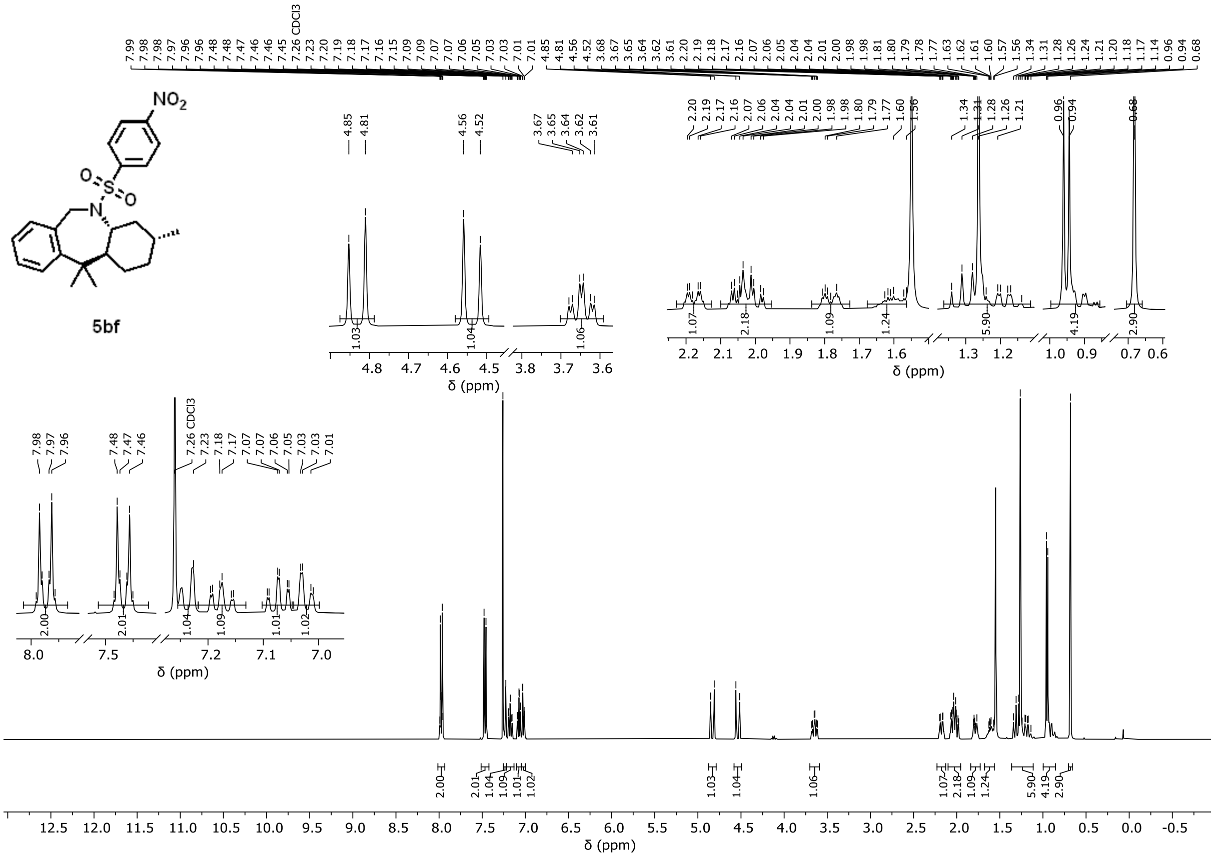

Figure **S144**: ^{1}H NMR (400 MHz, $CDCl_3$) of **5bf**.

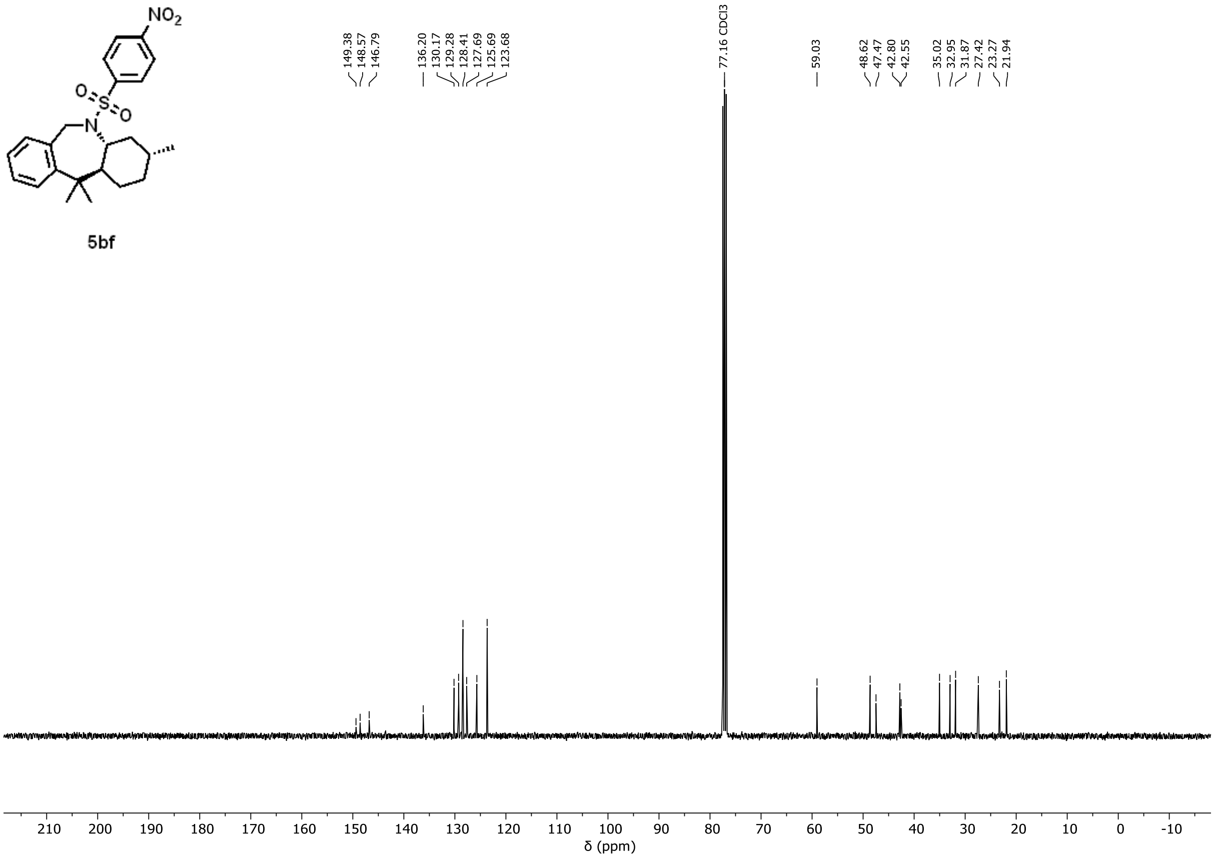

Figure **S145**: ^{13}C NMR (101 MHz, $CDCl_3$) of **5bf**.

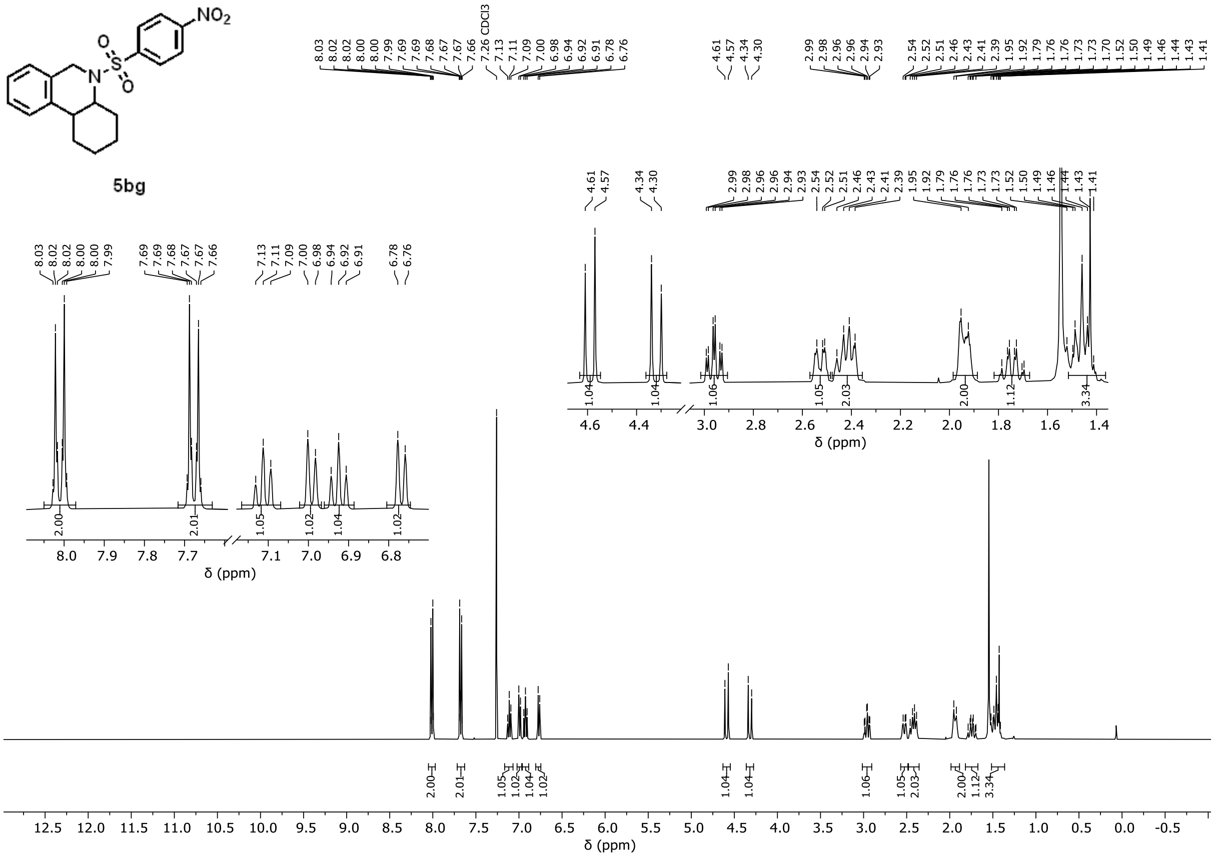

Figure **S146**: ^{1}H NMR (400 MHz, $CDCl_3$) of **5bg**.

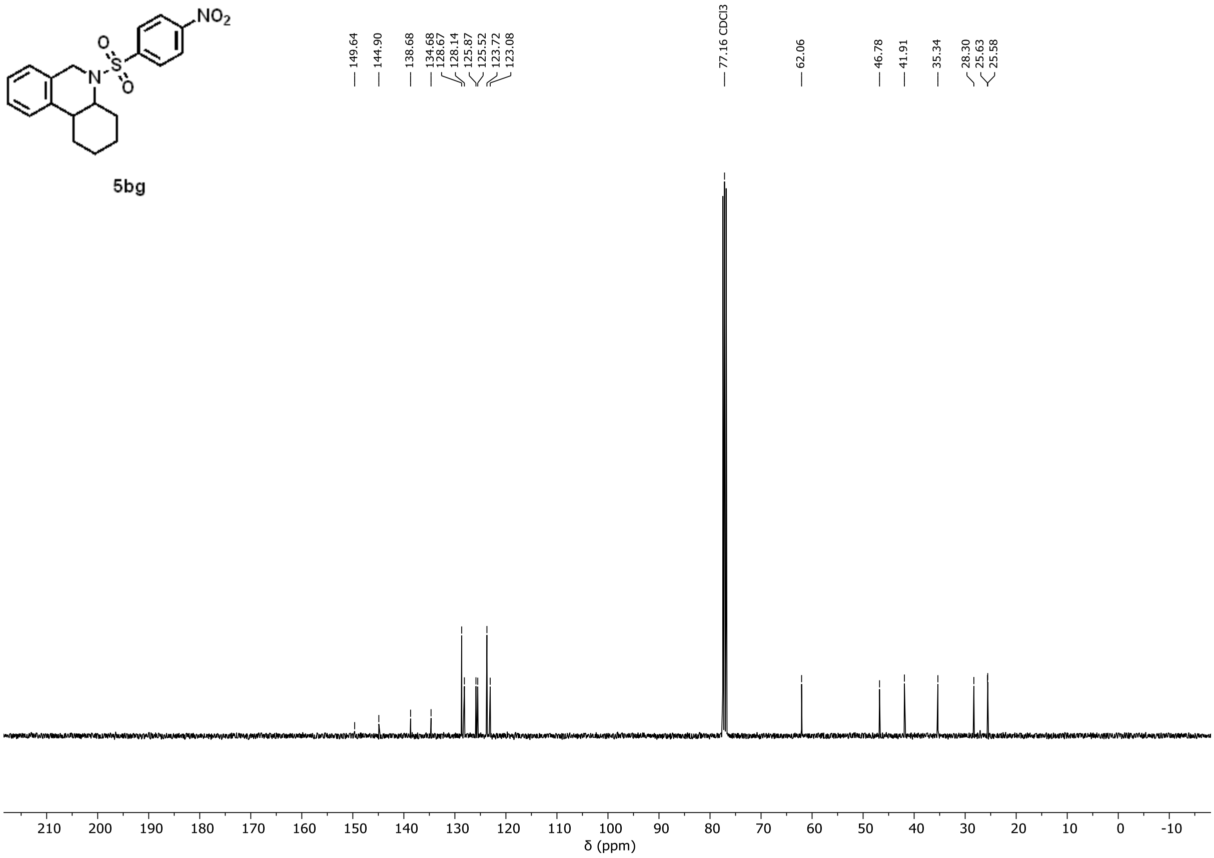

Figure **S147**: ^{13}C NMR (101 MHz, $CDCl_3$) of **5bg**.

9 References

[1] Gütz, C.; Klöckner, B.; Waldvogel, S.R. *Electrochemical Screening for Electroorganic Synthesis, Org. Process Res. Dev.* **2016***, 20,* **1***,* 26–32.

[2] Pollok, D.; Gleede, B.; Stenglein, A.; Waldvogel, S.R. *Preparative Batch-Type Electrosynthesis: A Tutorial, Aldrichimica Acta* **2021**, *54*, **1**, 3–15.

[3] Dey, S.; Schepmann, D.; Wünsch, B. *Role of the phenolic OH moiety of GluN2B-selective NMDA antagonists with 3-benzazepine scaffold, Bioorg. Med. Chem. Lett.* **2016**, *26*, **3**, 889–893.

[4] Busujima, T.; Tanaka, H. *An Efficient and Convenient Synthesis of Acyl CoA: Monoacylglycerol Acyltransferase 2 Inhibitor, 2-[2-(4-tert-Butylphenyl)ethyl]-N-[4-(3-cyclopentylpropyl)-2-fluorophenyl]-1,2,3,4-tetrahydroisoquinoline-6-sulfonamide, Heterocycles* **2016**, *92*, **3**, 470–484.

[5] Atapalkar, R. S.; Kulkarni, A. A. *Direct amidation of acids in a screw reactor for the continuous flow synthesis of amides, Chem. Comm.* **2023**, **59**, 9231–9234.

[6] Kim, H.-K.; Lee, A. *One-pot synthesis of carbamates and thiocarbamates from Boc-protected amines, Tetrahedron Lett.* **2016**, *57*, **44**, 4890–4892.

[7] Peterson, S. L.; Stucka, S. M.; Dinsmore, C. J. *Parallel Synthesis of Ureas and Carbamates from Amines and CO2 under Mild Conditions, Org. Lett.* **2010**, *12*, **6**, 1340–1343.
